# NEFA Promotes Bovine Granulosa Cell Apoptosis via Activation of the PERK/eIF2α/ATF4/CHOP Pathway

**DOI:** 10.3390/vetsci12121186

**Published:** 2025-12-11

**Authors:** Jiaxing Guo, Shenghong Zhang, Yunfei Zhai, Cheng Wang, Min Liu, Lian Li

**Affiliations:** College of Animal Science and Technology, Nanjing Agricultural University, No.1 Weigang, Nanjing 210095, China; 2023105016@stu.njau.edu.cn (J.G.); 2024105017@stu.njau.edu.cn (S.Z.); 2022205004@stu.njau.edu.cn (Y.Z.); 2023805121@stu.njau.edu.cn (C.W.); 2024805118@stu.njau.edu.cn (M.L.)

**Keywords:** non-esterified fatty acids, bovine granulosa cells, lipotoxicity, endoplasmic reticulum stress

## Abstract

Previous studies have identified oxidative stress and inflammatory responses in granulosa cells (GCs) of periparturient dairy cows. However, whether non-esterified fatty acids (NEFA)-induced endoplasmic reticulum (ER) stress is involved in GC apoptosis remains unclear. In this study, treatment with NEFA (0.9 mM, 24 h) activated the ER stress pathway. This was evidenced by increased expression of both CHOP and GRP78. Furthermore, upregulation of pro-apoptotic factors BAX and Caspase-3 and downregulation of the anti-apoptotic factor Bcl-2 were observed. Pretreatment of GCs with 4-phenylbutyric acid (4-PBA, 2.5 mM, 2 h) reversed the ER stress and apoptotic effects. This suggests that NEFA-induced apoptosis is mediated through activation of the PERK pathway of ER stress, and that 4-PBA alleviates this effect. Furthermore, targeted metabolomics revealed disruptions in lipid and hormone metabolism in GCs following NEFA treatment. Analysis revealed an increase in the levels of 26 types of fatty acids, while a decrease was detected in the levels of 3 types of fatty acids. In summary, NEFA induces ER stress and disrupts intracellular fatty acid, ultimately leading to cell apoptosis. Our findings offer valuable insights for developing strategies to regulate follicular development in dairy cows and mitigate the impacts of postpartum negative energy balance (NEB).

## Simple Summary

Negative energy balance (NEB) in postpartum dairy cows negatively impacts reproductive performance. Elevated levels of non-esterified fatty acids (NEFA), key metabolites during the NEB state, compromise ovarian cell function, disrupt hormone secretion, and induce oxidative stress, endoplasmic reticulum (ER) stress within ovaries. These NEFAs induce endoplasmic reticulum (ER) stress, activating the PERK pathway which in turn triggers apoptosis in follicular granulosa cells (GCs), and concurrently disrupt ovarian function by inducing oxidative stress. Targeted metabolomics confirmed widespread metabolic disruption, with significant changes in 29 fatty acids. These findings aim to develop new approaches for regulating reproduction in early-lactation dairy cows, thereby improving fertility and advancing our theoretical understanding.

## 1. Introduction

Impaired fertility in high-yielding dairy cows has emerged as a critical issue in dairy farming systems [1]. In high-yielding Holstein cows, a negative energy balance (NEB) during early lactation is an adaptive metabolic state. However, when the magnitude and duration of NEB are sufficient to induce significant hepatic oxidative stress and immune dysregulation, it precipitates reproductive impairments including attenuated estrus expression, delayed ovulation, and reduced conception rates [2]. In dairy production, NEB typically occurs during the peripartum period (around calving) and early lactation phase, resulting from reduced dry matter intake (DMI) coupled with suddenly increasing milk yield [3]. To meet energy demands, dairy cows mobilize adipose tissue reserves via lipolysis, which rapidly increases circulating levels of metabolites such as non-esterified fatty acids (NEFA) [4,5]. Studies demonstrate that circulating NEFA concentrations in dairy cows remain below 0.20 mM during the late lactation and dry period, but escalate to exceed 0.75 mM by 10 days postpartum [6]. Although NEB is a normal physiological process to which dairy cows must adapt, high-producing cows often fail to cope with this metabolic challenge. This failure can subsequently lead to reproductive issues, thereby ultimately undermines the economic returns of dairy operations [7]. Rectal examinations of anestrous or delayed-ovulation cows reveal ovarian quiescence, characterized by normal ovarian morphology but impaired follicular growth leading to atresia [4,8,9].

During antral follicular development, granulosa cells (GCs) play a key role in regulating follicular growth and oocyte maturation through bidirectional communication with both oocytes and somatic cells [10,11]. In early lactation dairy cows, the follicular microenvironment is critical for maintaining GCs function. Disruption of this microenvironment disrupts normal follicular development. NEFA plasma concentrations rapidly equilibrate follicular fluid concentrations and can disrupt follicular microenvironment [12,13]. It is widely believed that excessive NEFA resulting from NEB may impair oocyte development, and GC apoptosis is a major factor leading to follicular atresia [14]. Hence, we focus on the role of excessive NEFA and its associated pathways related to GC apoptosis [15,16]. Targeted metabolomic analysis of adipose tissue in periparturient dairy cows revealed that subclinical ketosis in postpartum cows is linked to alterations in polyunsaturated fatty acids (PUFAs) [17]. In previous studies, specific PUFAs (such as linolenic acid, arachidonic acid, α-linolenic acid, and eicosapentaenoic acid) have been demonstrated to possess pro-apoptotic properties [18,19].

Elevated concentrations of NEFA may induce lipotoxicity, altering the follicular microenvironment to expose GCs to stress conditions. This consequently may trigger both apoptosis and functional dysregulation in GCs [20,21,22]. Similarly, in vivo studies using high-fat diet mouse models revealed that lipid accumulation in ovarian cells contributes to increased apoptosis in both GCs and cumulus cells [23]. Complementarily, in vitro experiments demonstrated that palmitic acid treatment of porcine GCs directly causes reduced cell proliferation and upregulation of apoptosis-associated proteins [24]. Studies on bovine granulosa cell (BGCs) cultures indicate that elevated NEFA concentrations suppress cell proliferation and dysregulate steroidogenesis [20]. What specific mechanisms underlie NEFA-induced lipotoxic damage in GCs cultured in vitro?

Our prior research demonstrated that NEFA promotes reactive oxygen species (ROS) accumulation, activating oxidative stress pathways and induces apoptosis in BGCs through the AKT/FoxO1 signaling axis [22]. However, lipotoxicity extends beyond oxidative stress induction, concurrently triggering other cellular stress responses. Specifically, NEFA-induced lipotoxicity impairs mitochondrial structure and function, which subsequently provokes endoplasmic reticulum (ER) stress activation [25]. In obesity-induced ovarian dysfunction in mice, lipotoxicity impairs estrogen biosynthesis in GCs by activating the ER stress pathway [26]. In vitro cultured bovine hepatocytes exposed to NEFA exhibit robust induction of ER stress, characterized by significantly elevated expression of hallmark ER stress markers PERK, GRP78, and CHOP [27].Critically, this effect is dose-dependently alleviated by treatment with the 4-phenylbutyric acid (4-PBA), an inhibitor of ER stress [28]. Although NEFA has been confirmed to induce ER stress, the specific signaling pathways through which it mediates stress responses and induces apoptosis in bovine GCs have not been systematically studied.

Elucidating the mechanisms of lipotoxic damage in GCs of follicles from dairy cows experiencing NEB provides a theoretical foundation for understanding aberrant follicular development during early lactation. We hypothesized that elevated NEFA levels and the persistent stimulation by low concentrations of NEFAs both lead to fatty acid buildup within cells. This elevation also causes sustained endoplasmic reticulum stress. Through the unfolded protein response (UPR) pathway, such prolonged stress can ultimately induce apoptosis. Therefore, this study employs BGCs to investigate the regulatory mechanisms underlying NEFA-induced lipotoxic damage in the ovary. We aim to investigate how elevated levels of NEFA affect lipid metabolism and steroidogenesis in GCs, and to quantify genes and proteins expression within the PERK/eIF2α/ATF4/CHOP signaling pathway and elucidate the underlying mechanism by which NEFA-induced activation of the PERK-mediated ER stress cascade promotes cellular apoptosis.

## 2. Materials and Methods

### 2.1. Ethical Statement

The research protocols were approved by the Institutional Animal Care and Use Committee (IACUC) of Nanjing Agricultural University, China (animal protocol approval number: EAE-GZU-2022-T083; date: 4 March 2024), all ovaries for experimental use were obtained from local slaughterhouses.

### 2.2. Experimental Animals and Cell Culture

Ovaries were collected from dairy cows with a body condition score of approximately 2.5. Follicles from these ovaries were undergoing active development but had not yet ovulated. Ovaries were collected from slaughterhouses near Nanjing and transported to the laboratory within 3 h in thermos containers filled with pre-warmed physiological saline (0.9% NaCl). Upon arrival, ovaries were rinsed with physiological saline (pre-warmed to 37 °C and supplemented with 100 U/mL penicillin and 100 μg/mL streptomycin) to remove blood residues. Follicular fluid was aspirated from follicles 3–6 mm in diameter using sterile syringes, and centrifuged at 1200 rpm (~250× *g*) for 5 min. After discarding the supernatant, the pellet was washed twice with PBS (SH30256.01, HyClone, Logan, UT, USA) under identical centrifugation conditions. Cells were then resuspended in complete culture medium—DMEM/F12 (Gibco Life Technologies, Grand Island, NY, USA) supplemented with 10% fetal bovine serum (FBS; 04-0011Acs, Sigma-Aldrich, St. Louis, MO, USA) and 1% penicillin-streptomycin (100 U/mL penicillin, 100 μg/mL streptomycin, Solarbio, Beijing, China)—and plated in T25 culture flasks. Following 24 h incubation at 37 °C with 5% CO_2_, non-adherent cells were removed by gentle washing, leaving adherent ovarian GCs for subsequent experiments. Cultures were maintained with medium replacement every 48 h. At 90–100% confluency, cells were detached using 0.25% trypsin-EDTA (Gibco Life Technologies, CA, USA), centrifuged (1200 rpm, 5 min), resuspended, diluted to appropriate densities, and passed into new T25 culture flask. To prevent GCs luteinization, the subsequent experiments were conducted within one week after transferring the cells into T25 flasks. In the follow-up experiments, cells were seeded at the following densities: 1 × 10^6^ cells per well for 6-well plates and 1 × 10^4^ cells per well for 96-well plates. Our research group has previously conducted immunofluorescence identification of bovine ovarian granulosa cells [29].

### 2.3. The NEFA Stock Solution

The 10 mM NEFA stock solution was prepared by first dissolving 1.12 g potassium hydroxide (KOH) in 200 mL ultrapure water to produce a 0.1 M KOH solution. Subsequently, palmitic acid (81.8 mg, P5585, Sigma-Aldrich, St. Louis, MO, USA), linoleic acid (13.74 mg, L1376, Sigma-Aldrich, St. Louis, MO, USA), palmitoleic acid (14.89 μL, P9417, Sigma-Aldrich, St. Louis, MO, USA), oleic acid (134.83 μL, O1008, Sigma-Aldrich, St. Louis, MO, USA), and stearic acid (15.18 μL, S4751, Sigma-Aldrich, St. Louis, MO, USA) were added and dissolved in the alkaline solution at 60 °C with constant stirring [21,30]. After the acids were fully dissolved, the pH was adjusted to 7.2 using 1 M hydrochloric acid (HCl). The solution was then aliquoted and stored at −20 °C. Diluting the stock solution in pre-warmed culture medium immediately prior to use.

### 2.4. Cell Viability Analysis by CCK-8 Assays

For NEFA cytotoxicity assessment: GCs were plated in 96-well plates at a density of 1 × 10^5^ cells per well and treated with NEFA at concentration gradients of 0, 0.3, 0.6, 0.7, 0.8, 0.9, and 1.0 mM. After 24 h of treatment, The Cell Counting Kit-8 (CCK-8, Institute of Bioengineering, Nanjing, China) working solution was added to each well. Following 4 h of incubation at 37 °C, absorbance was measured at 450 nm using a microplate reader. Cell viability was determined after treatment with varying concentrations of NEFA. GCs cultured were divided into experimental groups: low NEFA group, 0.6 mM NEFA for 24 h; low NEFA accumulation group, 0.6 mM NEFA for 48 h; high NEFA group, 0.9 mM NEFA for 24 h and control group, no NEFA treatment. Cell viability was calculated according to the standard formula:

Viability (%) = [OD (treatment) − OD (blank)]/[OD (control) − OD (blank)] × 100%

For 4-PBA (AbMole BioScience, Nanjing, China) cytoprotective evaluation: GCs (1 × 10^5^ cells/well in 96-well plates) were pretreated with 4-PBA at concentrations of 0, 1.0, 2.5, 5.0, and 7.5 mM for 2 h. CCK-8 working solution was subsequently added without removing 4-PBA. Afterward, cells were treated with NEFA for 24 h under the same conditions. CCK-8 working solution was then added, and absorbance was measured after incubation. Cell viability was calculated using the same standardized formula. For subsequent investigations, experimental cohorts were established as follows: Control group; NEFA group (0.9 mM, 24 h); 4-PBA group (2.5 mM, 2 h); NEFA+4-PBA group (2.5 mM 4-PBA pretreatment for 2 h followed by 0.9 mM NEFA for 24 h).

### 2.5. Imaging of Intracellular Lipid Droplets

The Oil Red O working solution (Beyotime, Shanghai, China) was prepared immediately prior to use by mixing Oil Red O stock solution with its diluent at a 3:2 (*v*/*v*) ratio. The mixture was filtered through a 0.44 μm membrane filter and pre-warmed to 37 °C for no more than two hours before application. GCs grown on glass coverslips were subjected to experimental treatments: low NEFA group, 0.6 mM NEFA for 24 h; low NEFA accumulation group, 0.6 mM NEFA for 48 h; high NEFA group, 0.9 mM NEFA for 24 h and control group, no NEFA treatment (same as below). Following treatment completion, culture medium was carefully aspirated, followed by a single wash with sterile phosphate-buffered saline (PBS). For fixation, 500 μL of 4% paraformaldehyde solution (Beyotime, Shanghai, China) was added per well of 6-well plates for 20 min at room temperature, followed by two PBS washes. Cells were then briefly covered with pre-stain rinse solution for 20 s before complete removal. Subsequently, 1 mL of pre-warmed Oil Red O working solution was applied per well and incubated for 20 min. Following removal of the staining solution, cells underwent a 30 s rinse solution treatment and a 20 s PBS wash. Nuclear counterstaining was performed using hematoxylin (Beyotime, Shanghai, China) for 5 min, followed by a 10 min PBS wash and two differentiation steps with 75% ethanol. Finally, coverslips were mounted onto glass slides and imaged using a virtual microscope scanning system (Dotslide, OLYMPUS, Tokyo, Japan).

### 2.6. Reactive Oxygen Species (ROS) Fluorescence Detection

A 10 μM DCFH-DA (Beyotime, Shanghai, China) working solution was prepared in serum-free medium. GCs cultured on coverslips were divided into experimental groups: low NEFA group, 0.6 mM NEFA for 24 h; low NEFA accumulation group, 0.6 mM NEFA for 48 h; high NEFA group, 0.9 mM NEFA for 24 h and control group, no NEFA treatment [21]. Following treatments, culture medium was carefully aspirated and followed by two washes with sterile PBS. Cells were then stained with the DCFH-DA working solution for 30 min in the dark at 37 °C. After incubation, unincorporated dye was removed through three thorough PBS washes. Coverslips were mounted using anti-fade mounting medium (Beyotime, Shanghai, China) and immediately imaged under a laser scanning confocal microscope (excitation/emission: 488/525 nm) with a 10× objective.

### 2.7. Apoptosis Analysis by Flow Cytometry

Following experimental treatments, culture medium from each well was collected into labeled centrifuge tubes. After one PBS wash, the wash solution was collected. For cell detachment, 500 μL of EDTA-free trypsin (Gibco, CA, USA) solution was added per well of the 6-well plates. After incubation, the original collected medium was reintroduced to neutralize trypsin activity. The cell suspension was centrifuged at 1500 rpm (∼300× *g*) for 5 min. Following centrifugation, the supernatant was discarded, and the pellet was washed twice with PBS using identical centrifugation parameters. Cells were resuspended in 100 μL of 1× Annexin V Binding Buffer. Immediately prior to analysis, 5 μL Annexin V-FITC and 5 μL propidium iodide (PI) (Biosharp, Nanjing, China) were added to each sample. Following 10 min of incubation in the dark at room temperature, samples were analyzed within 1 h using a flow cytometer (FACSCalibur, BD Biosciences, Bedford, MA, USA). Data processing was performed with FlowJo software (Version 10.10.0).

### 2.8. Targeted Metabolomic Profiling of Lipids

Targeted metabolomics was employed to assess the impact of NEFA treatment (0.9 mM NEFA, 24 h) on 29 fatty acids in BGCs. BGCs served as the experimental model, divided into control and treatment groups. Treatment group cells were cultured in medium supplemented with high-concentration NEFA (0.9 mM), while control cells were maintained in standard culture medium. After 24 h of incubation, cells were washed twice with ice-cold PBS to remove residual medium. Culture dishes were then transferred to an ice bath, and 200 μL of pre-chilled PBS was added. Using a sterile cell scraper, adherent cells were dislodged and concentrated along one side of the tilted dish. The cell suspension was transferred to pre-cooled centrifuge tubes and centrifuged at 200–1000× *g* for 5 min at 4 °C. Following supernatant removal, the cell pellet was resuspended and aliquoted into cryogenic thread-cap vials (pre-chilled to −80 °C). Samples underwent flash-freezing in liquid nitrogen for 60 min before long-term storage at −80 °C. For subsequent analysis, frozen samples were shipped on dry ice to LC-Bio Technology Co., Ltd. (Hangzhou, China) for targeted metabolomic profiling. Chemicals and reagents: LC-MS grade methanol (MeOH), ethyl acetate and ammonium acetate were purchased from CNW (Shanghai, China). Primary water was purchased from WATSON and used in all experiments. All of the standards were purchased from Alta Scientific Co., Ltd. (Tianjin, China). Sample Preparation: Add 500 μL of saline into the sample tube, vortex and mix well, ultrasonic extraction for 10 min, then add 10 μL of 200 ng/mL internal standard solution and 1200 μL of ethyl acetate, vortex and mix well. The extract was vortexed for 10 min, incubated on ice for 10 min, centrifuged (20,000 rcf, 4 °C) for 15 min, and the supernatant was freeze-dried and re-dissolved with 100 μL of the initial mobile phase (1 mM aqueous ammonium acetate: methanol = 6:4), centrifuged (20,000 rcf, 4 °C) for 15 min, and then the supernatant was transferred to the vials for LC-MS/MS detection and analysis. LC-MS/MS Conditions: The separation and quantification of the target compounds were performed using a Waters Acquity UPLC system coupled with an AB Sciex Triple Quad 5500+ mass spectrometer (Hangzhou, China). The analytical conditions were as follows. UPLC Conditions: The chromatographic separation was carried out on an Agilent Poroshell 120 EC-C18 2.7 μm (2.1 × 100 mm) column (Hangzhou, China). The mobile phase consisted of (A) 1 mmol/L aqueous ammonium acetate solution and (B) methanol. Injection volume: 10 μL, Temperature: 40 °C. ESI-MS/MS Conditions: the data was acquired under multiple reaction monitoring (MRM) mode with ESI ion source. The instrumental parameters were optimized for maximal sensitivity with the voltage and source and gas settings as follows: ion spray voltage (IS): 4500 V in positive mode and −4500 V in negative mode; curtain gas (CUR): 35 psi; collision gas (CAD): 8; temperature (TEM): 450 °C; ion source gas 1 (GS1): 60 psi; ion source gas 2 (GS2): 60 psi.

### 2.9. Total RNA Extraction and Real-Time Quantitative PCR (RT-qPCR) for Gene Expression

Substantial evidence indicates that the lipotoxicity of elevated NEFA levels triggers endoplasmic reticulum (ER) stress and ultimately leads to apoptosis. All procedures for total RNA extraction and real-time quantitative PCR (RT-qPCR) were conducted under ice-cold conditions to preserve RNA integrity. Total RNA was isolated from GCs using the TRIzol reagent (Invitrogen, Carlsbad, CA, USA) method, involving sequential addition of chloroform, isopropanol, and 75% ethanol. Post-extraction, RNA concentration and purity (A260/A280 ratio ≥ 1.8) were determined using a NanoDrop ND-2000 spectrophotometer (Thermo Fisher Scientific, Shanghai, China). Based on quantified concentrations, calculated amounts of RNA were reverse transcribed into cDNA using the HiScript III RT SuperMix for qPCR (Vazyme Biotech, Nanjing, China) according to manufacturer specifications.

Quantitative PCR amplifications were performed in triplicate using ChamQ Universal SYBR qPCR Master Mix (Vazyme Biotech, Nanjing, China). Thermal cycling conditions followed the master mix protocol. Gene-specific primers (Table 1), designed via NCBI and synthesized by Tsingke Biotechnology (Beijing, China), generated amplicons of 80–150 bp. PCR results were analyzed for relative gene expression using the comparative Ct (2^−ΔΔCt^) method, with β-actin serving as the endogenous control. All reactions included no-template controls (NTCs) and exhibited intra-assay coefficients of variation <5%.

### 2.10. Protein Extraction and Western Blot Analysis

All protein extraction procedures were performed on ice to maintain sample integrity. Cells were lysed in 200 μL of ice-cold Radio Immunoprecipitation Assay (Beyotime, Shanghai, China) supplemented with 1% protease inhibitor PMSF (Beyotime, Shanghai, China), followed by constant shaking for 30 min at 4 °C. Lysates were centrifuged at 15,000 rpm (20,000× *g*) for 15 min at 4 °C. Protein concentrations in the supernatants were quantified using the BCA assay kit (Beyotime, Shanghai, China). Concentrated protein samples were mixed with 5× SDS loading buffer at a 4:1 ratio and denatured by heating at 95 °C for 10 min in a metal heating block. Using 12% SDS-PAGE gels (GenScript, Nanjing, China), denatured proteins were resolved and subsequently transferred electrophoretically to polyvinylidene difluoride membranes (PVDF; Life Technologies, Carlsbad, CA, USA).

Membranes were blocked with rapid blocking buffer for 15 min at room temperature and washed once with 1× TBST for 15 min. Primary antibodies were applied for overnight incubation at 4 °C. Following three 15 min TBST washes, secondary antibody (horseradish peroxidase, HRP-conjugated) incubation was performed on membranes for 2 h at RT. After three additional TBST washes, protein bands were detected using the enhanced chemiluminescence substrate (Biosharp, Shanghai, China) and imaged with an ultrasensitive chemiluminescence gel imaging system. Band intensity quantification was performed using Image J software (v1.53, National Institutes of Health, Bethesda, MD, USA). The antibody specifications are listed in Table 2.

### 2.11. Statistical Analysis

Statistical analyses were performed using GraphPad Prism software (Version 9.0, GraphPad Software, San Diego, CA, USA). All experimental results are presented as mean ± standard error of the mean (SEM). Differences between two groups were assessed using Student’s *t*-test, while multiple group comparisons employed one-way analysis of variance (ANOVA) with Dunnett’s post hoc test for comparisons against a single control group. For factorial experimental designs involving two independent variables, two-way ANOVA was applied followed by Šídák’s multiple comparisons test to evaluate all pairwise interactions. All statistical tests used *p* < 0.05 as the significance threshold.

## 3. Results

### 3.1. Effect of NEFA on Cell Viability

**Figure 1:**
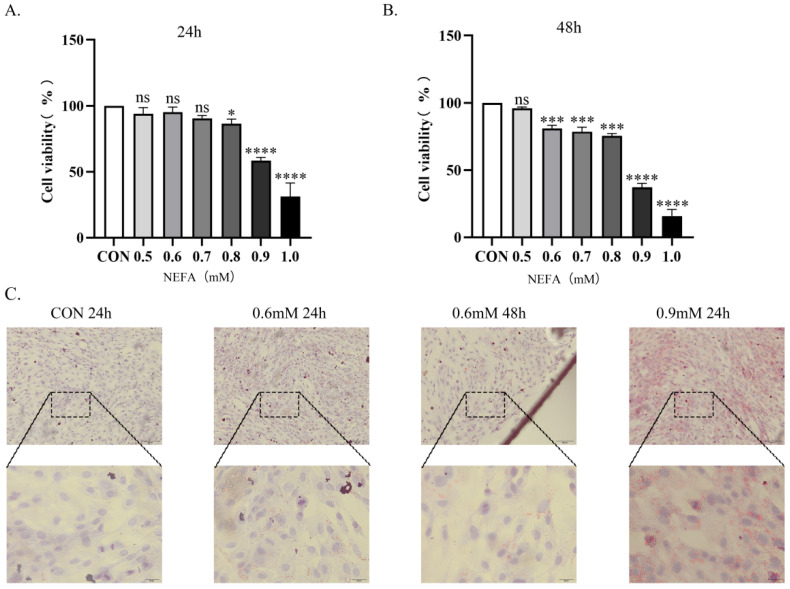
NEFA experimental concentration screening and the effect of NEFA supplementation on lipid accumulation of BGCs. (**A**) Effect of different concentrations of NEFA treatment for 24 h on the viability of BGCs. (**B**) Effect of different concentrations of NEFA treatment for 48 h on the viability of BGCs. (**C**) Light microscopy of lipid accumulation in BGC cytoplasm. Blue: Hematoxylin; Red: Oil-red-O; scale bar: 100 µm and 20 µm. All experiments were set up with three biological replications, and the statistical results were presented in the form of mean ± SEM. * *p*-value < 0.05, *** *p*-value < 0.001, **** *p*-value < 0.0001, ns, not significant. Same as below.

Experimental results show that cell viability significantly decreased after 24 h treatment with 0.9 mM NEFA compared to the control group (*p* < 0.0001, Figure 1A). Thus, 0.9 mM NEFA treatment for 24 h was selected for subsequent experiments. At 0.6 mM, cell viability remained unchanged following 24 h exposure, whereas a significant reduction in viability was observed after 48 h treatment (*p* < 0.001, Figure 1B).

### 3.2. Effects of Different NEFA Concentrations on Lipid Accumulation, ROS Production, and Apoptosis

**Figure 2:**
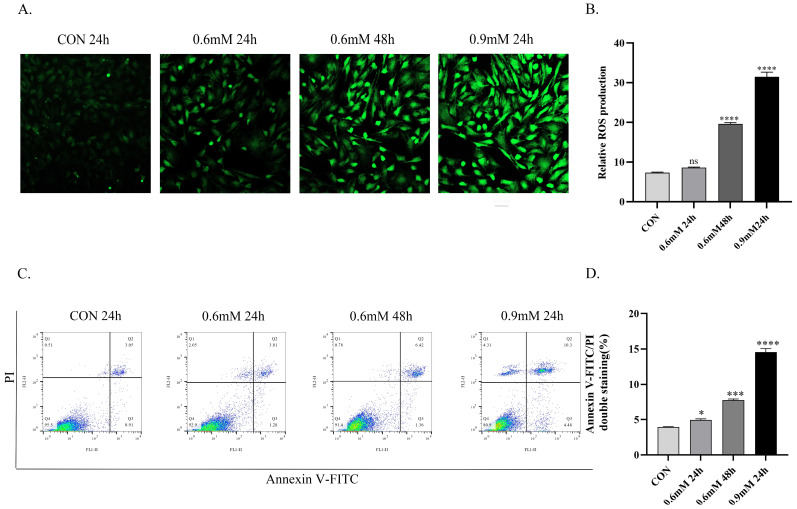
Effects of NEFA on ROS accumulation and apoptosis in different treatments. (**A**)Fluorescent microscopy visualized ROS generation using DCFH-DA staining with CON 24 h, 0.6 mM 24 h, 0.6 mM 48 h, 0.9 mM 24 h, Green: DCFH-DA, scale bar: 100 µm. (**B**) Comparison of relative fluorescence intensities between different treatment groups. (**C**) Apoptosis of BGCs analyzed by flow cytometry after treatment as in (**A**) and processed using FlowJo software. (**D**) Comparison of apoptosis rates between different treatment groups. All experiments were set up with three biological replications, and the statistical results were presented in the form of mean ± SEM. * *p*-value < 0.05, *** *p*-value < 0.001, **** *p*-value < 0.0001, ns, not significant.

Prolonged exposure to low-concentration NEFA also impacted cellular physiological functions. In Oil Red O-stained lipid droplet assays, compared to controls, the Low-NEFA group exhibited increased lipid droplet number without changes in size. The Low-NEFA cumulative exposure group showed sparsely distributed large lipid droplets. The High-NEFA group demonstrated elevated droplet count with enlarged particle size (Figure 1C). A similar trend was observed in ROS fluorescence staining with no significant difference in fluorescence intensity between Low-NEFA group and controls. Highly significant ROS accumulation in both Low-NEFA cumulative and High-NEFA groups versus controls (*p* < 0.0001, Figure 2A,B). Flow cytometric analysis using Annexin V-FITC/PI dual staining revealed, significantly increased apoptosis in the Low-NEFA group relative to controls. Apoptosis was significantly induced in both Low-NEFA cumulative and High-NEFA groups (*p* < 0.0001, Figure 2C,D).

### 3.3. Targeted Metabolomics Analysis of NEFA Effects on Fatty Acid in BGCs

**Figure 3:**
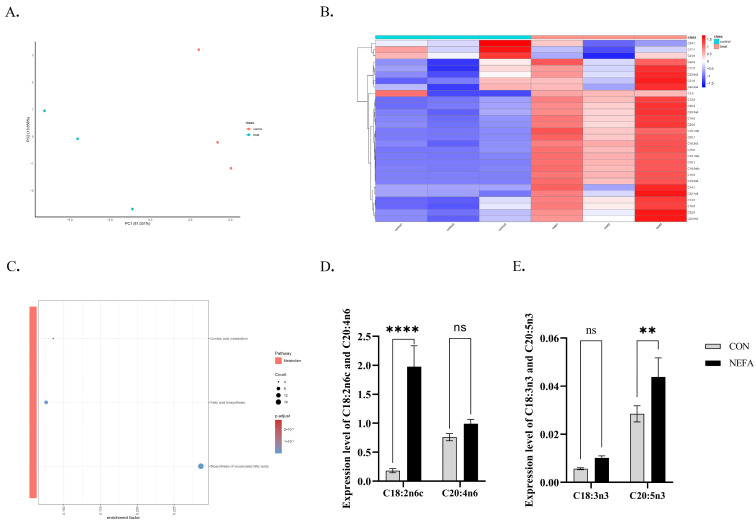
Targeted metabolomics analysis of fatty acid metabolism in BGCs following 0.9 mM NEFA treatment. (**A**) Principal component analysis of samples from CON and NEFA-treated. (**B**) Cluster heat map analysis of lipid metabolites in CON and NEFA-treated. (**C**) Lipid metabolite pathway enrichment analysis after NEFA-treated. (**D**) Expression level of C18:2n6c and C20:4n6. (**E**) Expression level of C18:3n3 and C20:5n3. All experiments were set up with three biological replications, and the statistical results were presented in the form of mean ± SEM. ** *p*-value < 0.01, **** *p*-value < 0.0001, ns, not significant. Same as below.

The experimental results show that NEFA exposure significantly altered cellular fatty acid profiles, with 26 upregulated and 3 downregulated species. Principal component analysis (PCA) demonstrated clear separation between NEFA-treated and control groups along PC1 (83.061% variance) and PC2 (13.65% variance) (Figure 3A). Hierarchical clustering revealed distinct expression patterns of 29 fatty acids, particularly upregulation of arachidonic acid (C20:4n6), linoleic acid (C18:2n6c), α-Linolenic acid (C18:3n3), docosahexaenoic acid (C22:6n3), and eicosapentaenoic acid (C20:5n3) (Figure 3C). KEGG analysis identified three significantly disrupted fatty acid metabolic pathways (*p* < 0.0001, Figure 3B). Biosynthesis of unsaturated fatty acids and linoleic acid metabolism pathway alterations indicate NEFA-mediated dysregulation of lipid biosynthesis.

### 3.4. NEFA Induces Endoplasmic Reticulum Stress in BGCs

**Figure 4:**
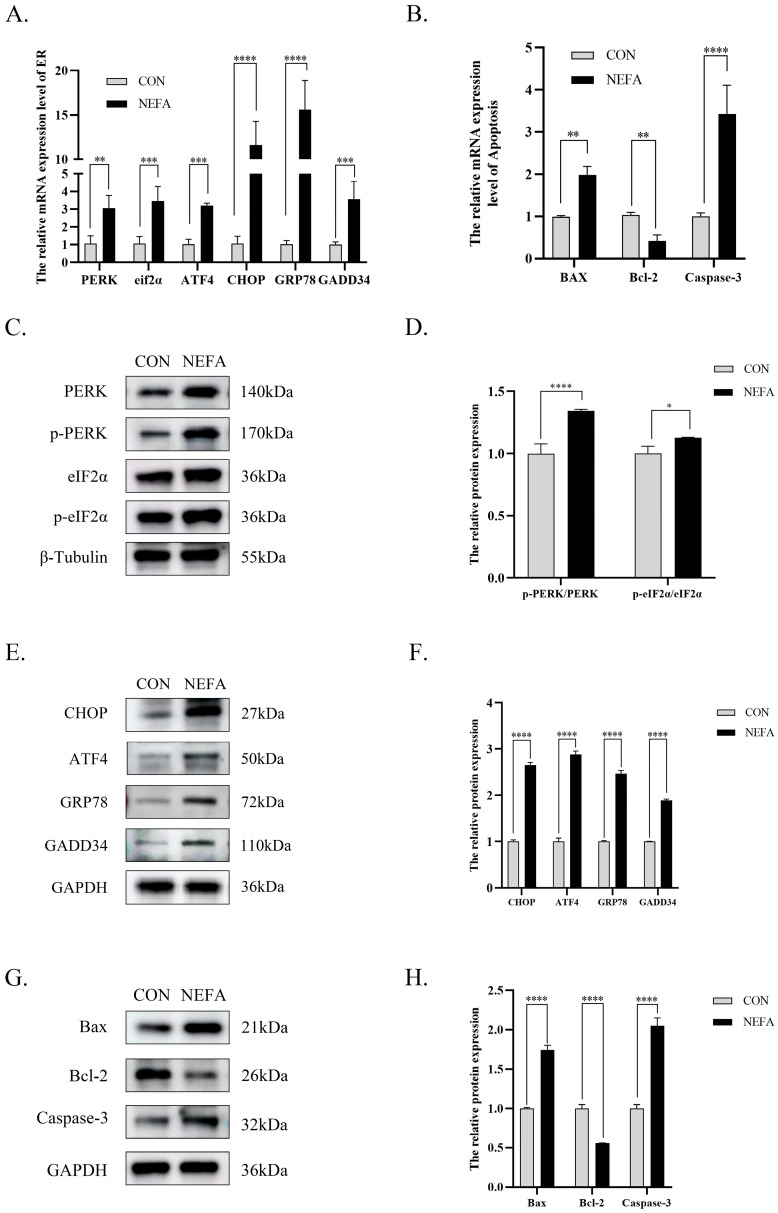
NEFA induces endoplasmic reticulum stress and alters mRNA and protein expression in BGCs via the PERK/eIF2α/ATF4/CHOP signaling pathway. (**A**,**B**) Effect of NEFA on the level of *PERK*, *eIF2α*, *ATF4*, *CHOP*, *GRP78*, *GADD34*, *BAX*, *Bcl-2* and *Caspase-3* mRNA expression. (**C**–**H**) Representative Western blotting bands of PERK, eIF2α, ATF4, CHOP, GRP78, GADD34, BAX, Bcl-2, Caspase-3. Quantification of relative protein levels were analyzed using Image J and the results were shown after the bands (protein/β-Tubulin, GAPDH). All experiments were set up with three biological replications, and the statistical results were presented in the form of mean ± SEM. * *p*-value < 0.05, ** *p*-value < 0.01, *** *p*-value < 0.001, **** *p*-value < 0.0001, ns, not significant. Western Blot original pictures see Figures S1–S4.

We assessed the impact of NEFA treatment (0.9 mM, 24 h) on ER stress pathways by quantifying mRNA and protein expression of key mediators. Compared to controls, the expression level of GRP78 (the core marker protein of ER stress) was significantly increased (*p* < 0.0001). The PERK pathway in ER stress was activated, with significantly increased expression levels of protein kinase R-like endoplasmic reticulum kinase (*PERK*), and eukaryotic translation initiation factor 2 alpha (*eIF2α*) (*p* < 0.001). Expression levels of the downstream active transcription factor 4(*ATF4*) and pro-apoptotic factors C/EBP homologous protein (*CHOP*) and growth arrest and DNA damage-inducible 34(*GADD34*) were significantly increased (*p* < 0.001, Figure 4A). Expression of pro-apoptotic genes Bcl2-Associated X (*BAX*), and caspase-3 was significantly increased(*p* < 0.01), while anti-apoptotic B-cell lymphoma-2 (*Bcl-2*) was significantly decreased (*p* < 0.01, Figure 4B). Western blot results corroborated qRT-PCR data at both transcriptional and translational levels (Figure 4C–H). In NEFA-treated groups, both phosphorylated and non-phosphorylated forms of PERK and eIF2α proteins were significantly increased (*p* < 0.01), with the phosphorylation ratios (p-PERK/total PERK and p-eIF2α/total eIF2α) also significantly increased (*p* < 0.05, Figure 4C,D). The relative expression levels of CHOP, ATF4, GRP78, and GADD34 were extremely increased (*p* < 0.0001, Figure 4E,F). The relative expression levels of BAX, caspase-3 were increased (*p* < 0.0001), while Bcl-2 protein expression was decreased (*p* < 0.0001, Figure 4G,H).

### 3.5. 4-PBA Alleviates NEFA-Induced ER Stress and Apoptosis in BGCs

**Figure 5:**
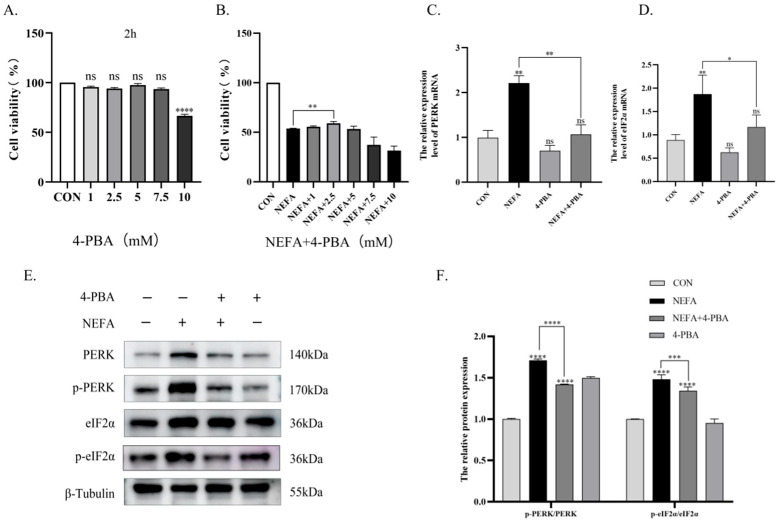
4-PBA alleviates NEFA-induced endoplasmic reticulum stress in BGCs. (**A**) Effect of different concentrations of NEFA treatment for 24 h on the viability of BGCs. (**B**) Viability of BGCs pretreated with various 4-PBA concentrations for 2 h before NEFA exposure. (**C**,**D**) Changes in the mRNA expression levels of *PERK*, and *eIF2α* in cells treated with 4-PBA. (**E**,**F**) Representative Western blot images of PERK, eIF2α, p-PERK, p-eIF2α. Quantification of relative protein levels was performed using Image J, and the results are presented as ratios to β-Tubulin or GAPDH. All experiments were set up with three biological replications, and the statistical results were presented in the form of mean ± SEM. * *p*-value < 0.05, ** *p*-value < 0.01, *** *p*-value < 0.001, **** *p*-value < 0.0001, ns, not significant. Western Blot original pictures see Figures S1–S4.

**Figure 6:**
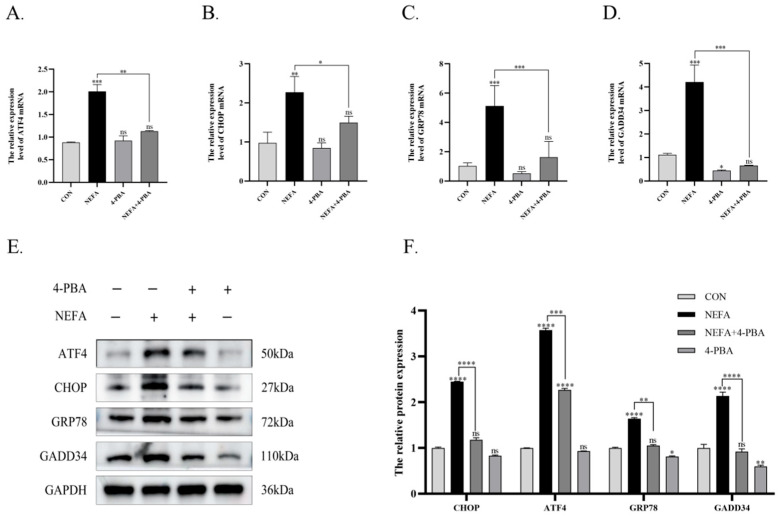
4-PBA alleviated the changes in ATF4 and related genes induced by NEFA in BGCs. (**A**–**D**) Changes in the mRNA expression levels of *ATF4*, *CHOP*, *GRP78*, and *GADD34* in cells treated with 4-PBA. (**E**,**F**) Representative Western blot images of ATF4, CHOP, GRP78, and GADD34. Quantification of relative protein levels was performed using Image J, and the results are presented as ratios to β-Tubulin or GAPDH. All experiments were set up with three biological replications, and the statistical results were presented in the form of mean ± SEM. * *p*-value < 0.05, ** *p*-value < 0.01, *** *p*-value < 0.001, **** *p*-value < 0.0001, ns, not significant. Western Blot original pictures see Figures S1–S4.

**Figure 7:**
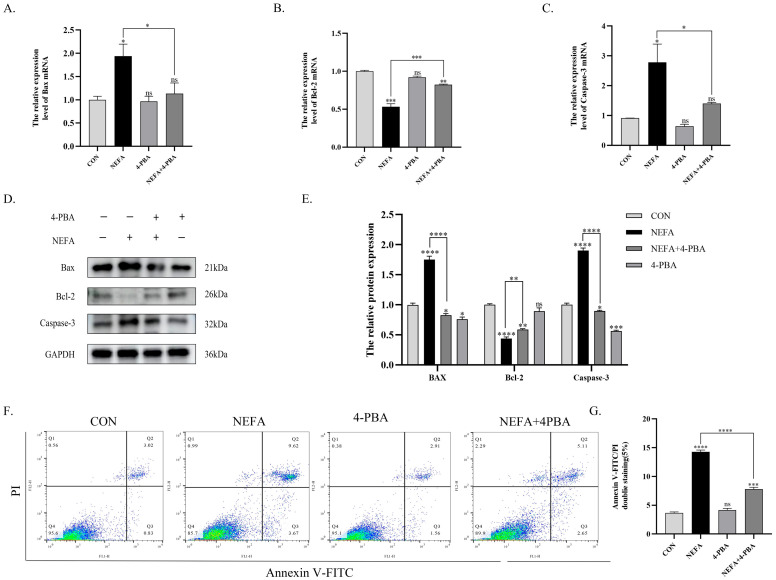
4-PBA reduces NEFA-induced apoptosis in BGCs. (**A**–**C**) Changes in the mRNA expression levels of *BAX*, *Bcl-2*, and *Caspase-3* in cells treated with 4-PBA. (**D**,**E**) Representative Western blot images of BAX, Bcl-2, and cleaved Caspase-3. Quantification of relative protein levels was performed using Image J, with results expressed as ratios to GAPDH. (**F**) Apoptosis in BGCs treated with CON, NEFA, 4-PBA, and NEFA+4-PBA was determined by flow cytometry and analyzed using FlowJo. (**G**) Comparison of apoptosis rates between different treatment groups. All experiments were set up with three biological replications, and the statistical results were presented in the form of mean ± SEM. * *p*-value < 0.05, ** *p*-value < 0.01, *** *p*-value < 0.001, **** *p*-value < 0.0001, ns, not significant. Western Blot original pictures see Figures S1–S4.

As an ER stress inhibitor, 4-PBA preserved cell viability at non-toxic concentrations. Notably, a concentration of 2.5 mM 4-PBA significantly increased granulosa cell viability compared to the NEFA-treated group. (Figure 5A,B). Compared to the NEFA group alone, pharmacological inhibition of endoplasmic reticulum stress by 4-PBA suppressed PERK signaling pathway activation and consequently attenuated apoptosis in BGCs. Pretreatment with 4-PBA decreased NEFA-induced upregulation of *PERK*, *p-PERK*, *eIF2α*, *p-eIF2α*, *ATF4*, *CHOP*, *GRP78*, and *GADD34* (*p* < 0.05, Figure 5C,D and Figure 6A–D), with concordant reduction in corresponding protein expression levels. (*p* < 0.001, Figure 5E,F and Figure 6E,F). Building on these findings, the 4-PBA-preconditioned group showed significant downregulation of BAX and Caspase-3 transcripts and protein expression, along with concomitant upregulation of Bcl-2 transcripts and protein levels compared to the NEFA group (*p* < 0.05, Figure 7A–E). Flow cytometric analysis demonstrated that 4-PBA pretreatment significantly downregulated NEFA-induced apoptosis relative to the NEFA group (*p* < 0.001, Figure 7F,G).

In summary, 4-PBA alleviates NEFA-induced apoptosis in BGCs through PERK signaling pathway inhibition.

## 4. Discussion

Our findings reveal that NEFA activates the PERK pathway, leading to endoplasmic reticulum (ER) stress, apoptosis, intracellular lipid accumulation, and alterations in the hormonal profile of bovine granulosa cells. Together, these effects provide a novel perspective on how NEFA impairs follicular development. Persistent ER stress converts the unfolded protein response (UPR) from an adaptive, cytoprotective program into a terminal, pro-apoptotic signaling cascade. Dissociation of BiP from PERK unleashes PERK activity, resulting in the rapid phosphorylation of eIFα at Ser51. Phosphorylated eIF2α selectively enhances translation of ATF4, which cooperates with CHOP to amplify transcriptional output. Subsequently, CHOP orchestrates the expression of pro-apoptotic genes, thereby inducing the apoptotic program [31,32,33]. Our central finding is the activation of the PERK-eIF2α-ATF4-CHOP axis of the UPR in NEFA-treated BGCs, culminating in apoptosis. This was evidenced by the upregulation of GRP78, increased phosphorylation of PERK and eIF2α, and subsequent elevation of the pro-apoptotic executors BAX and Caspase-3. These findings were consistent with observations reported by Hua, thus demonstrating that NEFA treatment induces ER stress in GCs. Concomitantly, elevated expression of GADD34—a key regulatory factor in ER stress—was observed. Upon ER stress induction, PERK pathway activation promotes eIF2α phosphorylation, which attenuates global protein synthesis to alleviate ER burden. Conversely, GADD34 facilitates eIF2α dephosphorylation, thereby restoring protein translation and promoting cellular recovery from ER stress [34]. Our study further revealed a significant upregulation of pro-apoptotic executors BAX and Caspase-3 in NEFA-treated cells. To delineate the causal relationship between ER stress and apoptosis, we employed the ER stress inhibitor 4-PBA in our experimental paradigm. 4-PBA mitigates ER stress not only by activating the PI3K/AKT axis in PC12 cells and by attenuating TNFα-induced ER stress in human airway smooth muscle (hASM) cells, but also exerts comparable protective effects in bovine endometrial epithelial cells [35,36]. Our findings demonstrate that 4-PBA attenuates the expression of key PERK pathway components and concurrently suppresses pro-apoptotic executors (BAX, Caspase-3). Collectively, these results establish that NEFA induces GC apoptosis via sustained activation of ER stress pathways, with PERK signaling serving as a core causative factor. Given that GCs are essential for oocyte nourishment and follicular integrity, their apoptosis represents a direct mechanism by which elevated NEFA levels could compromise follicle development and contribute to the follicular atresia observed in NEB-affected cows.

In addition to ER stress, NEFA-induced lipotoxicity disrupts both fatty-acid and endogenous hormones within GCs. In this study, Oil Red O staining demonstrated excessive intracellular lipid accumulation in NEFA-treated GCs. Targeted metabolomics revealed elevated levels of multiple eicosanoids, particularly arachidonic acid (AA) and eicosapentaenoic acid (EPA). These polyunsaturated fatty acids (AA, EPA, linoleic acid, and α-linolenic acid) are metabolized by specific enzymes to generate substantial amounts of oxidized lipids, which exert multifaceted and pronounced cytotoxic effects [37], including membrane structure destruction, cell death induction, DNA damage, immunosuppression, and amplification of inflammation [38,39]. Oxidized lipids potently induce ER stress in GCs through multiple mechanisms: They upregulate ERO-1α expression, whose enzymatic cycle generates excessive H_2_O_2_ within the ER lumen, which accumulates and triggers ER stress. These lipids are precursors for oxidized metabolites, which are known cytotoxic agents reported to exacerbate ER stress and inflammation in other systems [40,41]. This NEFA-induced lipotoxicity likely creates a vicious cycle within GCs, where initial metabolic insult from lipid overload is amplified by the generation of damaging oxidized derivatives, further straining cellular homeostasis. NEFA, key metabolites during postpartum NEB in dairy cows, which elevated levels of NEFA compromise reproductive efficiency through multifaceted pathways: direct impairment of ovarian cell function, disruption of hormonal secretion, and induction of oxidative/ER stress. Elevated circulating NEFA levels in NEB-affected dairy cows substantially contribute to ovarian pathologies, compromising reproductive efficiency in a significant proportion of cases [42,43]. As demonstrated by Chang, increased NEFA concentrations in NEB cows severely impair follicular development [44]. Metabolic profiling of postpartum anovulatory cows revealed marked elevations in serum NEFA and β-hydroxybutyrate (BHBA) levels, along with reduced estradiol (E2) and diminished reproductive performance compared to healthy controls [45]. Comparative analysis of postpartum NEB-affected dairy cows demonstrated significantly elevated milk urea nitrogen (MUN) and circulating NEFA levels relative to healthy controls, concomitant with markedly depressed daily milk yield, estrus frequency, and conception rates [46]. GCs, which envelop the oocyte, critically support its normal development through bidirectional signaling. Consequently, elevated NEFA levels not only directly impair GCs function but also likely disrupt ovarian homeostasis [47]. More profoundly, sustained elevation of circulating NEFA levels disrupts follicular microenvironment homeostasis by altering the biochemical composition of follicular fluid. Concomitantly, NEFA-induced ER stress impairs key physiological processes essential for follicular integrity. This concept is supported by studies linking ER stress in the ovary to pathologies like ovarian hyperstimulation syndrome (OHSS) [48], and polycystic ovary syndrome (PCOS)-associated fibrosis [49]. NEB triggers adipose tissue lipolysis, elevating circulating NEFA levels. Since resumption of cyclicity and breeding typically occur 50–60 days postpartum, the cumulative damage to the follicle pool during the preceding weeks of NEB could significantly hamper conception rates. Therefore, mitigating NEFA-induced GCs damage during early lactation could be a key strategy for improving reproductive efficiency and reducing the economic burden of subfertility in dairy operations.

## 5. Conclusions

**Figure 8:**
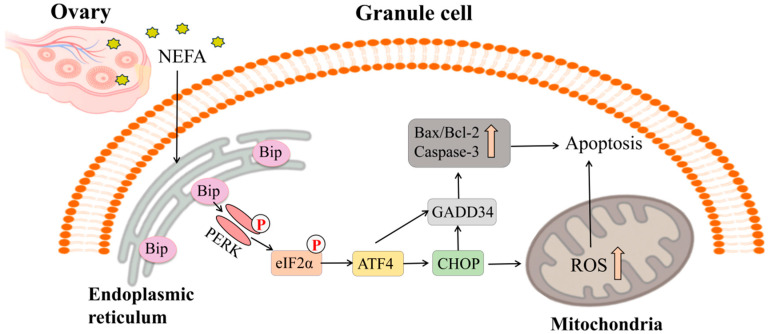
NEFA promotes bovine granulosa cell apoptosis via activation of the PERK/eIF2α/ATF4/CHOP pathway.

Collectively, our findings elucidate a mechanistic pathway: PERK-driven ER stress and apoptosis, through which NEFA directly impairs BGCs viability (Figure 8). We propose that this cellular damage may represent a key contributing factor to the follicular atresia associated with elevated NEFA levels in dairy cows. Our in vitro model has limitations; future studies need to use more complex models to validate the pathophysiological relevance of these pathways during terminal follicular development.

## Figures and Tables

**Table 1:** Primer sequences for qRT-PCR.

Gene	Sequence (5′–3′)	
*PERK*	F: CACAGGGACCTCAAGCCTTC	R:TCCTCGTCTTGGTCCATTGC
*eIF2α*	F: GCATTCTTCGCCATGTTGCTGAG	R: TAGGCACCGTATCCAGGTCTCTTG
*ATF4*	F: TTTTCACGGCATTCAGCAGC	R: ATGTTGCGAGGTTTTGGTGC
*CHOP*	F: AGATGAAAATCGGGGCACCTG	R: CAGTAAGCCAAGCCAGAGAG
*GRP78*	F: CGTGCGTTTGAGAGCTCAGT	R: TTCATCTTTCCAGCGCCGTC
*GADD34*	F: GAAGTTTCCGGCCTGGATGT	R: GGCTCGAGGACTCAAGTTGT
*BAX*	F: CATCATGGGCTGGACATTGG	R: AAGATGGTCACTGTCTGCCA
*Bcl-2*	F: ATGACCGAGTACCTGAACCG	R: GCCATACAGCTCCACAAAGG
*Caspase-3*	F: TTGAGACAGACAGTGGTGCT	R: TCTTTGCATTTCGCCAGGAA
*β-actin*	F: GGGCAGGTCATCACCATCGG	R: TCATTGTGCTGGGTGCCAGG
*HMOX1*	F: CAGGCACCTCTCCTGCGATG	R: AGACAGCAGGAGCCCTGACA
*NQO1*	F: CCGAGGATGCCCGAGTTCAG	R: ACTTGCCCAAGTGATGGCCC
*SOD2*	F: TGAGCCCTAACGGTGGTGGA	R: ATTGAAGCCGAGCCAACCCC

**Table 2:** Antibody information for Western blot analysis.

Antibody	Host Species	Brand	Catalog No.	Dilution
PERK	rabbit	ABclonal, China	A21957	1:500
p-PERK	rabbit	ABclonal, China	AP0886	1:500
eIF2α	rabbit	ABclonal, China	A0764	1:1000
p-eIF2α	rabbit	ABclonal, China	AP0692	1:500
ATF4	rabbit	ABclonal, China	A18687	1:500
CHOP	rabbit	ABclonal, China	A0221	1:1000
GRP78	rabbit	ABclonal, China	A11366	1:500
GADD34	rabbit	ABclonal, China	A16260	1:500
BAX	rabbit	ABclonal, China	A19684	1:1000
Bcl-2	rabbit	ABclonal, China	A19693	1:1000
Caspase-3	rabbit	ABclonal, China	A19664	1:2000
GAPDH	rabbit	ABclonal, China	A19056	1:50,000
β-Tubulin	rabbit	ABclonal, China	A12289	1:5000

## Data Availability

The original contributions presented in this study are included in the article/Supplementary Material. Further inquiries can be directed to the corresponding author.

## Supplementary Materials

The following supporting information can be downloaded at: https://www.mdpi.com/article/10.3390/vetsci12121186/s1, Figures S1–S4: Western Blot original pictures.

## Abbreviations

The following abbreviations are used in this manuscript:

4-PBA	4-phenylbutyric acid
AKT	protein kinase B
ATF4	activating transcription factor 4
ATF6	activating transcription factor 6
BAX	BCL2-Associated X
Bcl-2	B-cell lymphoma-2
BGCs	bovine granulosa cell
BHBA	β-hydroxybutyrate
BIP	immunoglobulin heavy chain binding protein
CD36	cluster of differentiation 36
CHOP	C/EBP Homologous Protein
DCFH-DA	2′,7′-Dichlorofluorescin diacetate
DMEM/F12	Dulbecco’s Modified Eagle Medium/Nutrient Mixture F-12
DMI	dry matter intake
E2	estradiol
eIF2α	eukaryotic translation initiation factor 2α
ER	endoplasmic reticulum
ERO-1α	endoplasmic reticulum oxidoreductin-1 alpha
FBS	fetal bovine serum
FoxO1	forkhead box protein O1
GADD34	growth arrest and DNA damage-inducible protein 34
GCs	granulosa cells
GRP78	glucose regulated protein 78kD
IRE1α	inositol-requiring enzyme 1α
KEGG	kyoto encyclopedia of genes and genomes
MUN	milk urea nitrogen
NEB	negative energy balance
NEFA	non-esterified fatty acids
OD	optical density
OHSS	ovarian hyperstimulation syndrome
P4	progesterone
PBS	phosphate-Buffered Saline
PCOS	polycystic ovary syndrome
PERK	protein kinase RNA–like endoplasmic reticulum kinase
PI	propidium Iodide
PI3K	phosphoinositide 3-Kinase
qRT-PCR	quantitative reverse transcription polymerase chain reaction
ROS	reactive oxygen species
SDS	sodium dodecyl sulfate
SR-BI	scavenger receptor class B type I
TUDCA	tauroursodeoxycholic acid
UPR	unfolded protein response
VEGFA	Vascular endothelial growth factor A
FSHR	follicle-stimulating hormone receptor

## References

[1] Ospina P., Nydam D., Stokol T., Overton T. (2010). Associations of elevated nonesterified fatty acids and beta-hydroxybutyrate concentrations with early lactation reproductive performance and milk production in transition dairy cattle in the northeastern United States. J. Dairy Sci..

[2] Perry R.C., Corah L.R., Cochran R.C., Beal W.E., Stevenson J.S., Minton J.E., Simms D.D., Brethour J.R. (1991). Influence of dietary energy on follicular development, serum gonadotropins, and first postpartum ovulation in suckled beef cows. J. Anim. Sci..

[3] Neto F.R.d.A., dos Santos J.C.G., Arce C.D.d.S., Borquis R.R.A., dos Santos D.J.A., Guimarães K.C., Nascimento A.V.D., de Oliveira H.N., Tonhati H. (2022). Genomic study of the resilience of buffalo cows to a negative energy balance. J. Appl. Genet..

[4] Kia S., Mohri M., Seifi H.A. (2023). Association of precalving serum NEFA concentrations with postpartum diseases and reproductive performance in multiparous Holstein cows: Cut-off values. Veter Med. Sci..

[5] Butler W.R. (2003). Energy balance relationships with follicular development, ovulation and fertility in postpartum dairy cows. Livest. Prod. Sci..

[6] Ribeiro E., Lima F., Greco L., Bisinotto R., Monteiro A., Favoreto M., Ayres H., Marsola R., Martinez N., Thatcher W. (2013). Prevalence of periparturient diseases and effects on fertility of seasonally calving grazing dairy cows supplemented with concentrates. J. Dairy Sci..

[7] Wathes D.C., Fenwick M., Cheng Z., Bourne N., Llewellyn S., Morris D.G., Kenny D., Murphy J., Fitzpatrick R. (2007). Influence of negative energy balance on cyclicity and fertility in the high producing dairy cow. Theriogenology.

[8] Roche J.R., Burke C.R., Crookenden M.A., Heiser A., Loor J.L., Meier S., Mitchell M.D., Phyn C.V.C., Turner S.A. (2018). Fertility and the transition dairy cow. Reprod. Fertil. Dev..

[9] Friggens N., Disenhaus C., Petit H. (2010). Nutritional sub-fertility in the dairy cow: Towards improved reproductive management through a better biological understanding. Animal.

[10] Alam M.H., Miyano T. (2020). Interaction between growing oocytes and granulosa cells in vitro. Reprod. Med. Biol..

[11] Shen L., Liu J., Luo A., Wang S. (2023). The stromal microenvironment and ovarian aging: Mechanisms and therapeutic opportunities. J. Ovarian Res..

[12] Shi M., Sirard M.-A. (2022). Metabolism of fatty acids in follicular cells, oocytes, and blastocysts. Reprod. Fertil..

[13] Furukawa E., Chen Z., Kubo T., Wu Y., Ueda K., Chelenga M., Chiba H., Yanagawa Y., Katagiri S., Nagano M. (2022). Simultaneous free fatty acid elevations and accelerated desaturation in plasma and oocytes in early postpartum dairy cows under intensive feeding management. Theriogenology.

[14] Matsuda F., Inoue N., Manabe N., Ohkura S. (2012). Follicular growth and atresia in mammalian ovaries: Regulation by survival and death of granulosa cells. J. Reprod. Dev..

[15] Asselin E., Xiao C.W., Wang Y.F., Tsang B.K. (2000). Mammalian follicular development and atresia: Role of apoptosis. Biol Signals Recept.

[16] Aardema H., van Tol H.T., Vos P.L. (2019). An overview on how cumulus cells interact with the oocyte in a condition with elevated NEFA levels in dairy cows. Anim. Reprod. Sci..

[17] Sparks B.B., Ford H., Michelotti T.C., Strieder-Barboza C. (2024). Adipose tissue oxylipin profile changes with subclinical ketosis and depot in postpartum dairy cows. J. Dairy Sci..

[18] Montecillo-Aguado M., Tirado-Rodriguez B., Huerta-Yepez S. (2023). The Involvement of Polyunsaturated Fatty Acids in Apoptosis Mechanisms and Their Implications in Cancer. Int. J. Mol. Sci..

[19] Christie W.W., Harwood J.L. (2020). Oxidation of polyunsaturated fatty acids to produce lipid mediators. Essays Biochem..

[20] Vanholder T., Leroy J.L., Van Soom A., Maes D., Coryn M., Fiers T., de Kruif A., Opsomer G. (2005). Effect of non-esterified fatty acids on bovine theca cell steroidogenesis and proliferation in vitro. Anim. Reprod. Sci..

[21] Lei Z., Ali I., Yang M., Yang C., Li Y., Li L. (2023). Non-Esterified Fatty Acid-Induced Apoptosis in Bovine Granulosa Cells via ROS-Activated PI3K/AKT/FoxO1 Pathway. Antioxidants.

[22] Wang Y., Li C., Li J., Wang G., Li L. (2020). Non-Esterified Fatty Acid-Induced Reactive Oxygen Species Mediated Granulosa Cells Apoptosis Is Regulated by Nrf2/p53 Signaling Pathway. Antioxidants.

[23] Wu L.L.-Y., Dunning K.R., Yang X., Russell D.L., Lane M., Norman R.J., Robker R.L. (2010). High-fat diet causes lipotoxicity responses in cumulus-oocyte complexes and decreased fertilization rates. Endocrinology.

[24] Shibahara H., Ishiguro A., Inoue Y., Koumei S., Kuwayama T., Iwata H. (2019). Mechanism of palmitic acid-induced deterioration of in vitro development of porcine oocytes and granulosa cells. Theriogenology.

[25] Celik C., Lee S.Y.T., Yap W.S., Thibault G. (2023). Endoplasmic reticulum stress and lipids in health and diseases. Prog. Lipid Res..

[26] Hua D., Zhou Y., Lu Y., Zhao C., Qiu W., Chen J., Ju R. (2020). Lipotoxicity Impairs Granulosa Cell Function Through Activated Endoplasmic Reticulum Stress Pathway. Reprod. Sci..

[27] Huang Y., Zhao C., Kong Y., Tan P., Liu S., Liu Y., Zeng F., Yuan Y., Zhao B., Wang J. (2021). Elucidation of the mechanism of NEFA-induced PERK-eIF2α signaling pathway regulation of lipid metabolism in bovine hepatocytes. J. Steroid Biochem. Mol. Biol..

[28] Cao Y. (2023). PA-mediated Regulation Mechanism of ROS-Endoplasmic Reticulum Stress on Apoptosis of UCE Cells in Dairy Cows. Master’s Thesis.

[29] Yang F., Li L., Zhao R., Wang G. (2020). Effects of melatonin on endoplasmic reticulum stress and autophagy in bovine ovarian granulosa cells induced by HT-2 toxin. J. Nanjing Agric. Univ..

[30] Kong F., Lei L., Cai L., Li J., Zhao C., Liu M., Qi D., Gao J., Li E., Gao W. (2025). Hypoxia-inducible factor 2α mediates nonesterified fatty acids and hypoxia-induced lipid accumulation in bovine hepatocytes. J. Dairy Sci..

[31] Zeeshan H.M.A., Lee G.H., Kim H.R., Chae H.J. (2016). Endoplasmic Reticulum Stress and Associated ROS. Int. J. Mol. Sci..

[32] Han J., Back S.H., Hur J., Lin Y.-H., Gildersleeve R., Shan J., Yuan C.L., Krokowski D., Wang S., Hatzoglou M. (2013). ER-stress-induced transcriptional regulation increases protein synthesis leading to cell death. Nat. Cell Biol..

[33] He M., Xu Z., Li J. (2024). Research Progress of Endoplasmic Reticulum Stress PERK-eIF2α-AFT4-CHOP Signaling Pathway in Hematological Malignancies. Cancer Res. Prev. Treat..

[34] Hicks D., Giresh K., Wrischnik L.A., Weiser D.C. (2023). The PPP1R15 Family of eIF2-alpha Phosphatase Targeting Subunits (GADD34 and CReP). Int. J. Mol. Sci..

[35] Wang Z., Zheng S., Gu Y., Zhou L., Lin B., Liu W. (2020). 4-PBA Enhances Autophagy by Inhibiting Endoplasmic Reticulum Stress in Recombinant Human Beta Nerve Growth Factor-Induced PC12 cells After Mechanical Injury via PI3K/AKT/mTOR Signaling Pathway. World Neurosurg..

[36] Delmotte P., Yap J.Q., Dasgupta D., Sieck G.C. (2023). Chemical Chaperone 4-PBA Mitigates Tumor Necrosis Factor Alpha-Induced Endoplasmic Reticulum Stress in Human Airway Smooth Muscle. Int. J. Mol. Sci..

[37] Liu T., Ai D. (2025). Roles of Lipoxygenases in Cardiovascular Diseases. J. Cardiovasc. Transl. Res..

[38] Ma X.-H., Liu J.-H., Liu C.-Y., Sun W.-Y., Duan W.-J., Wang G., Kurihara H., He R.-R., Li Y.-F., Chen Y. (2022). ALOX15-launched PUFA-phospholipids peroxidation increases the susceptibility of ferroptosis in ischemia-induced myocardial damage. Signal Transduct. Target. Ther..

[39] Qin Y., Huo F., Feng Z., Hou J., Ding Y., Wang Q., Gui Y., Yang Z., Yang J., Zhou G. (2025). CD36 promotes iron accumulation and dysfunction in CD8+ T cells via the p38-CEBPB-TfR1 axis in earlystage hepatocellular carcinoma. Clin. Mol. Hepatol..

[40] Sharifi S., Yamamoto T., Zeug A., Elsner M., Avezov E., Mehmeti I. (2024). Non-esterified fatty acid palmitate facilitates oxidative endoplasmic reticulum stress and apoptosis of β-cells by upregulating ERO-1α expression. Redox Biol..

[41] Haberzettl P., Hill B.G. (2013). Oxidized lipids activate autophagy in a JNK-dependent manner by stimulating the endoplasmic reticulum stress response. Redox Biol..

[42] Leroy J.L.M.R., Vanholder T., Mateusen B., Christophe A., Opsomer G., de Kruif A., Genicot G., Van Soom A. (2005). Non-esterified fatty acids in follicular fluid of dairy cows and their effect on developmental capacity of bovine oocytes in vitro. Reproduction.

[43] Leroy J., Opsomer G., Van Soom A., Goovaerts I., Bols P. (2008). Reduced fertility in high-yielding dairy cows: Are the oocyte and embryo in danger? Part I. The importance of negative energy balance and altered corpus luteum function to the reduction of oocyte and embryo quality in high-yielding dairy cows. Reprod. Domest. Anim..

[44] Zhao C., Xu R., Xin S., Jiang B., Feng S., Wang X., Xia C. (2025). AMPKα alleviates the inhibitory effect of NEFA on the function of bovine follicular granulosa cells cultured in vitro. Anim. Reprod. Sci..

[45] Sammad A., Khan M.Z., Abbas Z., Hu L., Ullah Q., Wang Y., Zhu H., Wang Y. (2022). Major Nutritional Metabolic Alterations Influencing the Reproductive System of Postpartum Dairy Cows. Metabolites.

[46] Beam S., Butler W. (1999). Effects of energy balance on follicular development and first ovulation in postpartum dairy cows. J. Reprod. Fertil. Suppl..

[47] Dumesic D.A., Meldrum D.R., Katz-Jaffe M.G., Krisher R.L., Schoolcraft W.B. (2014). Oocyte environment: Follicular fluid and cumulus cells are critical for oocyte health. Fertil. Steril..

[48] Takahashi N., Harada M., Hirota Y., Zhao L., Yoshino O., Urata Y., Izumi G., Takamura M., Hirata T., Koga K. (2016). A potential role of endoplasmic reticulum stress in development of ovarian hyperstimulation syndrome. Mol. Cell. Endocrinol..

[49] Takahashi N., Harada M., Hirota Y., Nose E., Azhary J.M., Koike H., Kunitomi C., Yoshino O., Izumi G., Hirata T. (2017). Activation of Endoplasmic Reticulum Stress in Granulosa Cells from Patients with Polycystic Ovary Syndrome Contributes to Ovarian Fibrosis. Sci. Rep..

