# Supplementary figures and images for: NEFA Promotes Bovine Granulosa Cell Apoptosis via Activation of the PERK/eIF2α/ATF4/CHOP Pathway (part 2 of 2)
Source: Vet Sci. 2025 Dec 11;12(12):1186. doi: 10.3390/vetsci12121186 (PMC12737492; doi:10.3390/vetsci12121186)

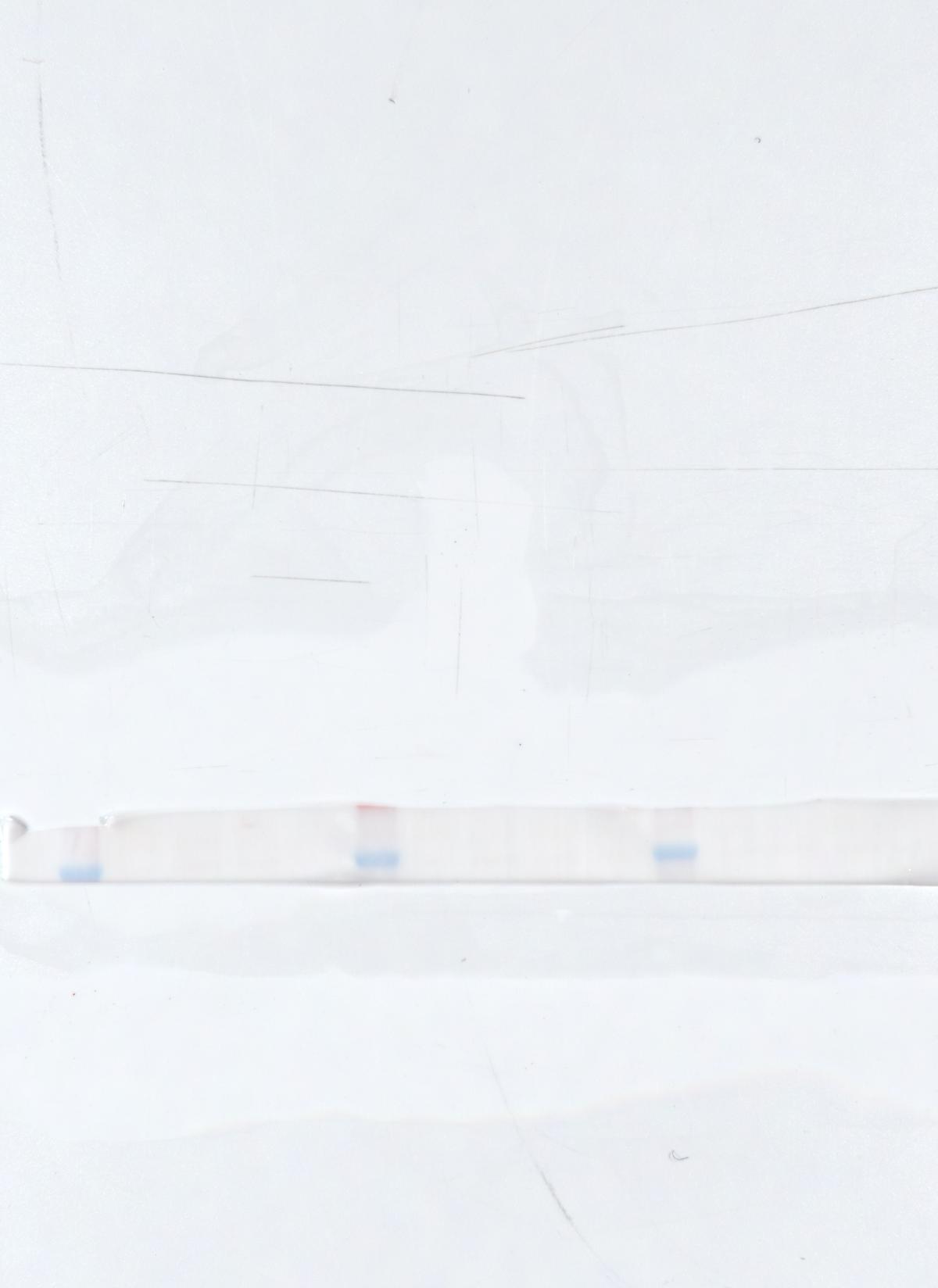

Supplement: Supplementary file 1 [file vetsci-12-01186-s001.zip › Supplementary Files/WB uncropped figure/Figure S3/GRP78 22 20250426_125839_Ch/GRP78 22 20250426_125839_Ch-Marker.jpg]

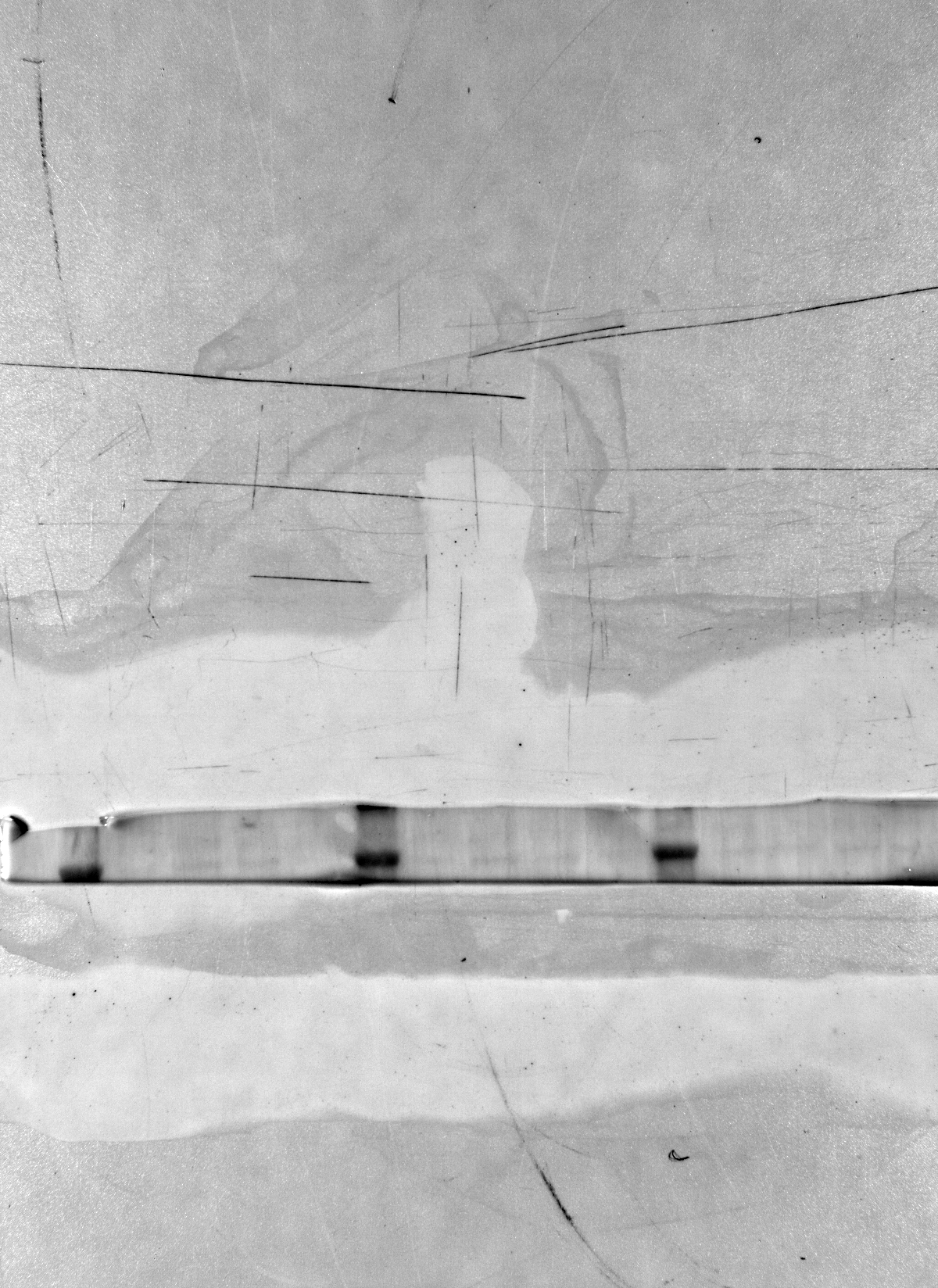

Supplement: Supplementary file 1 [file vetsci-12-01186-s001.zip › Supplementary Files/WB uncropped figure/Figure S3/GRP78 22 20250426_125839_Ch/GRP78 22 20250426_125839_Ch-Marker.tif]

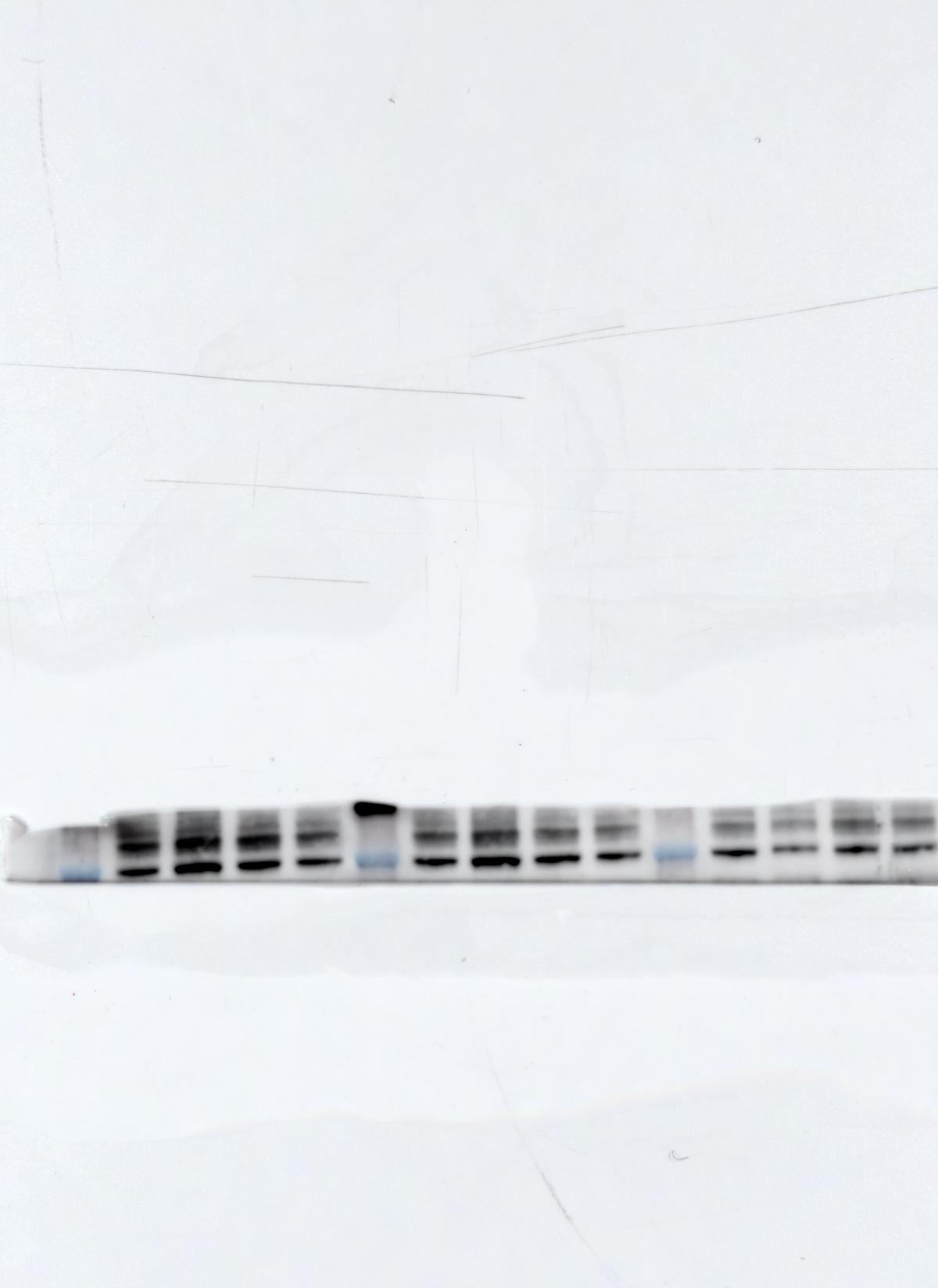

Supplement: Supplementary file 1 [file vetsci-12-01186-s001.zip › Supplementary Files/WB uncropped figure/Figure S3/GRP78 22 20250426_125839_Ch/GRP78 22 20250426_125839_Ch_Chemi+Marker.jpg]

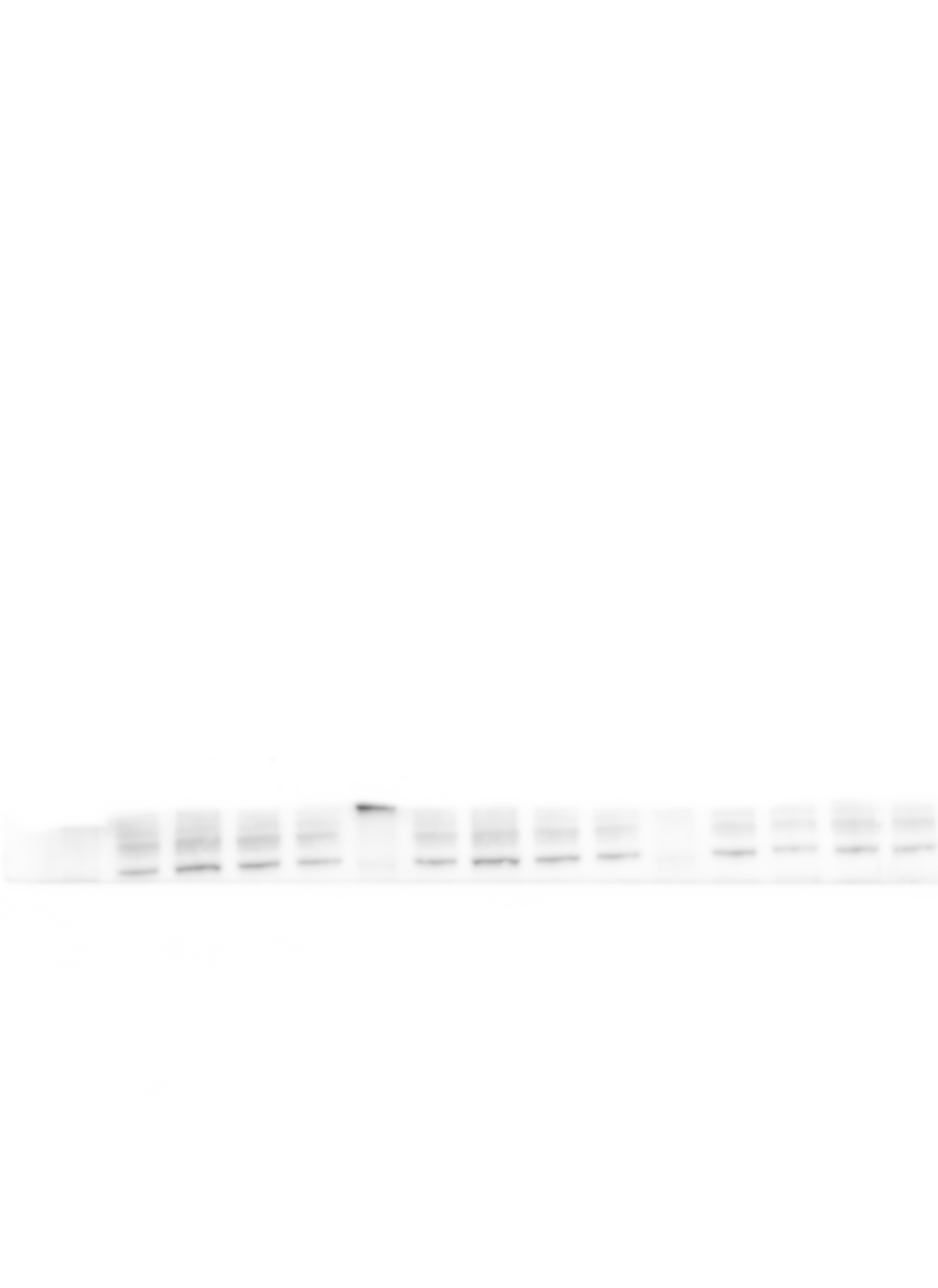

Supplement: Supplementary file 1 [file vetsci-12-01186-s001.zip › Supplementary Files/WB uncropped figure/Figure S3/GRP78 22 20250426_125839_Ch/GRP78 22 20250426_125839_Ch_Chemi.tif]

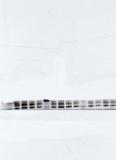

Supplement: Supplementary file 1 [file vetsci-12-01186-s001.zip › Supplementary Files/WB uncropped figure/Figure S3/GRP78 22 20250426_125839_Ch/GRP78 22 20250426_125839_Ch_Thumb.jpg]

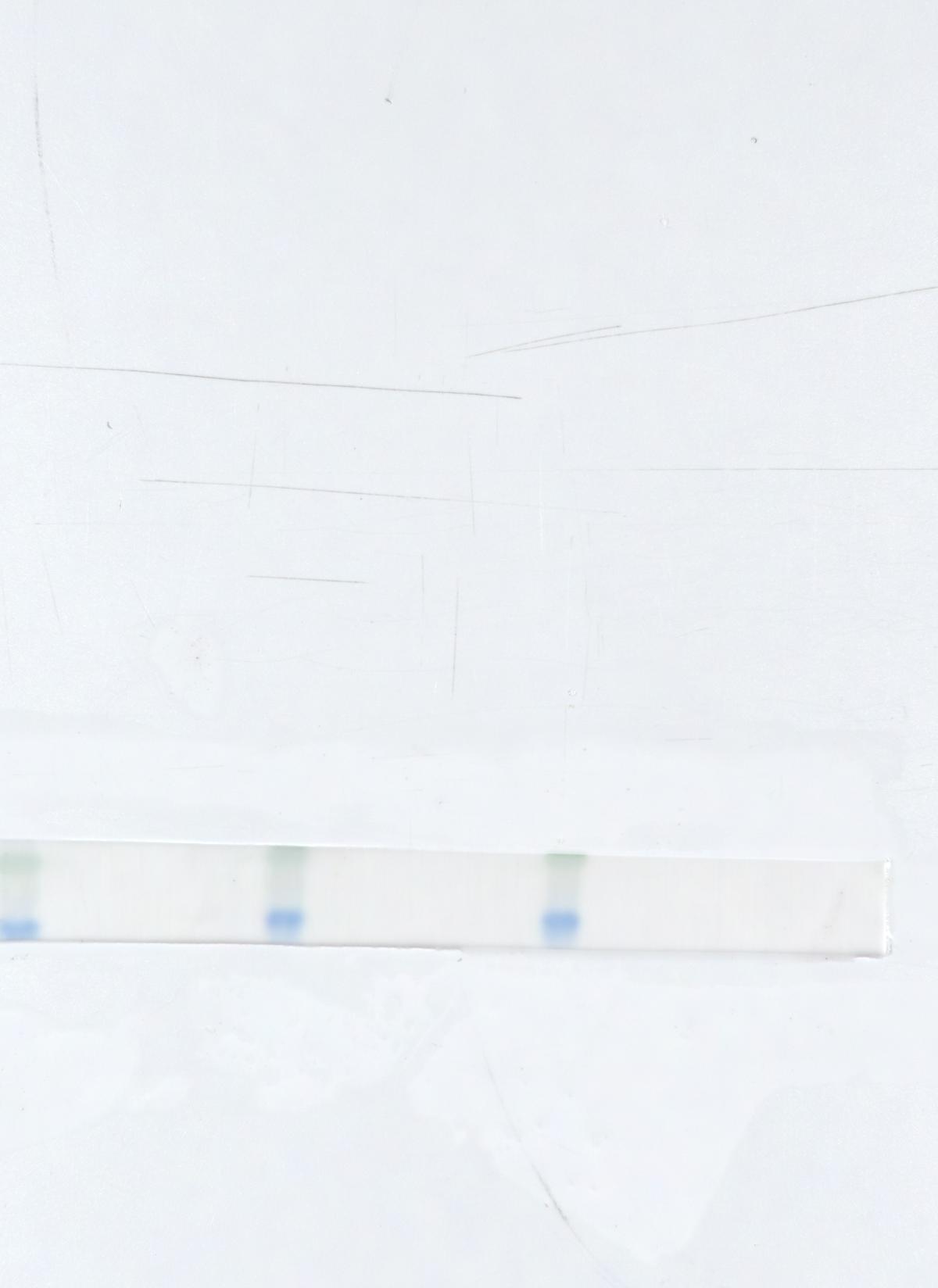

Supplement: Supplementary file 1 [file vetsci-12-01186-s001.zip › Supplementary Files/WB uncropped figure/Figure S4/BAX 20250421_163815_Ch/BAX 20250421_163815_Ch-Marker.jpg]

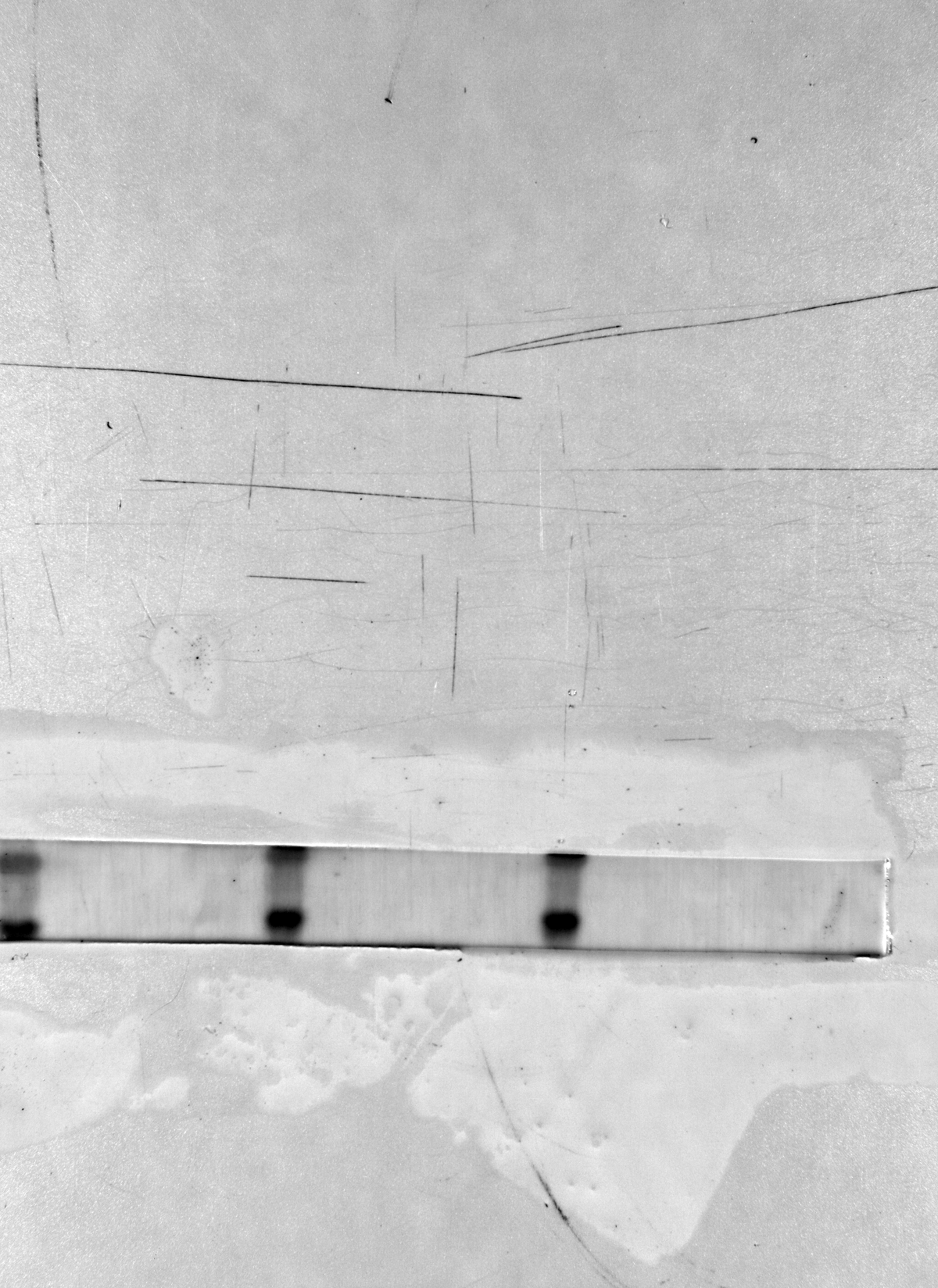

Supplement: Supplementary file 1 [file vetsci-12-01186-s001.zip › Supplementary Files/WB uncropped figure/Figure S4/BAX 20250421_163815_Ch/BAX 20250421_163815_Ch-Marker.tif]

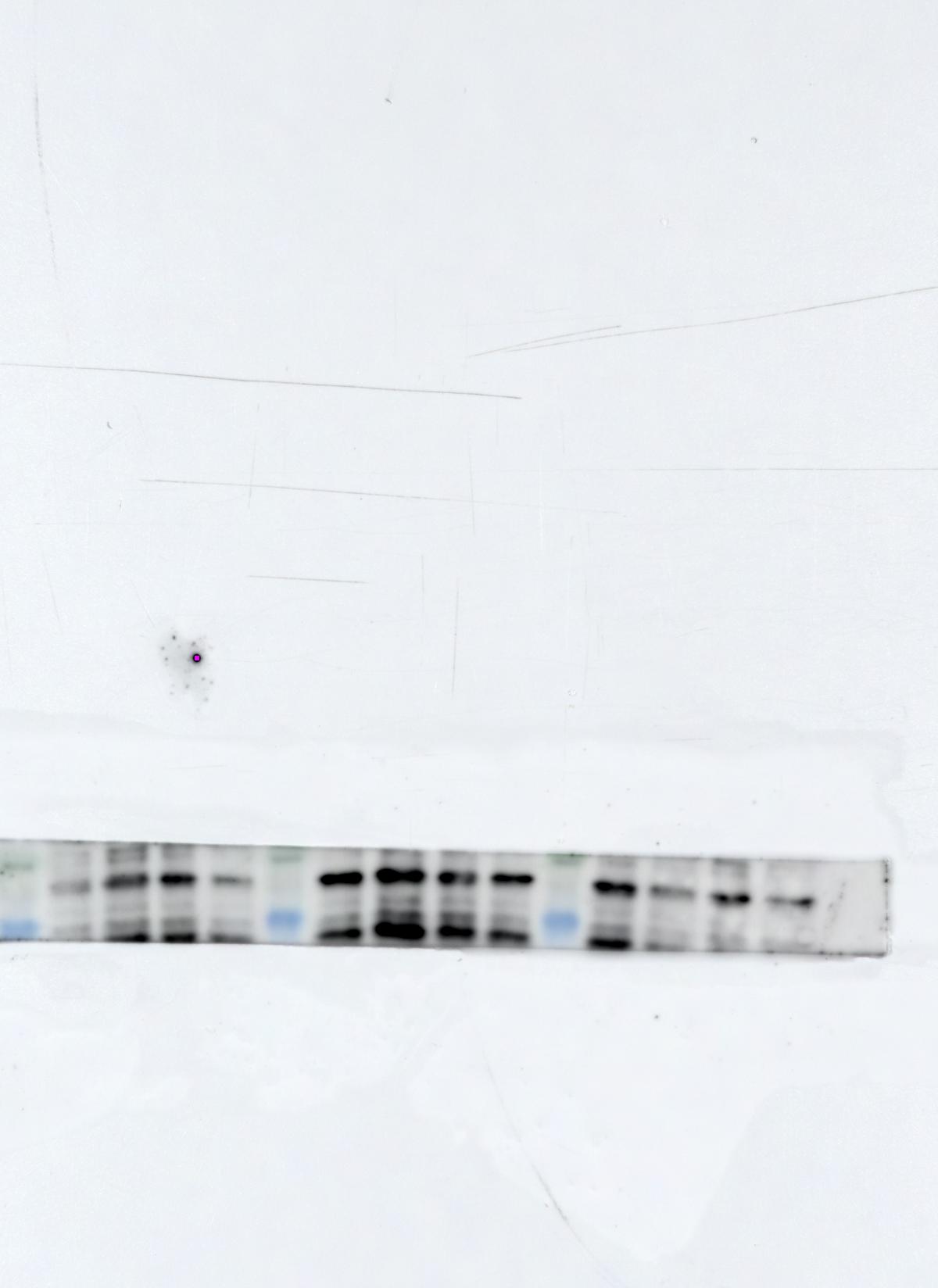

Supplement: Supplementary file 1 [file vetsci-12-01186-s001.zip › Supplementary Files/WB uncropped figure/Figure S4/BAX 20250421_163815_Ch/BAX 20250421_163815_Ch_Chemi+Marker.jpg]

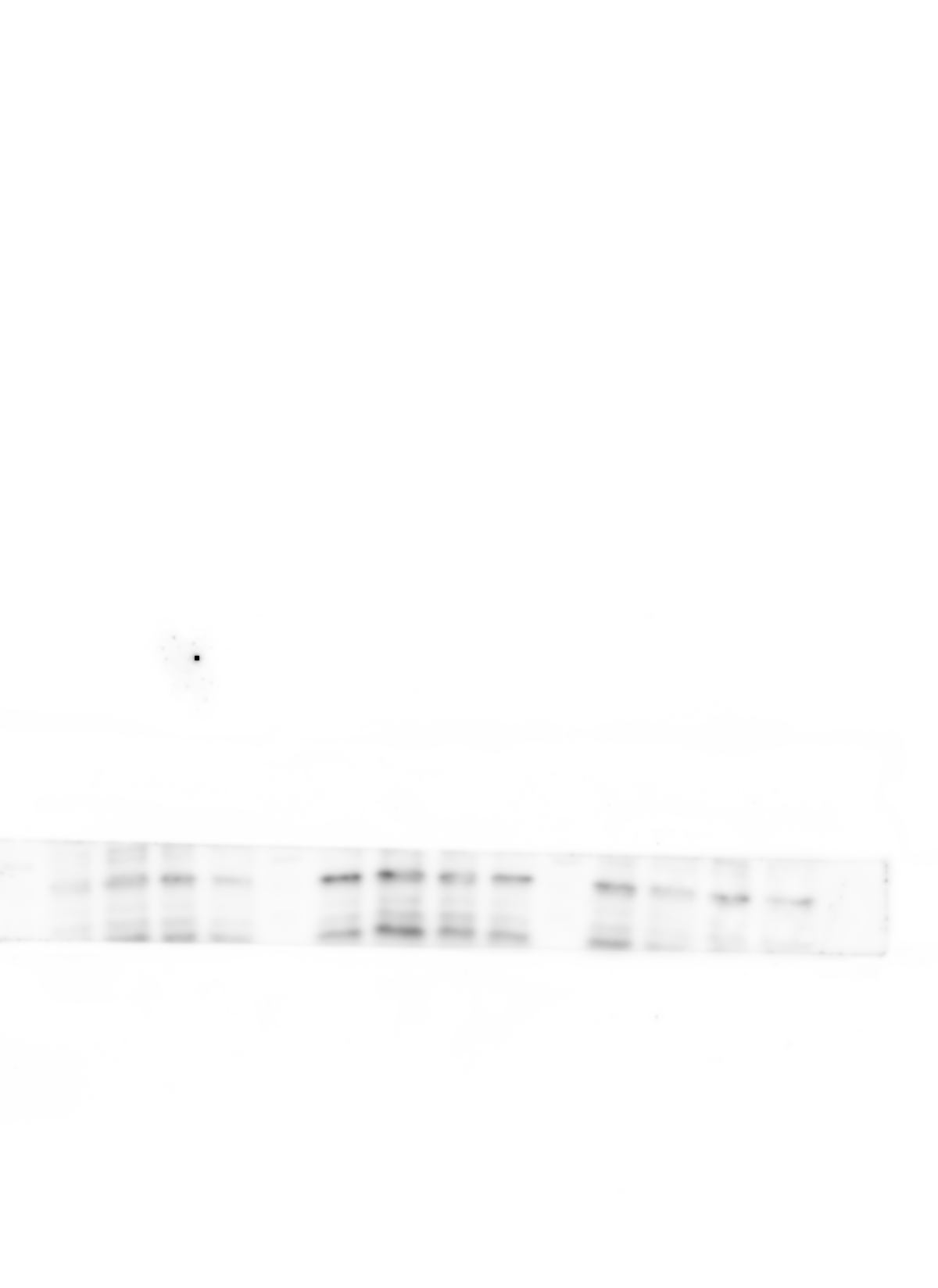

Supplement: Supplementary file 1 [file vetsci-12-01186-s001.zip › Supplementary Files/WB uncropped figure/Figure S4/BAX 20250421_163815_Ch/BAX 20250421_163815_Ch_Chemi.tif]

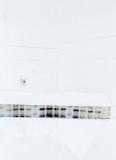

Supplement: Supplementary file 1 [file vetsci-12-01186-s001.zip › Supplementary Files/WB uncropped figure/Figure S4/BAX 20250421_163815_Ch/BAX 20250421_163815_Ch_Thumb.jpg]

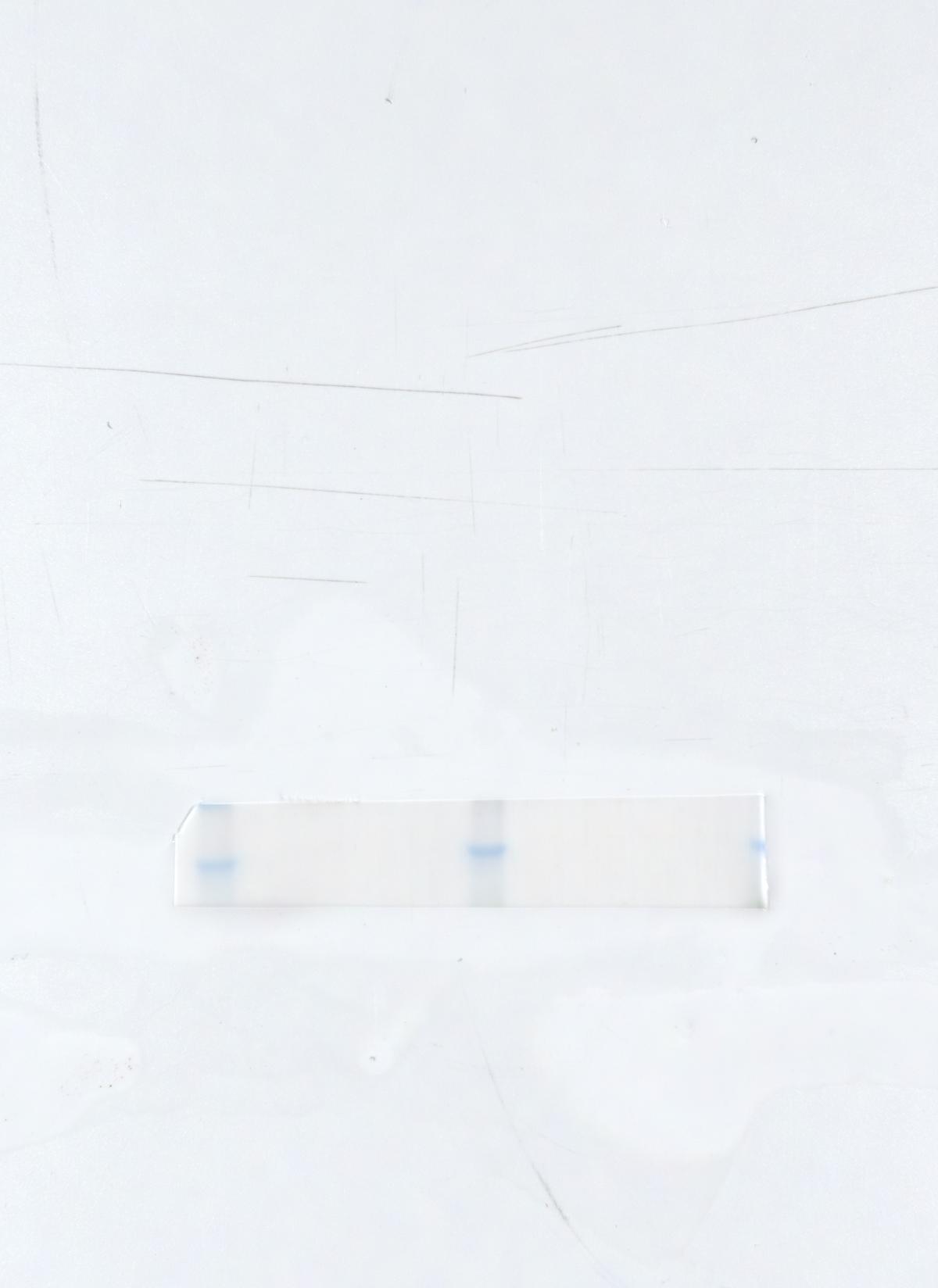

Supplement: Supplementary file 1 [file vetsci-12-01186-s001.zip › Supplementary Files/WB uncropped figure/Figure S4/C-3 2 20250421_164201_Ch/C-3 2 20250421_164201_Ch-Marker.jpg]

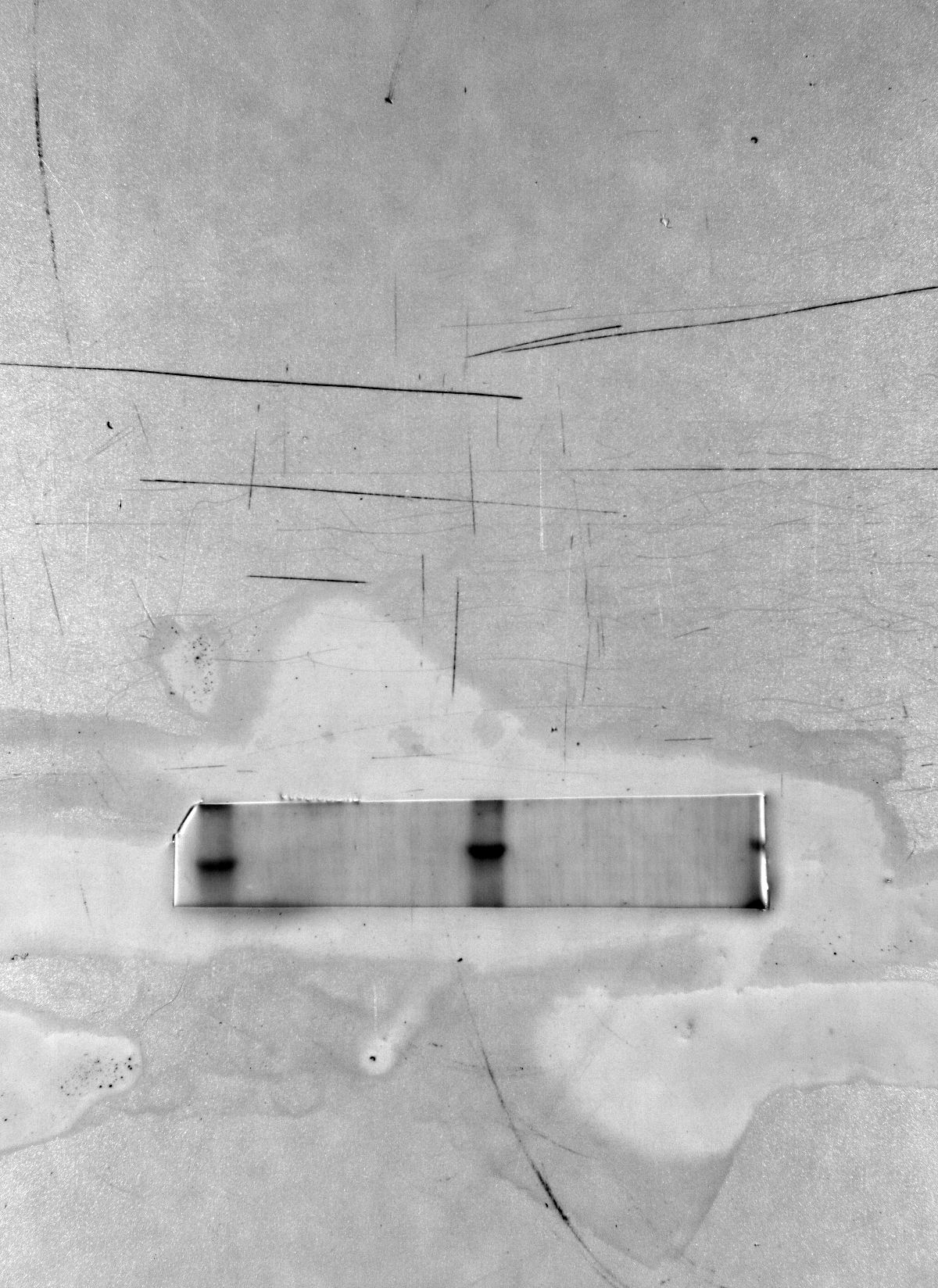

Supplement: Supplementary file 1 [file vetsci-12-01186-s001.zip › Supplementary Files/WB uncropped figure/Figure S4/C-3 2 20250421_164201_Ch/C-3 2 20250421_164201_Ch-Marker.tif]

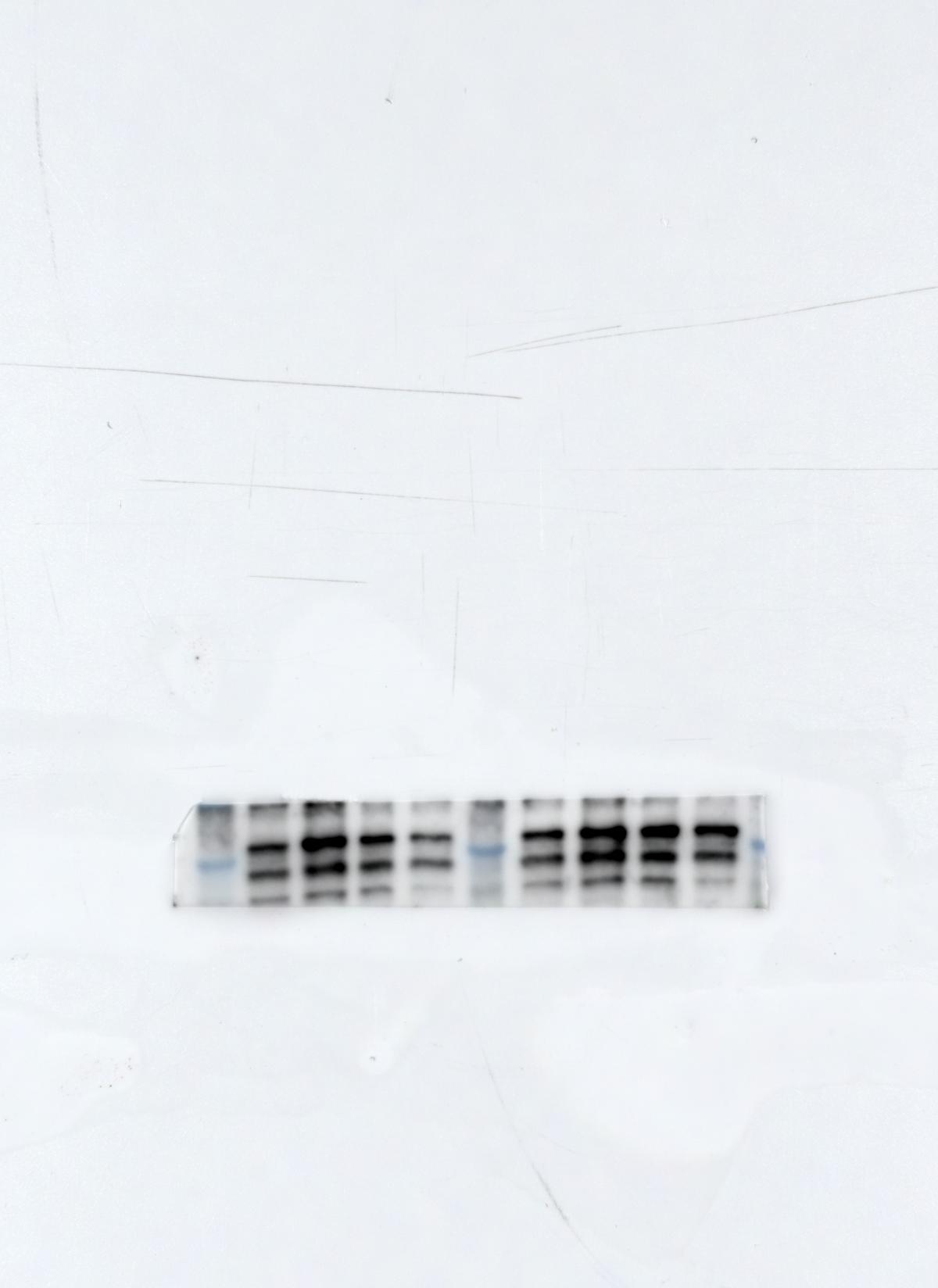

Supplement: Supplementary file 1 [file vetsci-12-01186-s001.zip › Supplementary Files/WB uncropped figure/Figure S4/C-3 2 20250421_164201_Ch/C-3 2 20250421_164201_Ch_Chemi+Marker.jpg]

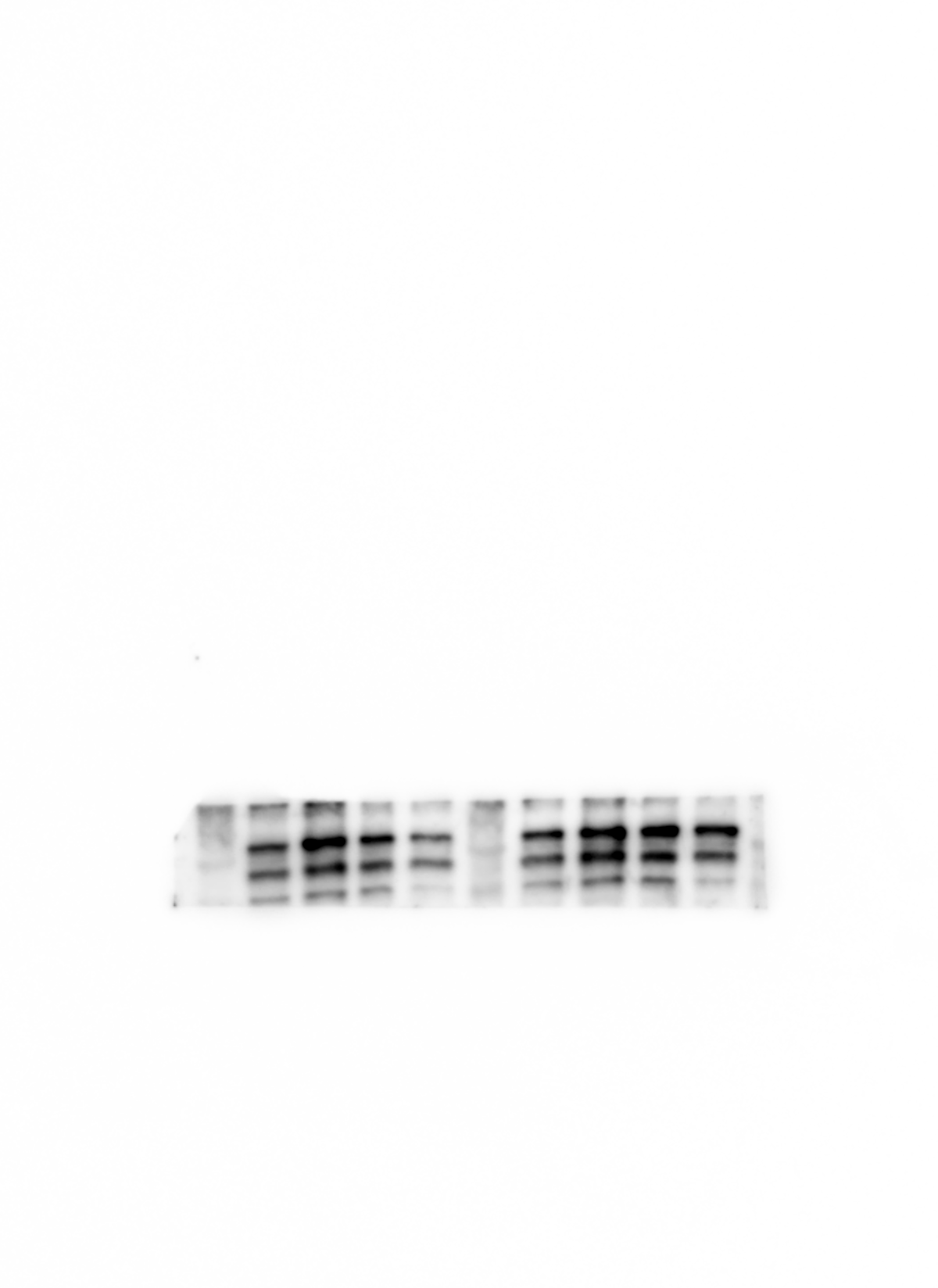

Supplement: Supplementary file 1 [file vetsci-12-01186-s001.zip › Supplementary Files/WB uncropped figure/Figure S4/C-3 2 20250421_164201_Ch/C-3 2 20250421_164201_Ch_Chemi.tif]

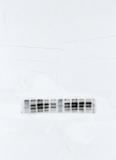

Supplement: Supplementary file 1 [file vetsci-12-01186-s001.zip › Supplementary Files/WB uncropped figure/Figure S4/C-3 2 20250421_164201_Ch/C-3 2 20250421_164201_Ch_Thumb.jpg]

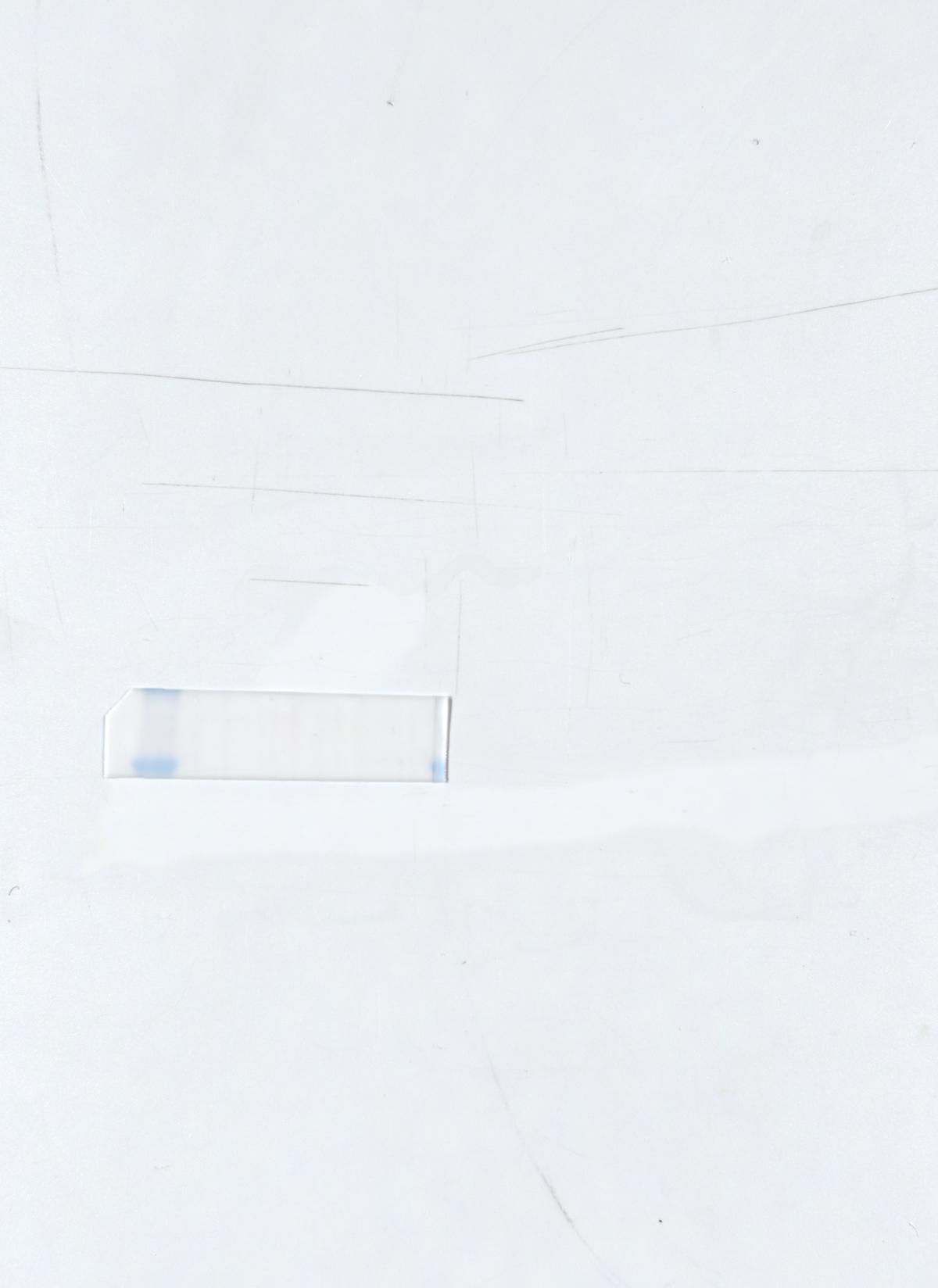

Supplement: Supplementary file 1 [file vetsci-12-01186-s001.zip › Supplementary Files/WB uncropped figure/Figure S4/GAPDH 2 20250429_140856_Ch/GAPDH 1 20250429_140856_Ch-Marker.jpg]

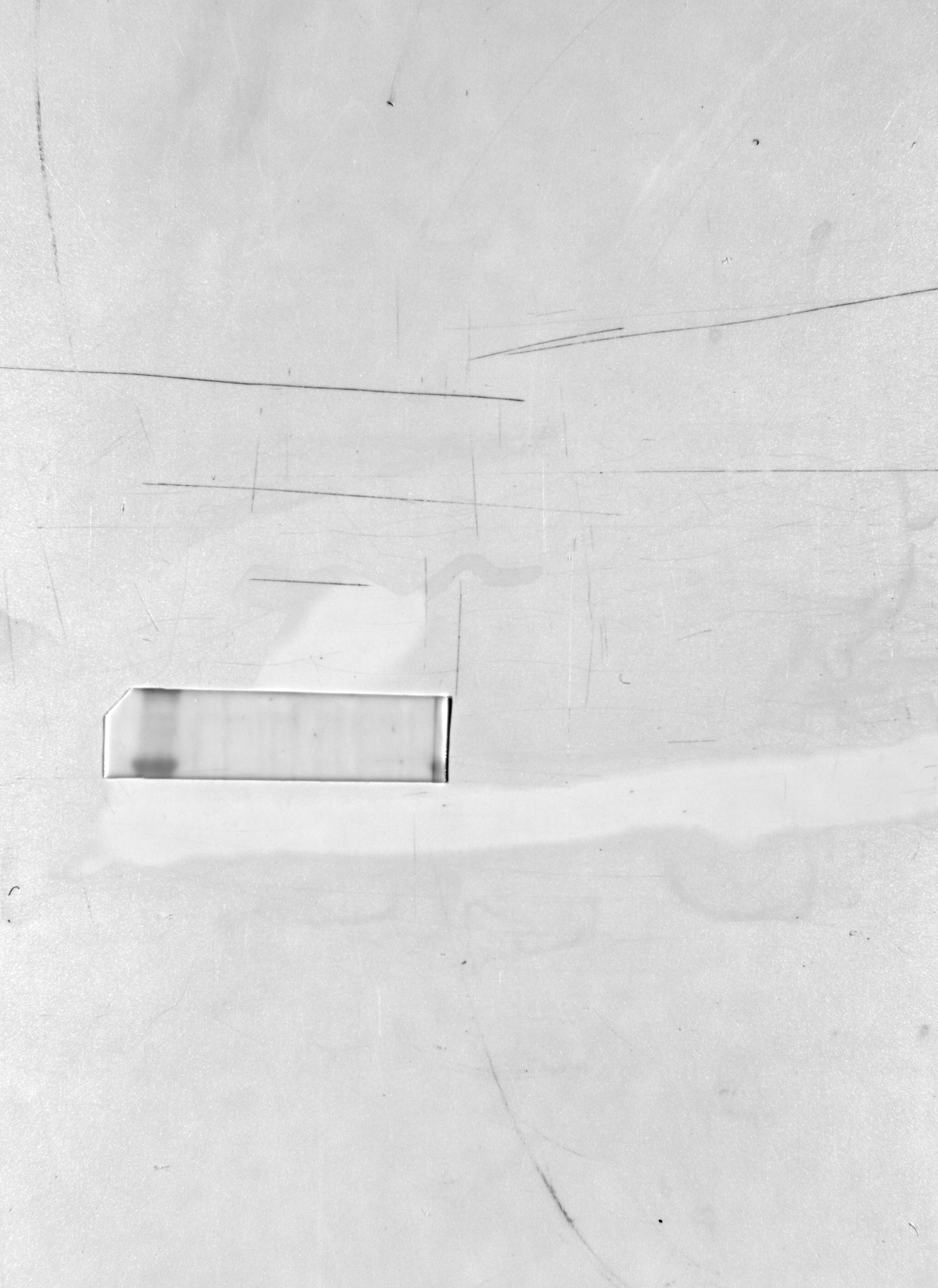

Supplement: Supplementary file 1 [file vetsci-12-01186-s001.zip › Supplementary Files/WB uncropped figure/Figure S4/GAPDH 2 20250429_140856_Ch/GAPDH 1 20250429_140856_Ch-Marker.tif]

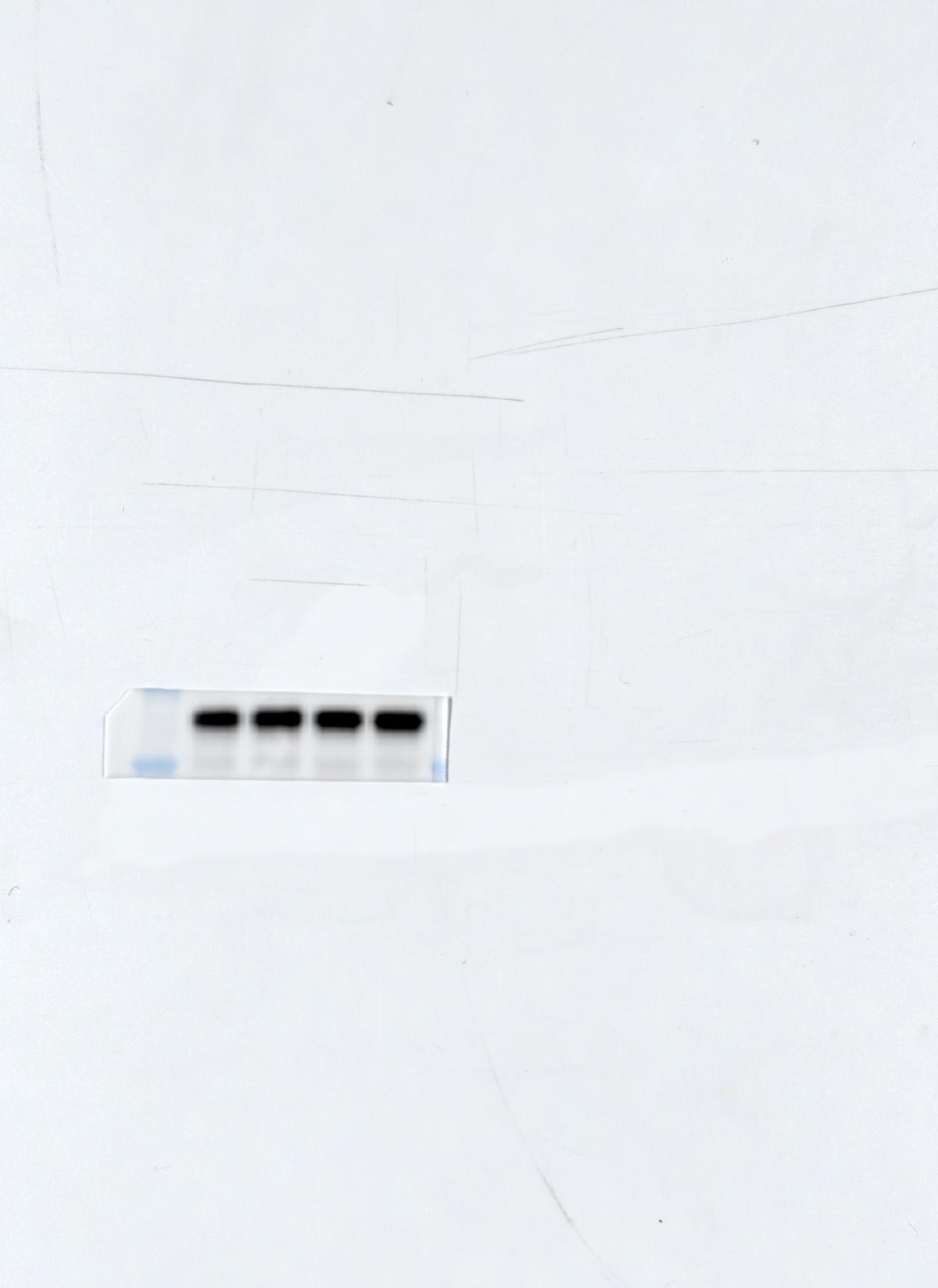

Supplement: Supplementary file 1 [file vetsci-12-01186-s001.zip › Supplementary Files/WB uncropped figure/Figure S4/GAPDH 2 20250429_140856_Ch/GAPDH 1 20250429_140856_Ch_Chemi+Marker.jpg]

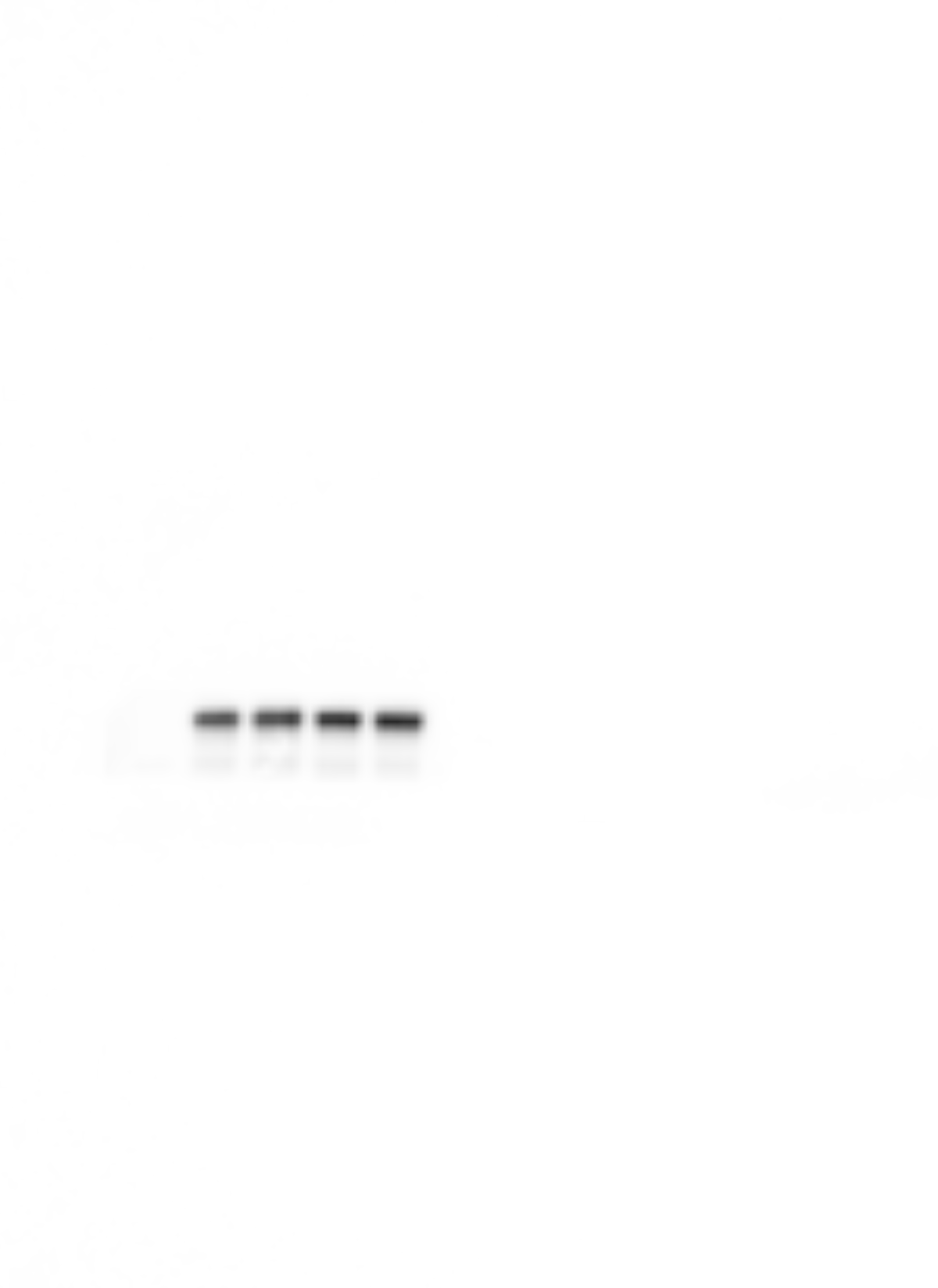

Supplement: Supplementary file 1 [file vetsci-12-01186-s001.zip › Supplementary Files/WB uncropped figure/Figure S4/GAPDH 2 20250429_140856_Ch/GAPDH 1 20250429_140856_Ch_Chemi.tif]

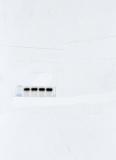

Supplement: Supplementary file 1 [file vetsci-12-01186-s001.zip › Supplementary Files/WB uncropped figure/Figure S4/GAPDH 2 20250429_140856_Ch/GAPDH 1 20250429_140856_Ch_Thumb.jpg]

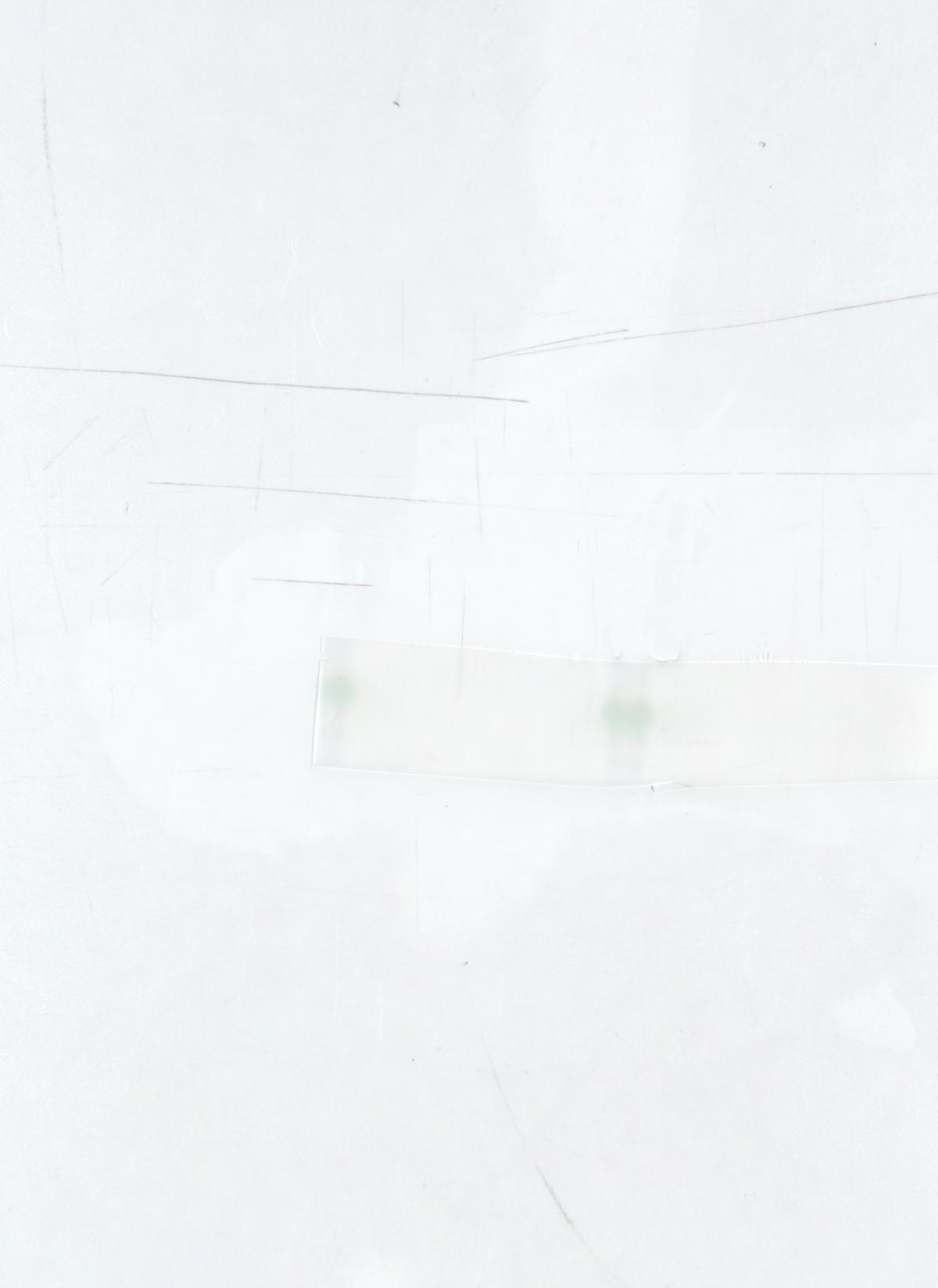

Supplement: Supplementary file 1 [file vetsci-12-01186-s001.zip › Supplementary Files/WB uncropped figure/Figure S4/hou bcl-2 2 20250620_145311_Ch/hou bcl-2 2 20250620_145311_Ch-Marker.jpg]

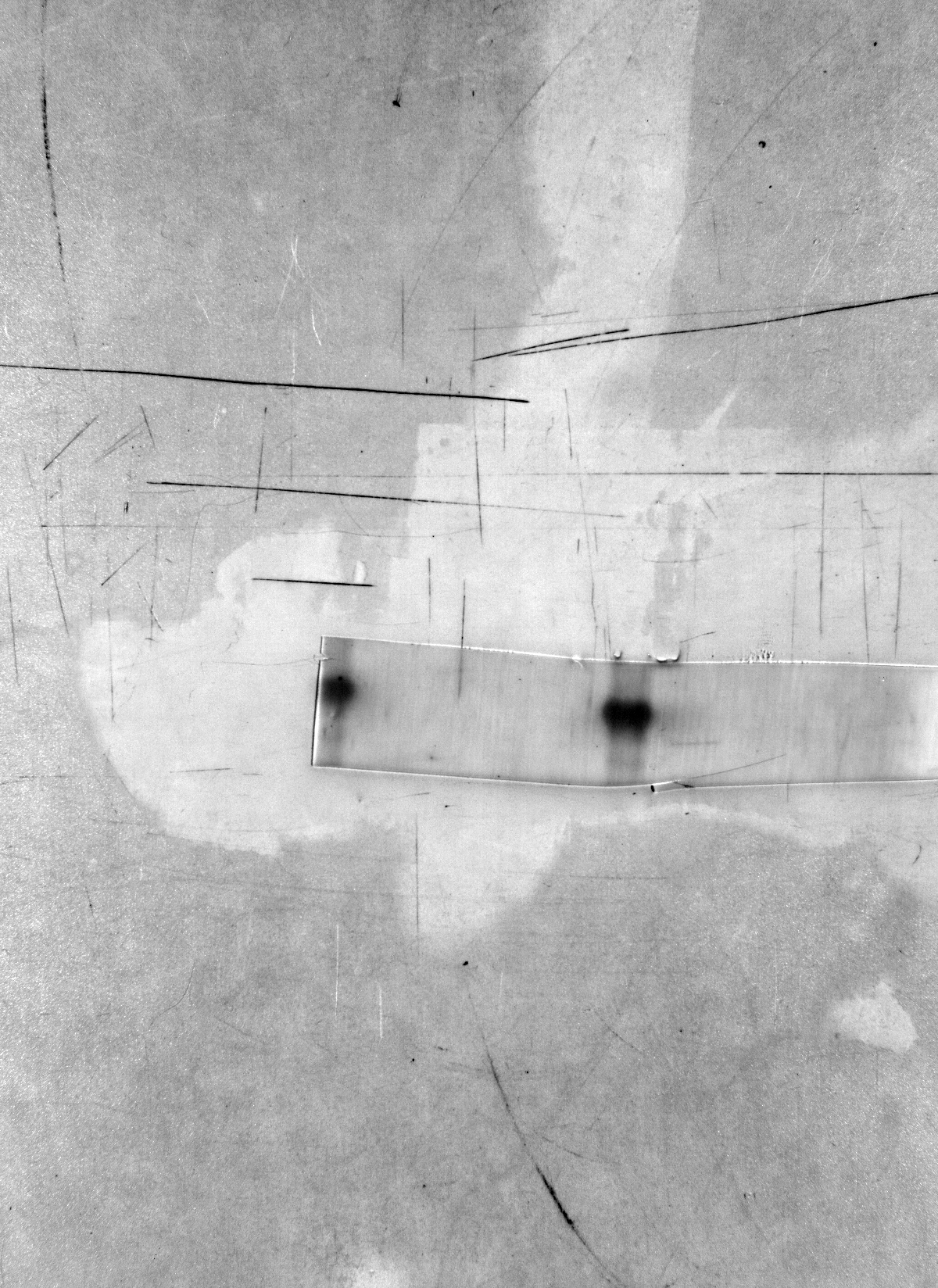

Supplement: Supplementary file 1 [file vetsci-12-01186-s001.zip › Supplementary Files/WB uncropped figure/Figure S4/hou bcl-2 2 20250620_145311_Ch/hou bcl-2 2 20250620_145311_Ch-Marker.tif]

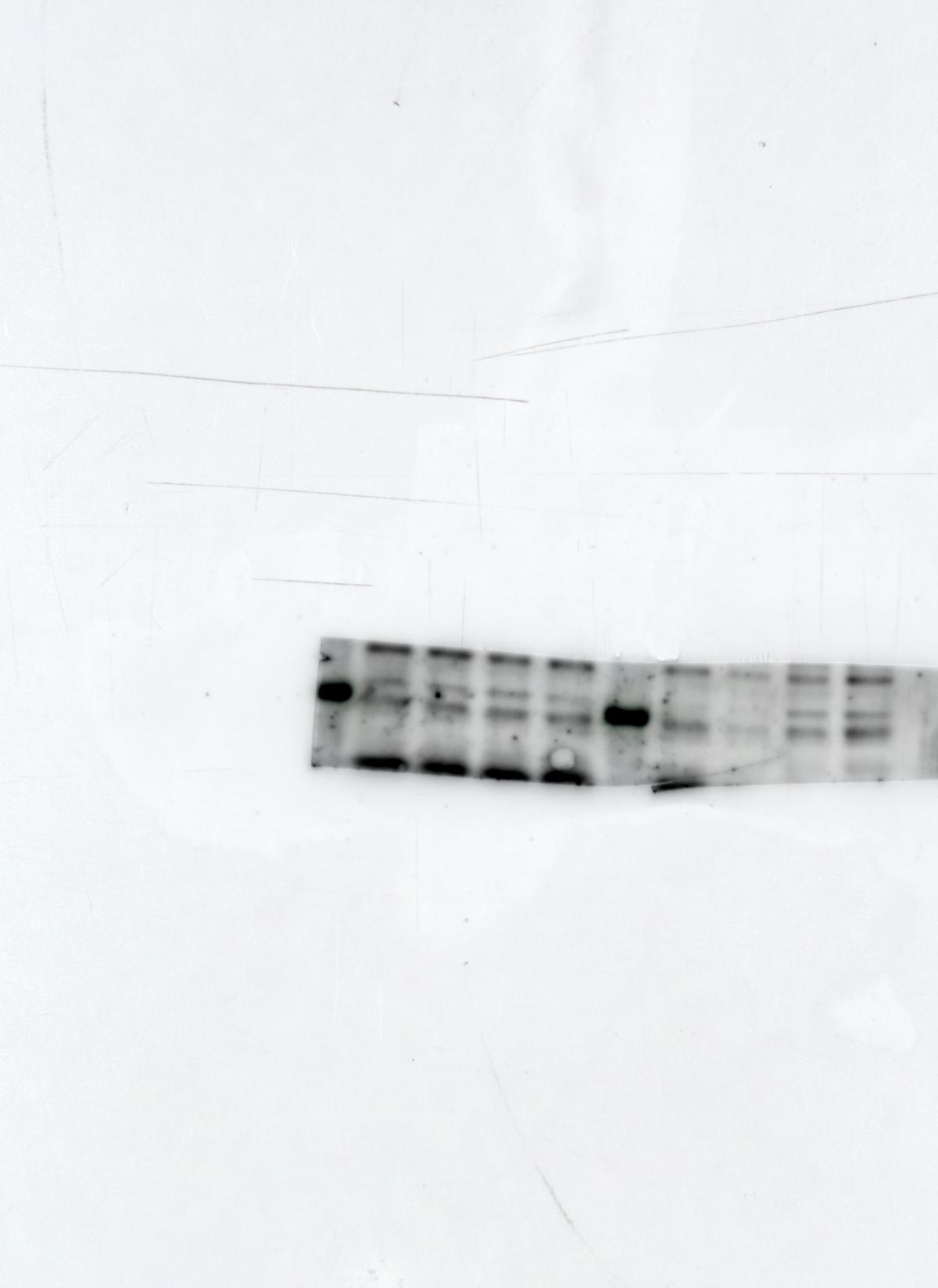

Supplement: Supplementary file 1 [file vetsci-12-01186-s001.zip › Supplementary Files/WB uncropped figure/Figure S4/hou bcl-2 2 20250620_145311_Ch/hou bcl-2 2 20250620_145311_Ch_Chemi+Marker.jpg]

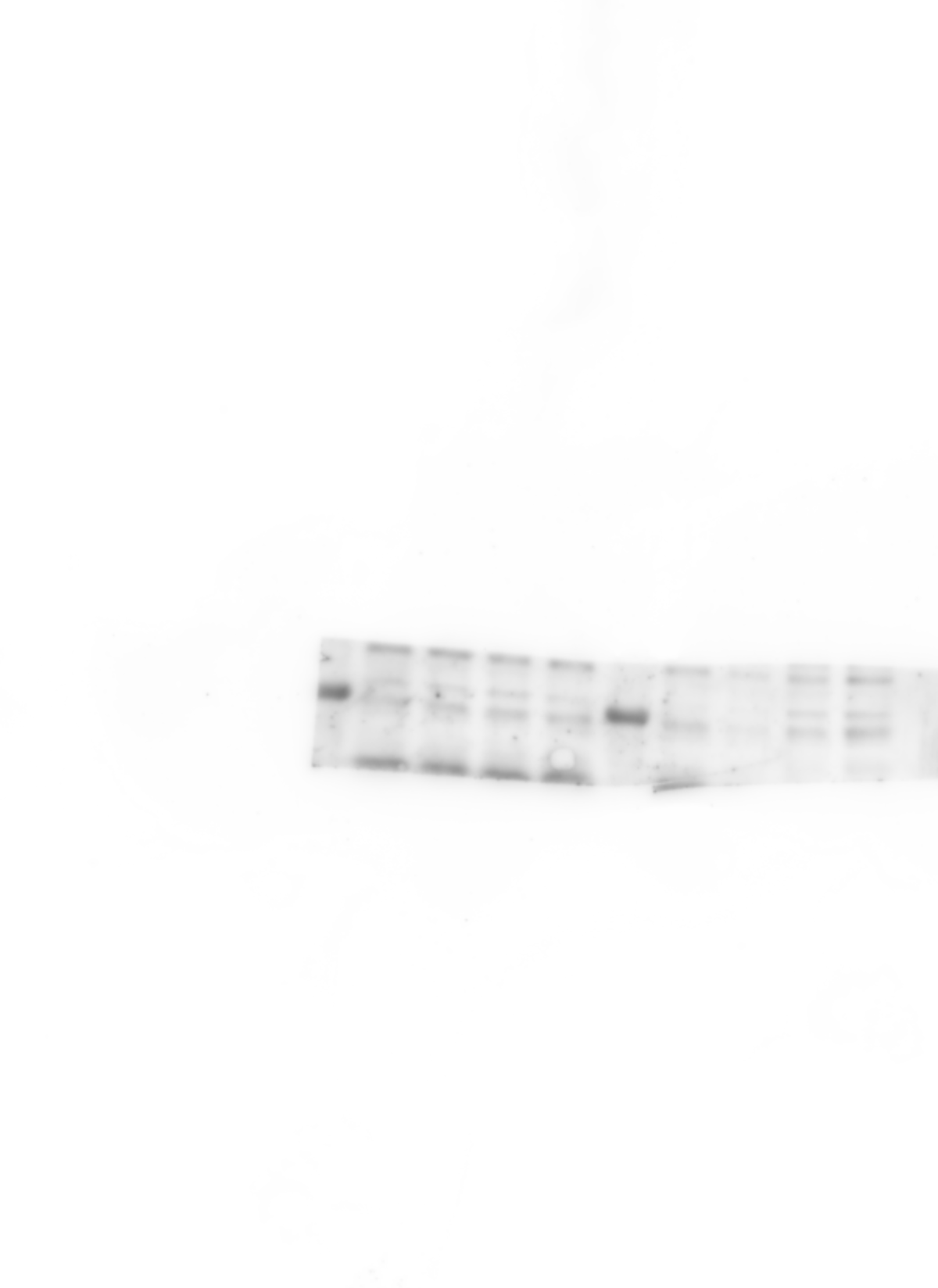

Supplement: Supplementary file 1 [file vetsci-12-01186-s001.zip › Supplementary Files/WB uncropped figure/Figure S4/hou bcl-2 2 20250620_145311_Ch/hou bcl-2 2 20250620_145311_Ch_Chemi.tif]

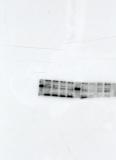

Supplement: Supplementary file 1 [file vetsci-12-01186-s001.zip › Supplementary Files/WB uncropped figure/Figure S4/hou bcl-2 2 20250620_145311_Ch/hou bcl-2 2 20250620_145311_Ch_Thumb.jpg]
