# Supplementary figures and images for: NEFA Promotes Bovine Granulosa Cell Apoptosis via Activation of the PERK/eIF2α/ATF4/CHOP Pathway (part 1 of 2)
Source: Vet Sci. 2025 Dec 11;12(12):1186. doi: 10.3390/vetsci12121186 (PMC12737492; doi:10.3390/vetsci12121186)

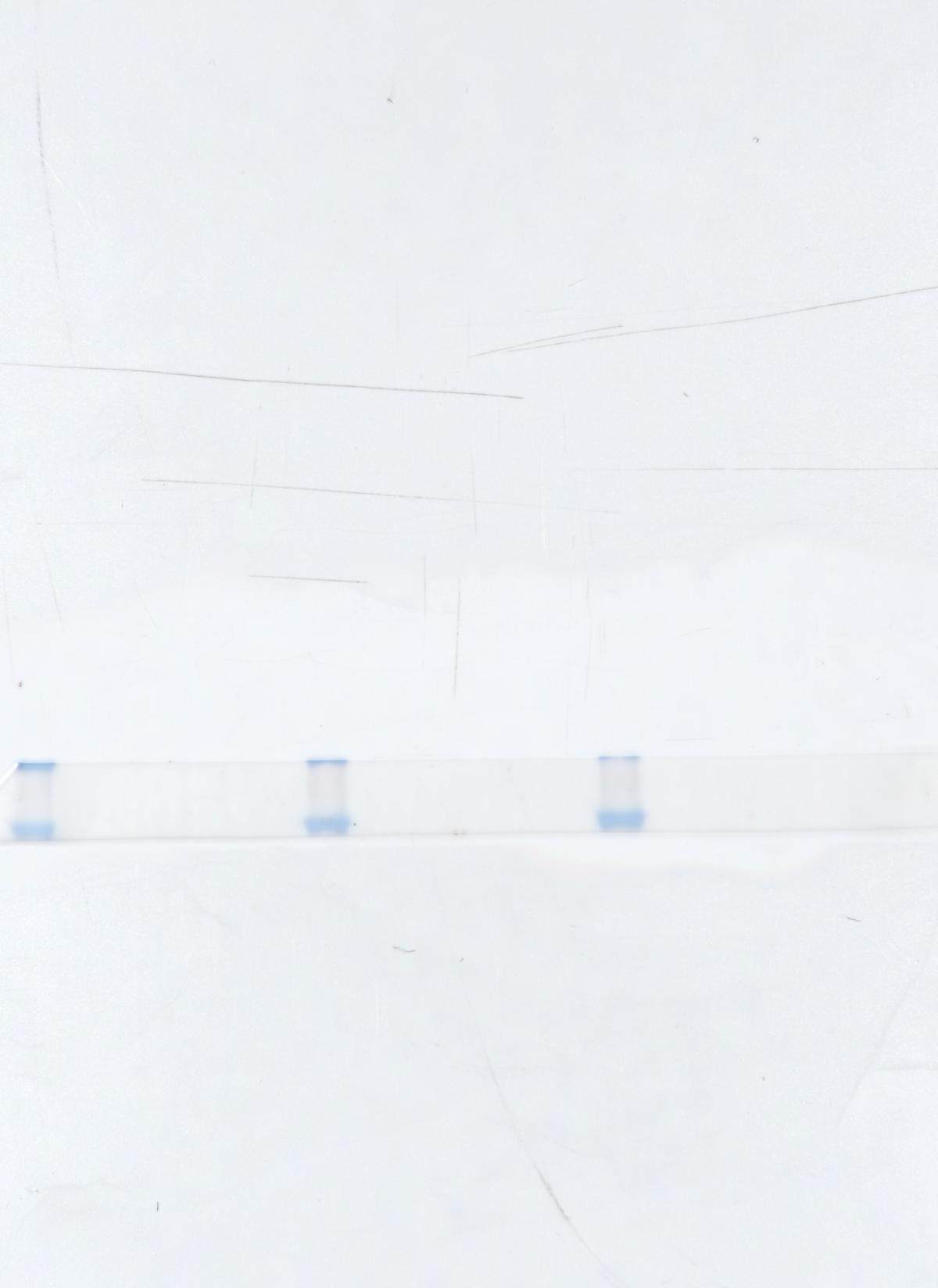

Supplement: Supplementary file 1 [file vetsci-12-01186-s001.zip › Supplementary Files/WB uncropped figure/Figure S1/ATF42 20250415_152523_Ch/ATF42 20250415_152523_Ch-Marker.jpg]

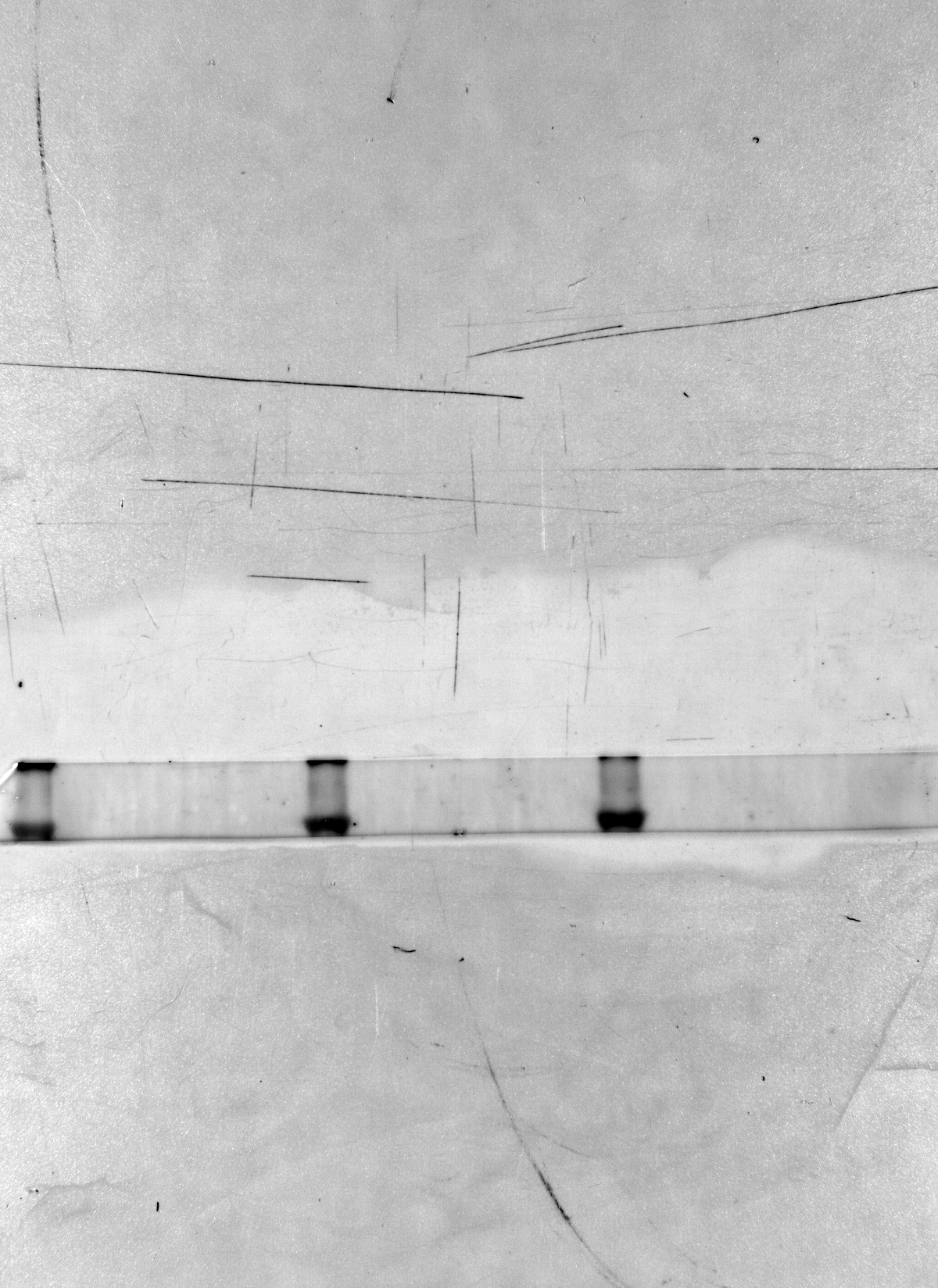

Supplement: Supplementary file 1 [file vetsci-12-01186-s001.zip › Supplementary Files/WB uncropped figure/Figure S1/ATF42 20250415_152523_Ch/ATF42 20250415_152523_Ch-Marker.tif]

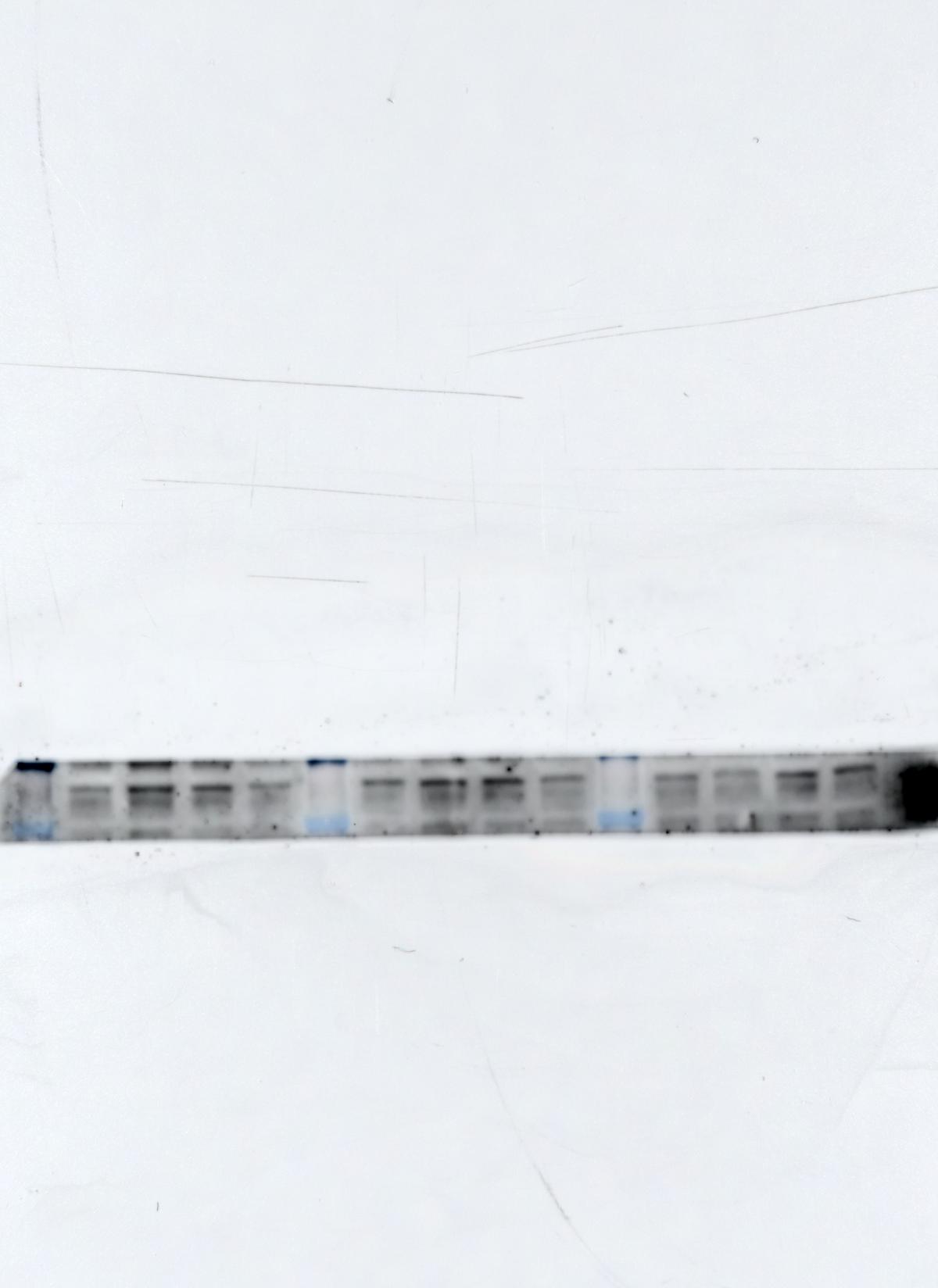

Supplement: Supplementary file 1 [file vetsci-12-01186-s001.zip › Supplementary Files/WB uncropped figure/Figure S1/ATF42 20250415_152523_Ch/ATF42 20250415_152523_Ch_Chemi+Marker.jpg]

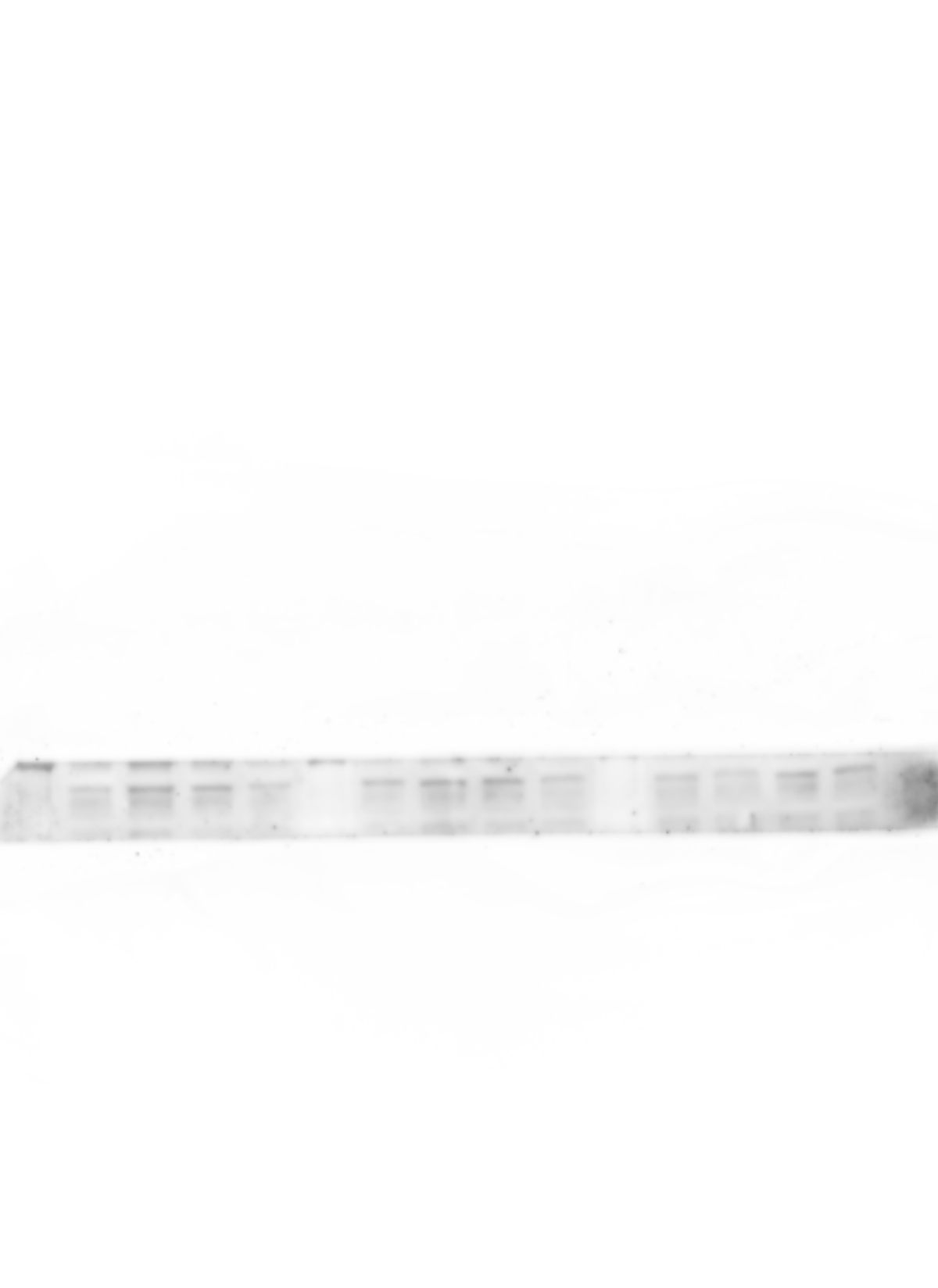

Supplement: Supplementary file 1 [file vetsci-12-01186-s001.zip › Supplementary Files/WB uncropped figure/Figure S1/ATF42 20250415_152523_Ch/ATF42 20250415_152523_Ch_Chemi.tif]

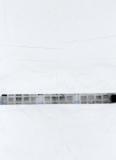

Supplement: Supplementary file 1 [file vetsci-12-01186-s001.zip › Supplementary Files/WB uncropped figure/Figure S1/ATF42 20250415_152523_Ch/ATF42 20250415_152523_Ch_Thumb.jpg]

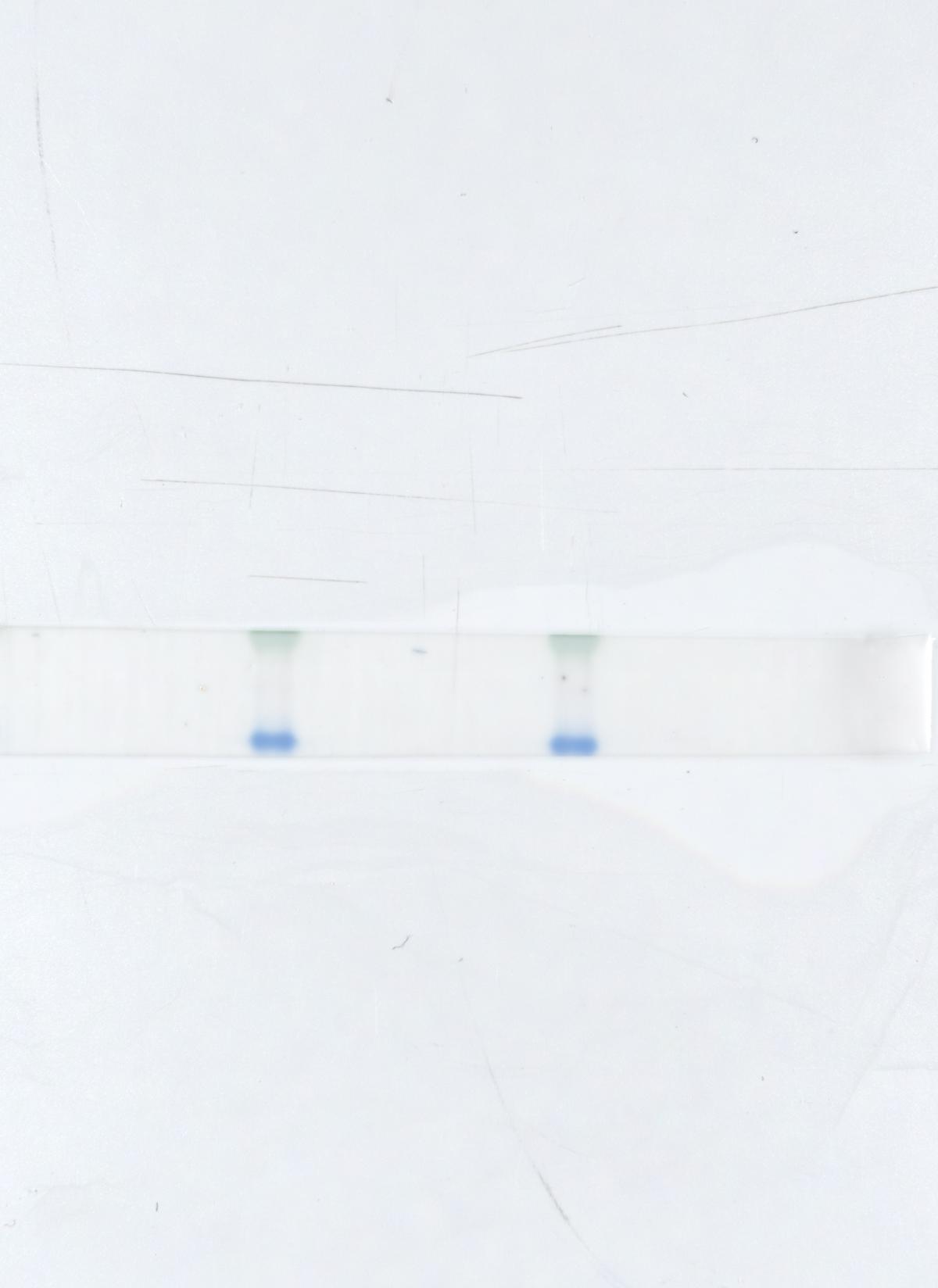

Supplement: Supplementary file 1 [file vetsci-12-01186-s001.zip › Supplementary Files/WB uncropped figure/Figure S1/BAX2 20250415_152950_Ch/BAX2 20250415_152950_Ch-Marker.jpg]

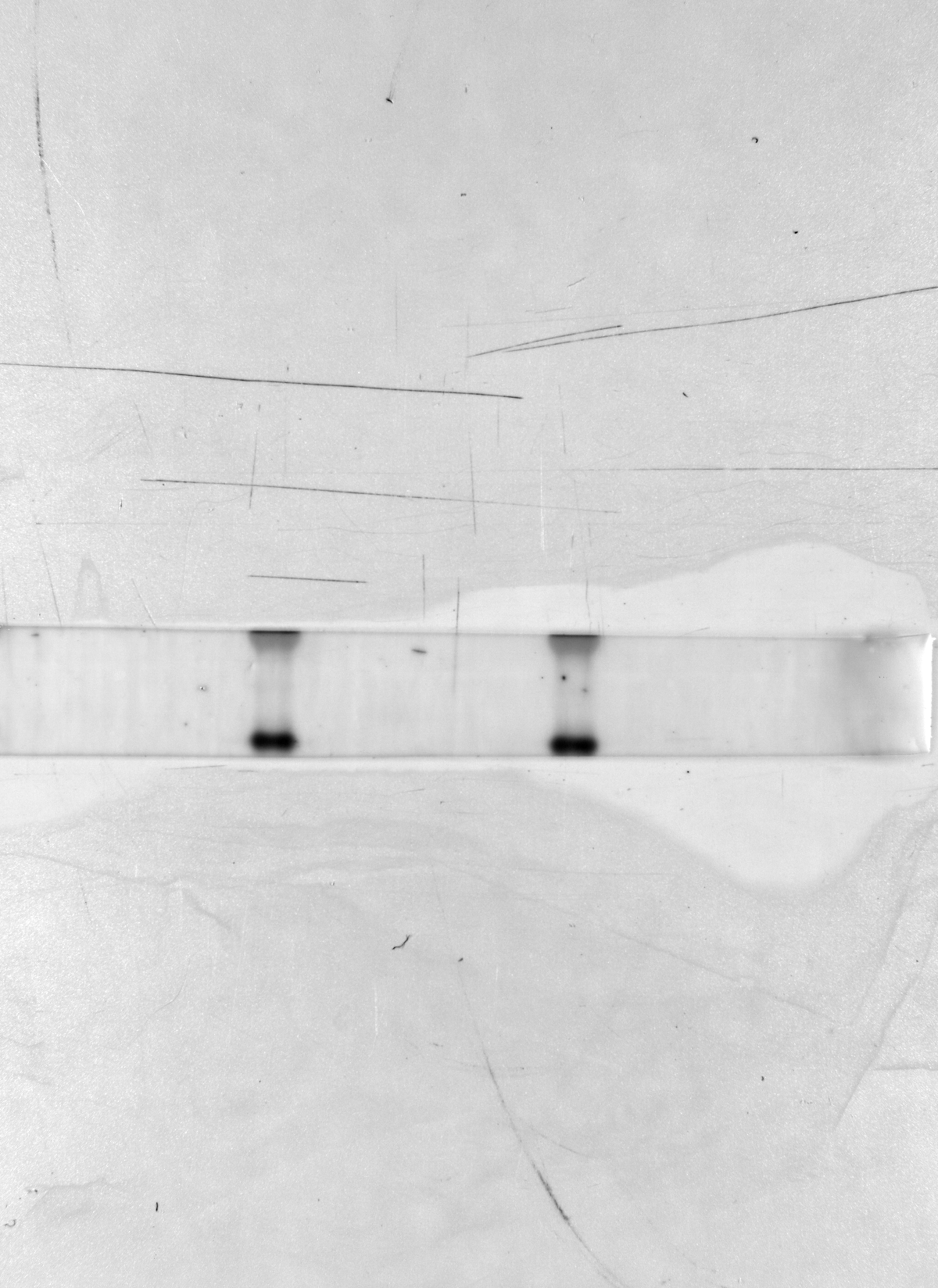

Supplement: Supplementary file 1 [file vetsci-12-01186-s001.zip › Supplementary Files/WB uncropped figure/Figure S1/BAX2 20250415_152950_Ch/BAX2 20250415_152950_Ch-Marker.tif]

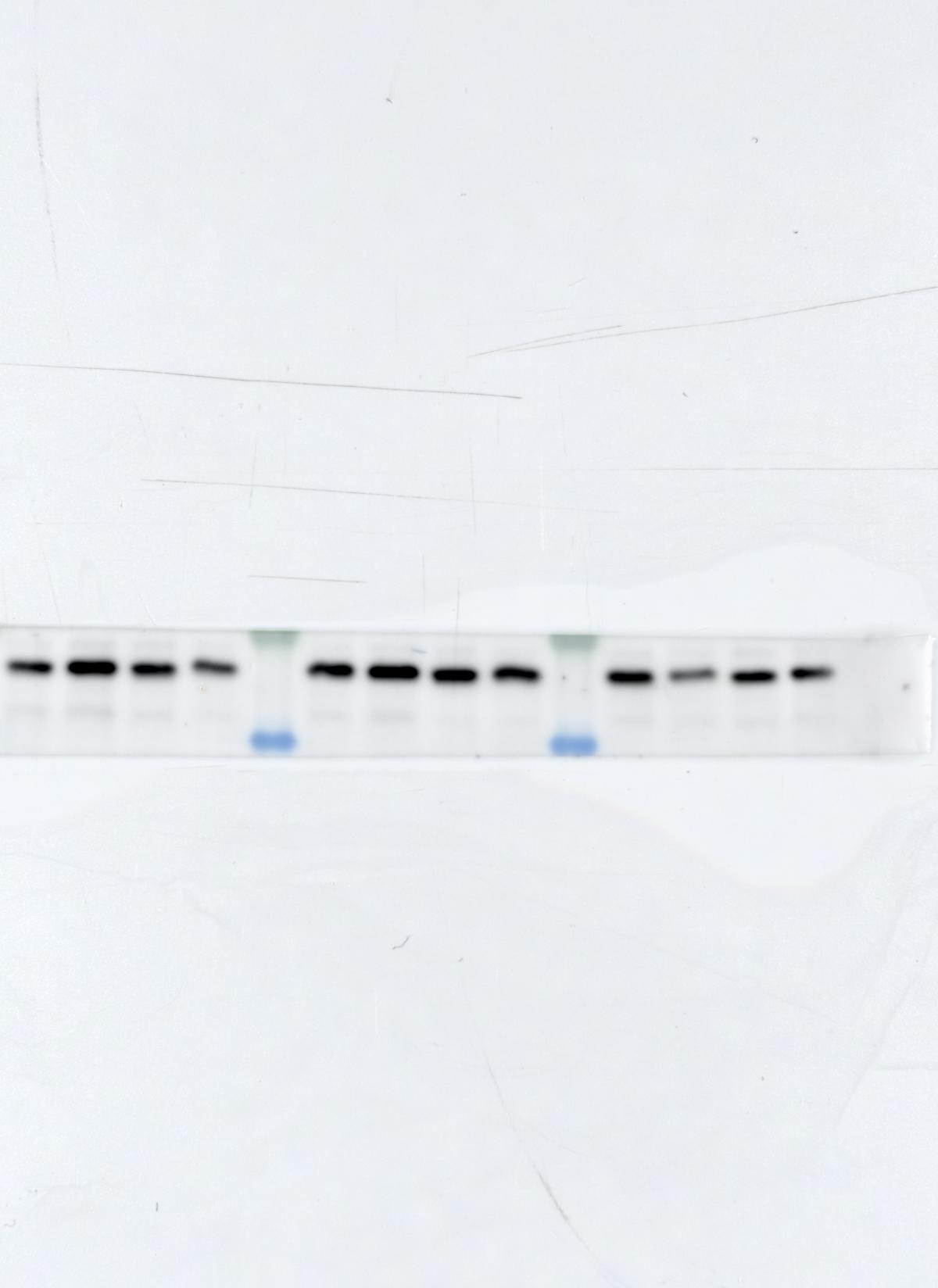

Supplement: Supplementary file 1 [file vetsci-12-01186-s001.zip › Supplementary Files/WB uncropped figure/Figure S1/BAX2 20250415_152950_Ch/BAX2 20250415_152950_Ch_Chemi+Marker.jpg]

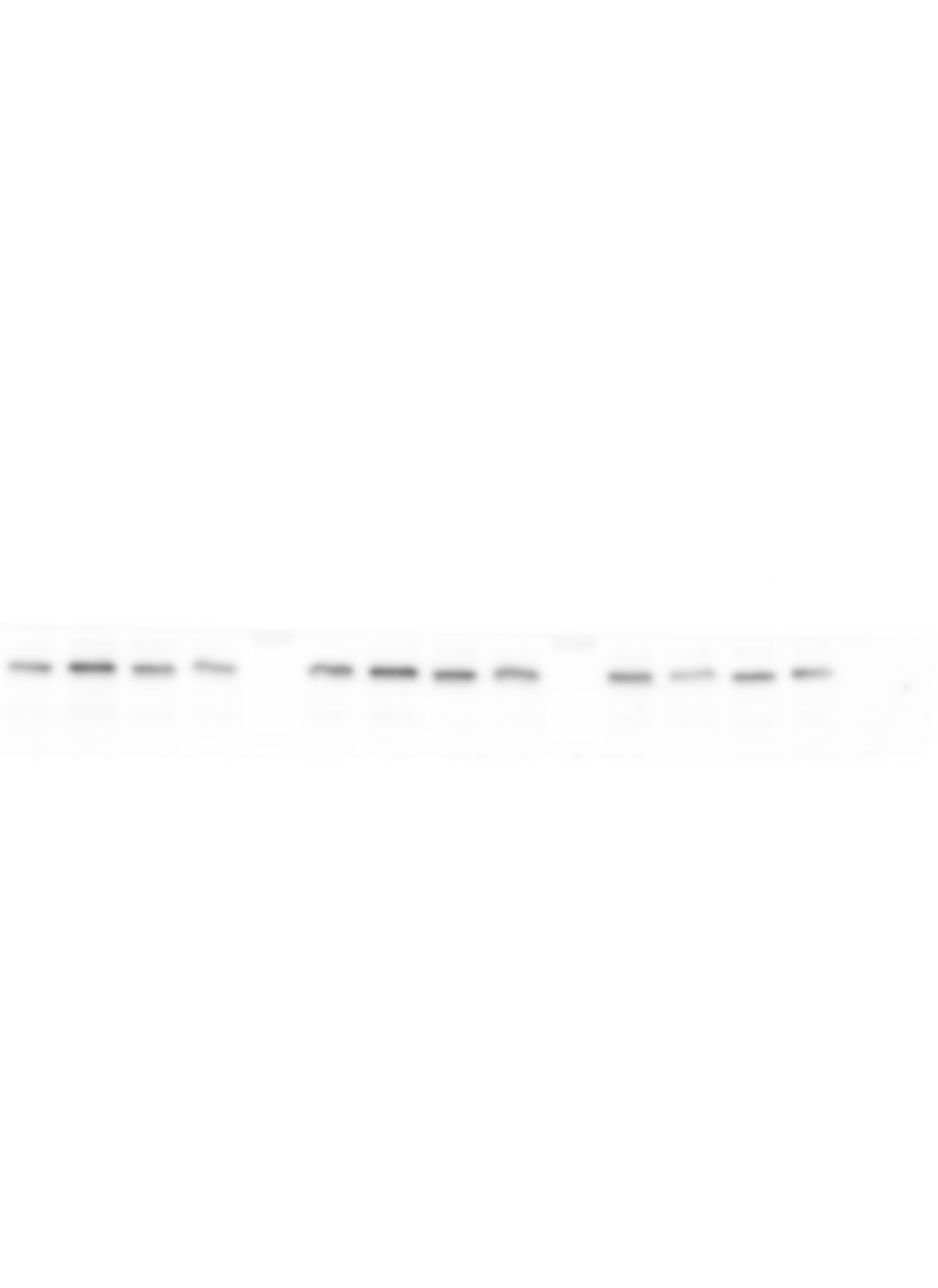

Supplement: Supplementary file 1 [file vetsci-12-01186-s001.zip › Supplementary Files/WB uncropped figure/Figure S1/BAX2 20250415_152950_Ch/BAX2 20250415_152950_Ch_Chemi.tif]

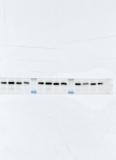

Supplement: Supplementary file 1 [file vetsci-12-01186-s001.zip › Supplementary Files/WB uncropped figure/Figure S1/BAX2 20250415_152950_Ch/BAX2 20250415_152950_Ch_Thumb.jpg]

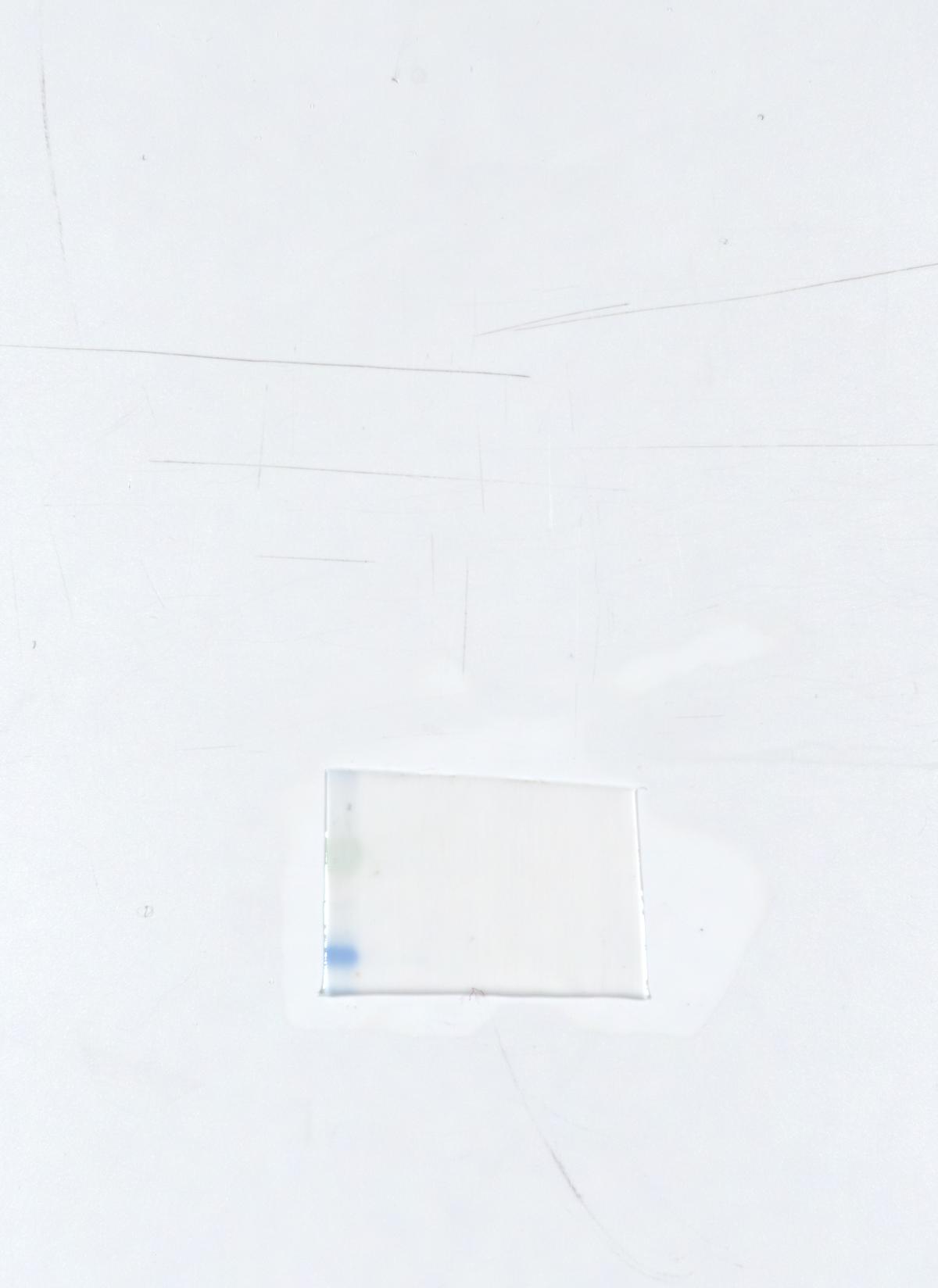

Supplement: Supplementary file 1 [file vetsci-12-01186-s001.zip › Supplementary Files/WB uncropped figure/Figure S1/BCL-2-2 20250402_151423_Ch/BCL-2-2 20250402_151423_Ch-Marker.jpg]

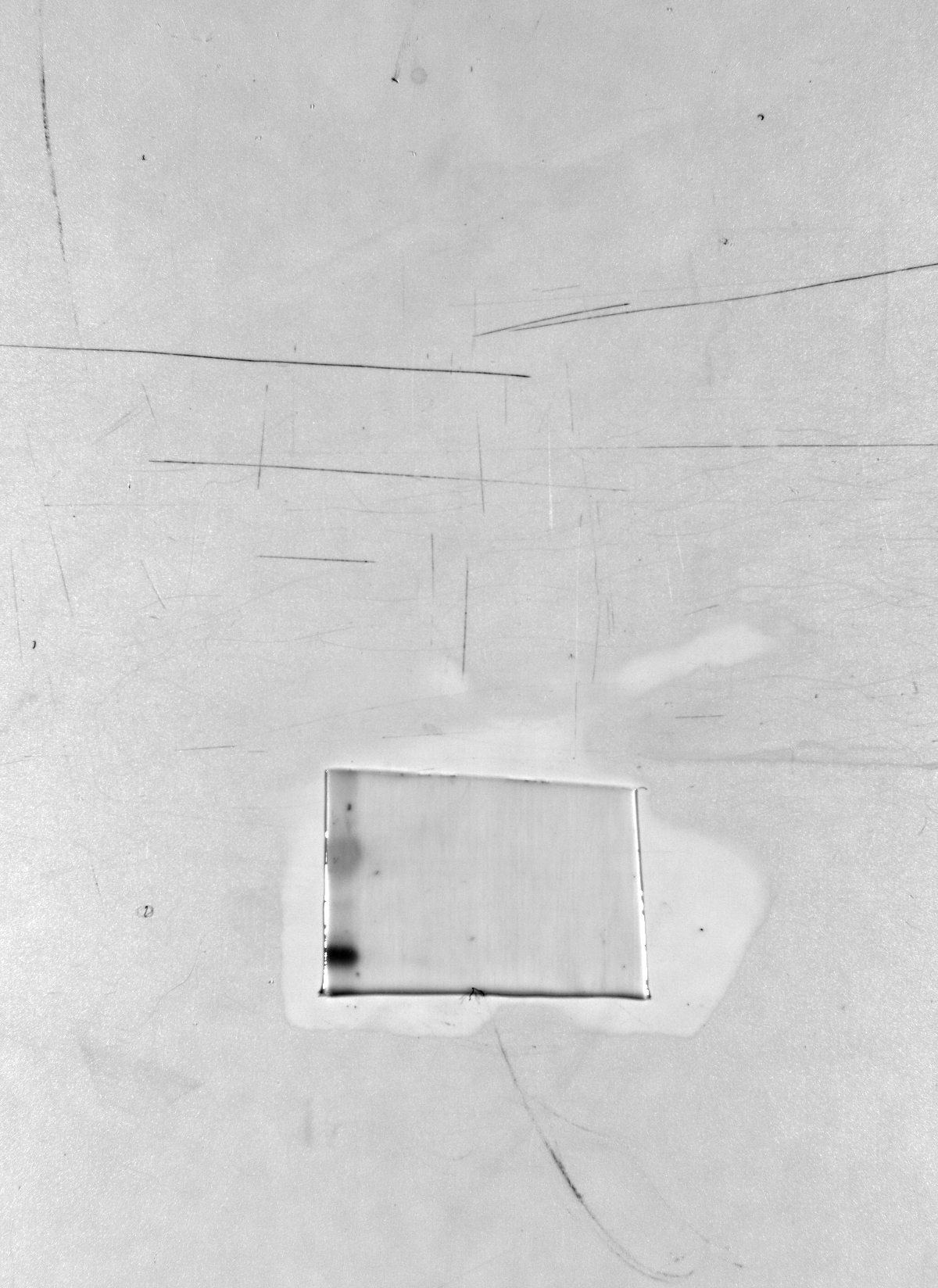

Supplement: Supplementary file 1 [file vetsci-12-01186-s001.zip › Supplementary Files/WB uncropped figure/Figure S1/BCL-2-2 20250402_151423_Ch/BCL-2-2 20250402_151423_Ch-Marker.tif]

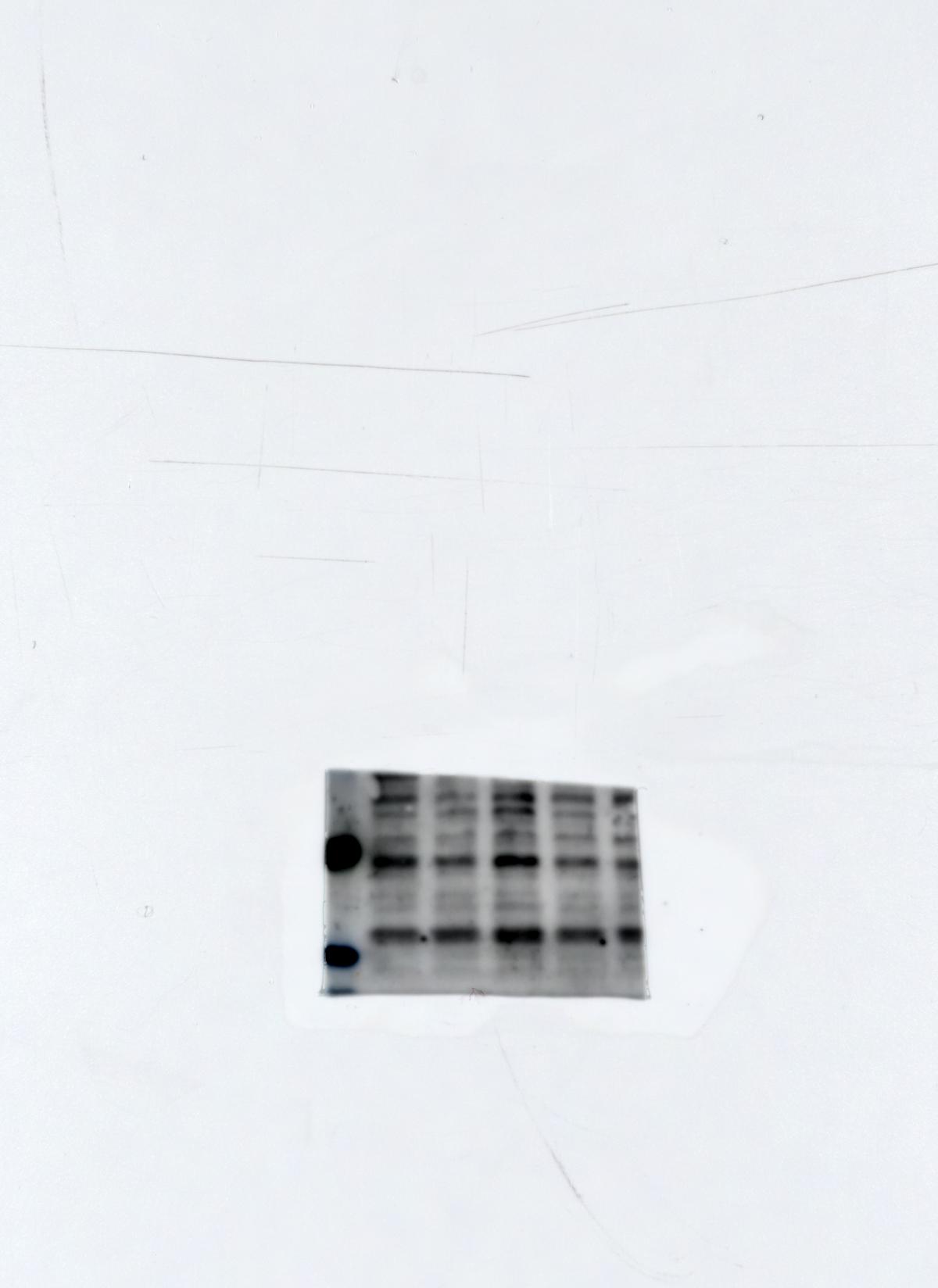

Supplement: Supplementary file 1 [file vetsci-12-01186-s001.zip › Supplementary Files/WB uncropped figure/Figure S1/BCL-2-2 20250402_151423_Ch/BCL-2-2 20250402_151423_Ch_Chemi+Marker.jpg]

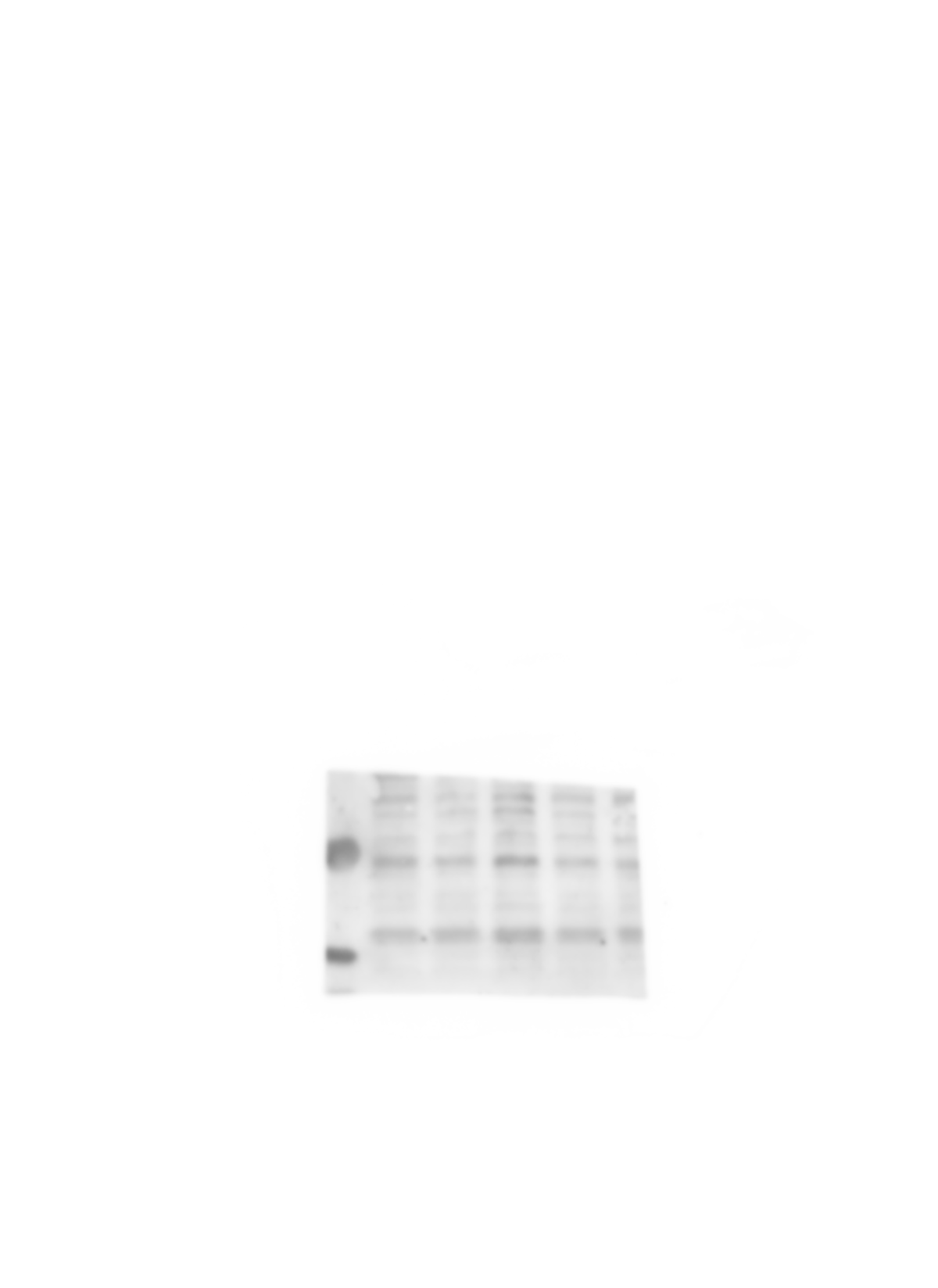

Supplement: Supplementary file 1 [file vetsci-12-01186-s001.zip › Supplementary Files/WB uncropped figure/Figure S1/BCL-2-2 20250402_151423_Ch/BCL-2-2 20250402_151423_Ch_Chemi.tif]

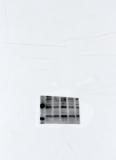

Supplement: Supplementary file 1 [file vetsci-12-01186-s001.zip › Supplementary Files/WB uncropped figure/Figure S1/BCL-2-2 20250402_151423_Ch/BCL-2-2 20250402_151423_Ch_Thumb.jpg]

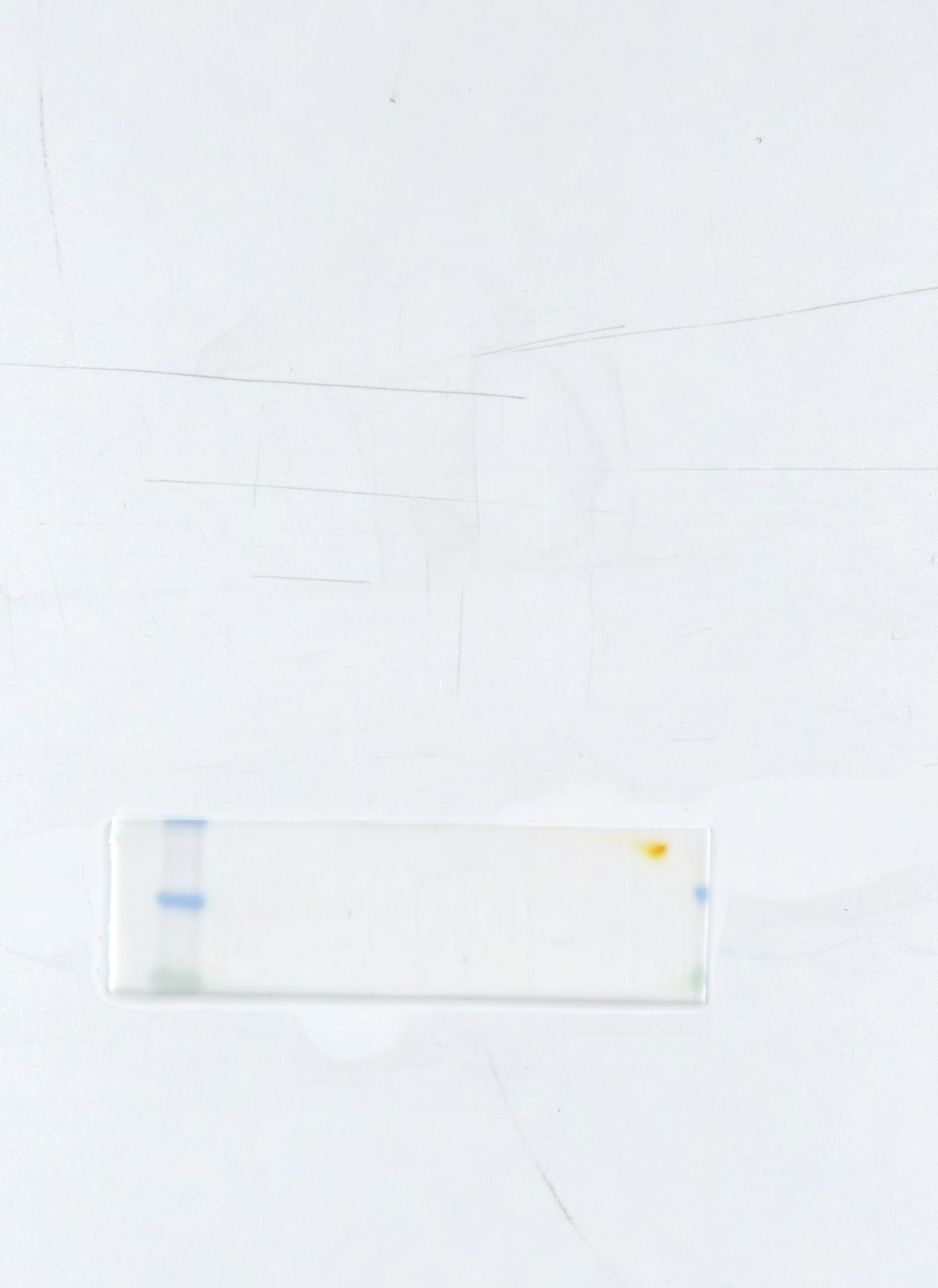

Supplement: Supplementary file 1 [file vetsci-12-01186-s001.zip › Supplementary Files/WB uncropped figure/Figure S1/C-3 11 20250426_124113_Ch/C-3 11 20250426_124113_Ch-Marker.jpg]

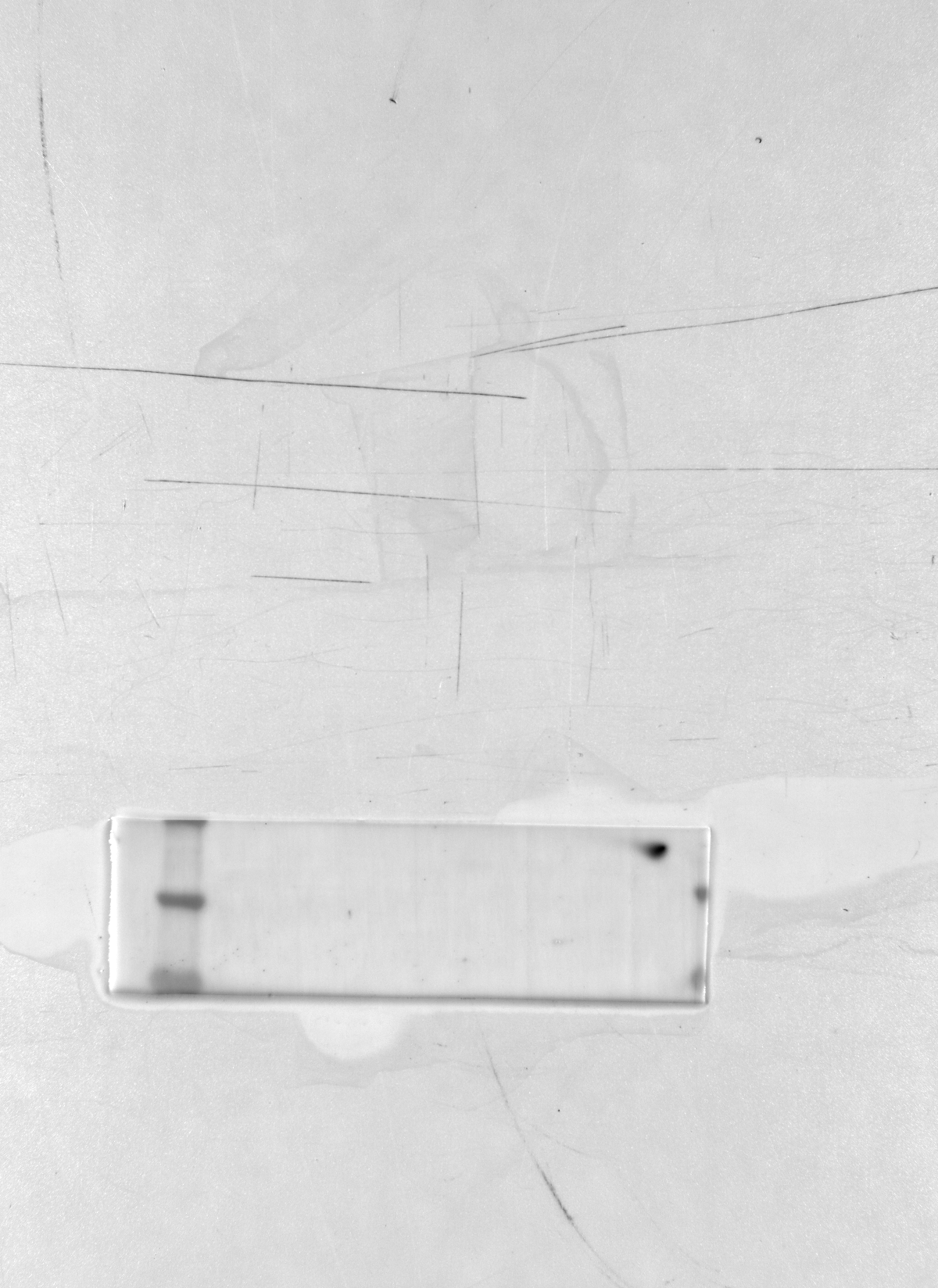

Supplement: Supplementary file 1 [file vetsci-12-01186-s001.zip › Supplementary Files/WB uncropped figure/Figure S1/C-3 11 20250426_124113_Ch/C-3 11 20250426_124113_Ch-Marker.tif]

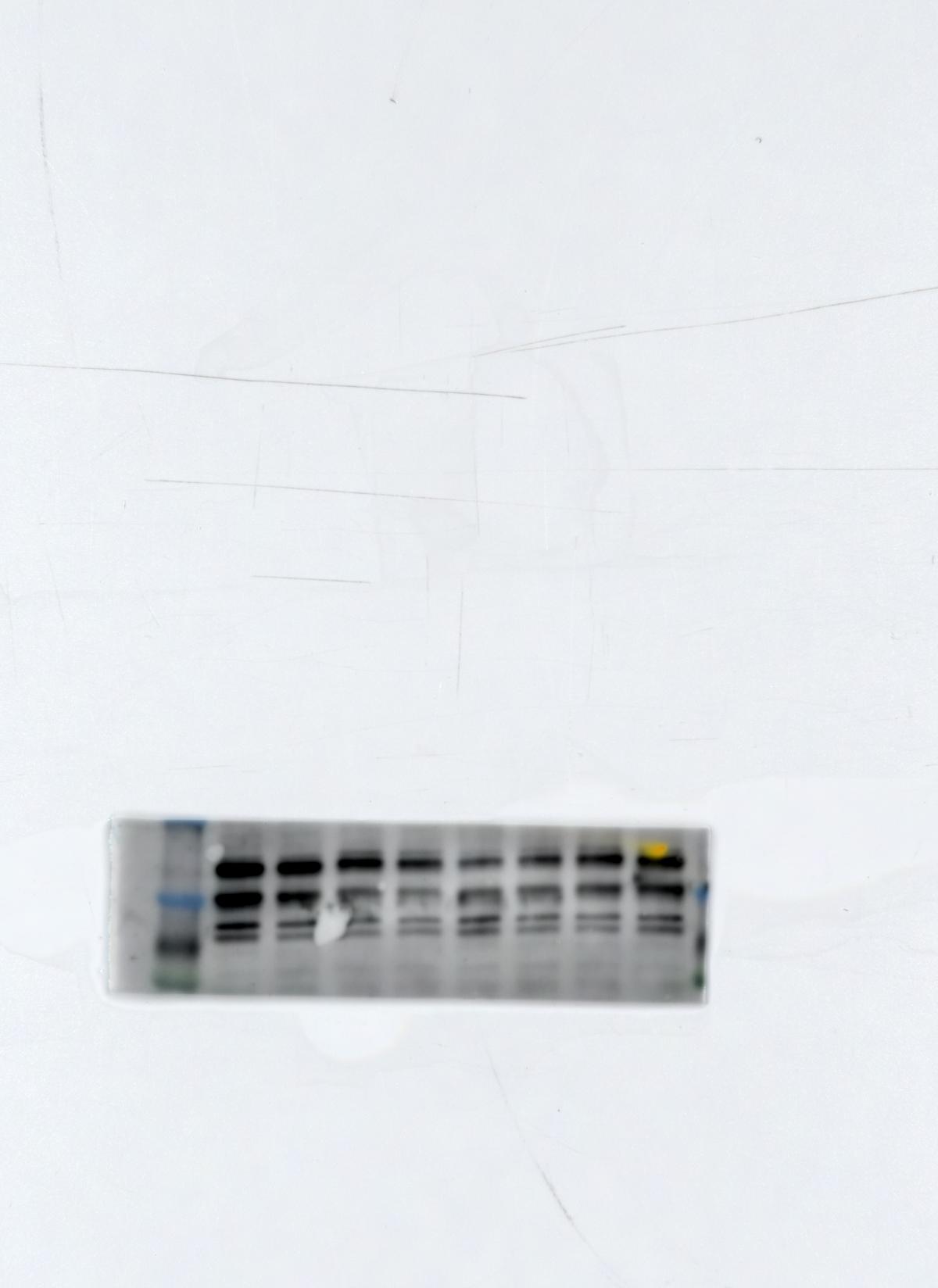

Supplement: Supplementary file 1 [file vetsci-12-01186-s001.zip › Supplementary Files/WB uncropped figure/Figure S1/C-3 11 20250426_124113_Ch/C-3 11 20250426_124113_Ch_Chemi+Marker.jpg]

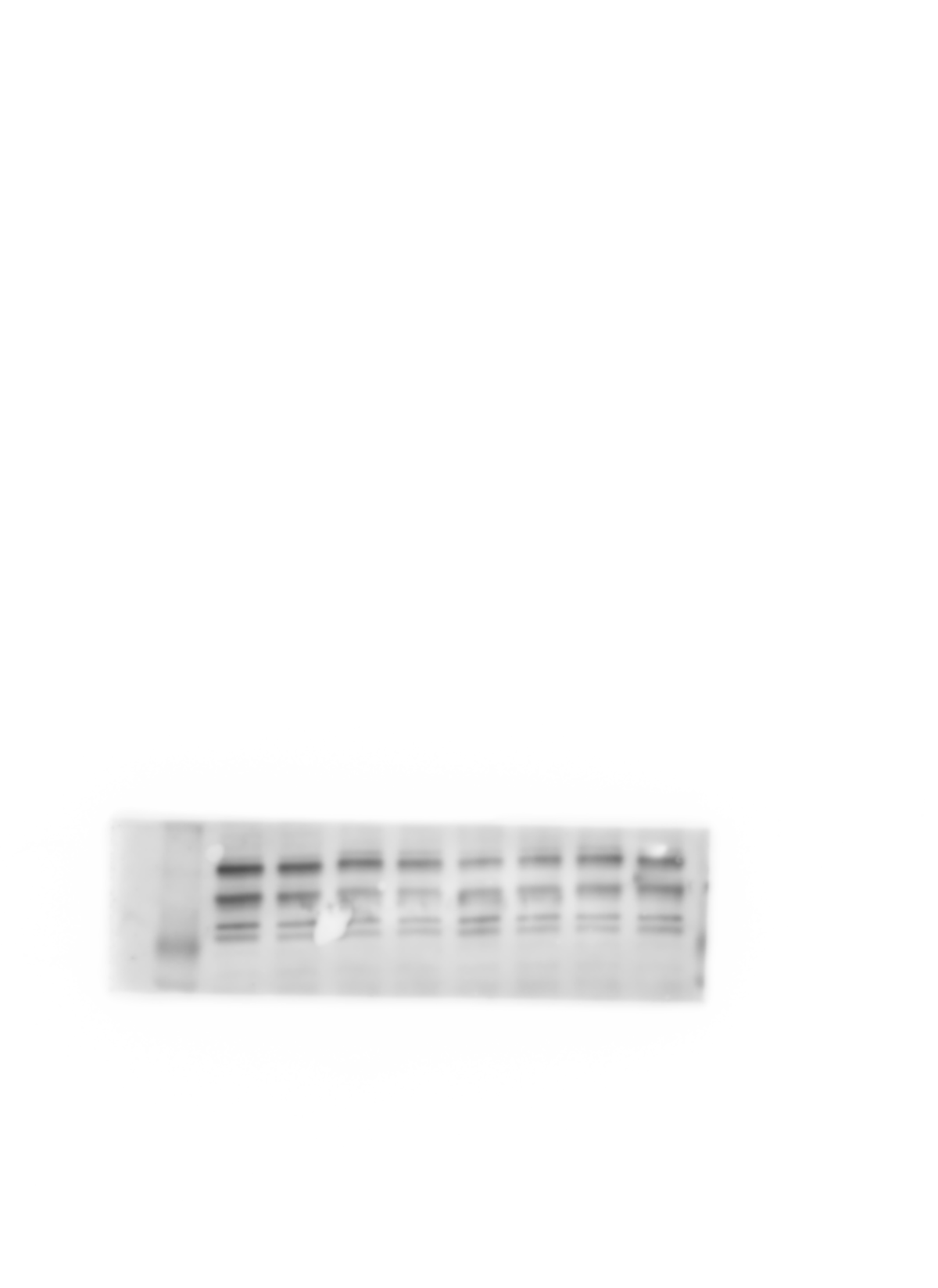

Supplement: Supplementary file 1 [file vetsci-12-01186-s001.zip › Supplementary Files/WB uncropped figure/Figure S1/C-3 11 20250426_124113_Ch/C-3 11 20250426_124113_Ch_Chemi.tif]

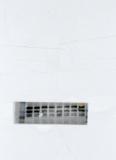

Supplement: Supplementary file 1 [file vetsci-12-01186-s001.zip › Supplementary Files/WB uncropped figure/Figure S1/C-3 11 20250426_124113_Ch/C-3 11 20250426_124113_Ch_Thumb.jpg]

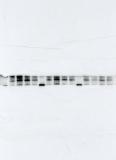

Supplement: Supplementary file 1 [file vetsci-12-01186-s001.zip › Supplementary Files/WB uncropped figure/Figure S1/CHOP 222 20250426/34a2f64fe35353ba672f39caea55b76.jpg]

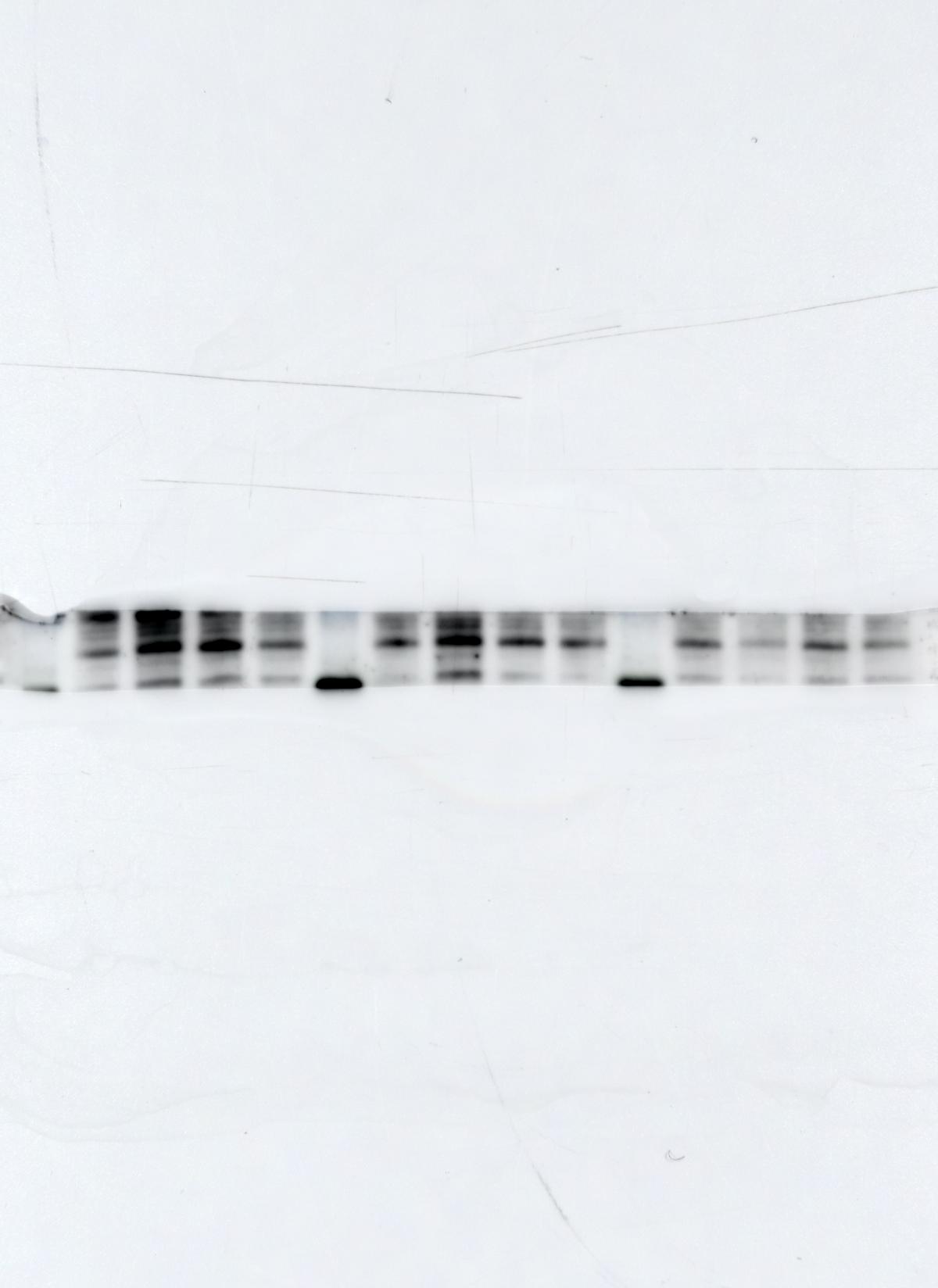

Supplement: Supplementary file 1 [file vetsci-12-01186-s001.zip › Supplementary Files/WB uncropped figure/Figure S1/CHOP 222 20250426/382db2ada1734659a6d7ae568d100f1.jpg]

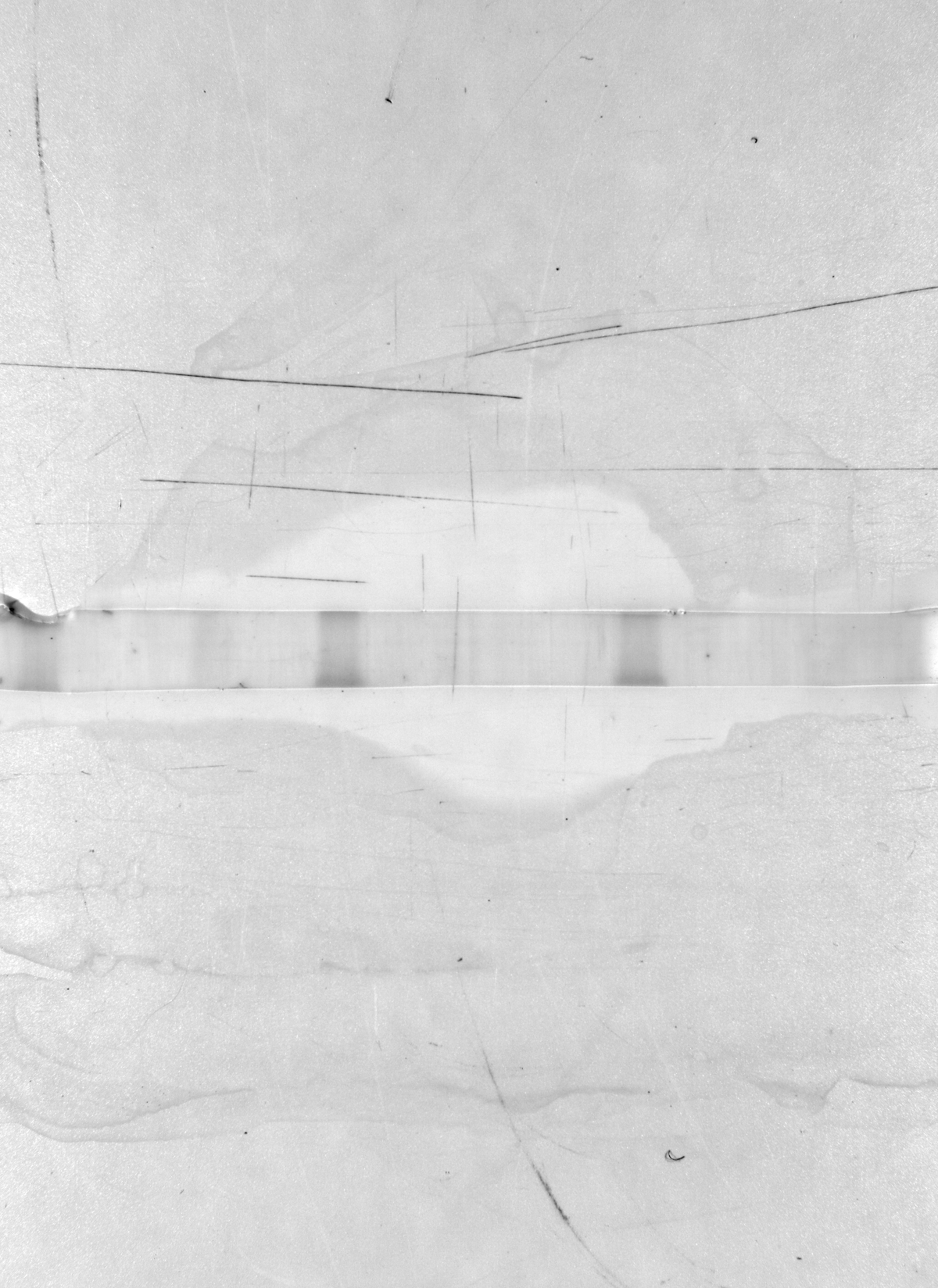

Supplement: Supplementary file 1 [file vetsci-12-01186-s001.zip › Supplementary Files/WB uncropped figure/Figure S1/CHOP 222 20250426/CHOP 20250426_142926_Ch-Marker.tif]

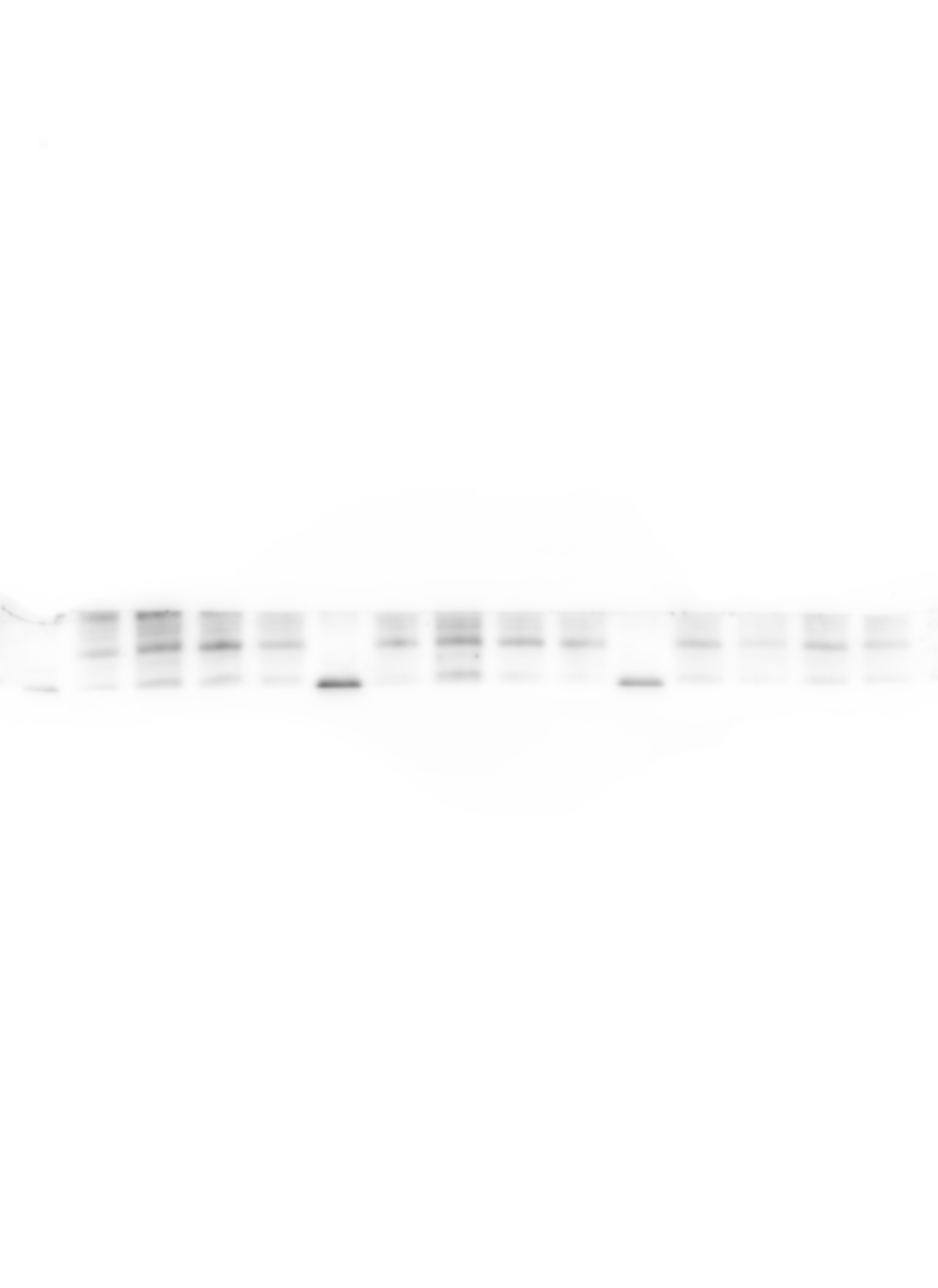

Supplement: Supplementary file 1 [file vetsci-12-01186-s001.zip › Supplementary Files/WB uncropped figure/Figure S1/CHOP 222 20250426/CHOP 20250426_142926_Ch_Chemi.tif]

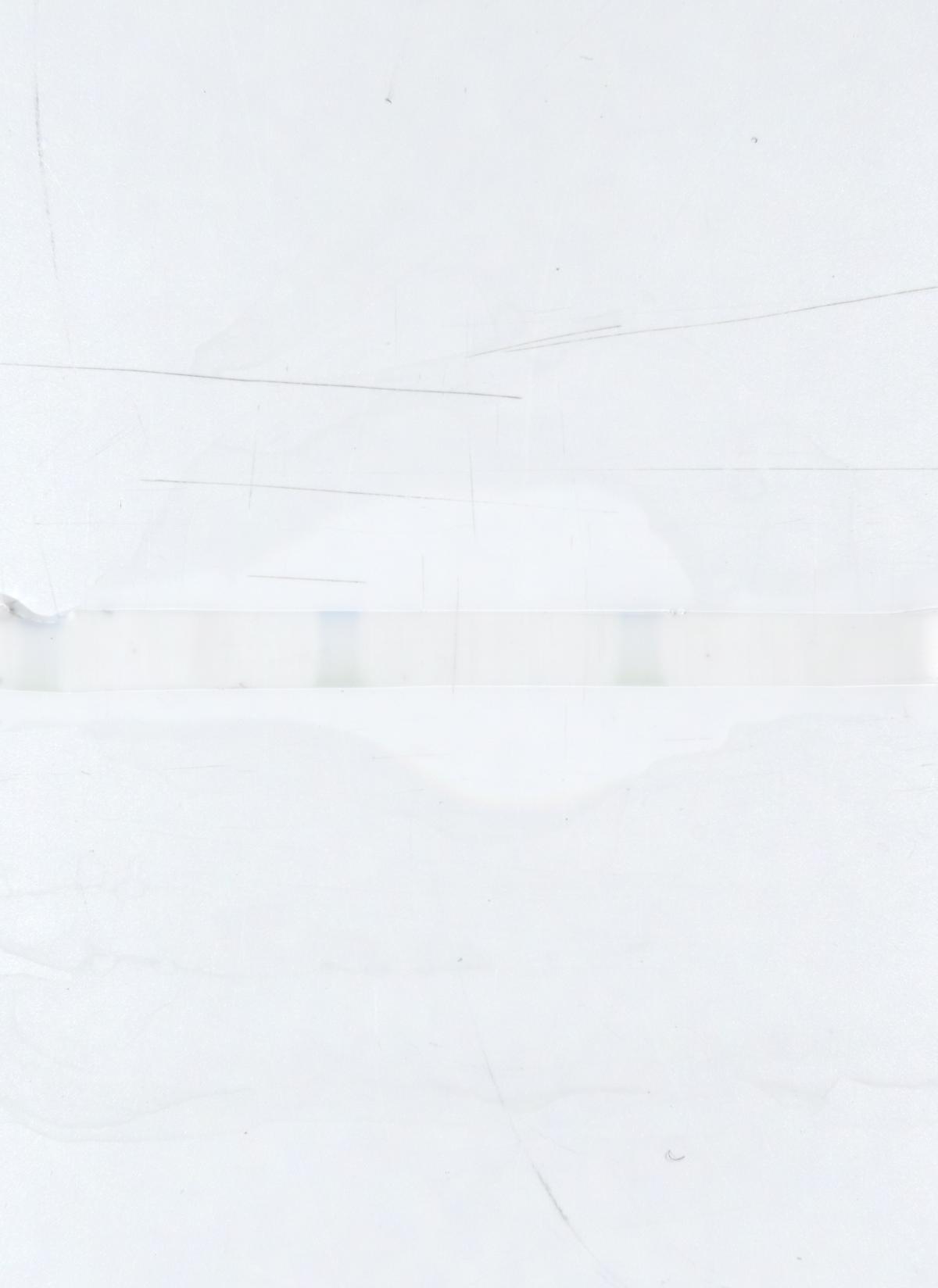

Supplement: Supplementary file 1 [file vetsci-12-01186-s001.zip › Supplementary Files/WB uncropped figure/Figure S1/CHOP 222 20250426/d4c91d650ea0de284926b29f82648de.jpg]

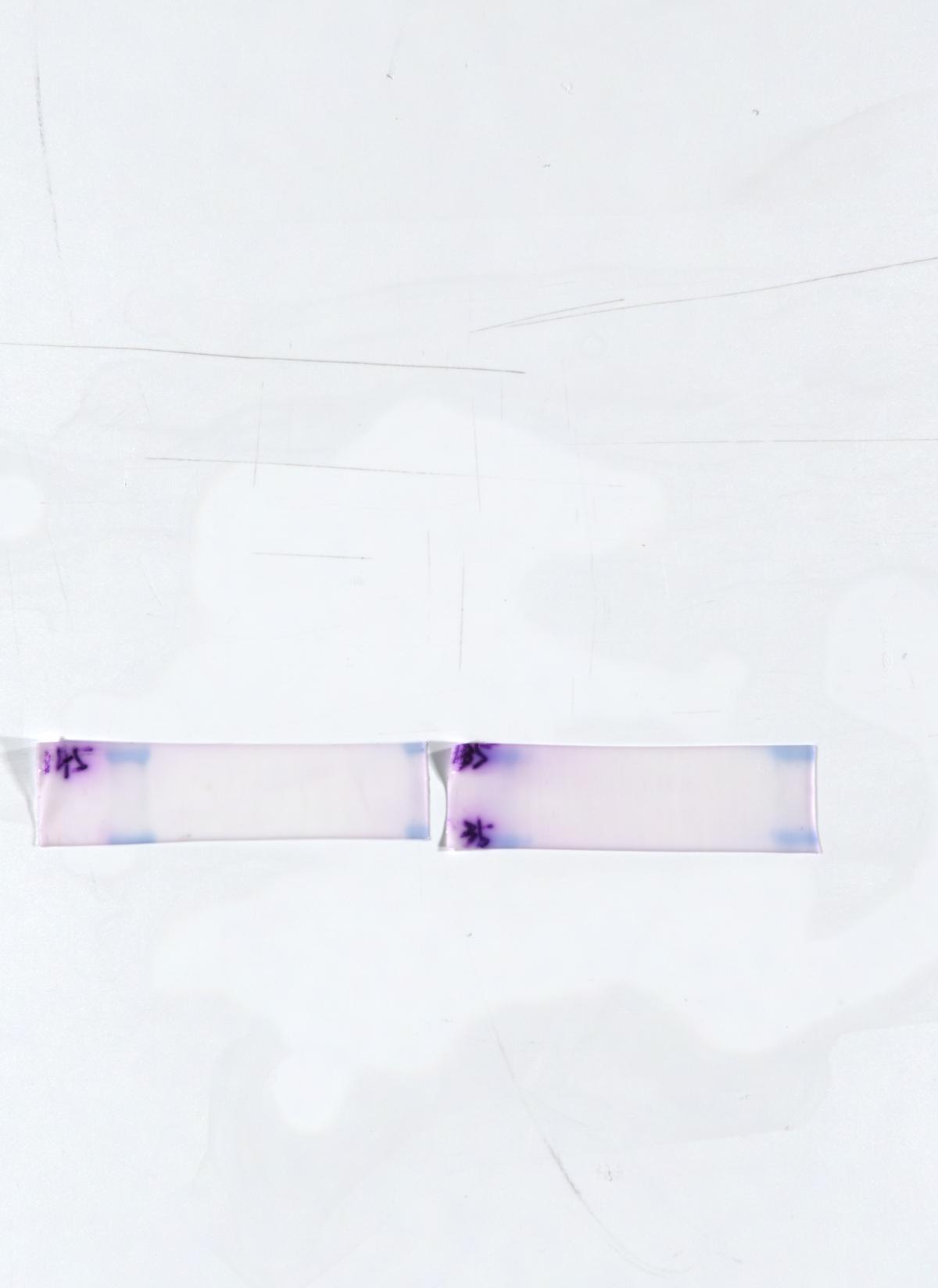

Supplement: Supplementary file 1 [file vetsci-12-01186-s001.zip › Supplementary Files/WB uncropped figure/Figure S1/eif2 20250330_102330_Ch/eif2 20250330_102330_Ch-Marker.jpg]

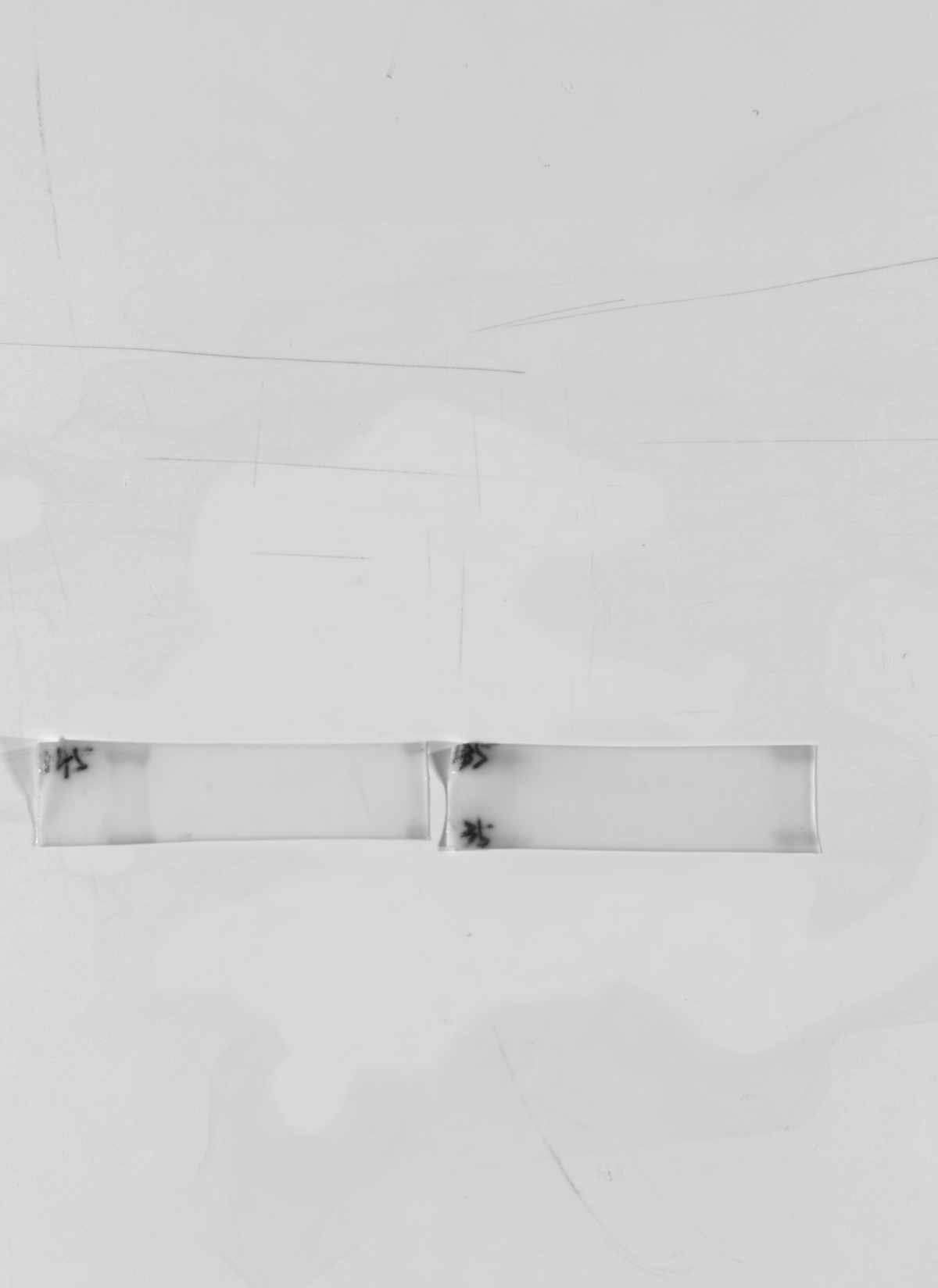

Supplement: Supplementary file 1 [file vetsci-12-01186-s001.zip › Supplementary Files/WB uncropped figure/Figure S1/eif2 20250330_102330_Ch/eif2 20250330_102330_Ch-Marker.tif]

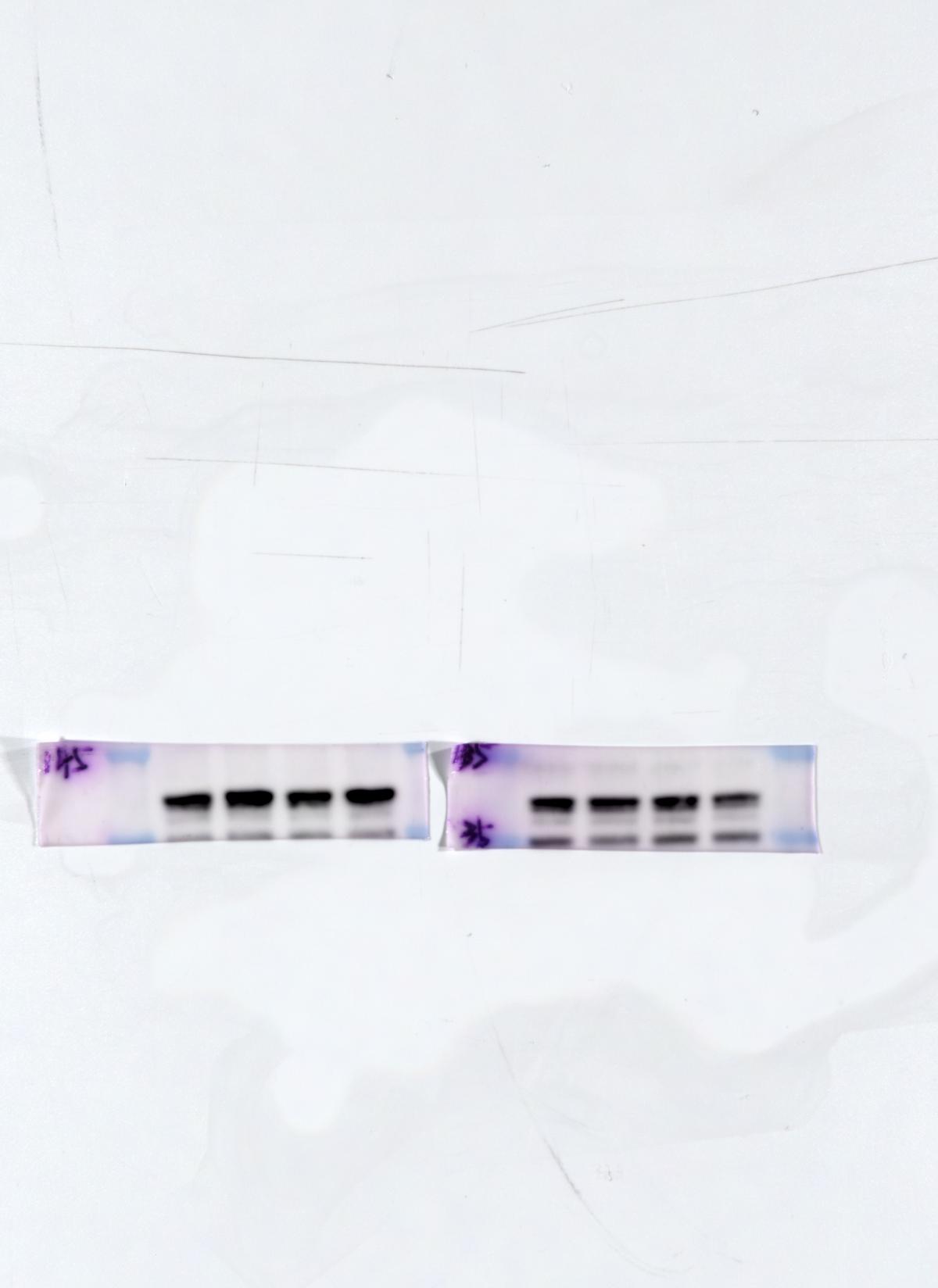

Supplement: Supplementary file 1 [file vetsci-12-01186-s001.zip › Supplementary Files/WB uncropped figure/Figure S1/eif2 20250330_102330_Ch/eif2 20250330_102330_Ch_Chemi+Marker.jpg]

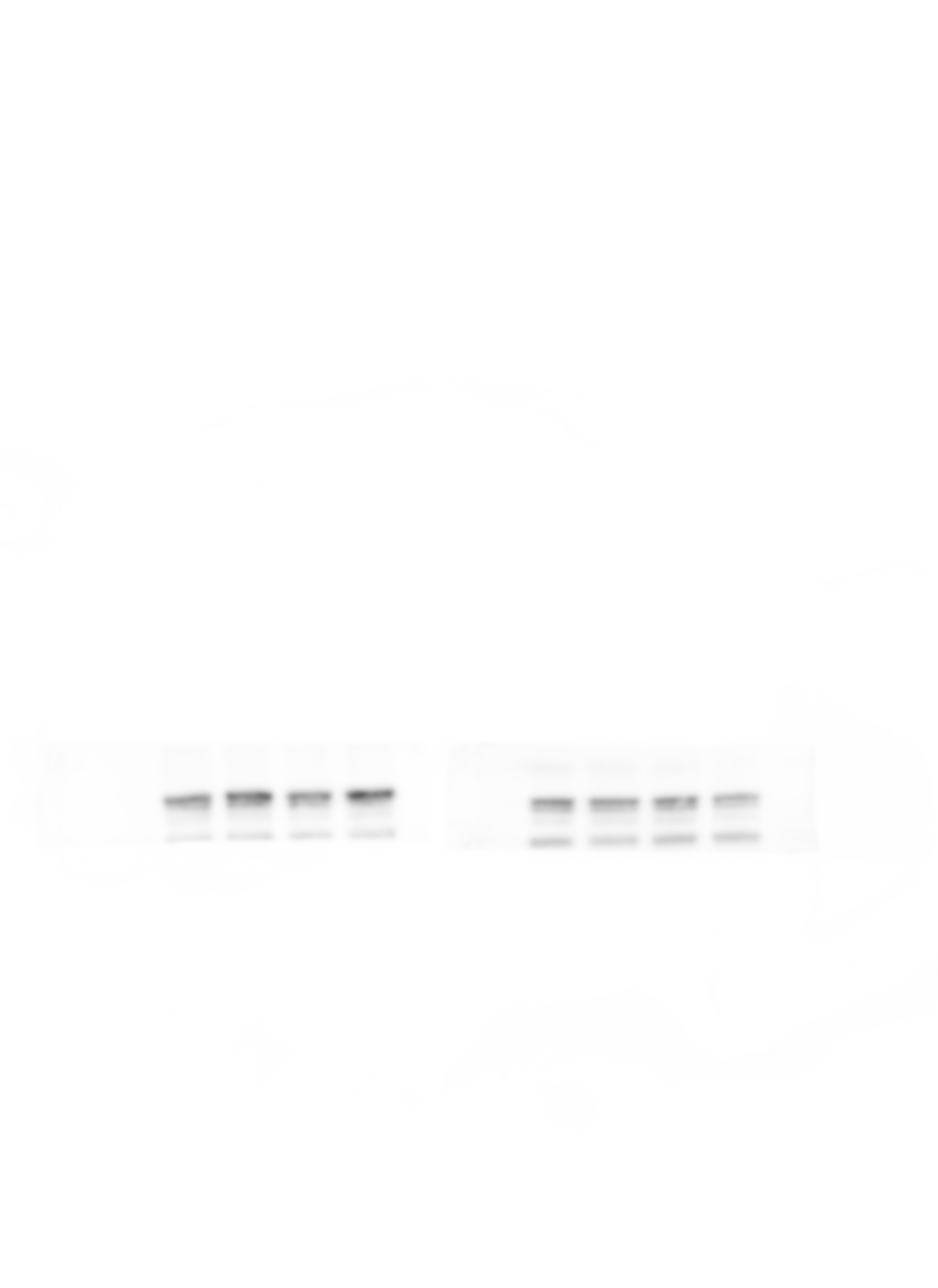

Supplement: Supplementary file 1 [file vetsci-12-01186-s001.zip › Supplementary Files/WB uncropped figure/Figure S1/eif2 20250330_102330_Ch/eif2 20250330_102330_Ch_Chemi.tif]

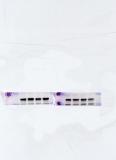

Supplement: Supplementary file 1 [file vetsci-12-01186-s001.zip › Supplementary Files/WB uncropped figure/Figure S1/eif2 20250330_102330_Ch/eif2 20250330_102330_Ch_Thumb.jpg]

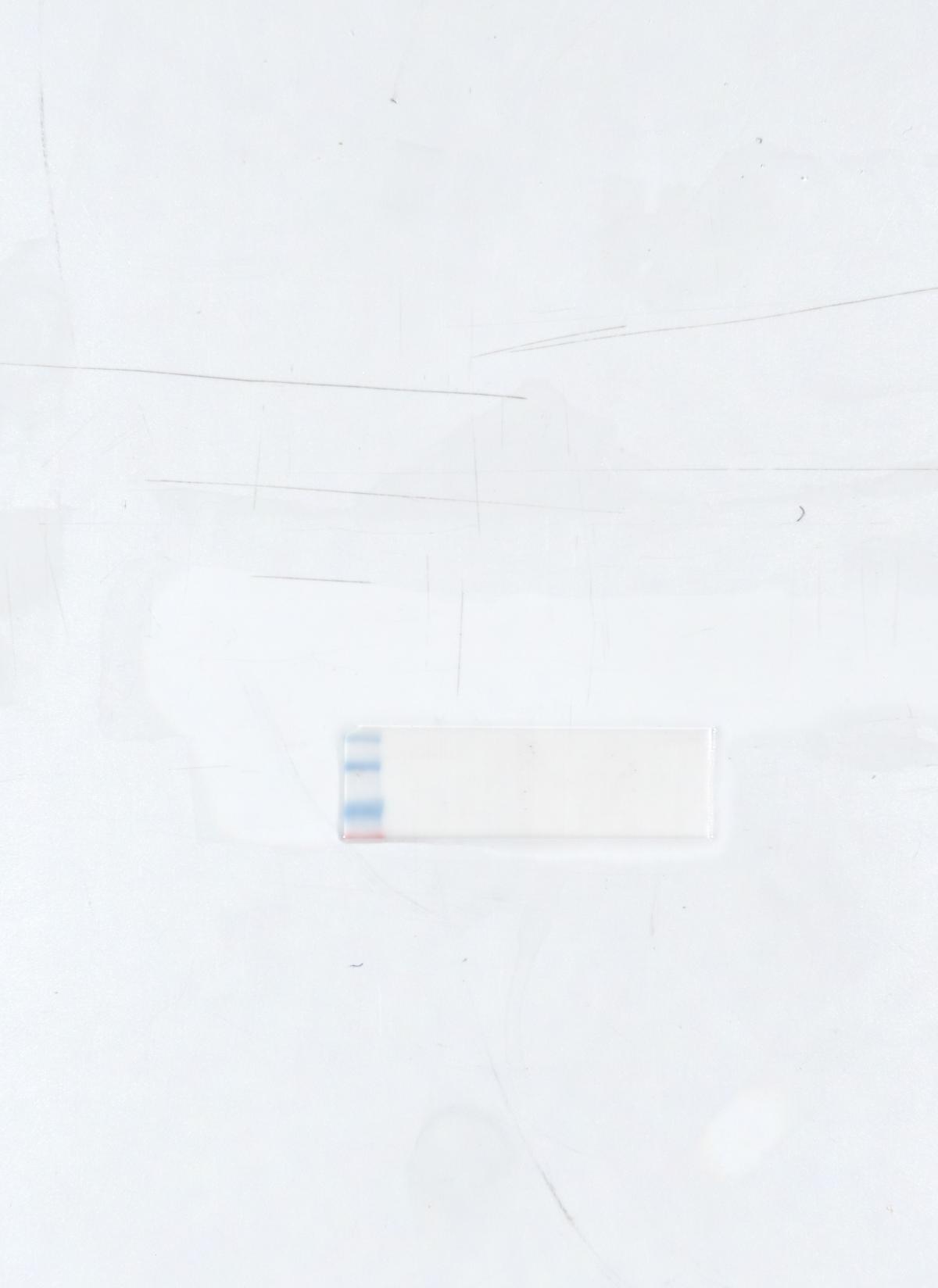

Supplement: Supplementary file 1 [file vetsci-12-01186-s001.zip › Supplementary Files/WB uncropped figure/Figure S1/gadd34 2 20250515_143354_Ch/gadd24 2 20250515_143354_Ch-Marker.jpg]

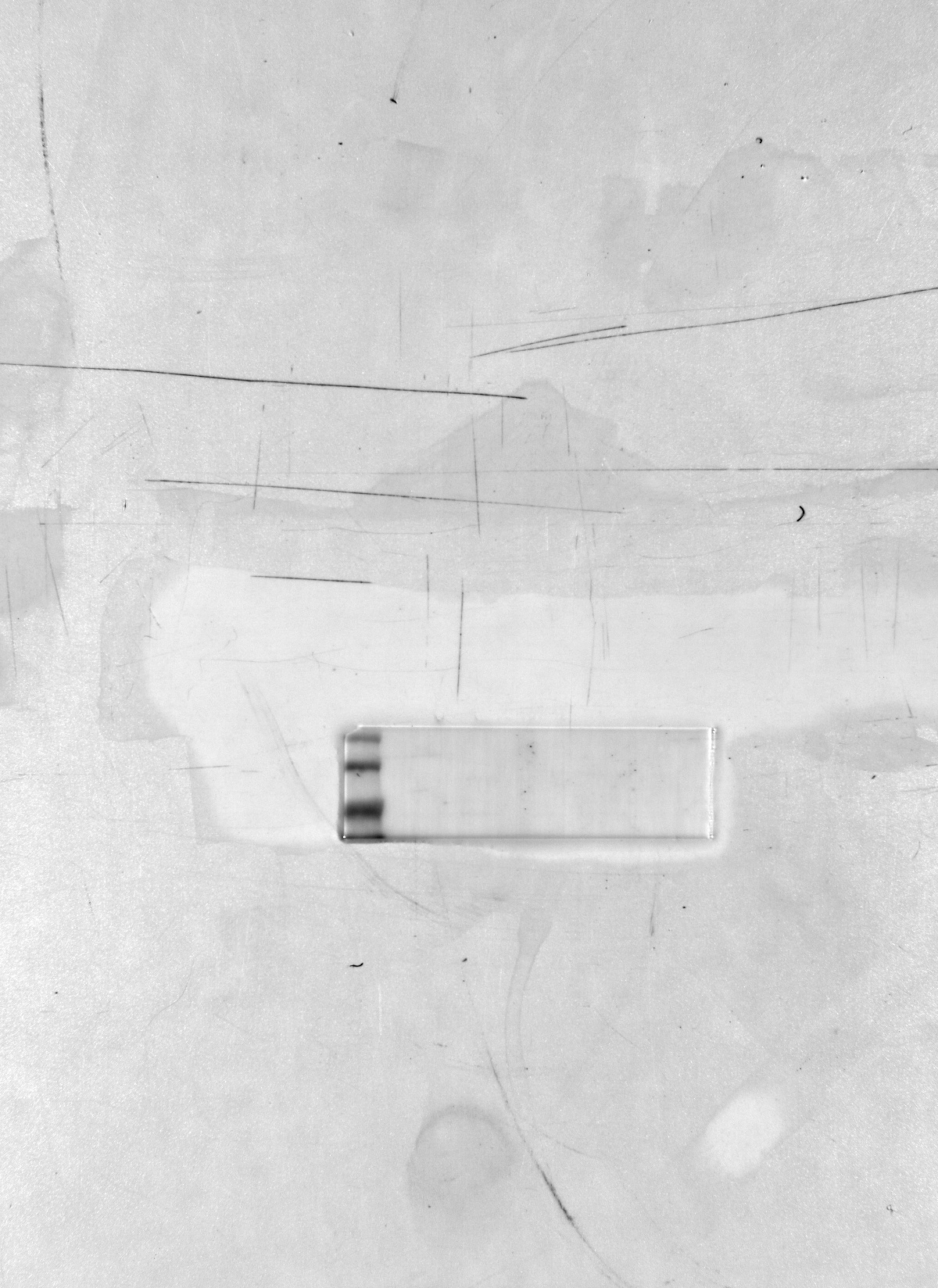

Supplement: Supplementary file 1 [file vetsci-12-01186-s001.zip › Supplementary Files/WB uncropped figure/Figure S1/gadd34 2 20250515_143354_Ch/gadd24 2 20250515_143354_Ch-Marker.tif]

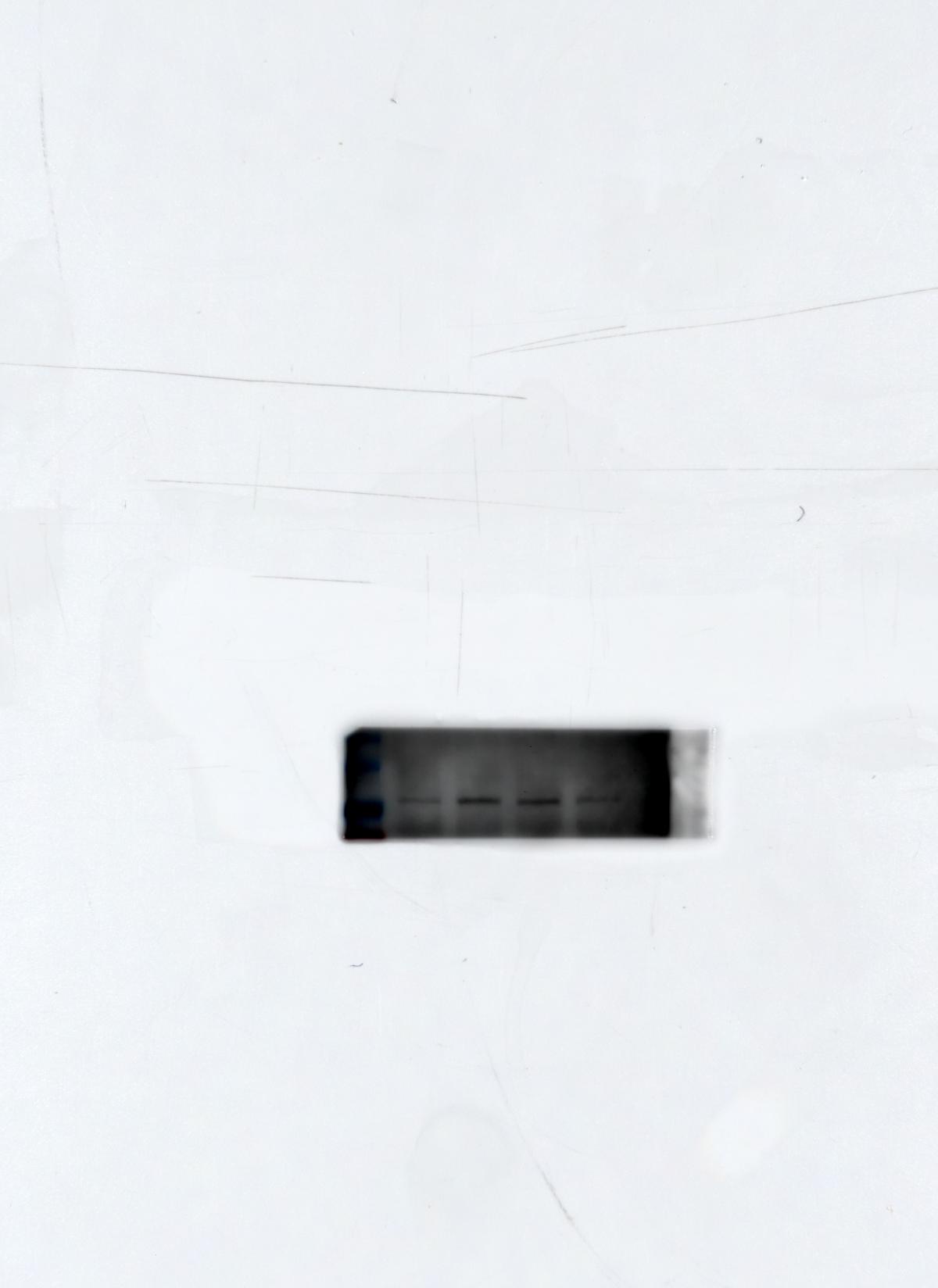

Supplement: Supplementary file 1 [file vetsci-12-01186-s001.zip › Supplementary Files/WB uncropped figure/Figure S1/gadd34 2 20250515_143354_Ch/gadd24 2 20250515_143354_Ch_Chemi+Marker.jpg]

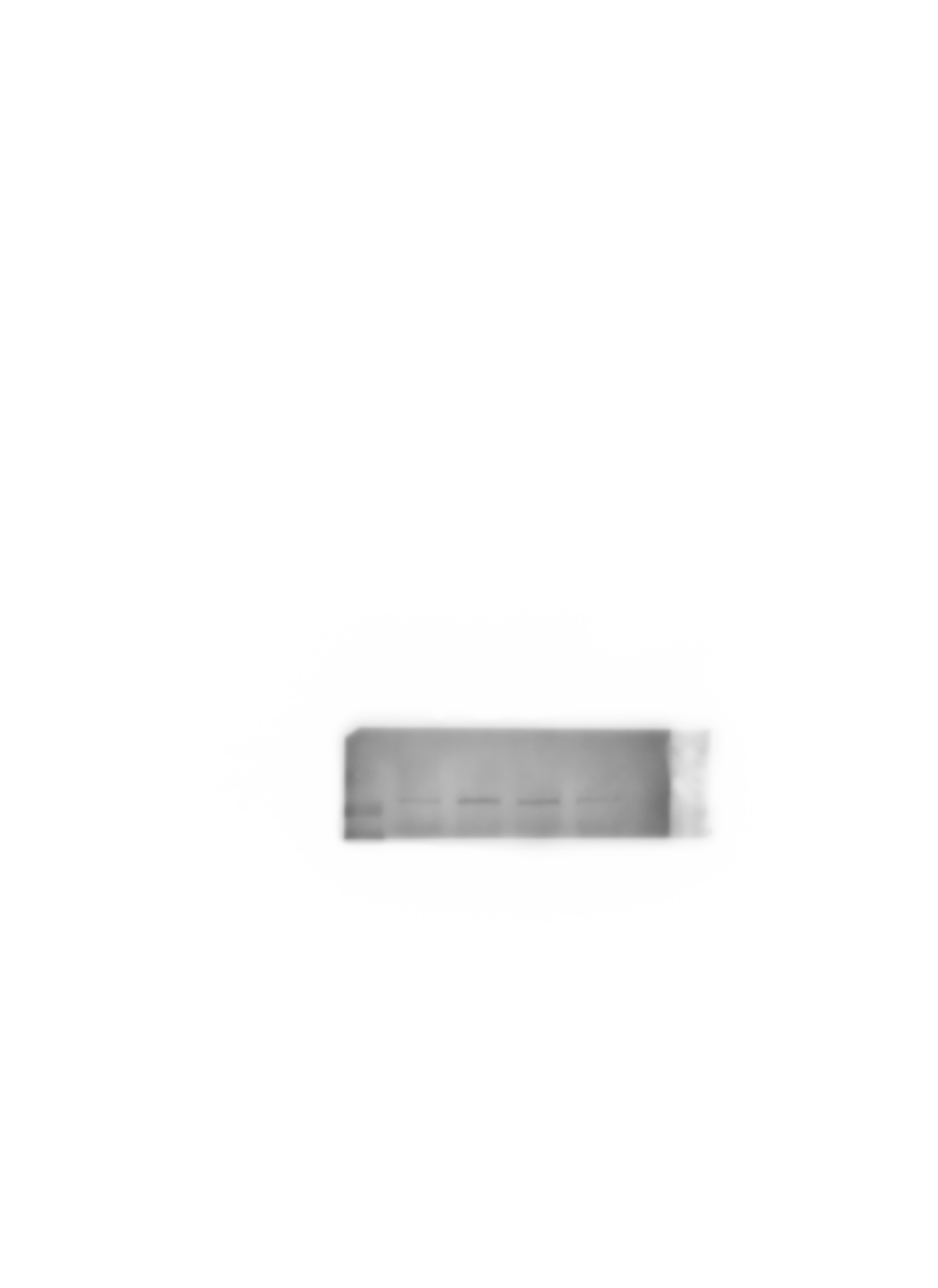

Supplement: Supplementary file 1 [file vetsci-12-01186-s001.zip › Supplementary Files/WB uncropped figure/Figure S1/gadd34 2 20250515_143354_Ch/gadd24 2 20250515_143354_Ch_Chemi.tif]

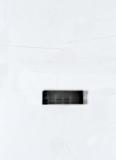

Supplement: Supplementary file 1 [file vetsci-12-01186-s001.zip › Supplementary Files/WB uncropped figure/Figure S1/gadd34 2 20250515_143354_Ch/gadd24 2 20250515_143354_Ch_Thumb.jpg]

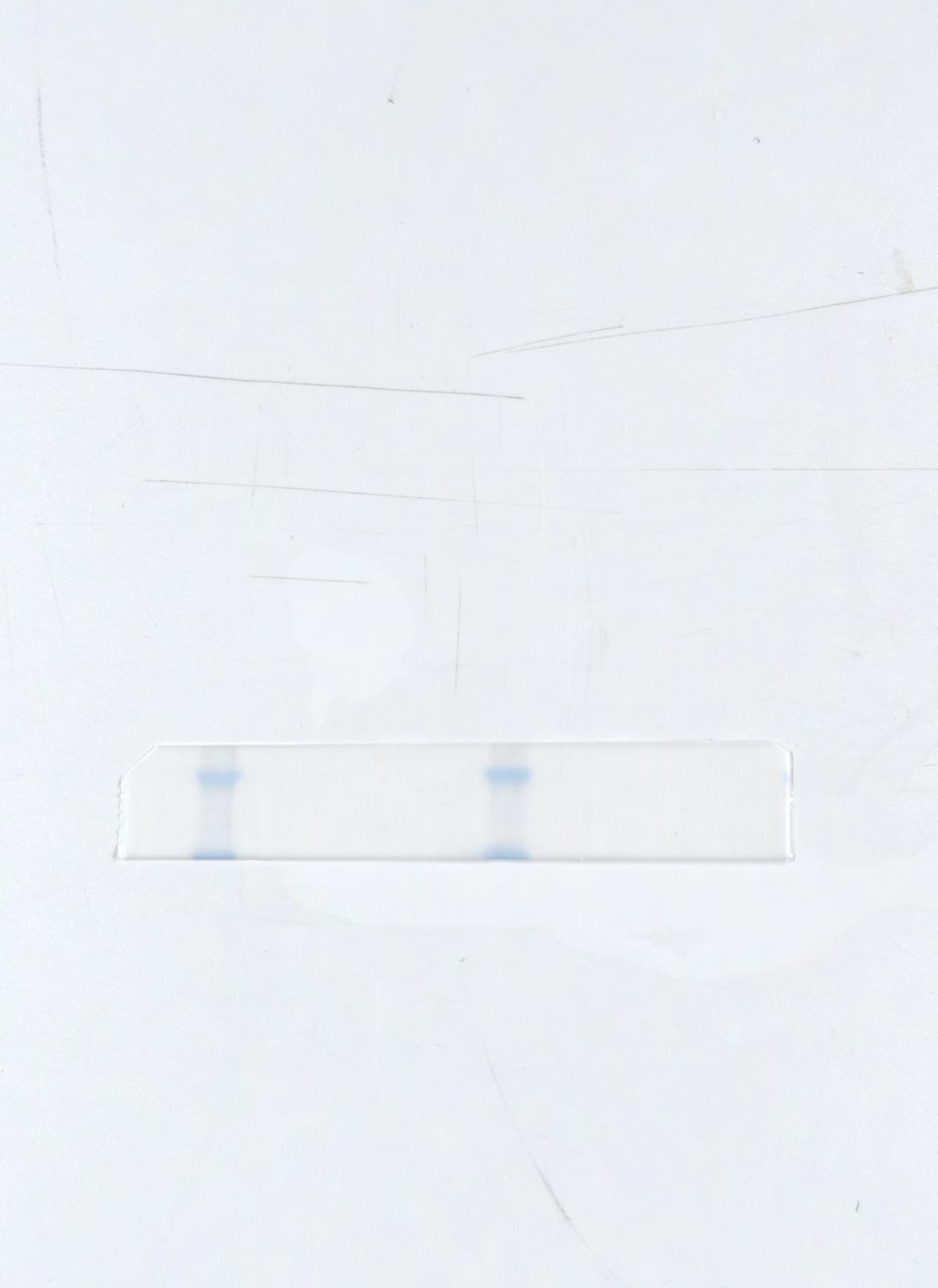

Supplement: Supplementary file 1 [file vetsci-12-01186-s001.zip › Supplementary Files/WB uncropped figure/Figure S1/GAPDH 11 20250502_174316_Ch/GAPDH 11 20250502_174316_Ch-Marker.jpg]

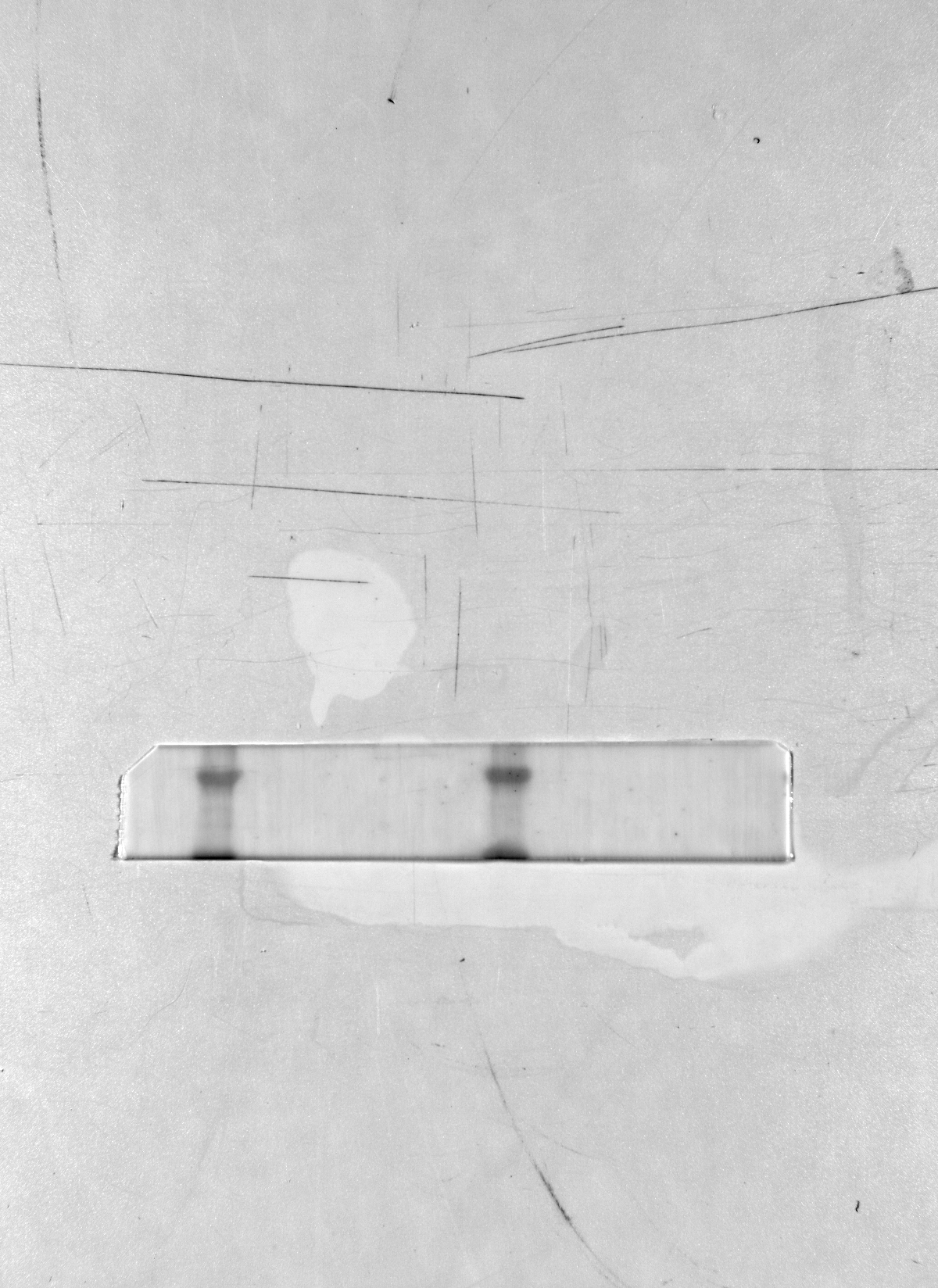

Supplement: Supplementary file 1 [file vetsci-12-01186-s001.zip › Supplementary Files/WB uncropped figure/Figure S1/GAPDH 11 20250502_174316_Ch/GAPDH 11 20250502_174316_Ch-Marker.tif]

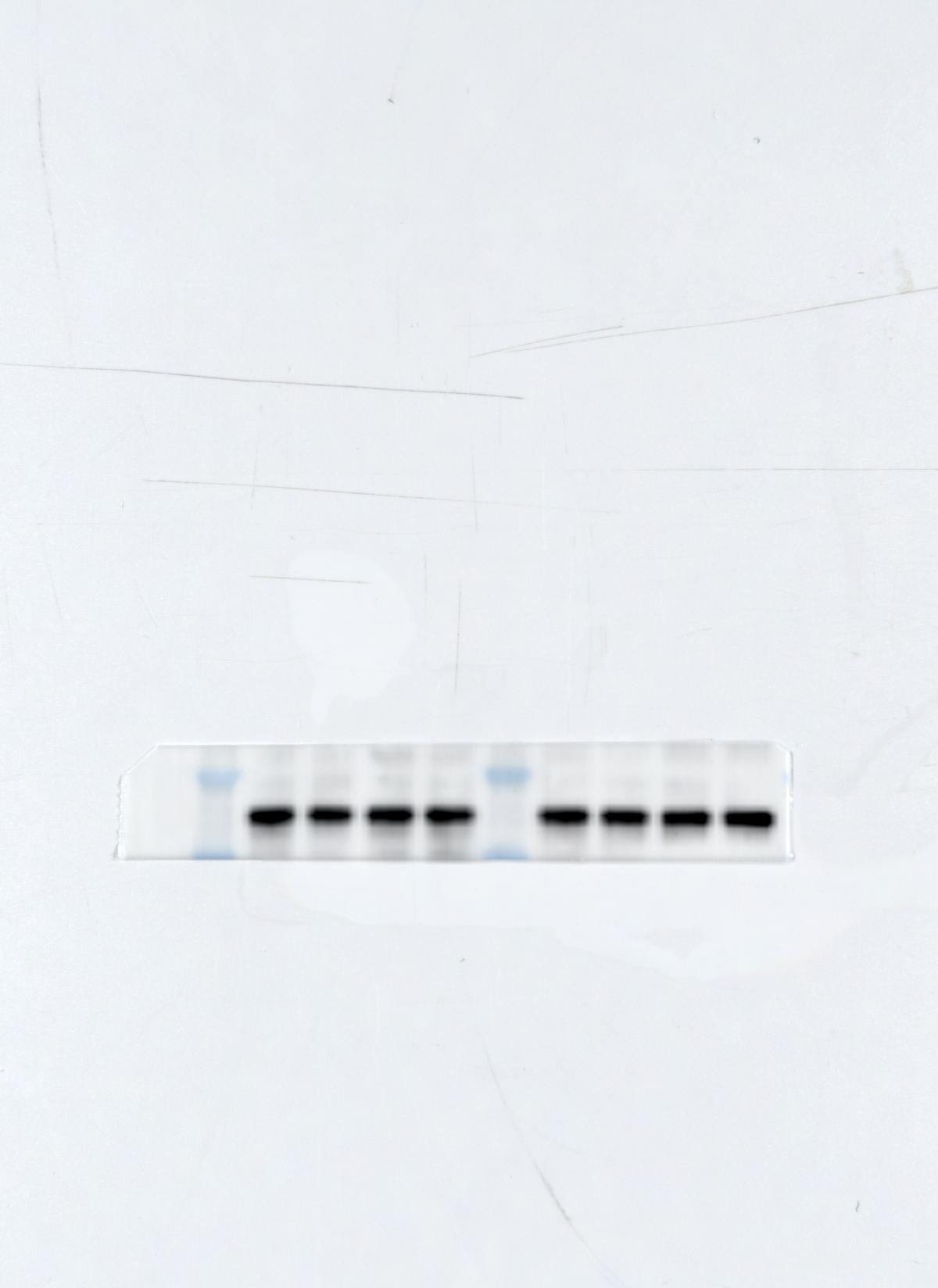

Supplement: Supplementary file 1 [file vetsci-12-01186-s001.zip › Supplementary Files/WB uncropped figure/Figure S1/GAPDH 11 20250502_174316_Ch/GAPDH 11 20250502_174316_Ch_Chemi+Marker.jpg]

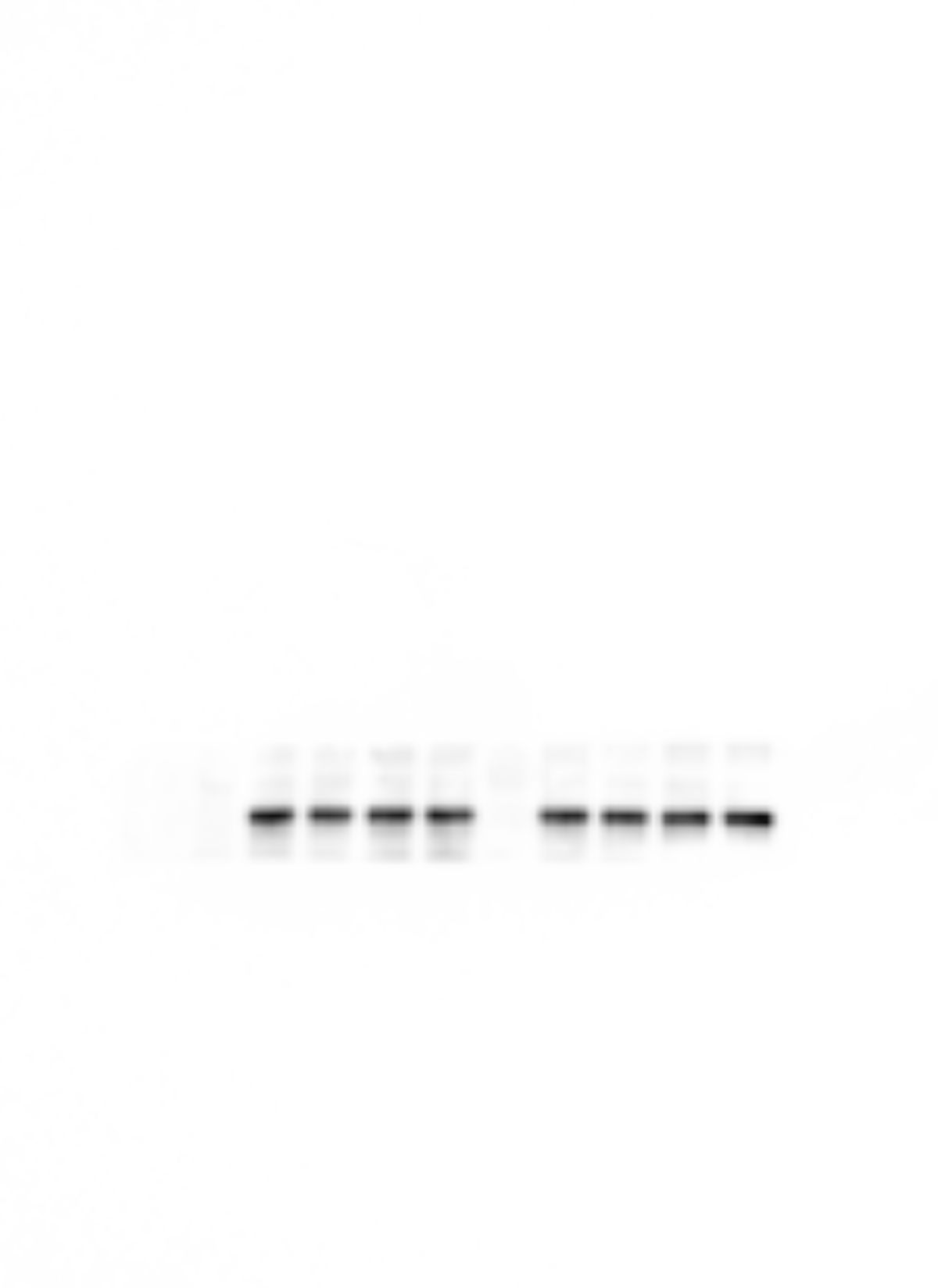

Supplement: Supplementary file 1 [file vetsci-12-01186-s001.zip › Supplementary Files/WB uncropped figure/Figure S1/GAPDH 11 20250502_174316_Ch/GAPDH 11 20250502_174316_Ch_Chemi.tif]

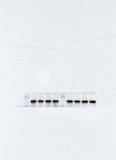

Supplement: Supplementary file 1 [file vetsci-12-01186-s001.zip › Supplementary Files/WB uncropped figure/Figure S1/GAPDH 11 20250502_174316_Ch/GAPDH 11 20250502_174316_Ch_Thumb.jpg]

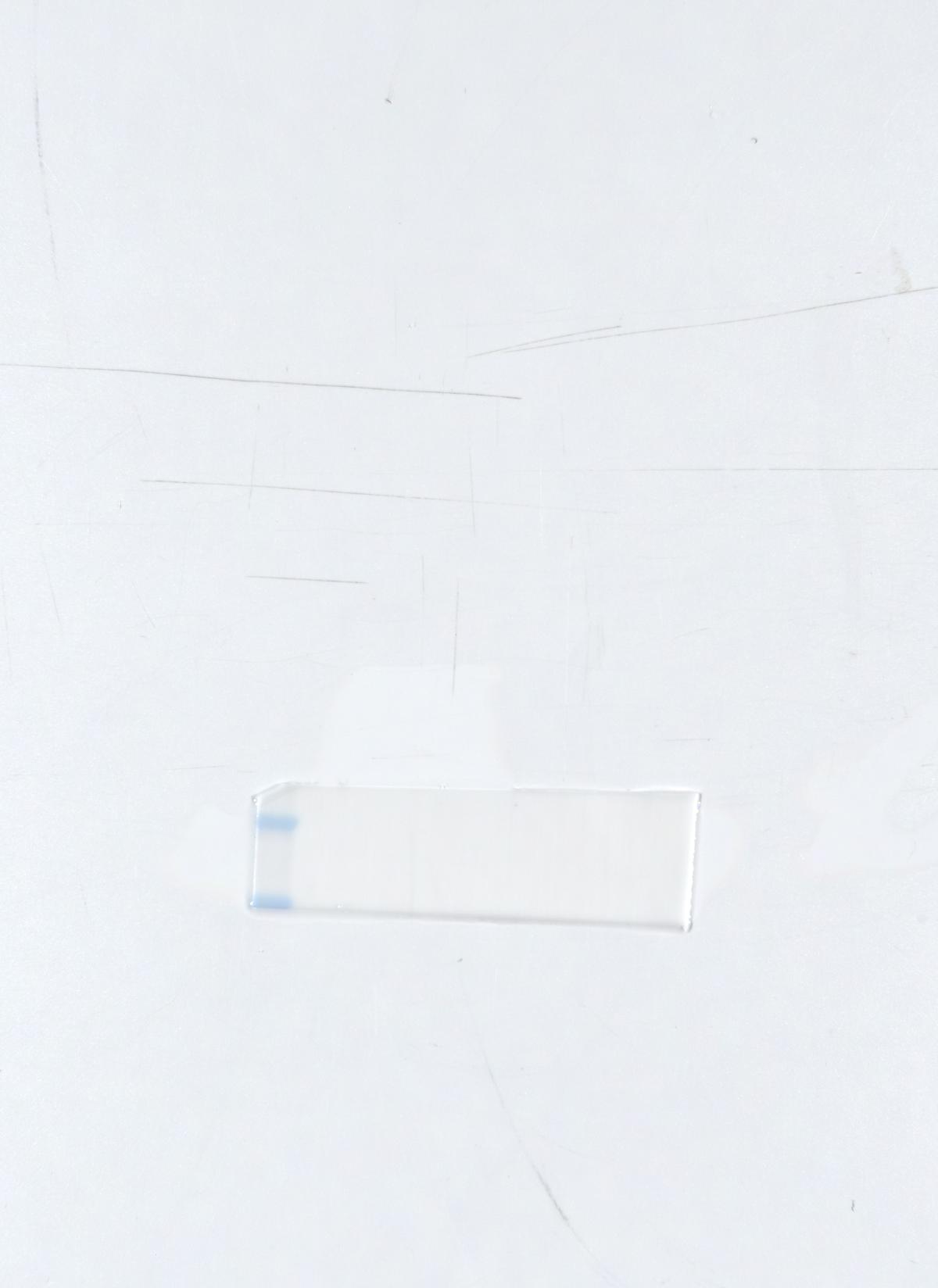

Supplement: Supplementary file 1 [file vetsci-12-01186-s001.zip › Supplementary Files/WB uncropped figure/Figure S1/GAPDH 2 20250502_174115_Ch/GAPDH 2 20250502_174115_Ch-Marker.jpg]

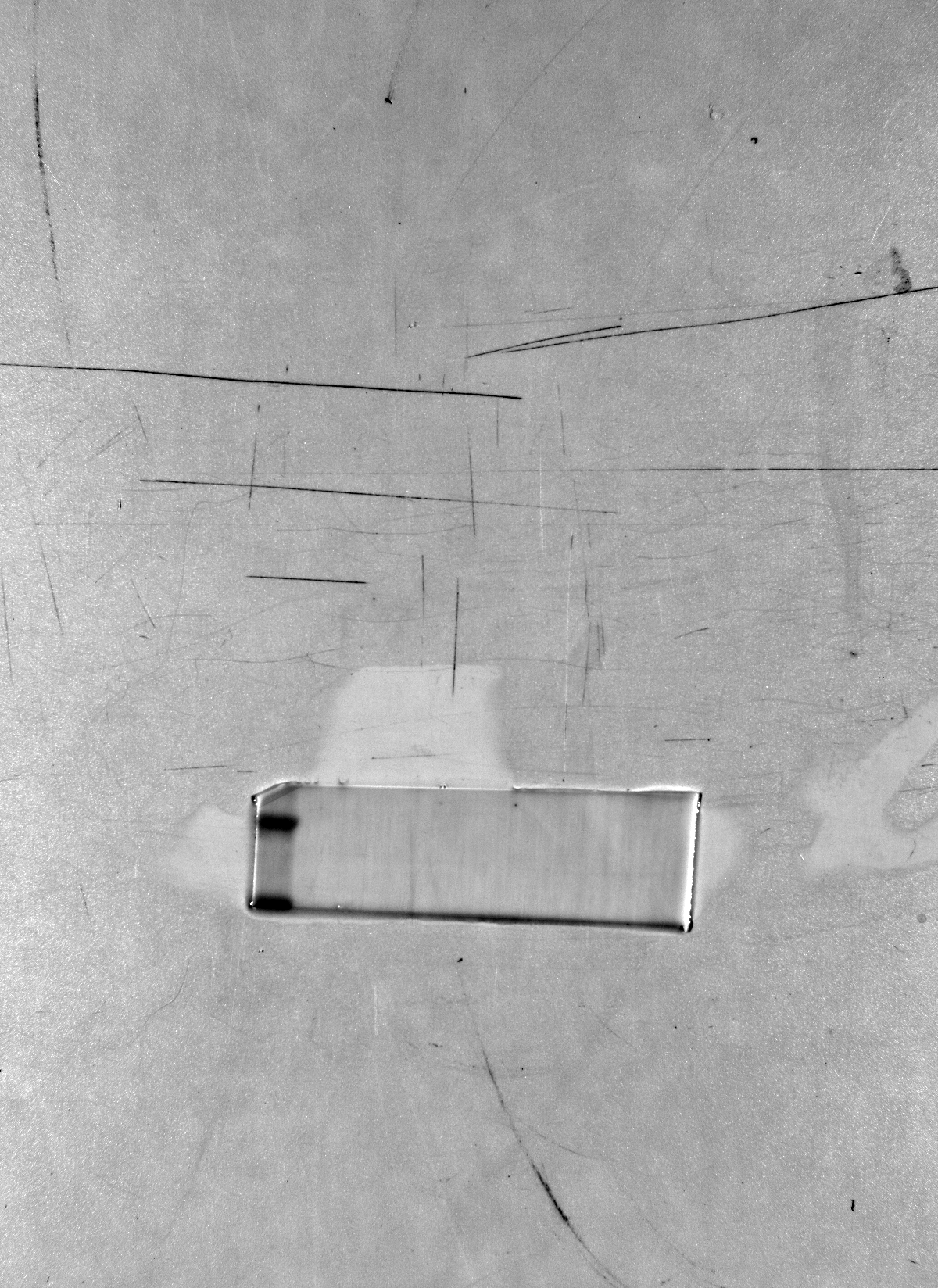

Supplement: Supplementary file 1 [file vetsci-12-01186-s001.zip › Supplementary Files/WB uncropped figure/Figure S1/GAPDH 2 20250502_174115_Ch/GAPDH 2 20250502_174115_Ch-Marker.tif]

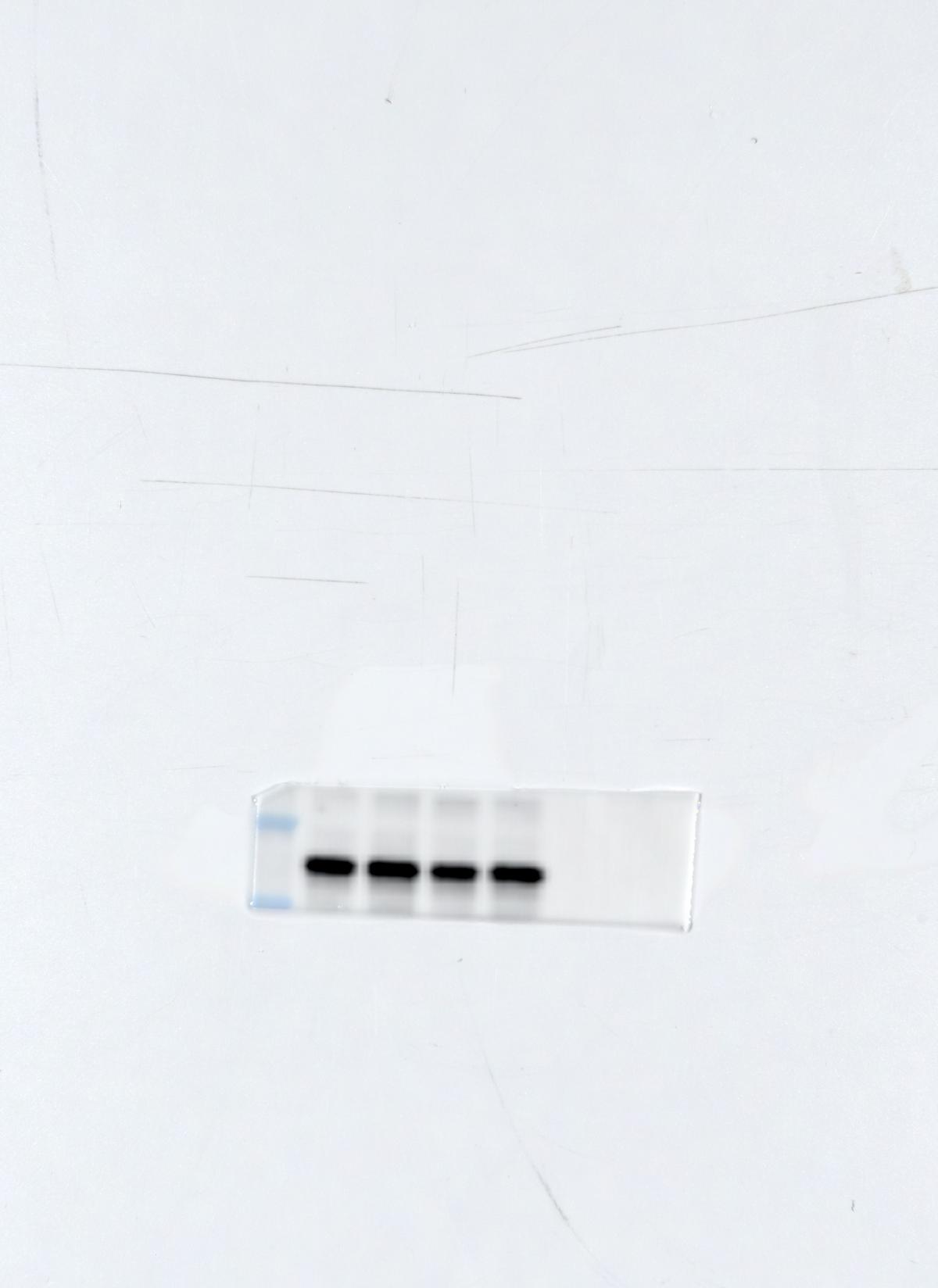

Supplement: Supplementary file 1 [file vetsci-12-01186-s001.zip › Supplementary Files/WB uncropped figure/Figure S1/GAPDH 2 20250502_174115_Ch/GAPDH 2 20250502_174115_Ch_Chemi+Marker.jpg]

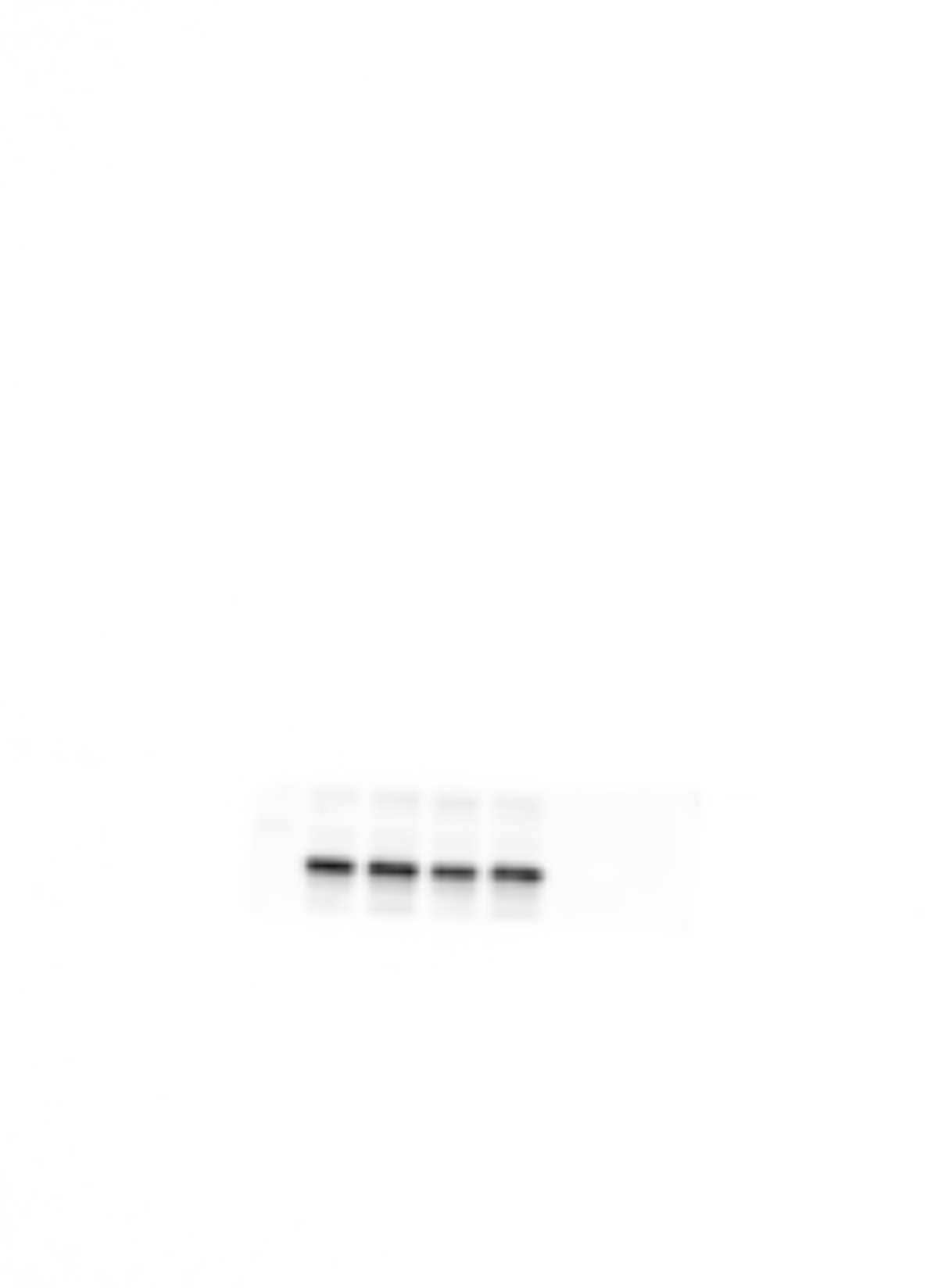

Supplement: Supplementary file 1 [file vetsci-12-01186-s001.zip › Supplementary Files/WB uncropped figure/Figure S1/GAPDH 2 20250502_174115_Ch/GAPDH 2 20250502_174115_Ch_Chemi.tif]

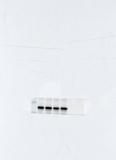

Supplement: Supplementary file 1 [file vetsci-12-01186-s001.zip › Supplementary Files/WB uncropped figure/Figure S1/GAPDH 2 20250502_174115_Ch/GAPDH 2 20250502_174115_Ch_Thumb.jpg]

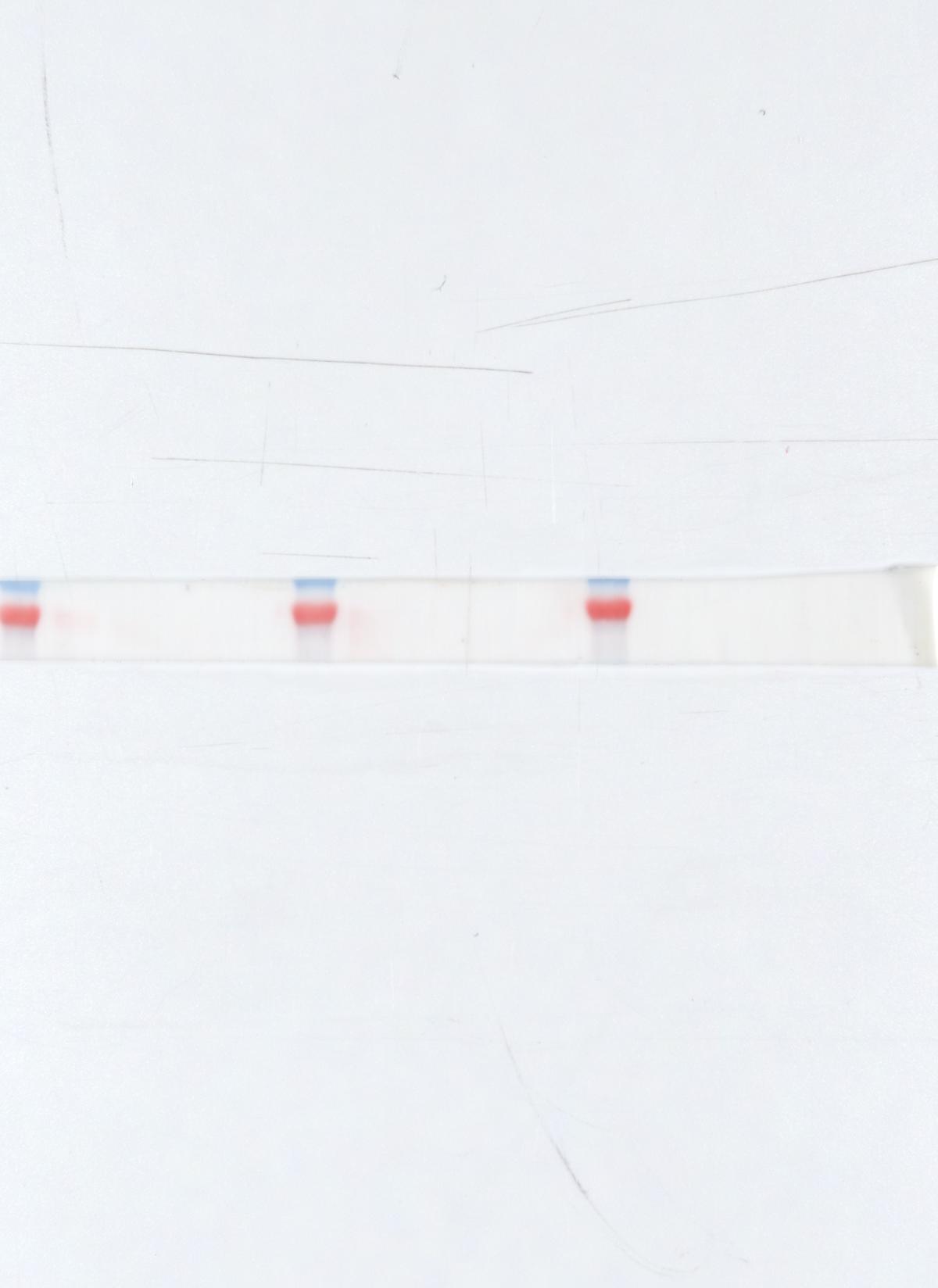

Supplement: Supplementary file 1 [file vetsci-12-01186-s001.zip › Supplementary Files/WB uncropped figure/Figure S1/grp78222 20250401_131719_Ch/grp78222 20250401_131719_Ch-Marker.jpg]

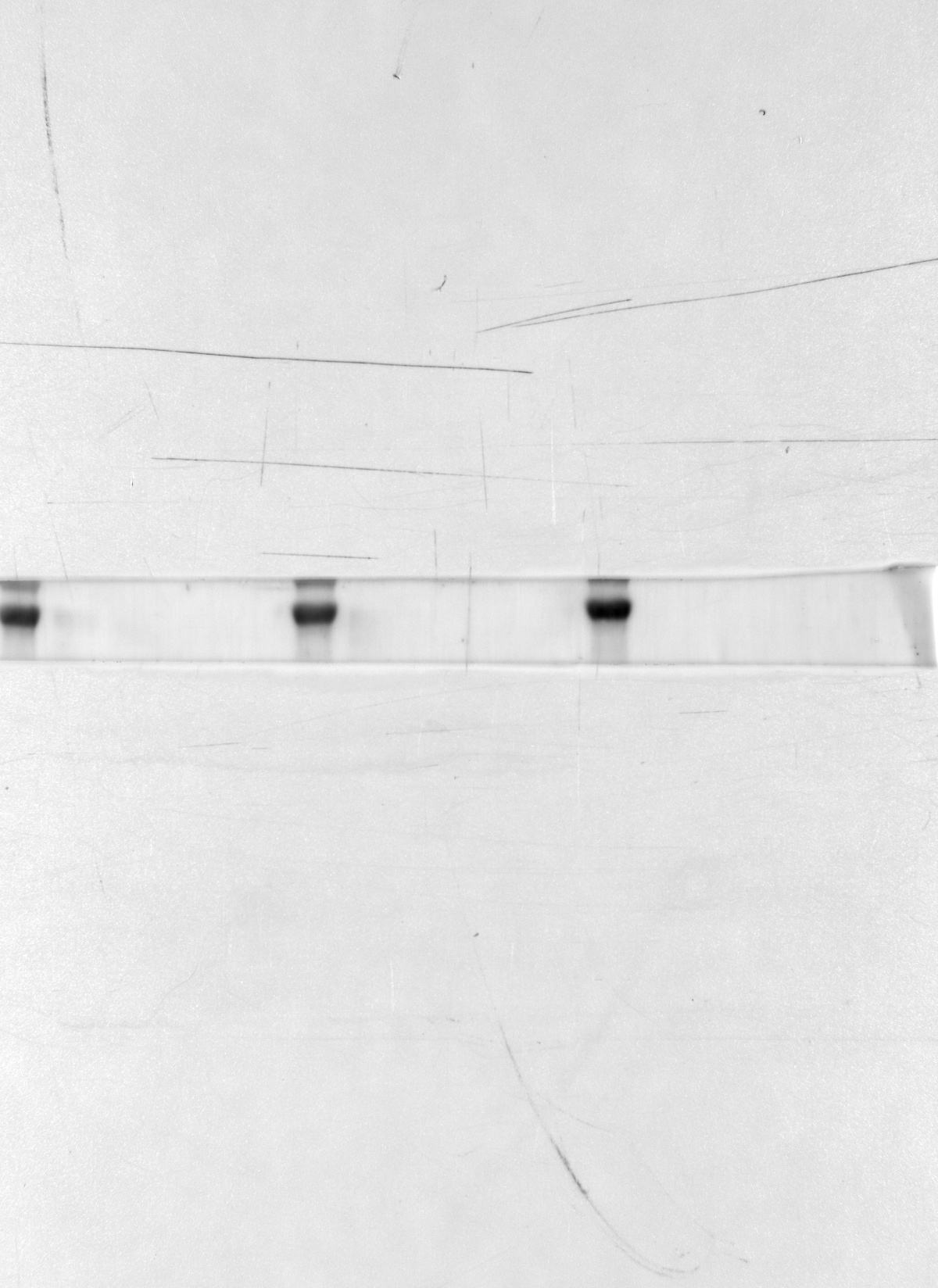

Supplement: Supplementary file 1 [file vetsci-12-01186-s001.zip › Supplementary Files/WB uncropped figure/Figure S1/grp78222 20250401_131719_Ch/grp78222 20250401_131719_Ch-Marker.tif]

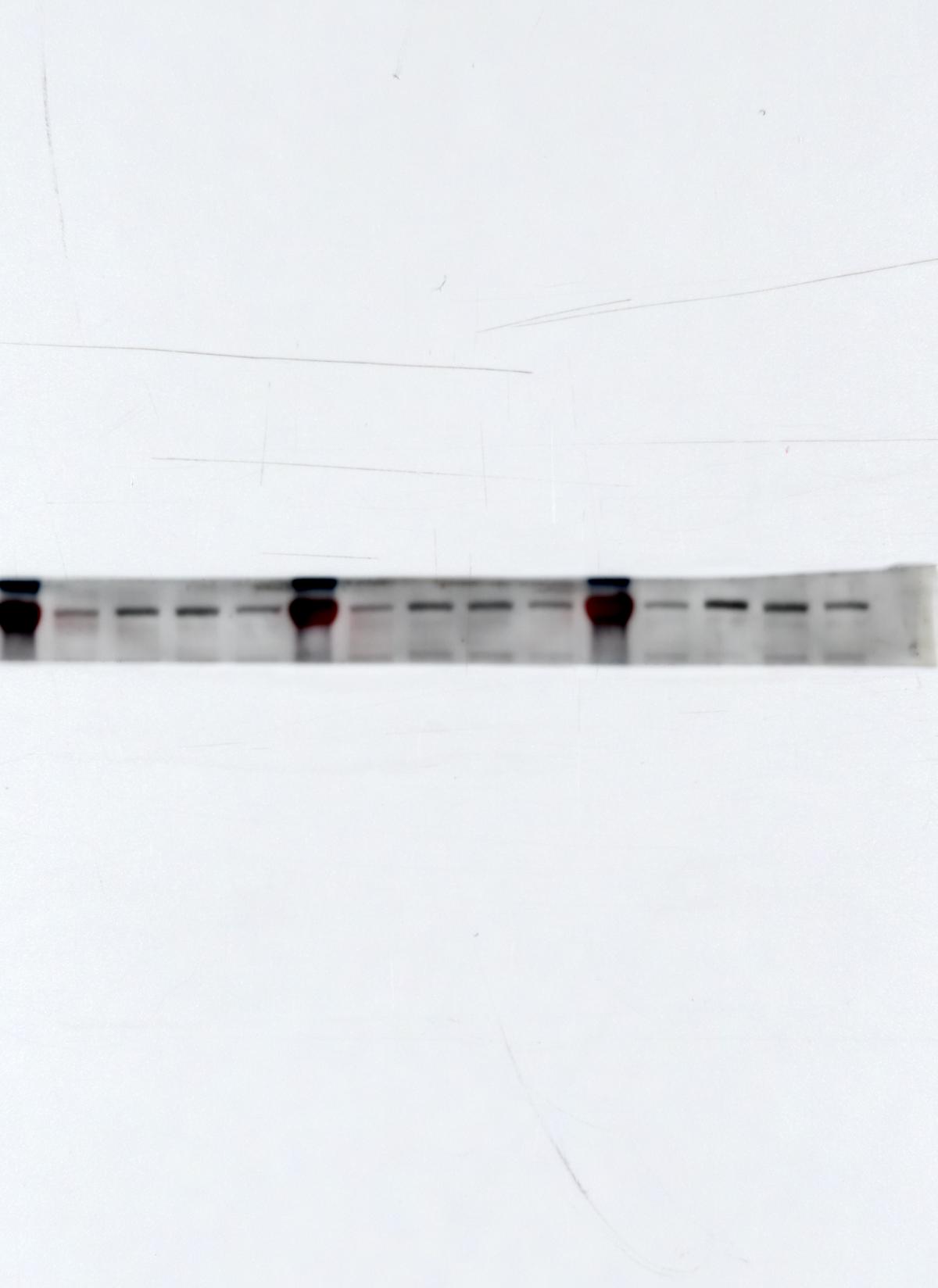

Supplement: Supplementary file 1 [file vetsci-12-01186-s001.zip › Supplementary Files/WB uncropped figure/Figure S1/grp78222 20250401_131719_Ch/grp78222 20250401_131719_Ch_Chemi+Marker.jpg]

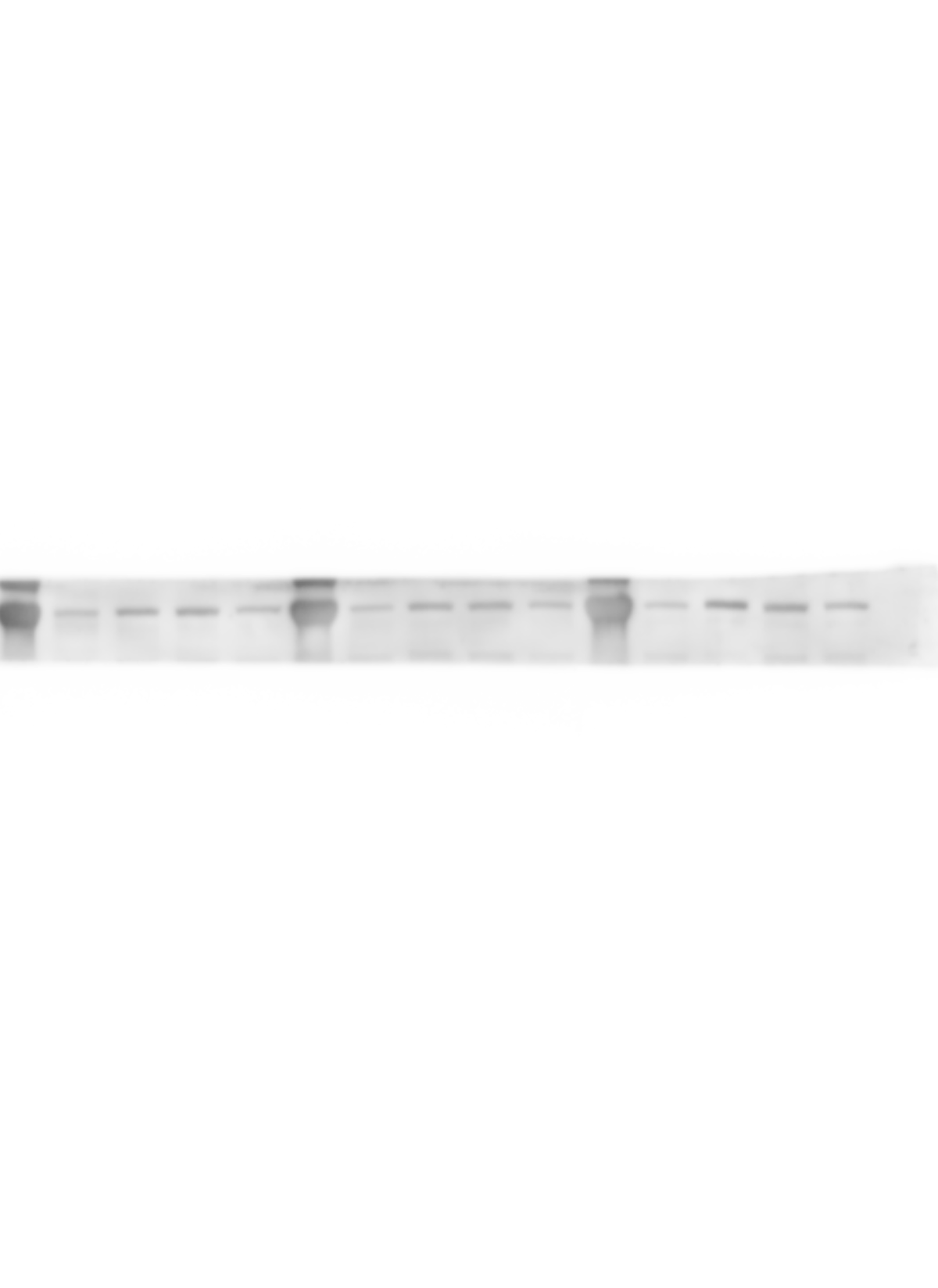

Supplement: Supplementary file 1 [file vetsci-12-01186-s001.zip › Supplementary Files/WB uncropped figure/Figure S1/grp78222 20250401_131719_Ch/grp78222 20250401_131719_Ch_Chemi.tif]

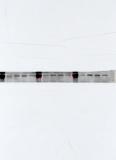

Supplement: Supplementary file 1 [file vetsci-12-01186-s001.zip › Supplementary Files/WB uncropped figure/Figure S1/grp78222 20250401_131719_Ch/grp78222 20250401_131719_Ch_Thumb.jpg]

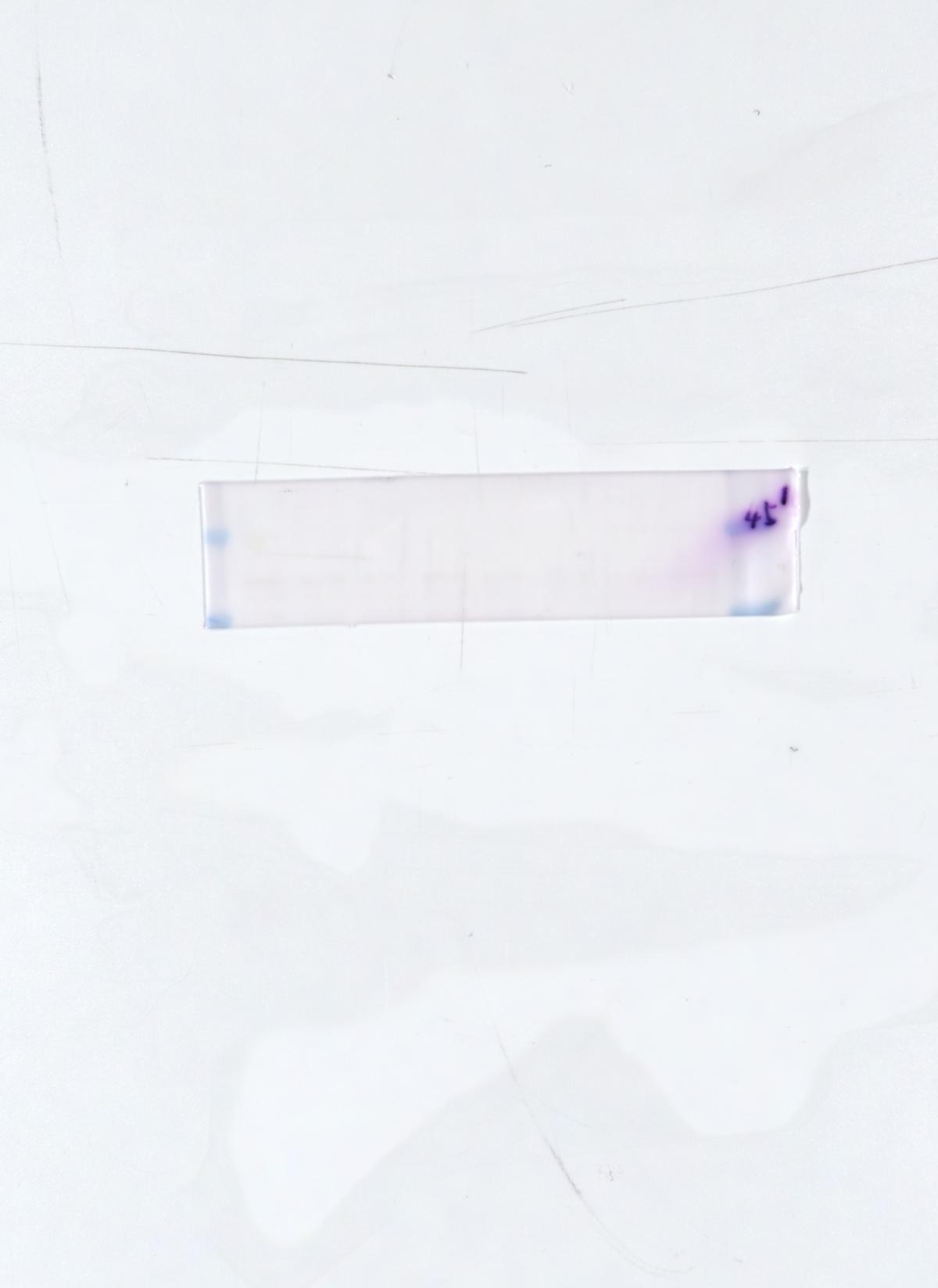

Supplement: Supplementary file 1 [file vetsci-12-01186-s001.zip › Supplementary Files/WB uncropped figure/Figure S1/p-eif2 20250330_101548_Ch/p-eif2 20250330_101548_Ch-Marker.jpg]

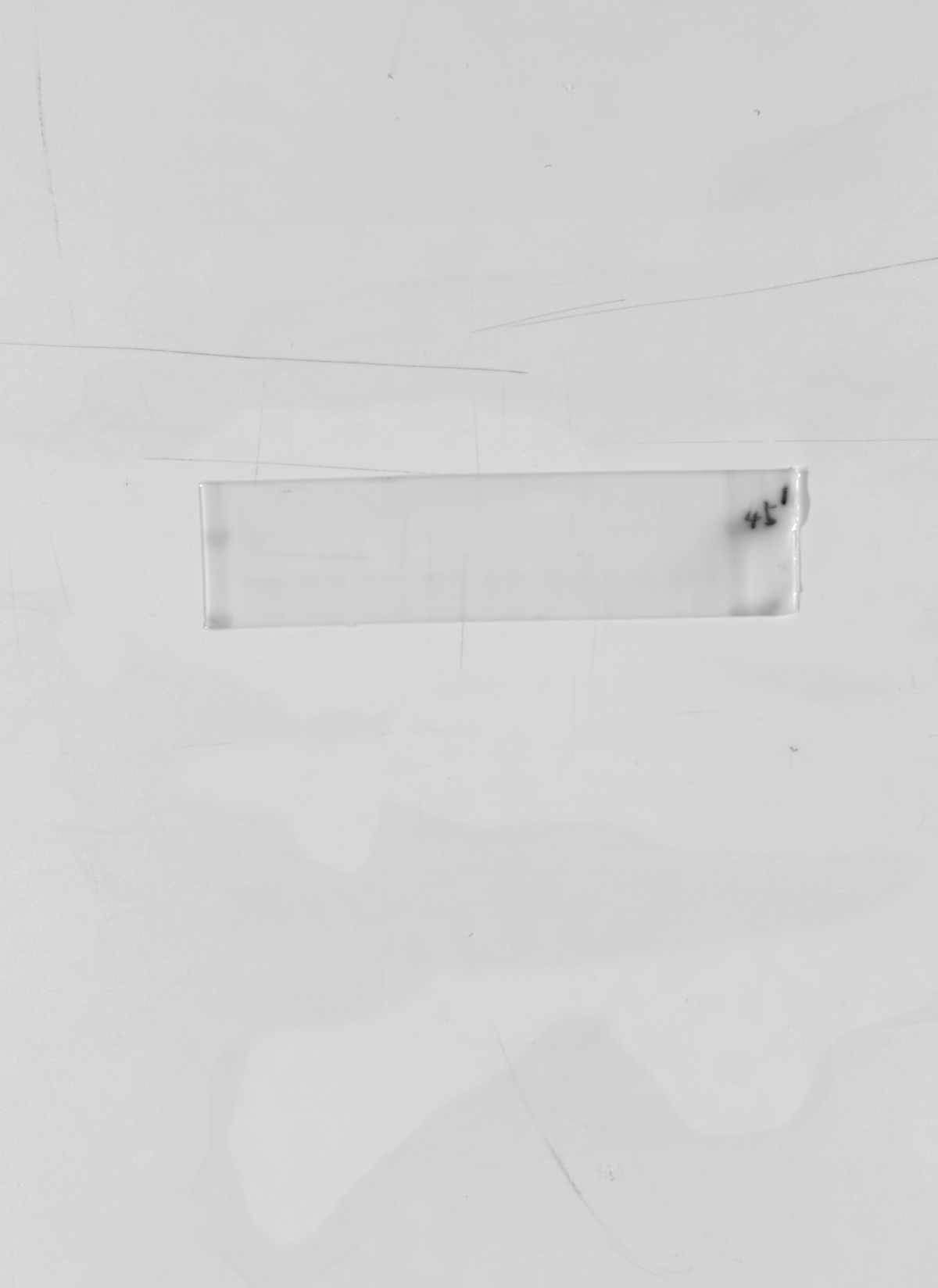

Supplement: Supplementary file 1 [file vetsci-12-01186-s001.zip › Supplementary Files/WB uncropped figure/Figure S1/p-eif2 20250330_101548_Ch/p-eif2 20250330_101548_Ch-Marker.tif]

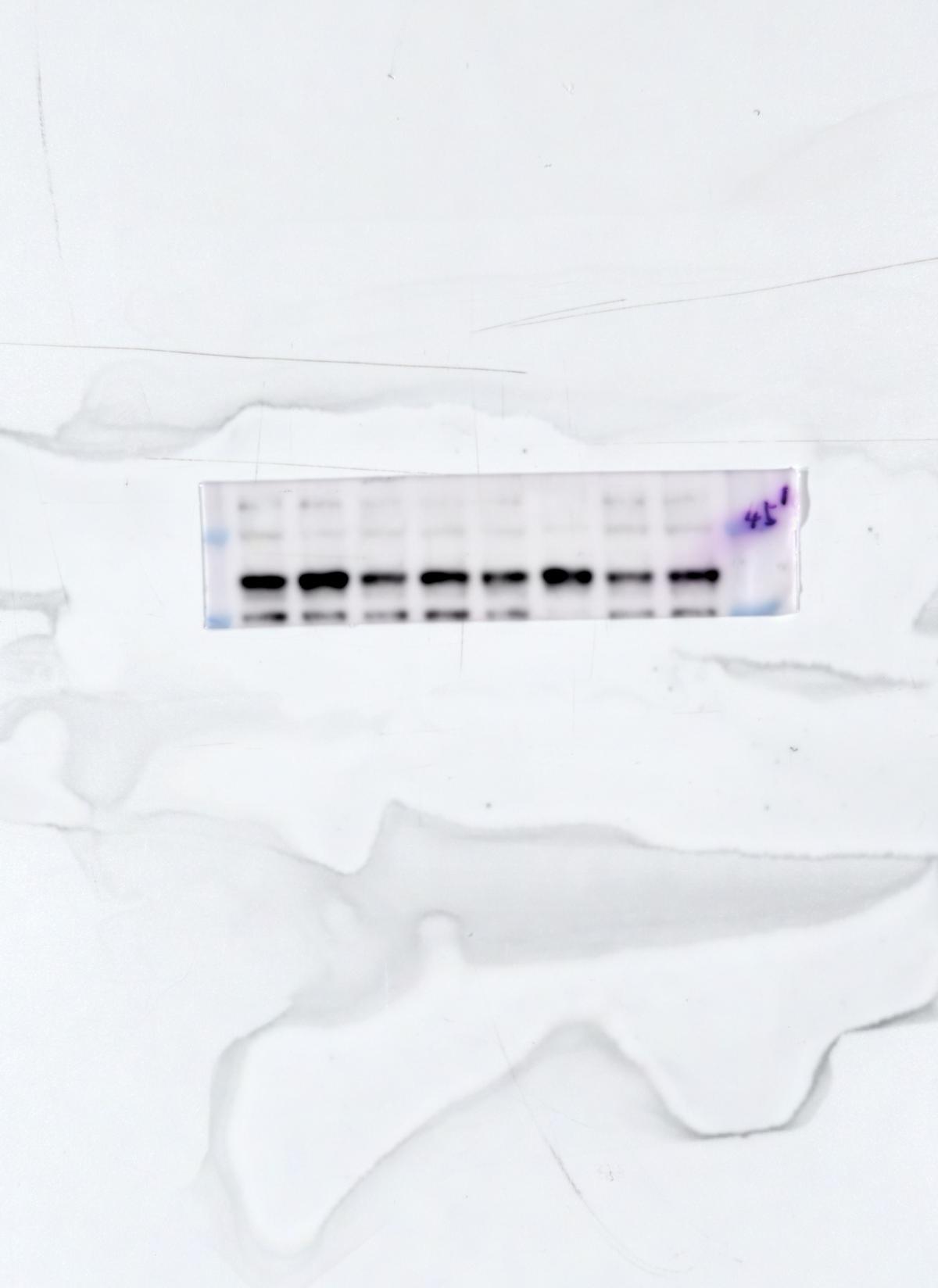

Supplement: Supplementary file 1 [file vetsci-12-01186-s001.zip › Supplementary Files/WB uncropped figure/Figure S1/p-eif2 20250330_101548_Ch/p-eif2 20250330_101548_Ch_Chemi+Marker.jpg]

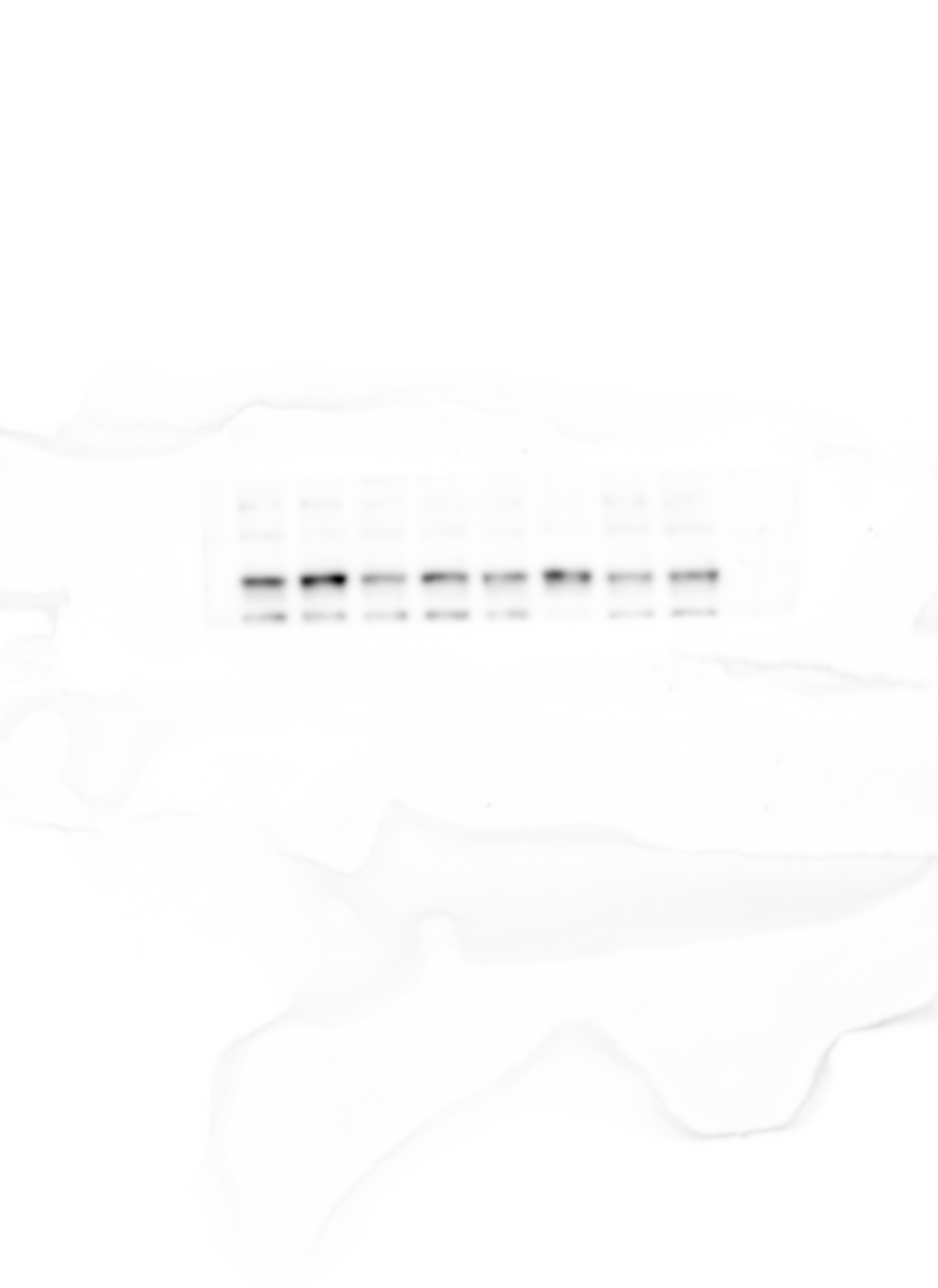

Supplement: Supplementary file 1 [file vetsci-12-01186-s001.zip › Supplementary Files/WB uncropped figure/Figure S1/p-eif2 20250330_101548_Ch/p-eif2 20250330_101548_Ch_Chemi.tif]

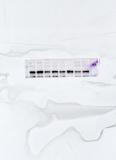

Supplement: Supplementary file 1 [file vetsci-12-01186-s001.zip › Supplementary Files/WB uncropped figure/Figure S1/p-eif2 20250330_101548_Ch/p-eif2 20250330_101548_Ch_Thumb.jpg]

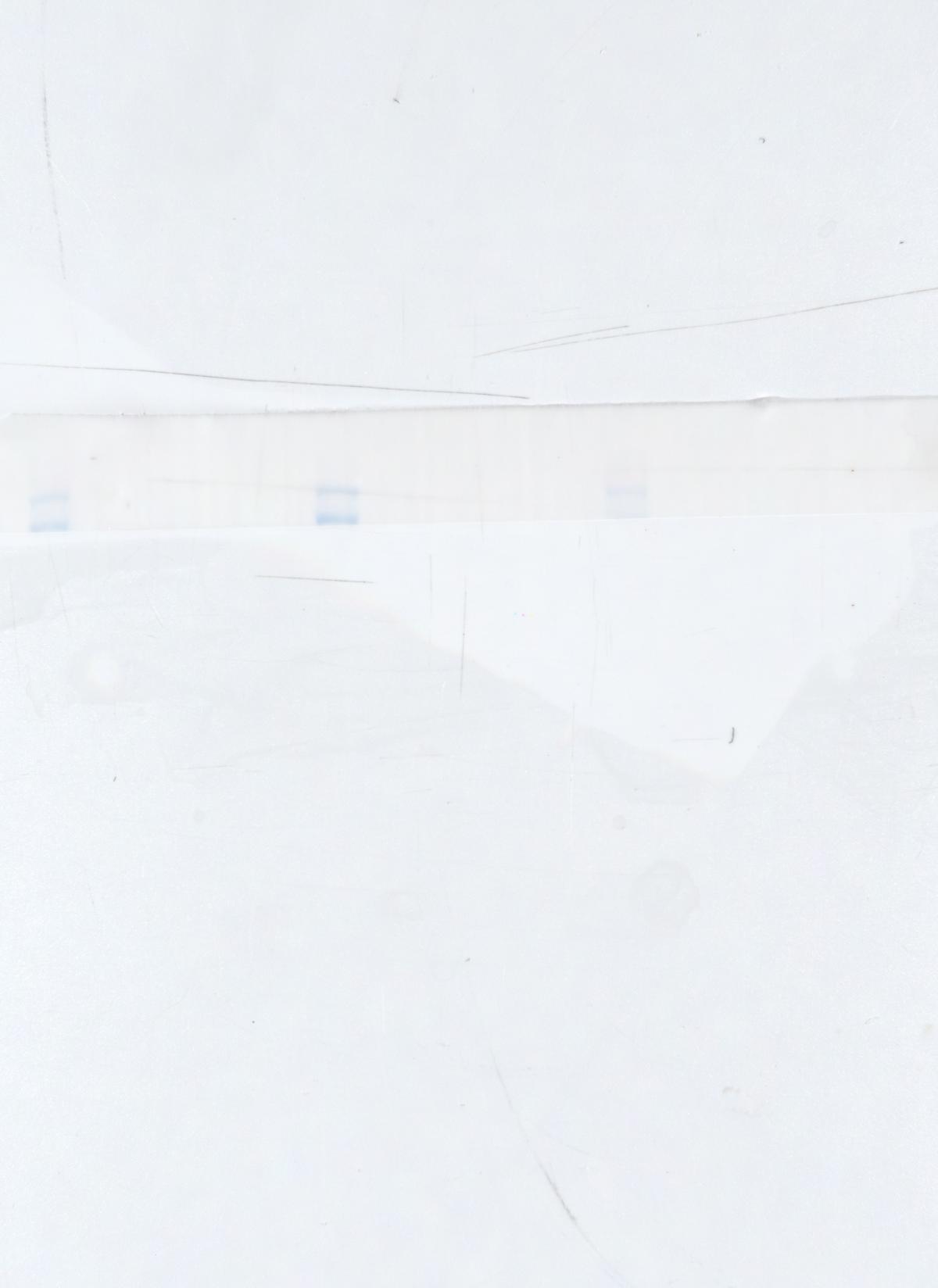

Supplement: Supplementary file 1 [file vetsci-12-01186-s001.zip › Supplementary Files/WB uncropped figure/Figure S1/p-PERK 20250429_135744_Ch/p-PERK 20250429_135744_Ch-Marker.jpg]

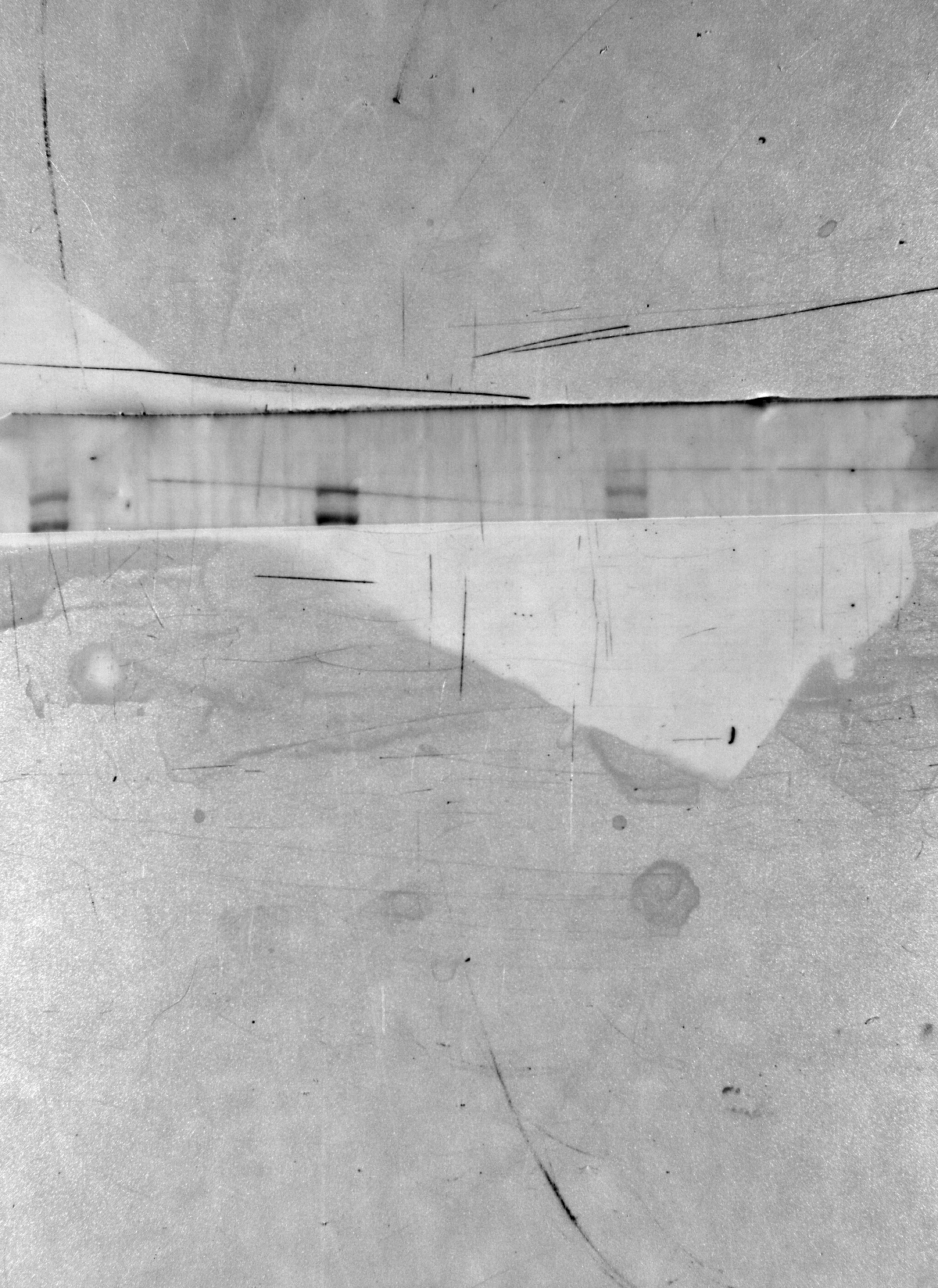

Supplement: Supplementary file 1 [file vetsci-12-01186-s001.zip › Supplementary Files/WB uncropped figure/Figure S1/p-PERK 20250429_135744_Ch/p-PERK 20250429_135744_Ch-Marker.tif]

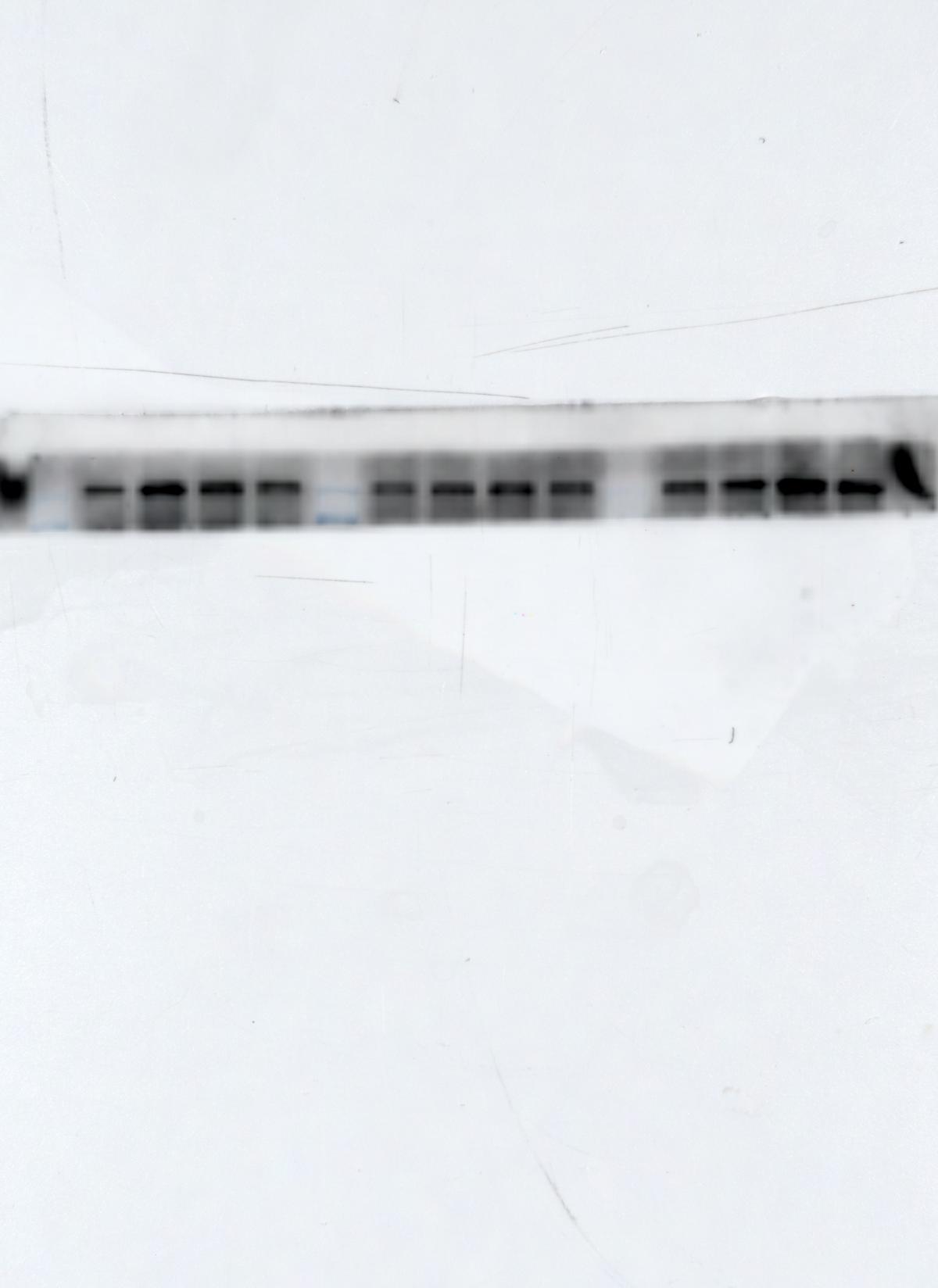

Supplement: Supplementary file 1 [file vetsci-12-01186-s001.zip › Supplementary Files/WB uncropped figure/Figure S1/p-PERK 20250429_135744_Ch/p-PERK 20250429_135744_Ch_Chemi+Marker.jpg]

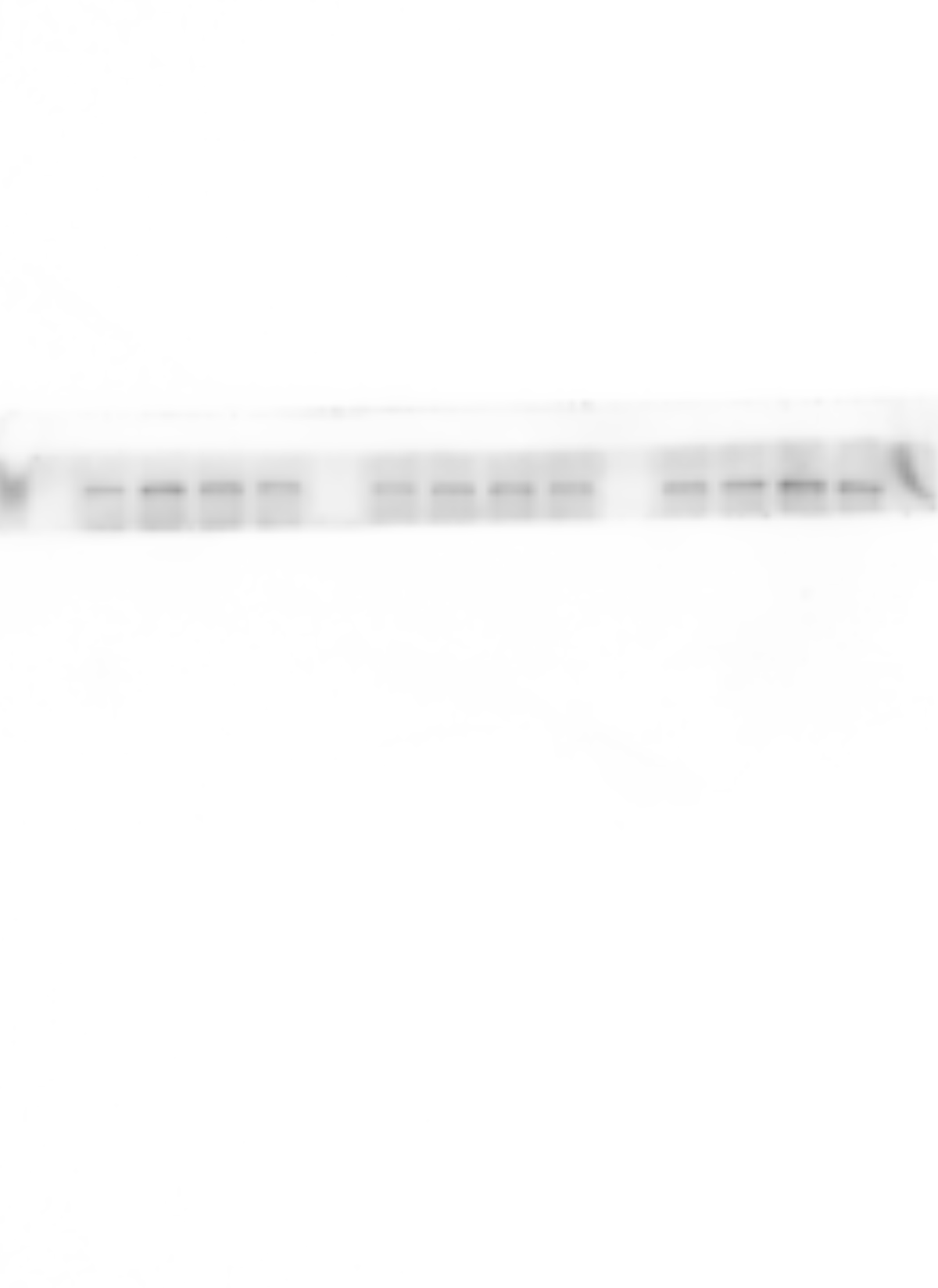

Supplement: Supplementary file 1 [file vetsci-12-01186-s001.zip › Supplementary Files/WB uncropped figure/Figure S1/p-PERK 20250429_135744_Ch/p-PERK 20250429_135744_Ch_Chemi.tif]

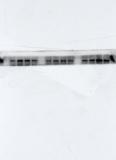

Supplement: Supplementary file 1 [file vetsci-12-01186-s001.zip › Supplementary Files/WB uncropped figure/Figure S1/p-PERK 20250429_135744_Ch/p-PERK 20250429_135744_Ch_Thumb.jpg]

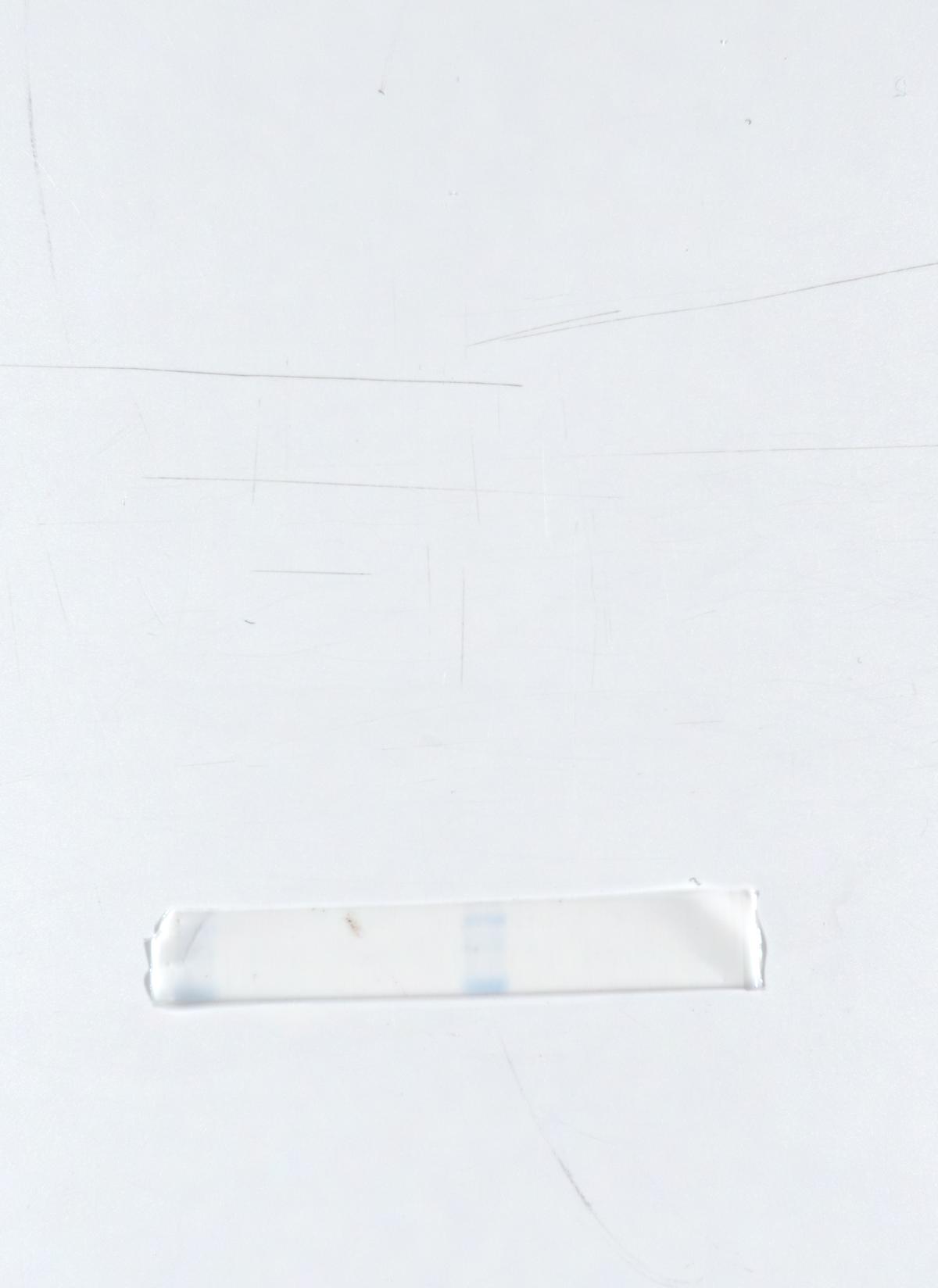

Supplement: Supplementary file 1 [file vetsci-12-01186-s001.zip › Supplementary Files/WB uncropped figure/Figure S1/PERK111 20250412_133355_Ch/PERK111 20250412_133355_Ch-Marker.jpg]

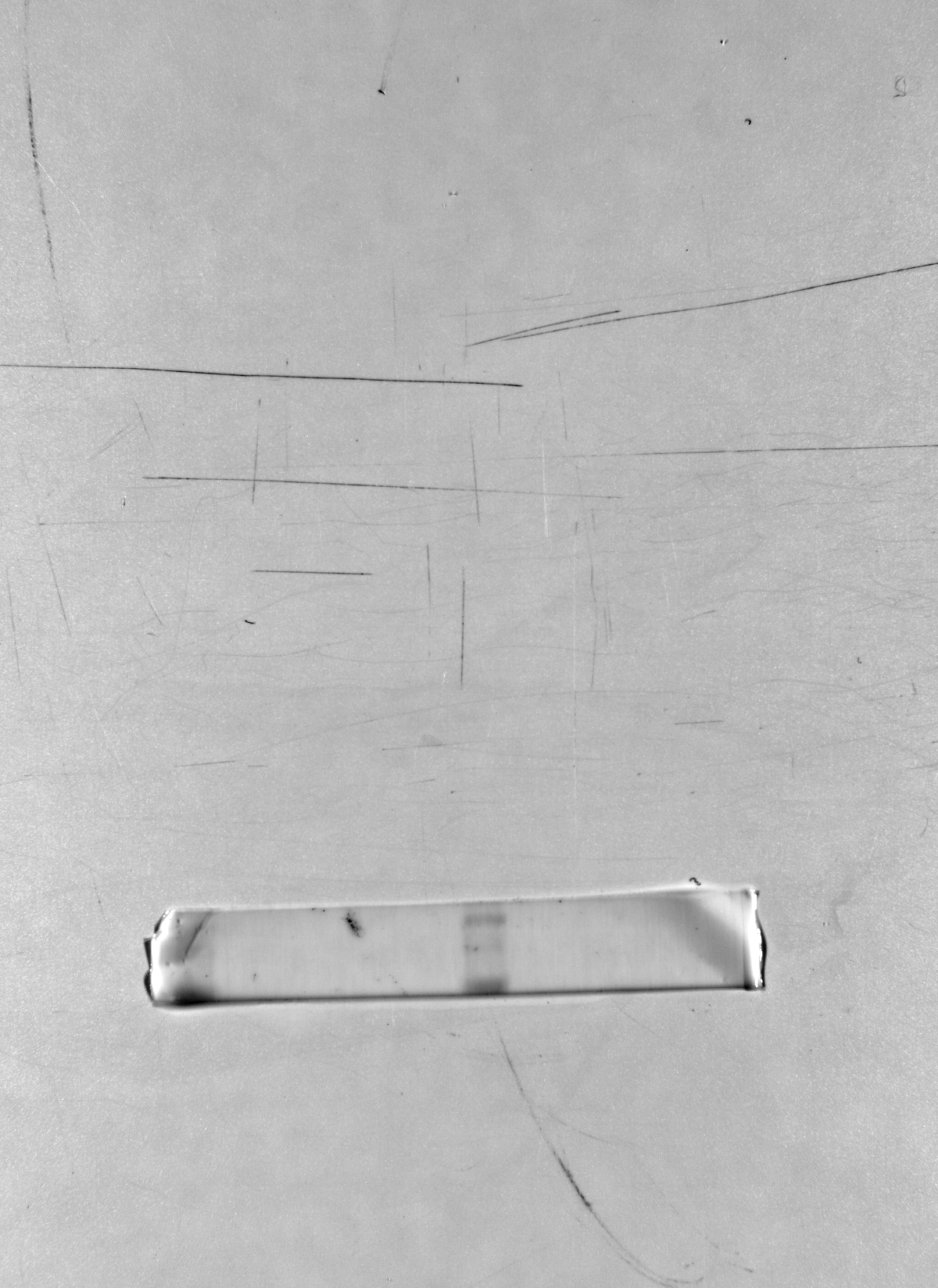

Supplement: Supplementary file 1 [file vetsci-12-01186-s001.zip › Supplementary Files/WB uncropped figure/Figure S1/PERK111 20250412_133355_Ch/PERK111 20250412_133355_Ch-Marker.tif]

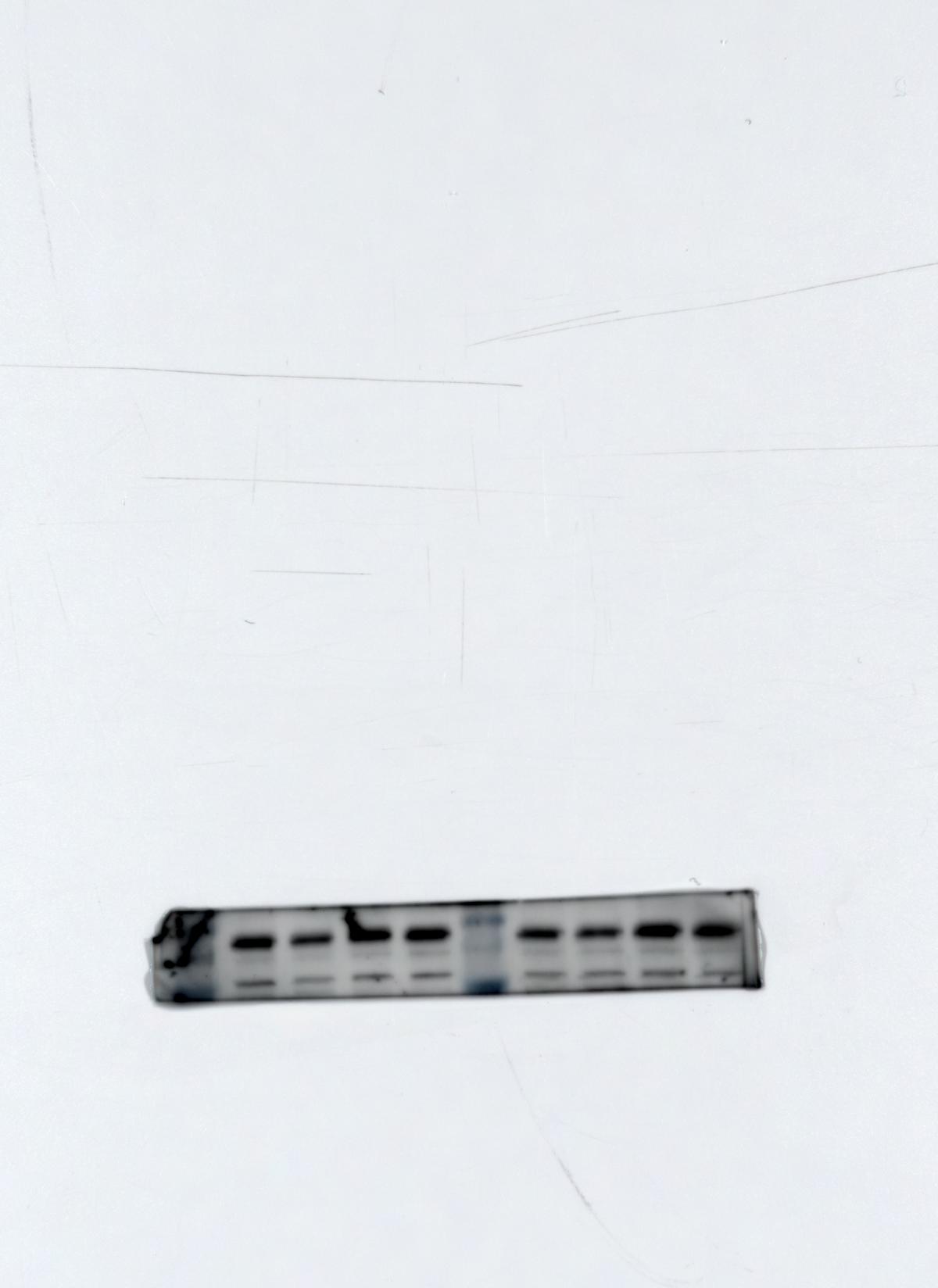

Supplement: Supplementary file 1 [file vetsci-12-01186-s001.zip › Supplementary Files/WB uncropped figure/Figure S1/PERK111 20250412_133355_Ch/PERK111 20250412_133355_Ch_Chemi+Marker.jpg]

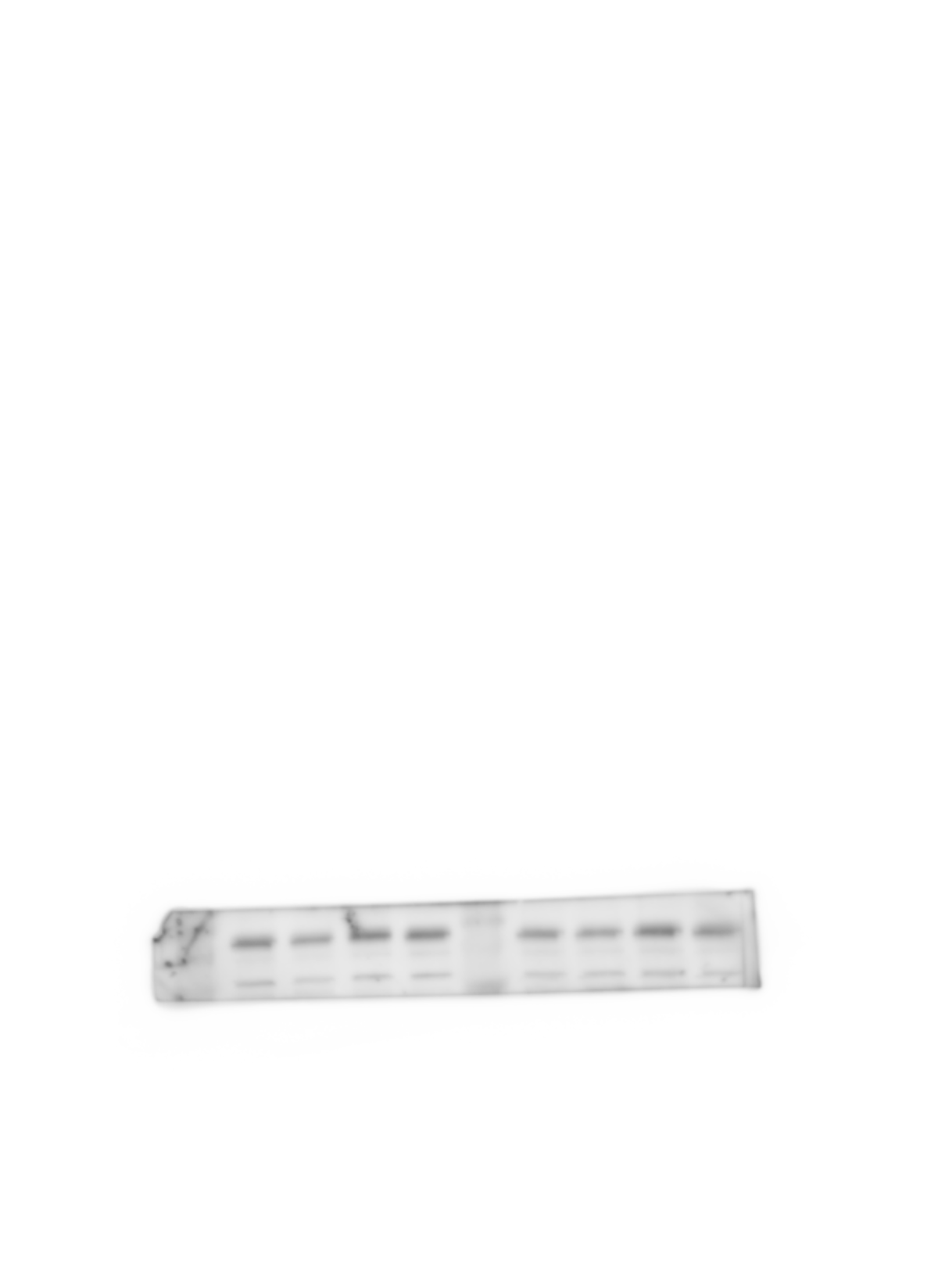

Supplement: Supplementary file 1 [file vetsci-12-01186-s001.zip › Supplementary Files/WB uncropped figure/Figure S1/PERK111 20250412_133355_Ch/PERK111 20250412_133355_Ch_Chemi.tif]

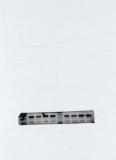

Supplement: Supplementary file 1 [file vetsci-12-01186-s001.zip › Supplementary Files/WB uncropped figure/Figure S1/PERK111 20250412_133355_Ch/PERK111 20250412_133355_Ch_Thumb.jpg]

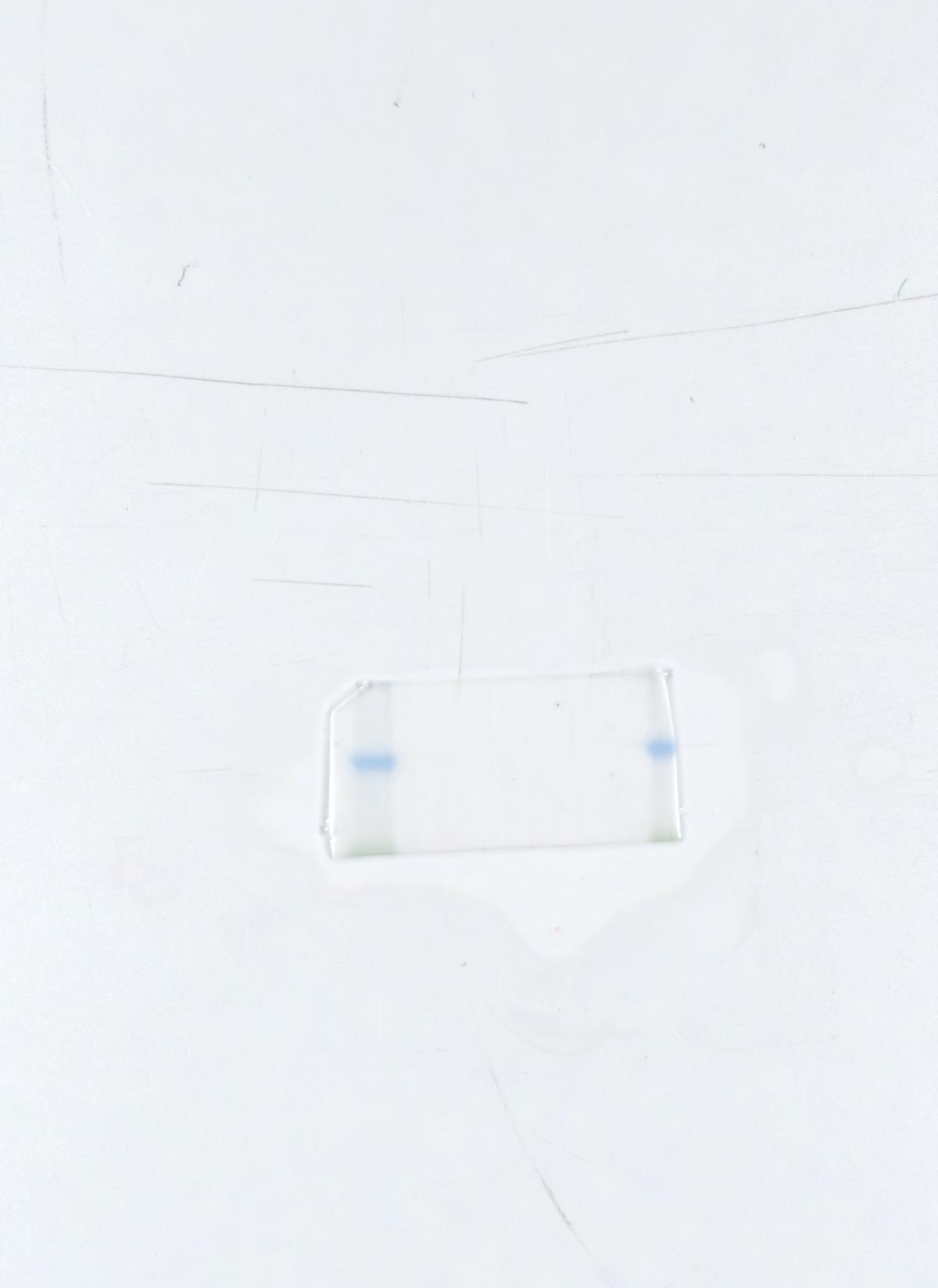

Supplement: Supplementary file 1 [file vetsci-12-01186-s001.zip › Supplementary Files/WB uncropped figure/Figure S2/eif2 20250415_150642_Ch/eif2 20250415_150642_Ch-Marker.jpg]

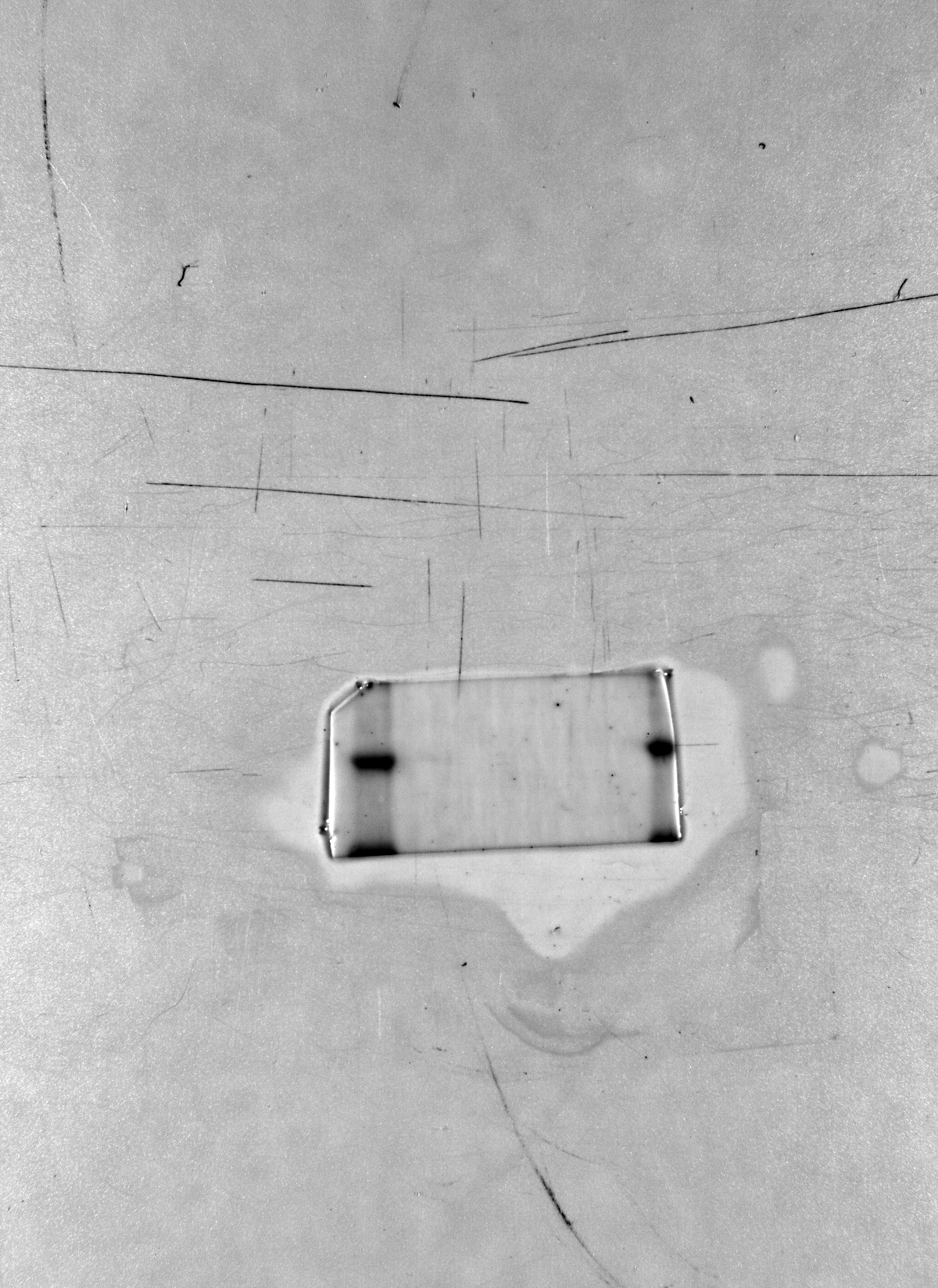

Supplement: Supplementary file 1 [file vetsci-12-01186-s001.zip › Supplementary Files/WB uncropped figure/Figure S2/eif2 20250415_150642_Ch/eif2 20250415_150642_Ch-Marker.tif]

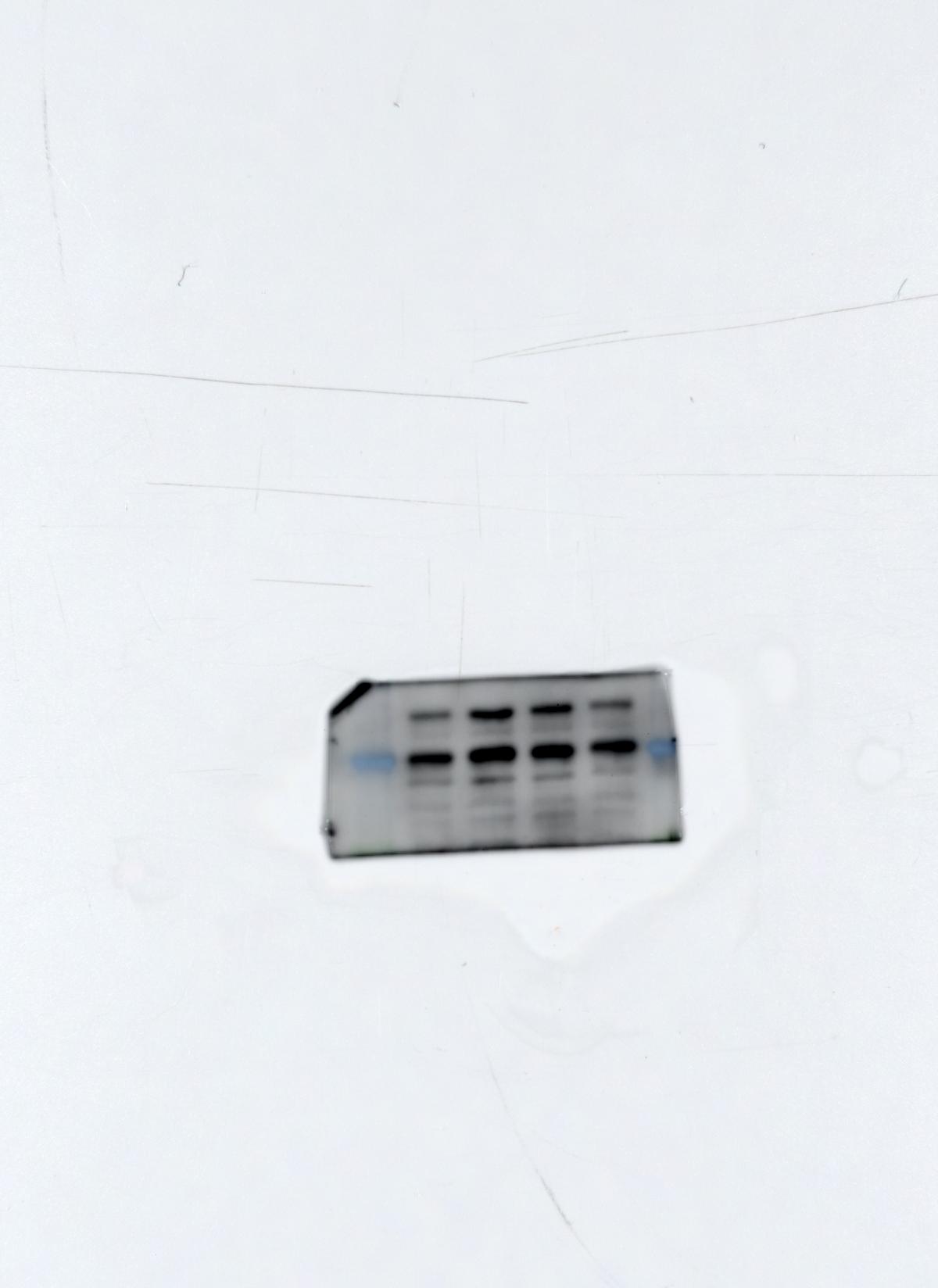

Supplement: Supplementary file 1 [file vetsci-12-01186-s001.zip › Supplementary Files/WB uncropped figure/Figure S2/eif2 20250415_150642_Ch/eif2 20250415_150642_Ch_Chemi+Marker.jpg]

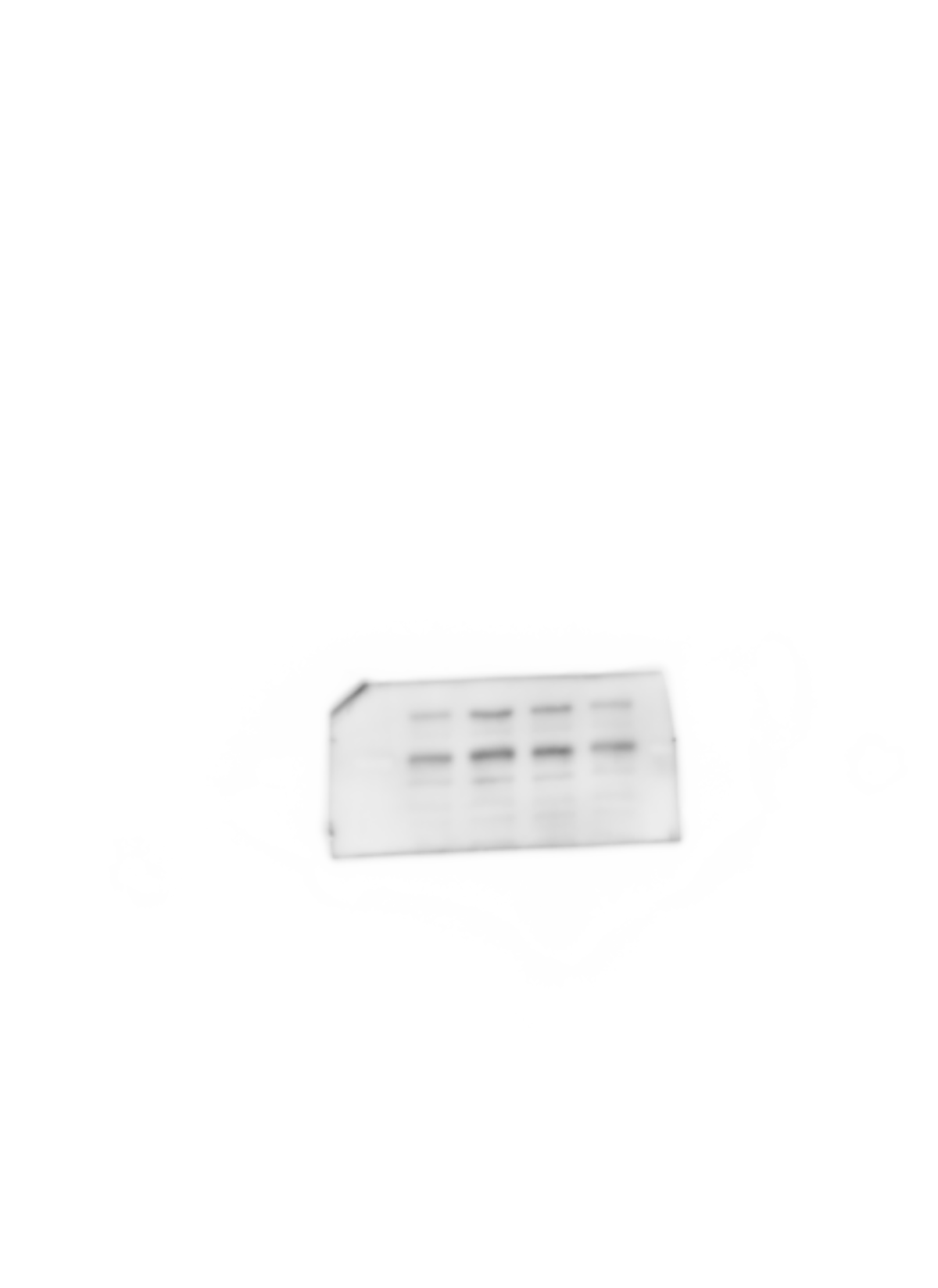

Supplement: Supplementary file 1 [file vetsci-12-01186-s001.zip › Supplementary Files/WB uncropped figure/Figure S2/eif2 20250415_150642_Ch/eif2 20250415_150642_Ch_Chemi.tif]

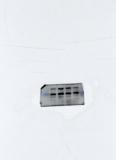

Supplement: Supplementary file 1 [file vetsci-12-01186-s001.zip › Supplementary Files/WB uncropped figure/Figure S2/eif2 20250415_150642_Ch/eif2 20250415_150642_Ch_Thumb.jpg]

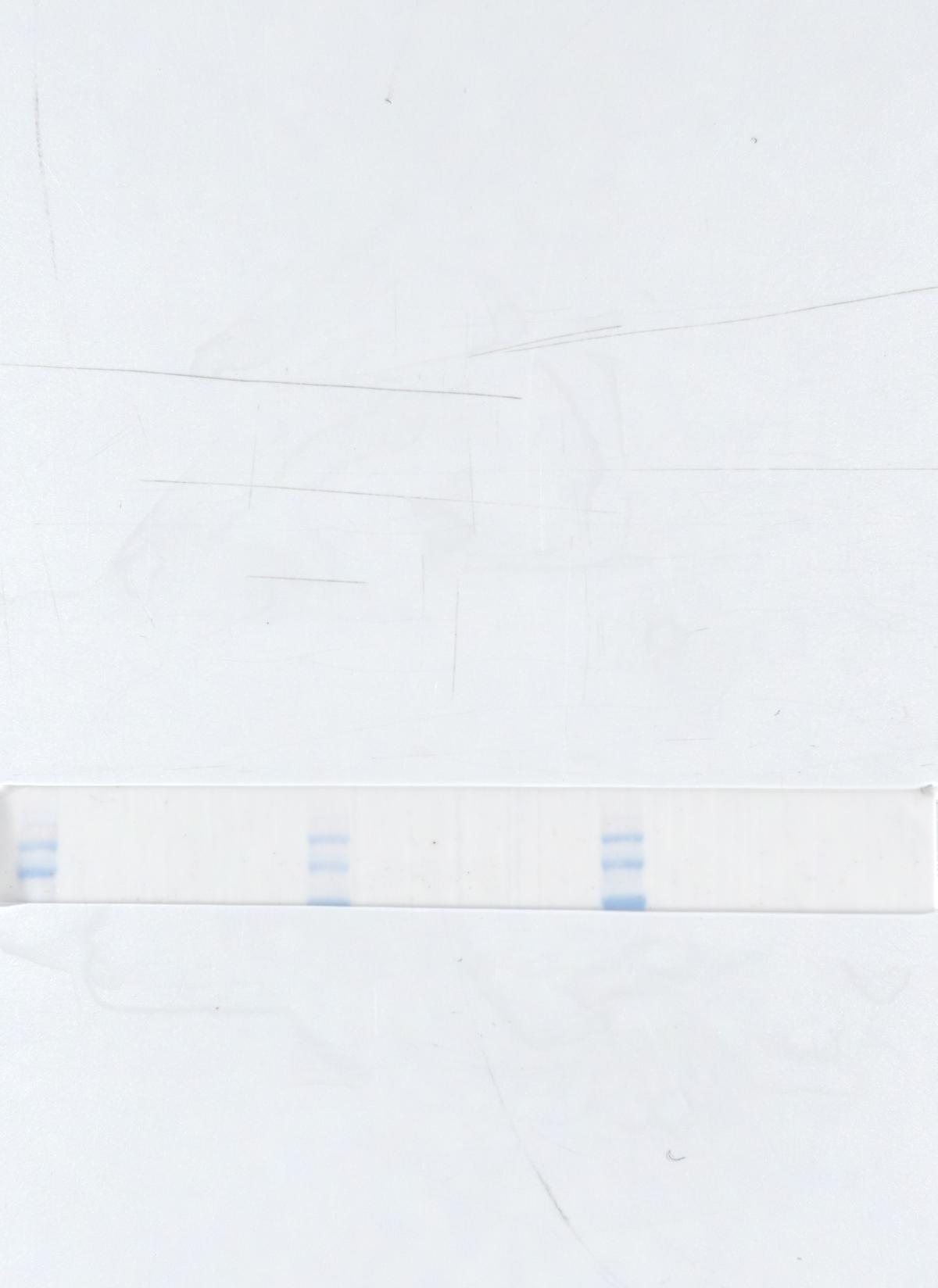

Supplement: Supplementary file 1 [file vetsci-12-01186-s001.zip › Supplementary Files/WB uncropped figure/Figure S2/p-PERK 2 20250426_125030_Ch/p-PERK 2 20250426_125030_Ch-Marker.jpg]

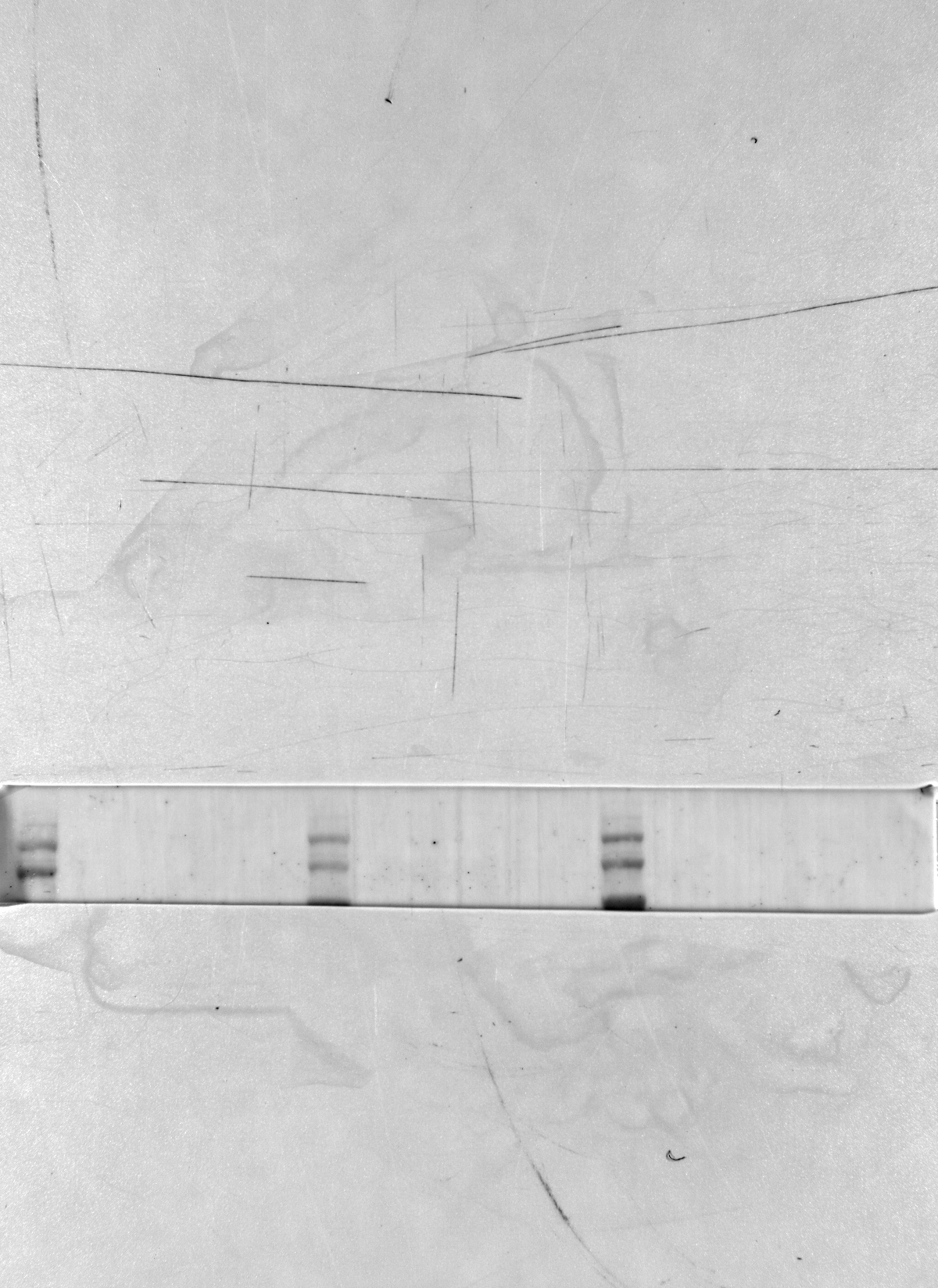

Supplement: Supplementary file 1 [file vetsci-12-01186-s001.zip › Supplementary Files/WB uncropped figure/Figure S2/p-PERK 2 20250426_125030_Ch/p-PERK 2 20250426_125030_Ch-Marker.tif]

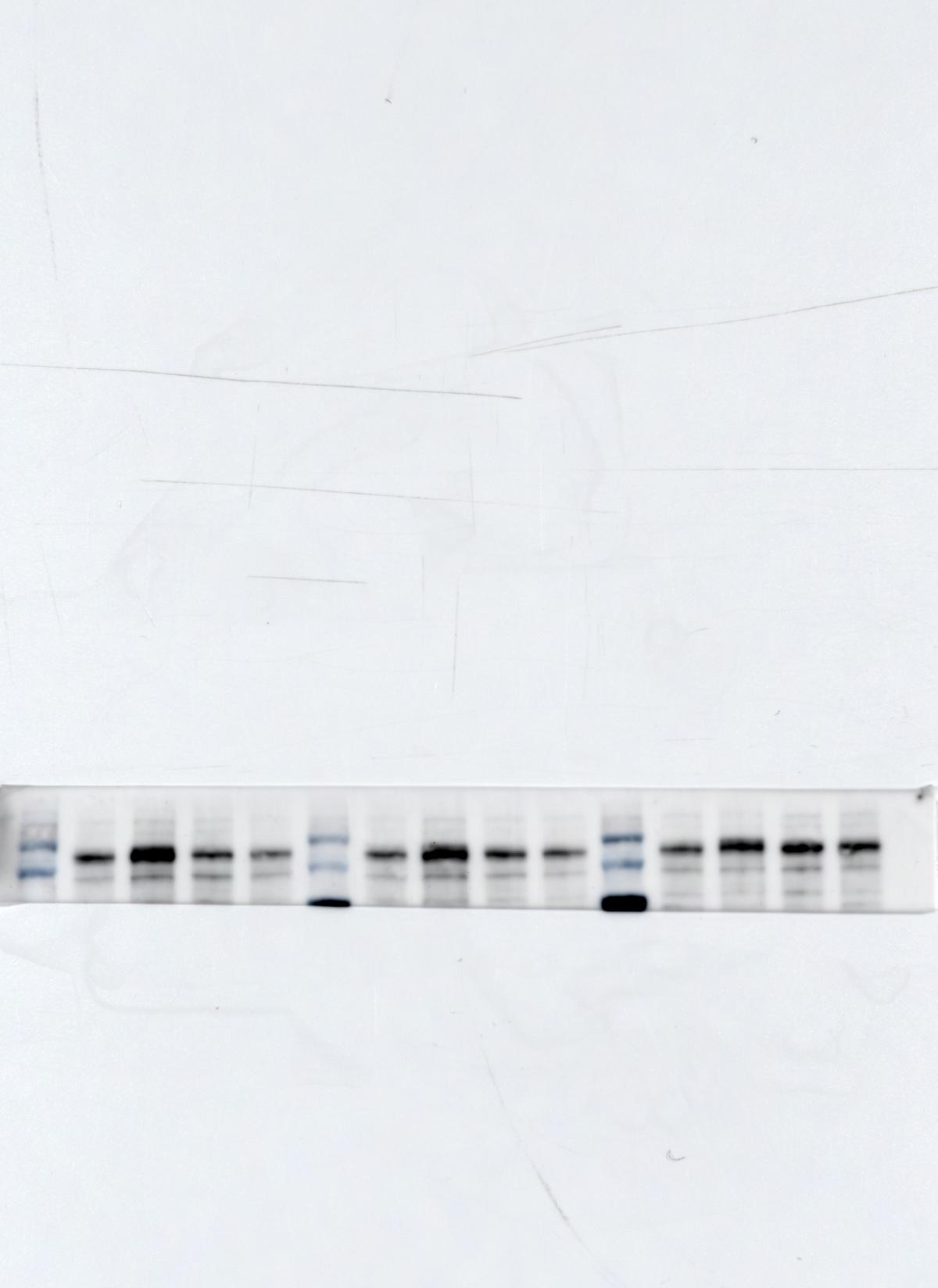

Supplement: Supplementary file 1 [file vetsci-12-01186-s001.zip › Supplementary Files/WB uncropped figure/Figure S2/p-PERK 2 20250426_125030_Ch/p-PERK 2 20250426_125030_Ch_Chemi+Marker.jpg]

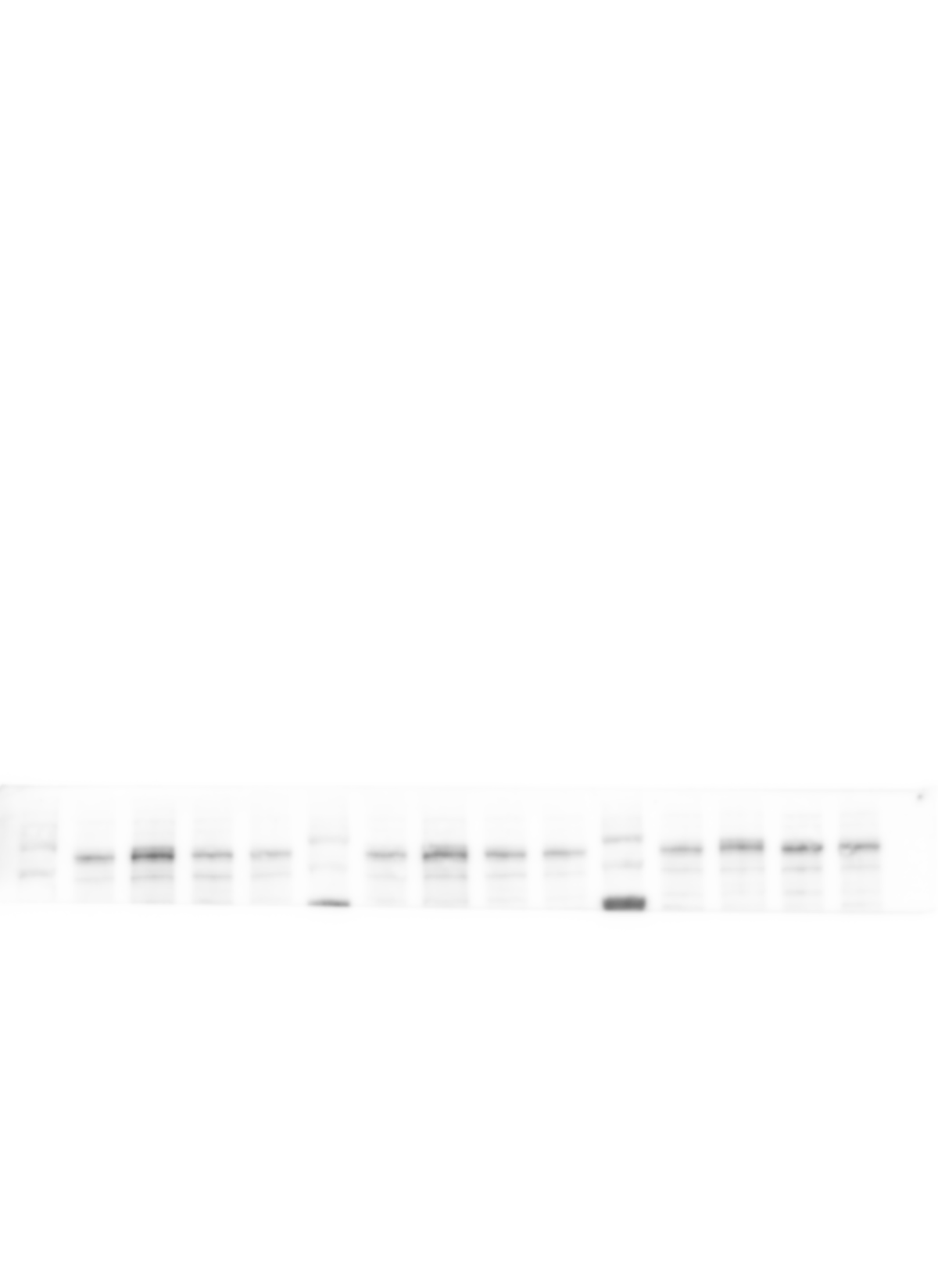

Supplement: Supplementary file 1 [file vetsci-12-01186-s001.zip › Supplementary Files/WB uncropped figure/Figure S2/p-PERK 2 20250426_125030_Ch/p-PERK 2 20250426_125030_Ch_Chemi.tif]

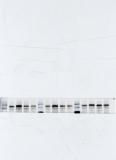

Supplement: Supplementary file 1 [file vetsci-12-01186-s001.zip › Supplementary Files/WB uncropped figure/Figure S2/p-PERK 2 20250426_125030_Ch/p-PERK 2 20250426_125030_Ch_Thumb.jpg]

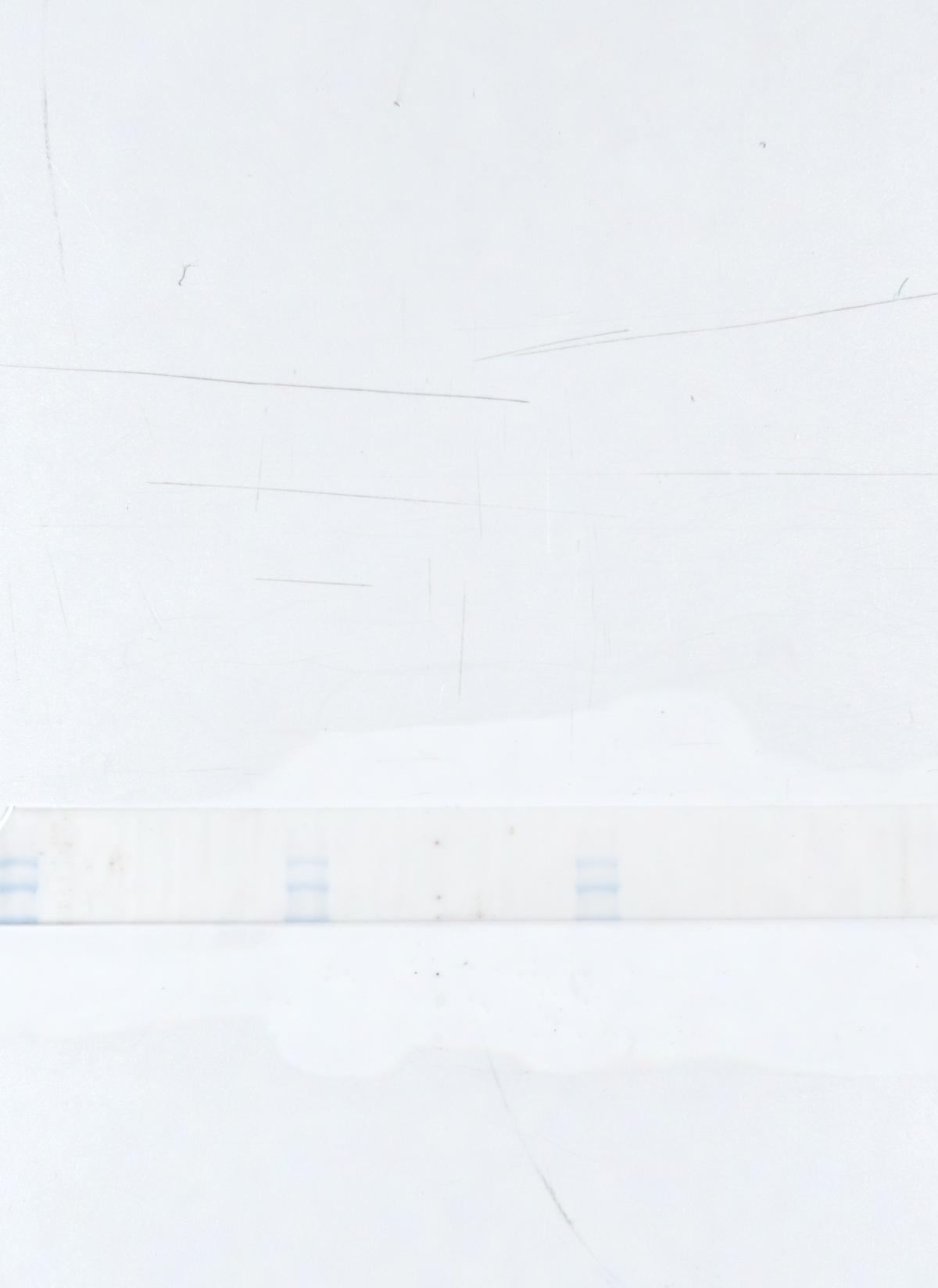

Supplement: Supplementary file 1 [file vetsci-12-01186-s001.zip › Supplementary Files/WB uncropped figure/Figure S2/PERK2 20250415_151315_Ch/PERK 20250415_151315_Ch-Marker.jpg]

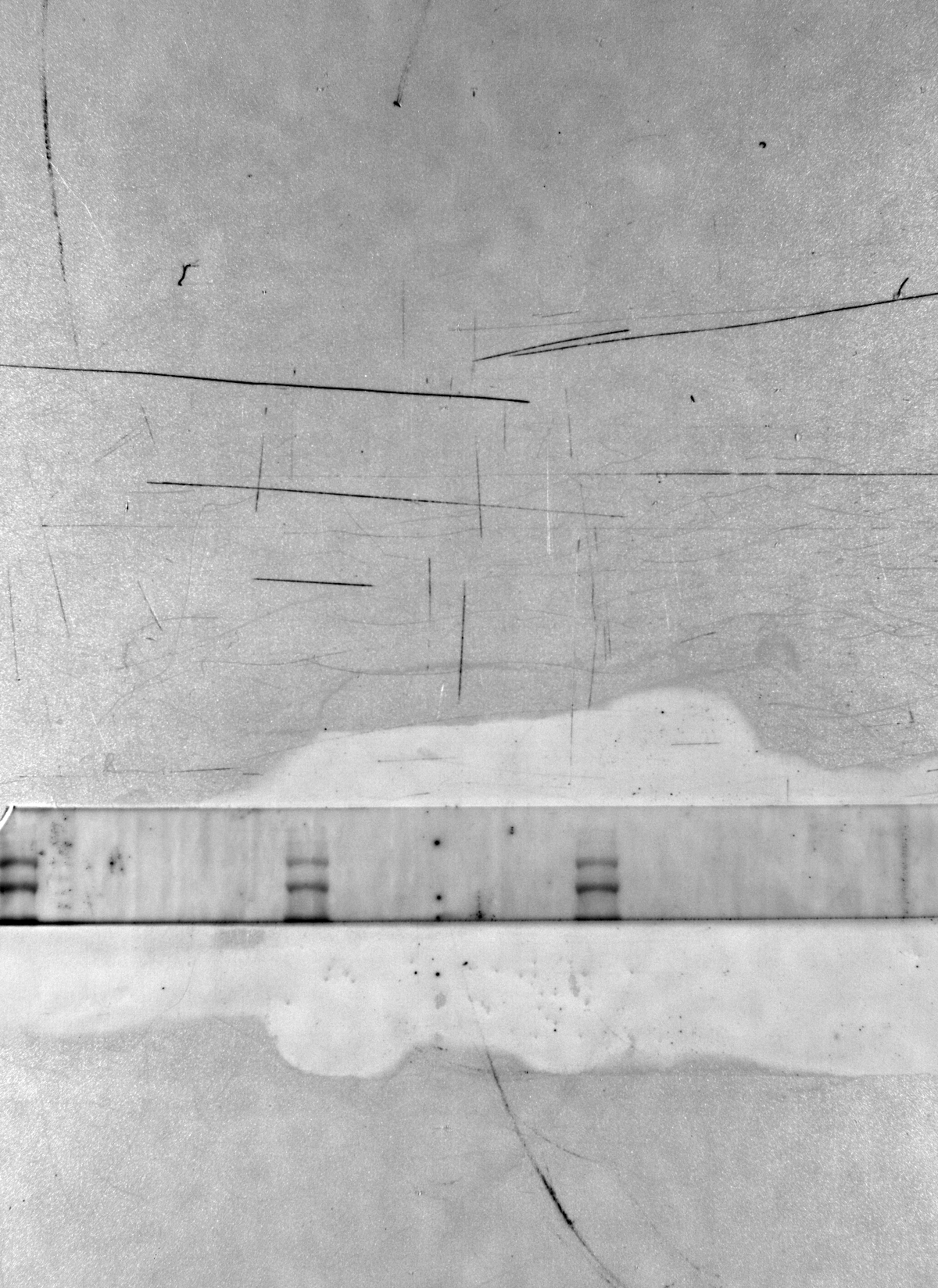

Supplement: Supplementary file 1 [file vetsci-12-01186-s001.zip › Supplementary Files/WB uncropped figure/Figure S2/PERK2 20250415_151315_Ch/PERK 20250415_151315_Ch-Marker.tif]

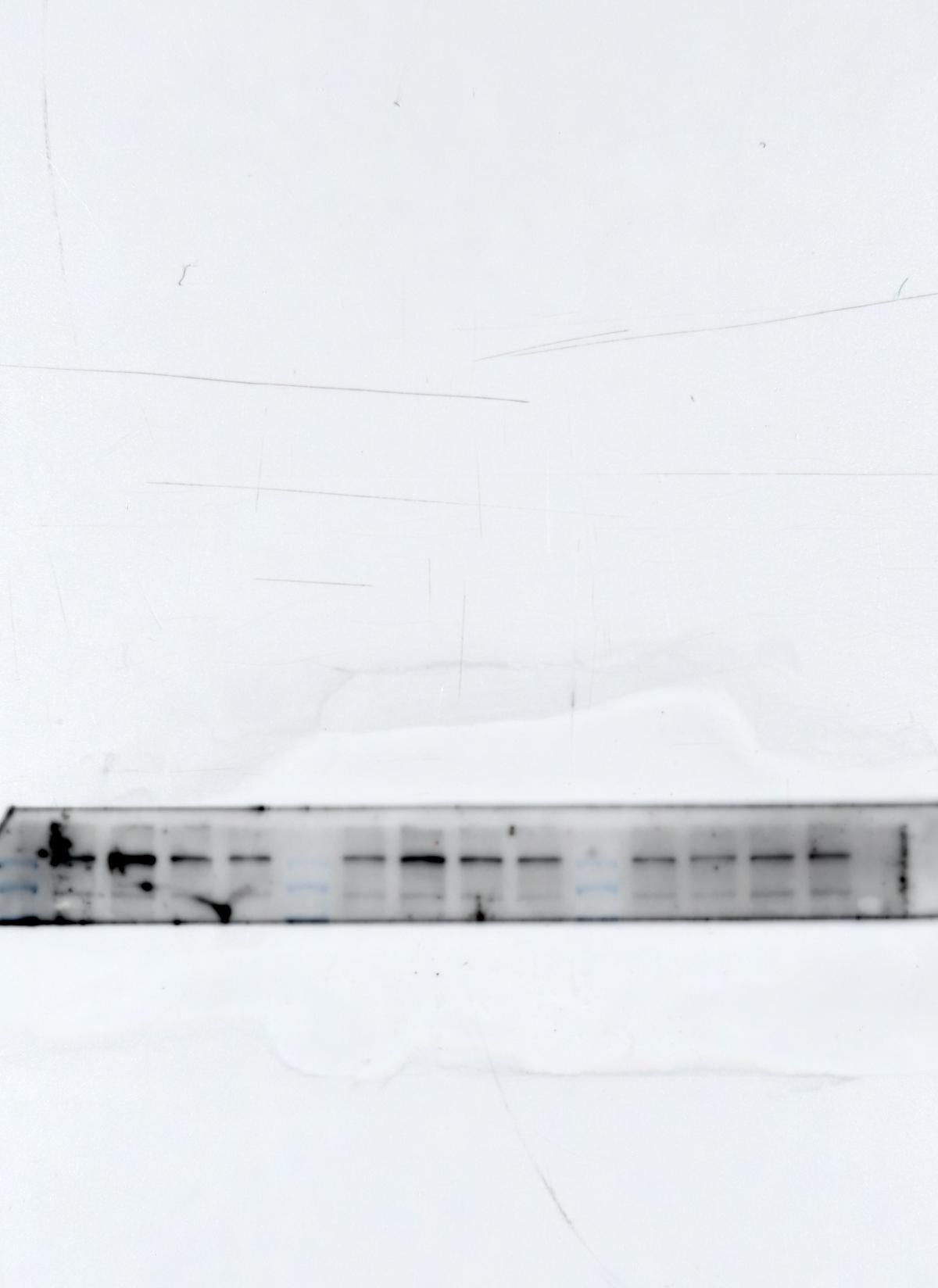

Supplement: Supplementary file 1 [file vetsci-12-01186-s001.zip › Supplementary Files/WB uncropped figure/Figure S2/PERK2 20250415_151315_Ch/PERK 20250415_151315_Ch_Chemi+Marker.jpg]

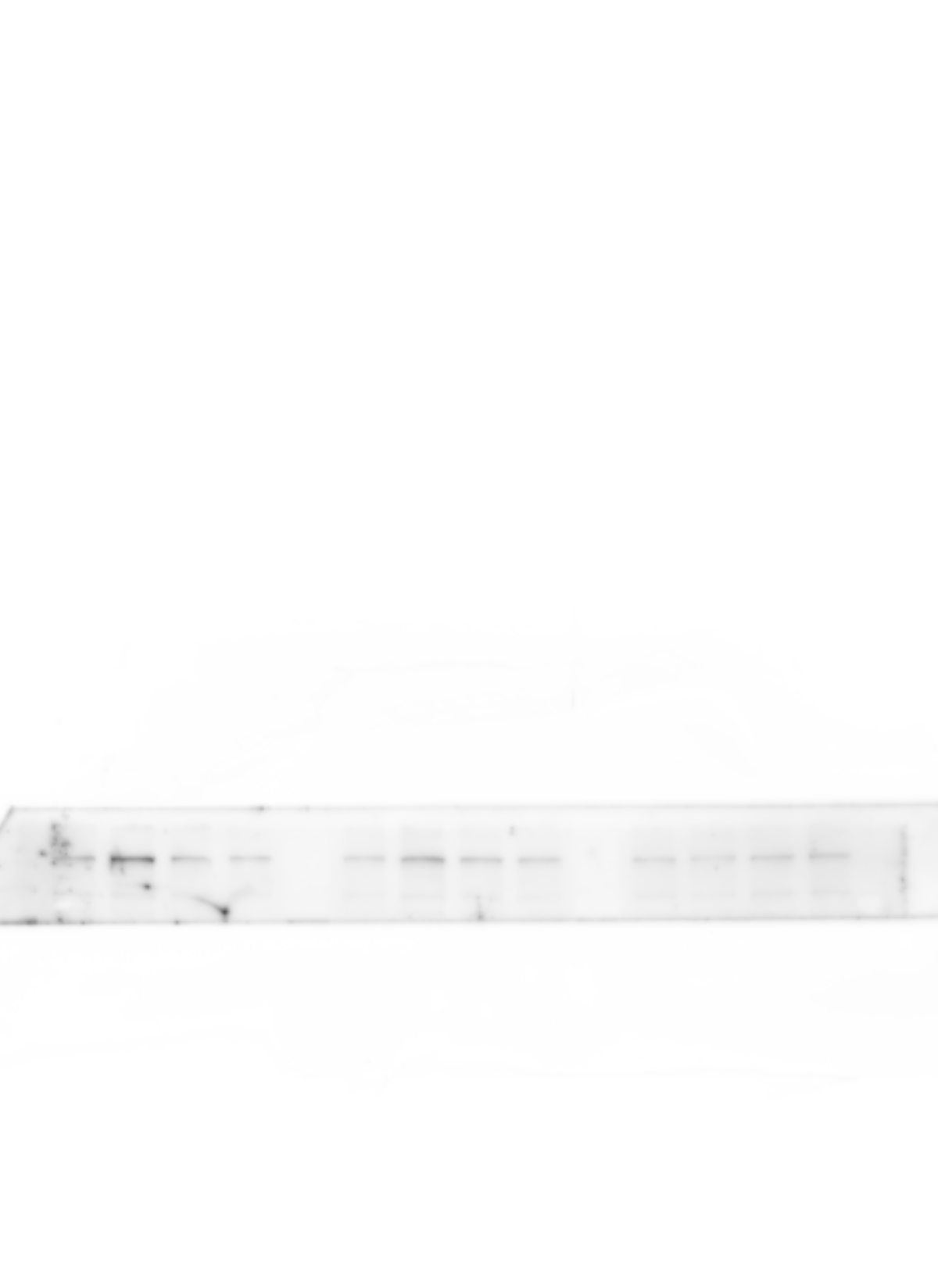

Supplement: Supplementary file 1 [file vetsci-12-01186-s001.zip › Supplementary Files/WB uncropped figure/Figure S2/PERK2 20250415_151315_Ch/PERK 20250415_151315_Ch_Chemi.tif]

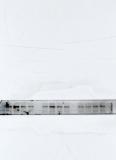

Supplement: Supplementary file 1 [file vetsci-12-01186-s001.zip › Supplementary Files/WB uncropped figure/Figure S2/PERK2 20250415_151315_Ch/PERK 20250415_151315_Ch_Thumb.jpg]

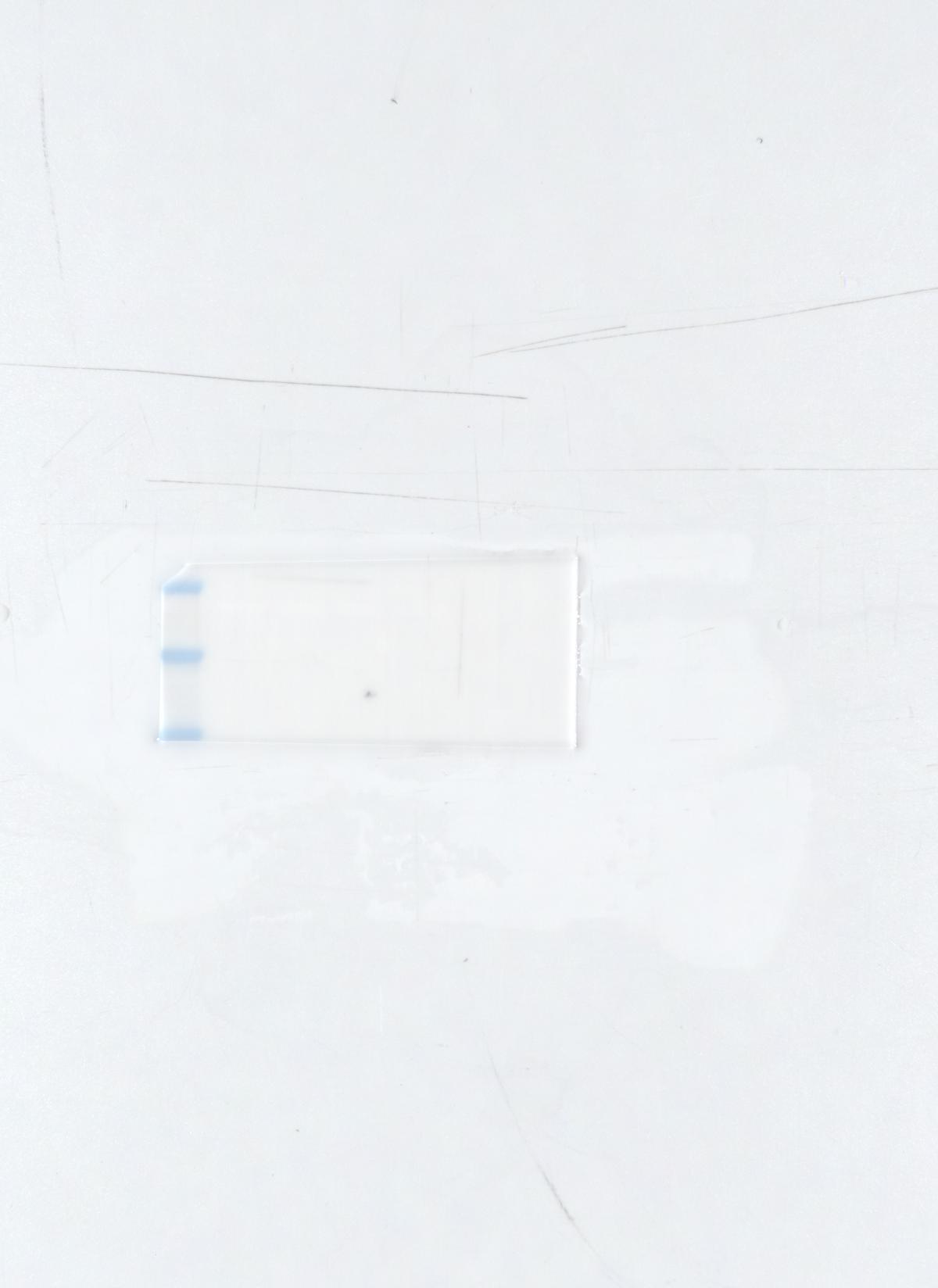

Supplement: Supplementary file 1 [file vetsci-12-01186-s001.zip › Supplementary Files/WB uncropped figure/Figure S2/Tubulin 2 20250523_135638_Ch/Tubulin 2 20250523_135638_Ch-Marker.jpg]

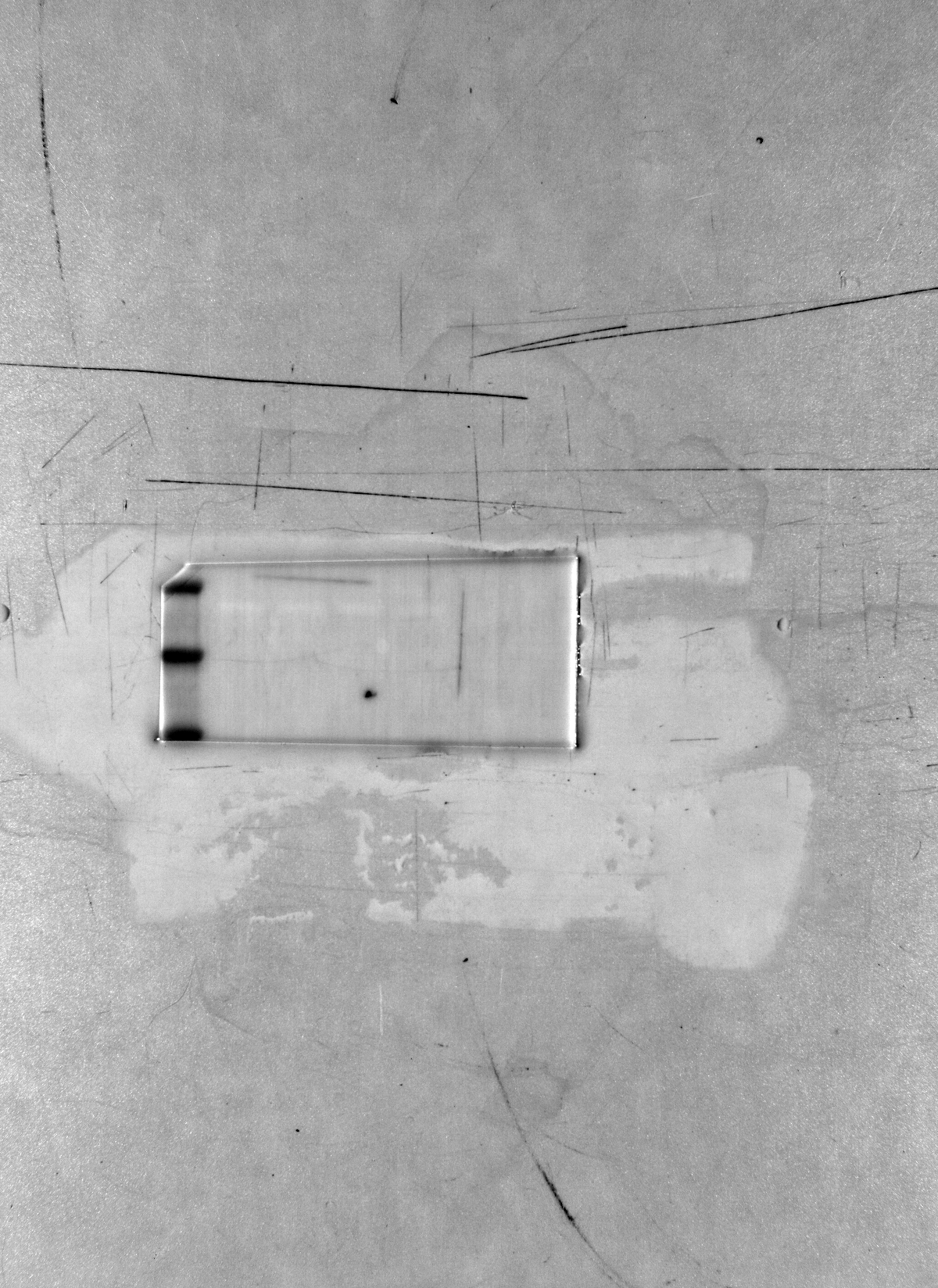

Supplement: Supplementary file 1 [file vetsci-12-01186-s001.zip › Supplementary Files/WB uncropped figure/Figure S2/Tubulin 2 20250523_135638_Ch/Tubulin 2 20250523_135638_Ch-Marker.tif]

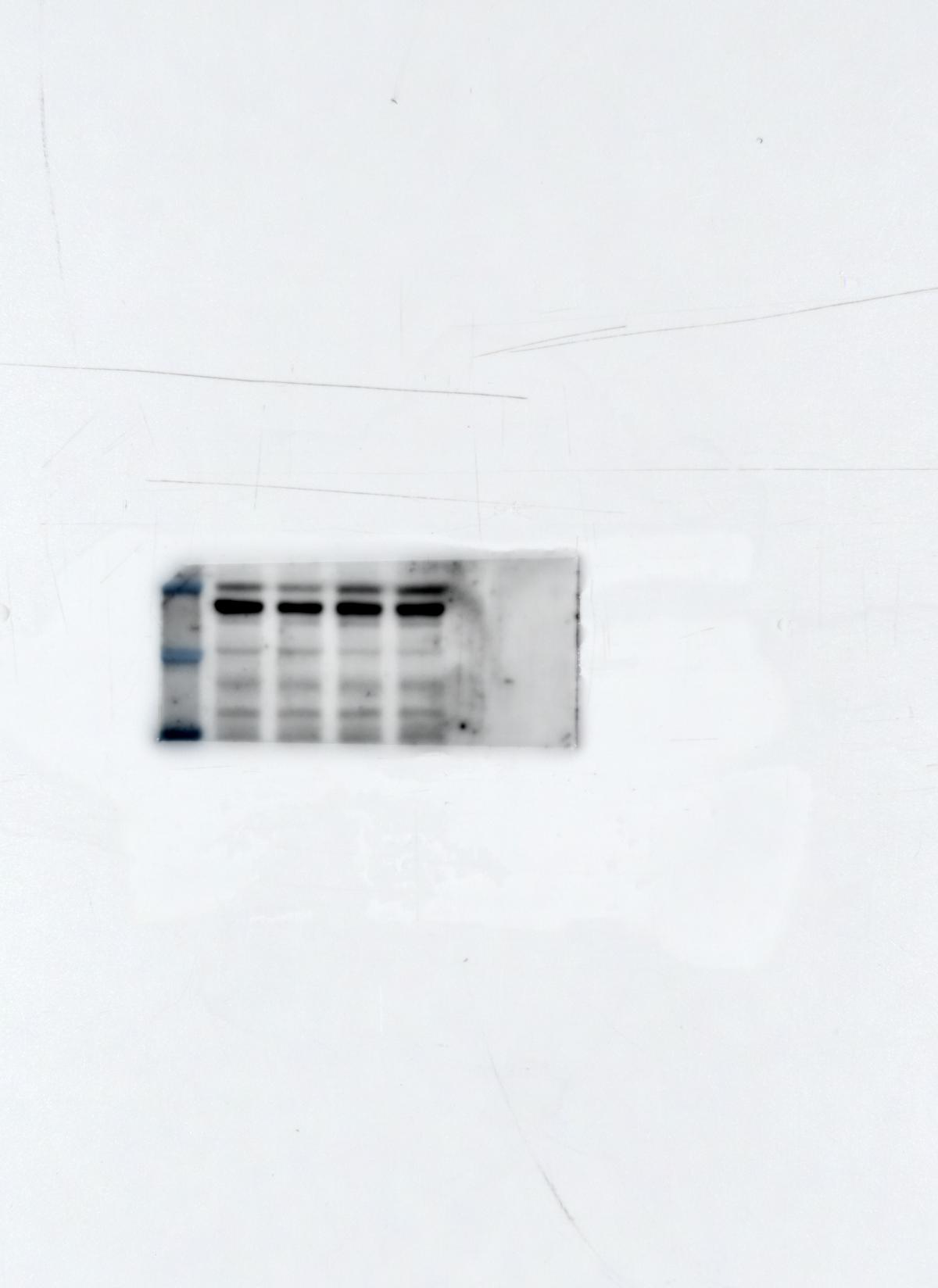

Supplement: Supplementary file 1 [file vetsci-12-01186-s001.zip › Supplementary Files/WB uncropped figure/Figure S2/Tubulin 2 20250523_135638_Ch/Tubulin 2 20250523_135638_Ch_Chemi+Marker.jpg]

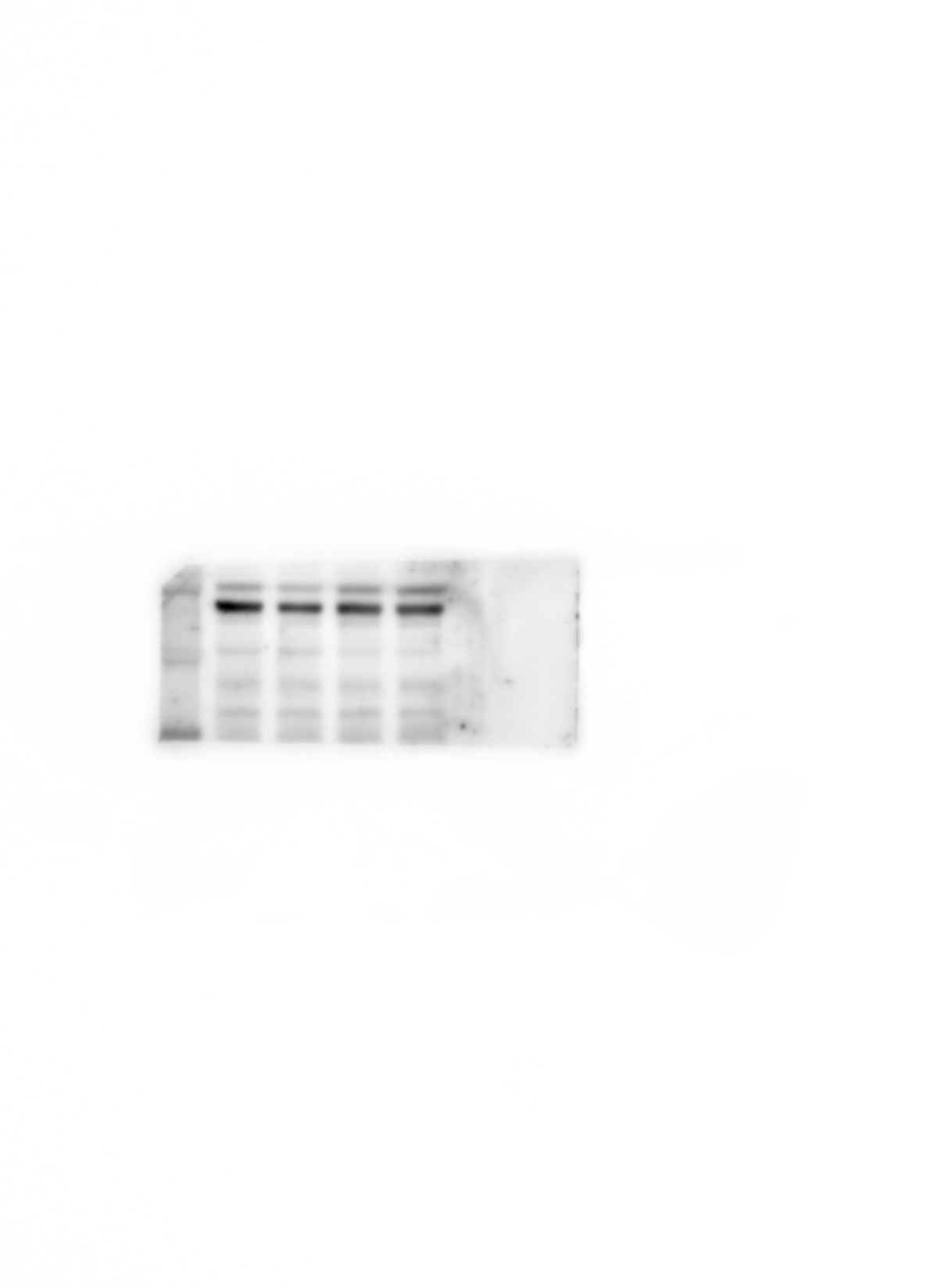

Supplement: Supplementary file 1 [file vetsci-12-01186-s001.zip › Supplementary Files/WB uncropped figure/Figure S2/Tubulin 2 20250523_135638_Ch/Tubulin 2 20250523_135638_Ch_Chemi.tif]

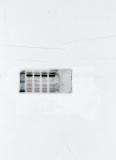

Supplement: Supplementary file 1 [file vetsci-12-01186-s001.zip › Supplementary Files/WB uncropped figure/Figure S2/Tubulin 2 20250523_135638_Ch/Tubulin 2 20250523_135638_Ch_Thumb.jpg]

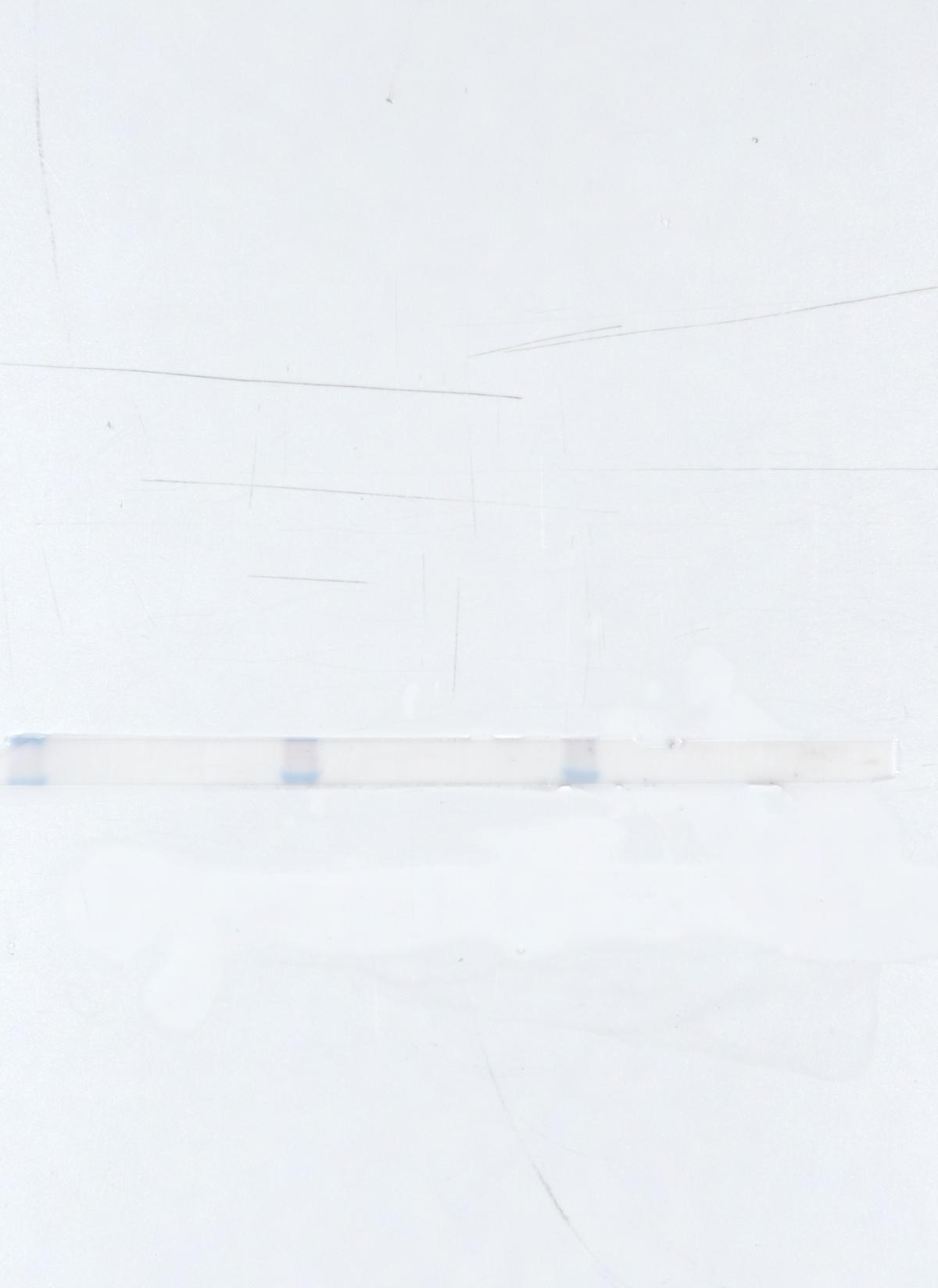

Supplement: Supplementary file 1 [file vetsci-12-01186-s001.zip › Supplementary Files/WB uncropped figure/Figure S3/ATF4 20250421_163344_Ch/ATF4 20250421_163344_Ch-Marker.jpg]

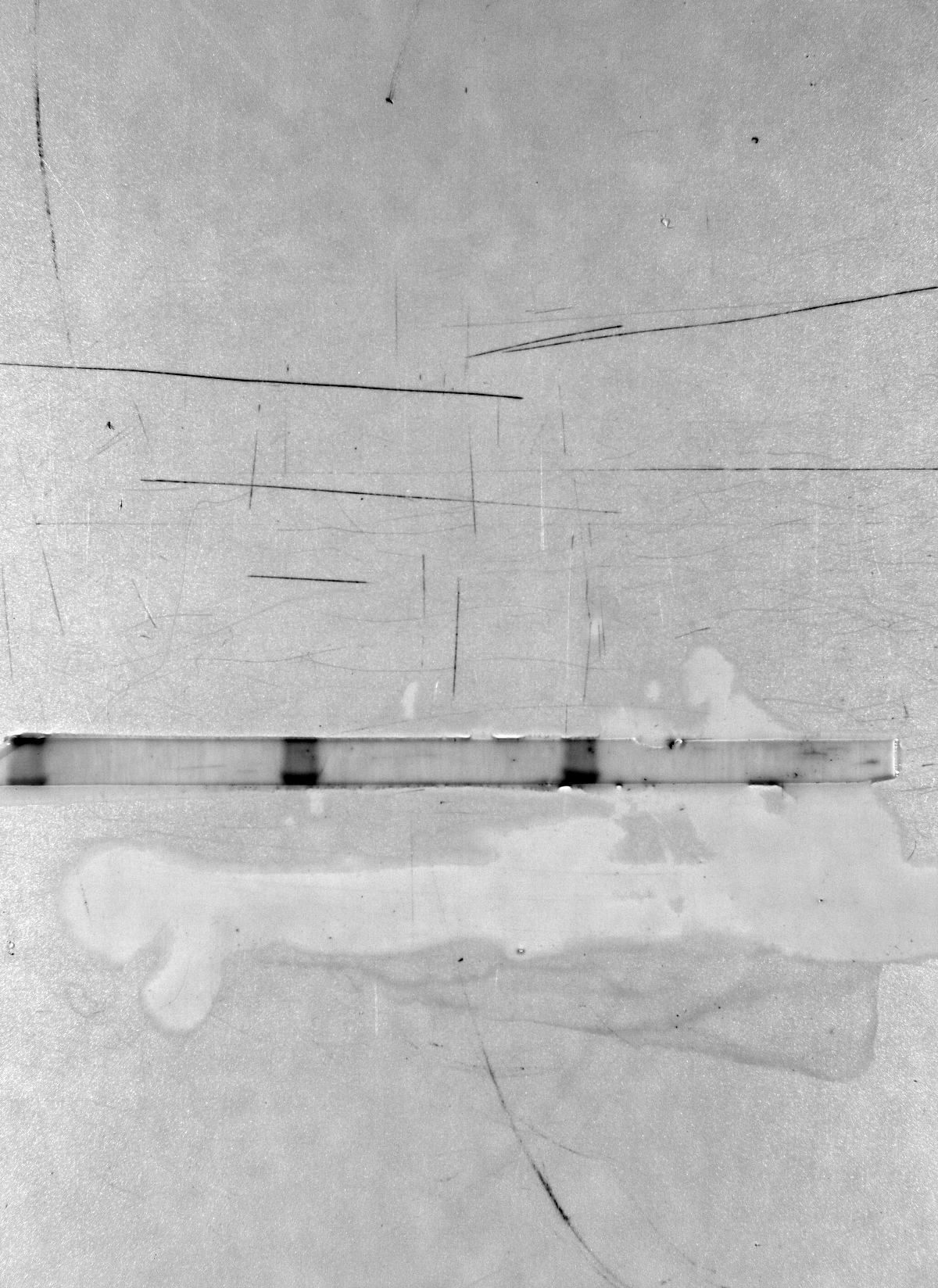

Supplement: Supplementary file 1 [file vetsci-12-01186-s001.zip › Supplementary Files/WB uncropped figure/Figure S3/ATF4 20250421_163344_Ch/ATF4 20250421_163344_Ch-Marker.tif]

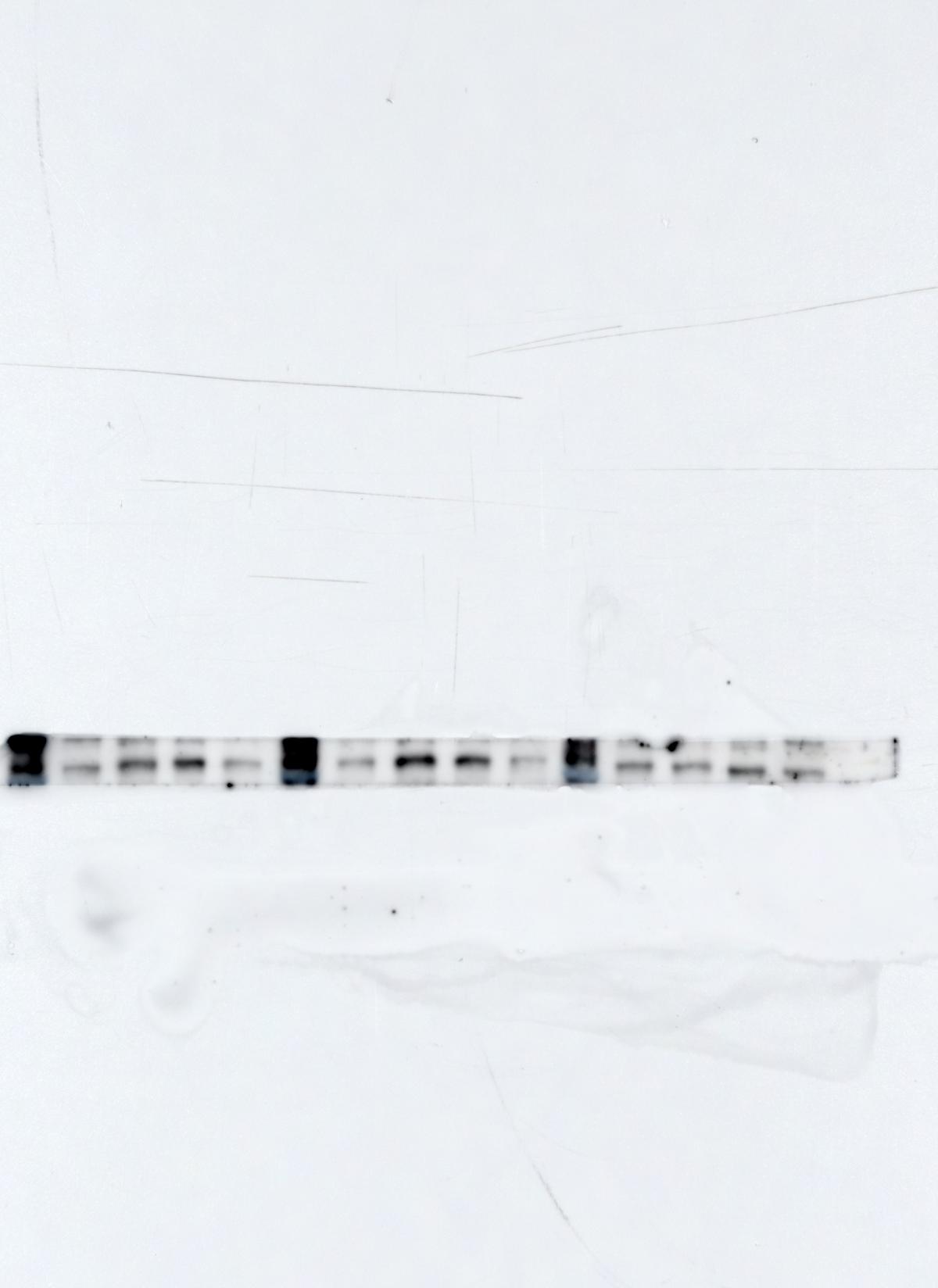

Supplement: Supplementary file 1 [file vetsci-12-01186-s001.zip › Supplementary Files/WB uncropped figure/Figure S3/ATF4 20250421_163344_Ch/ATF4 20250421_163344_Ch_Chemi+Marker.jpg]

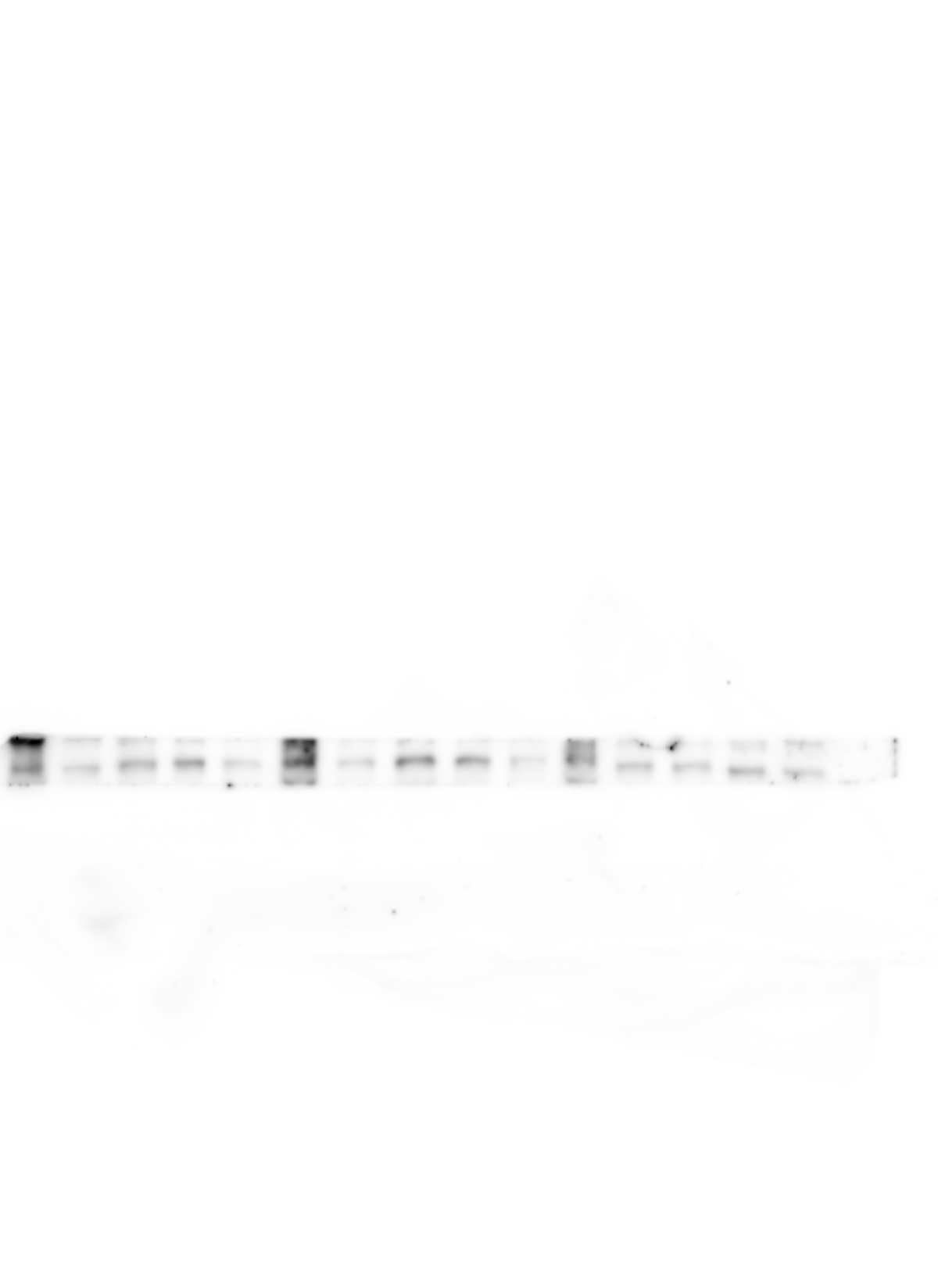

Supplement: Supplementary file 1 [file vetsci-12-01186-s001.zip › Supplementary Files/WB uncropped figure/Figure S3/ATF4 20250421_163344_Ch/ATF4 20250421_163344_Ch_Chemi.tif]

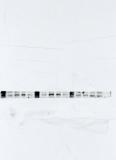

Supplement: Supplementary file 1 [file vetsci-12-01186-s001.zip › Supplementary Files/WB uncropped figure/Figure S3/ATF4 20250421_163344_Ch/ATF4 20250421_163344_Ch_Thumb.jpg]

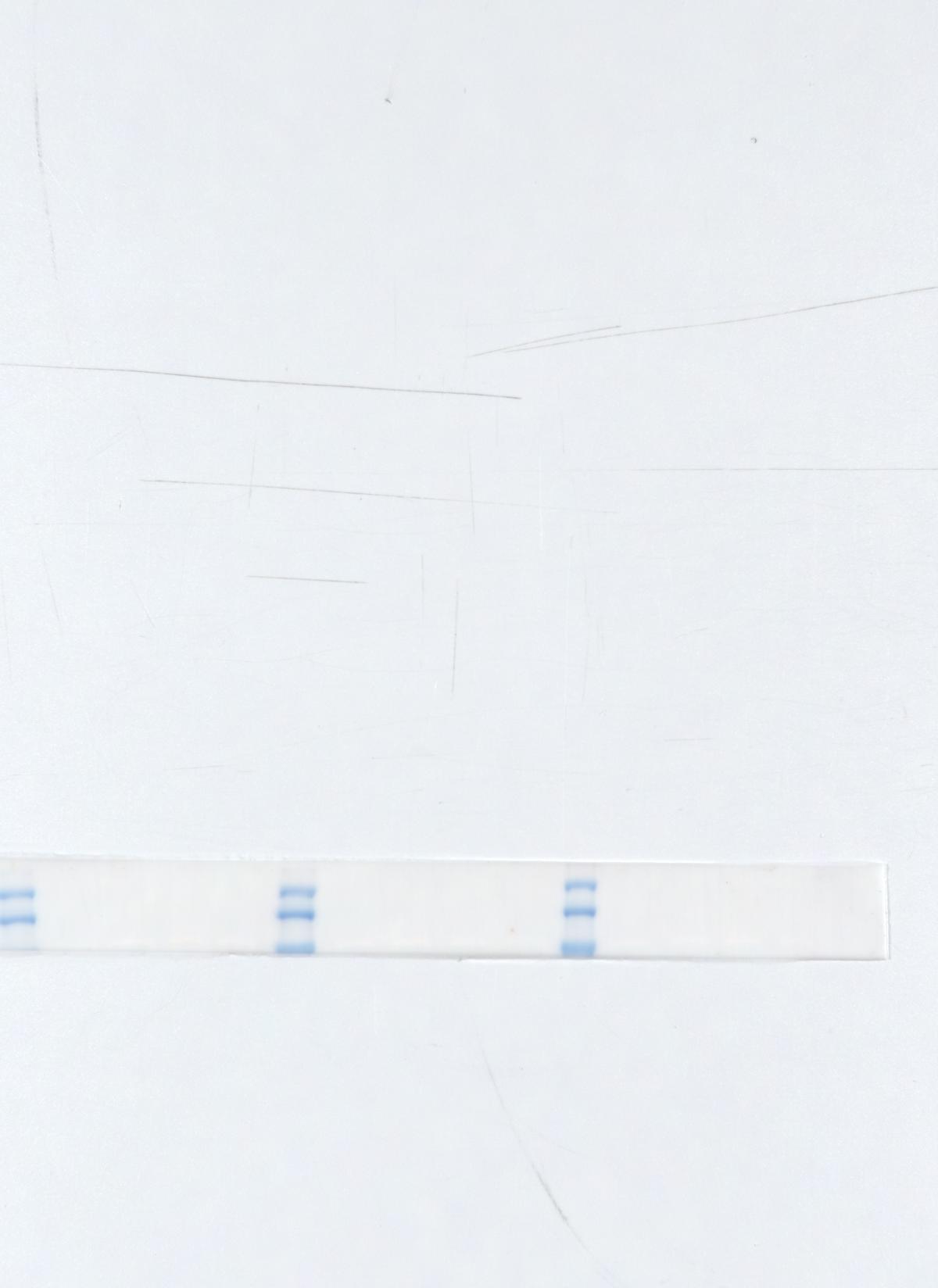

Supplement: Supplementary file 1 [file vetsci-12-01186-s001.zip › Supplementary Files/WB uncropped figure/Figure S3/GADD34 20250421_162258_Ch/GADD34 20250421_162258_Ch-Marker.jpg]

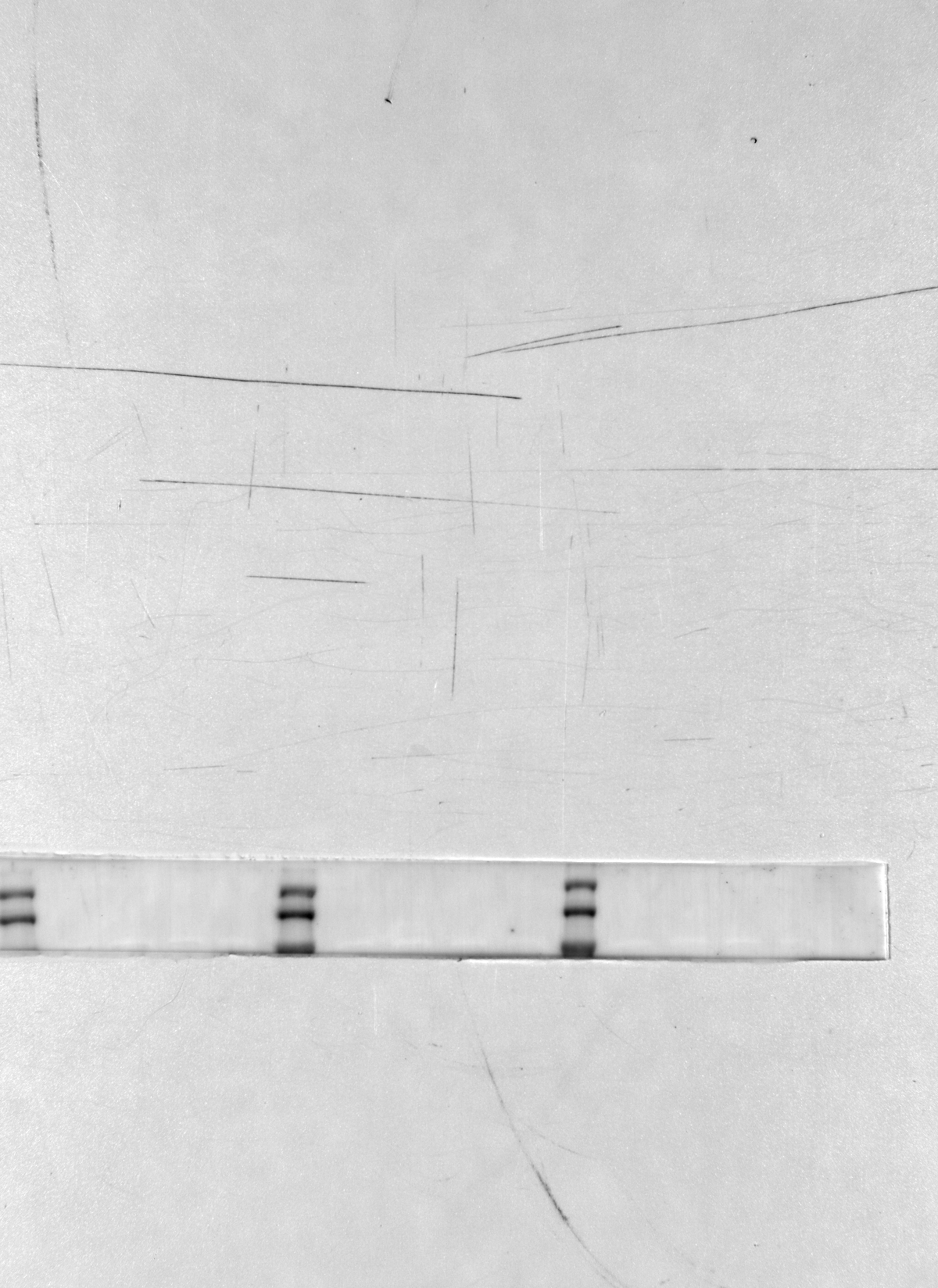

Supplement: Supplementary file 1 [file vetsci-12-01186-s001.zip › Supplementary Files/WB uncropped figure/Figure S3/GADD34 20250421_162258_Ch/GADD34 20250421_162258_Ch-Marker.tif]

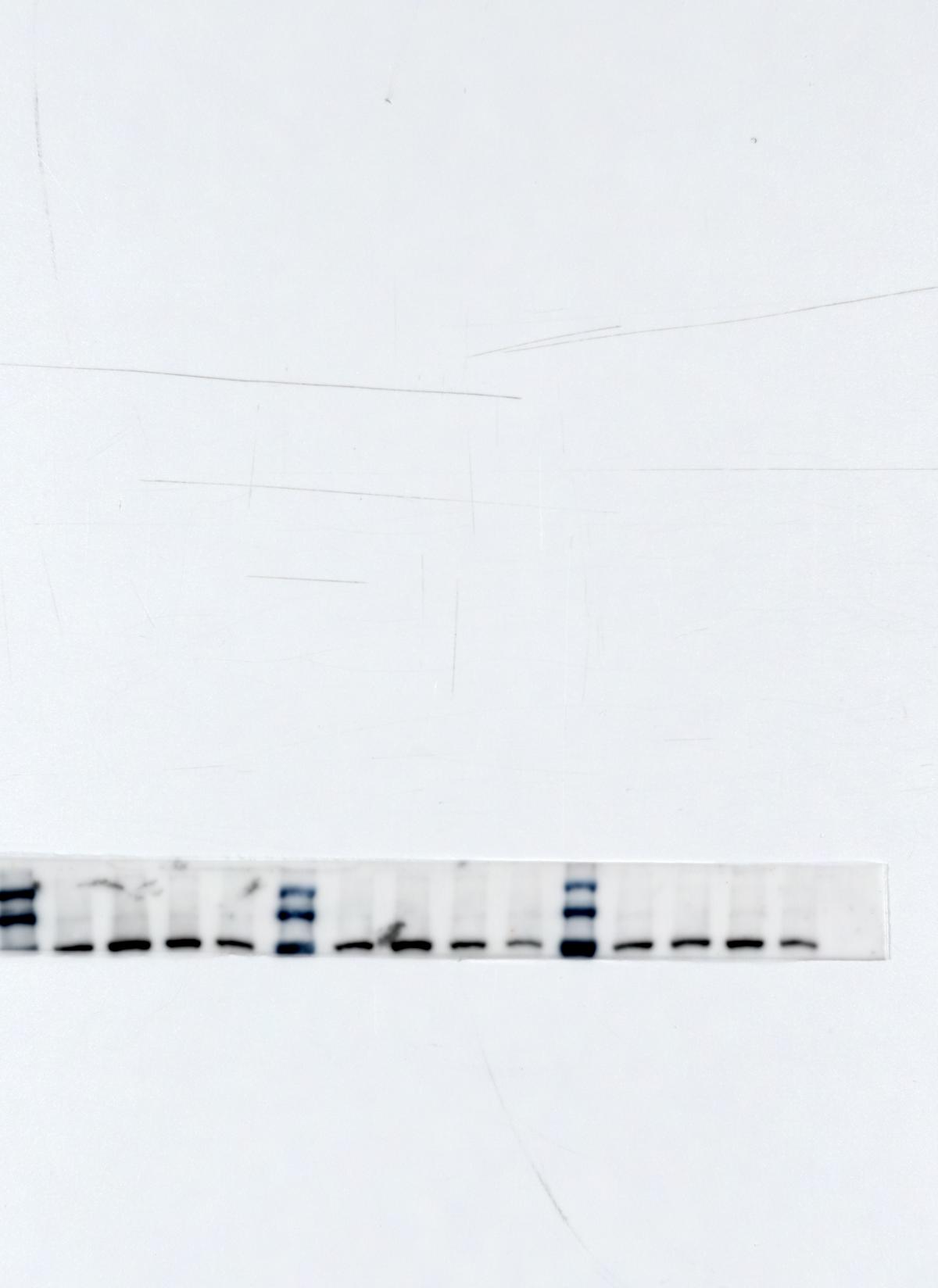

Supplement: Supplementary file 1 [file vetsci-12-01186-s001.zip › Supplementary Files/WB uncropped figure/Figure S3/GADD34 20250421_162258_Ch/GADD34 20250421_162258_Ch_Chemi+Marker.jpg]

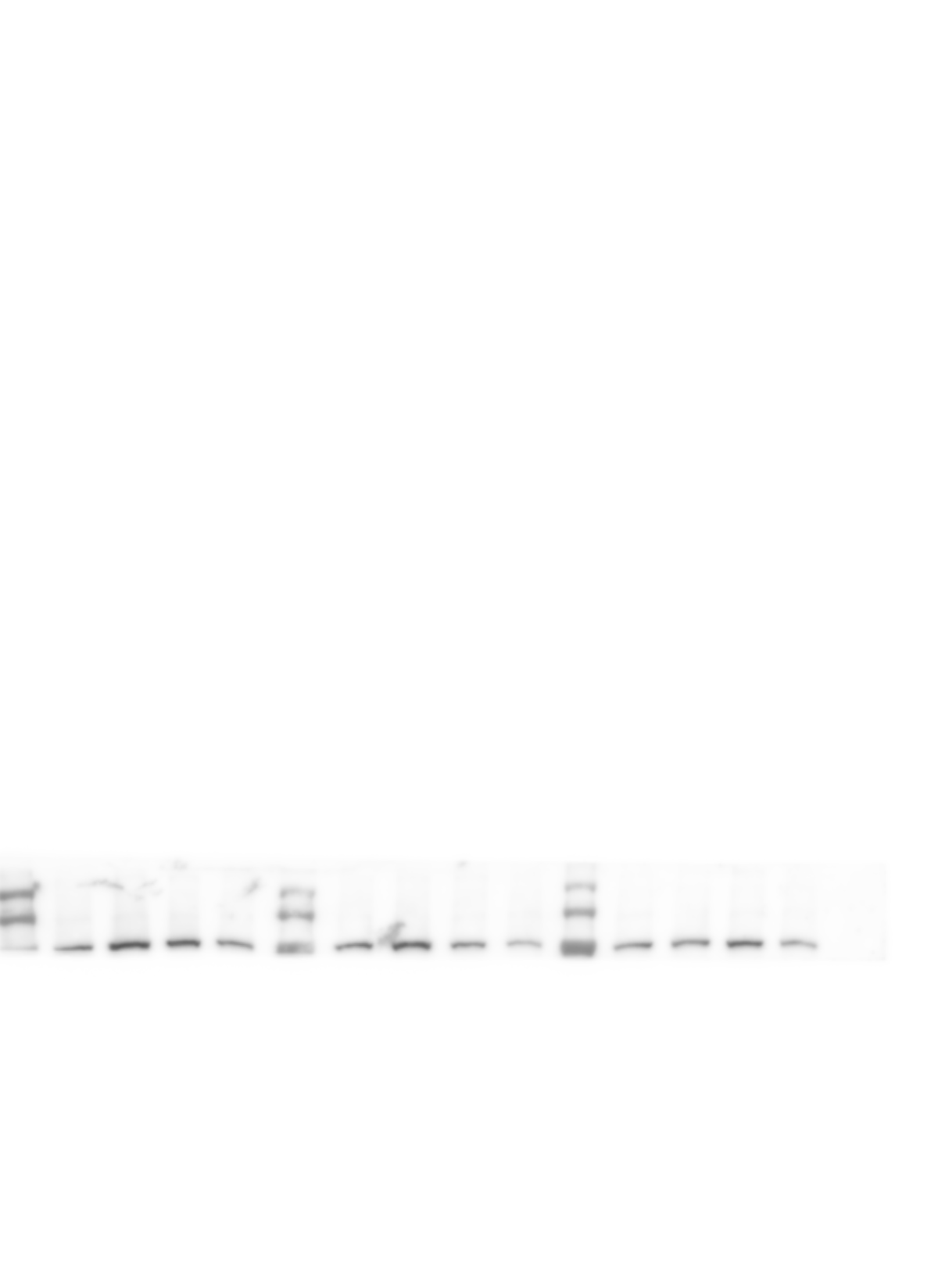

Supplement: Supplementary file 1 [file vetsci-12-01186-s001.zip › Supplementary Files/WB uncropped figure/Figure S3/GADD34 20250421_162258_Ch/GADD34 20250421_162258_Ch_Chemi.tif]

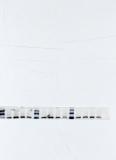

Supplement: Supplementary file 1 [file vetsci-12-01186-s001.zip › Supplementary Files/WB uncropped figure/Figure S3/GADD34 20250421_162258_Ch/GADD34 20250421_162258_Ch_Thumb.jpg]

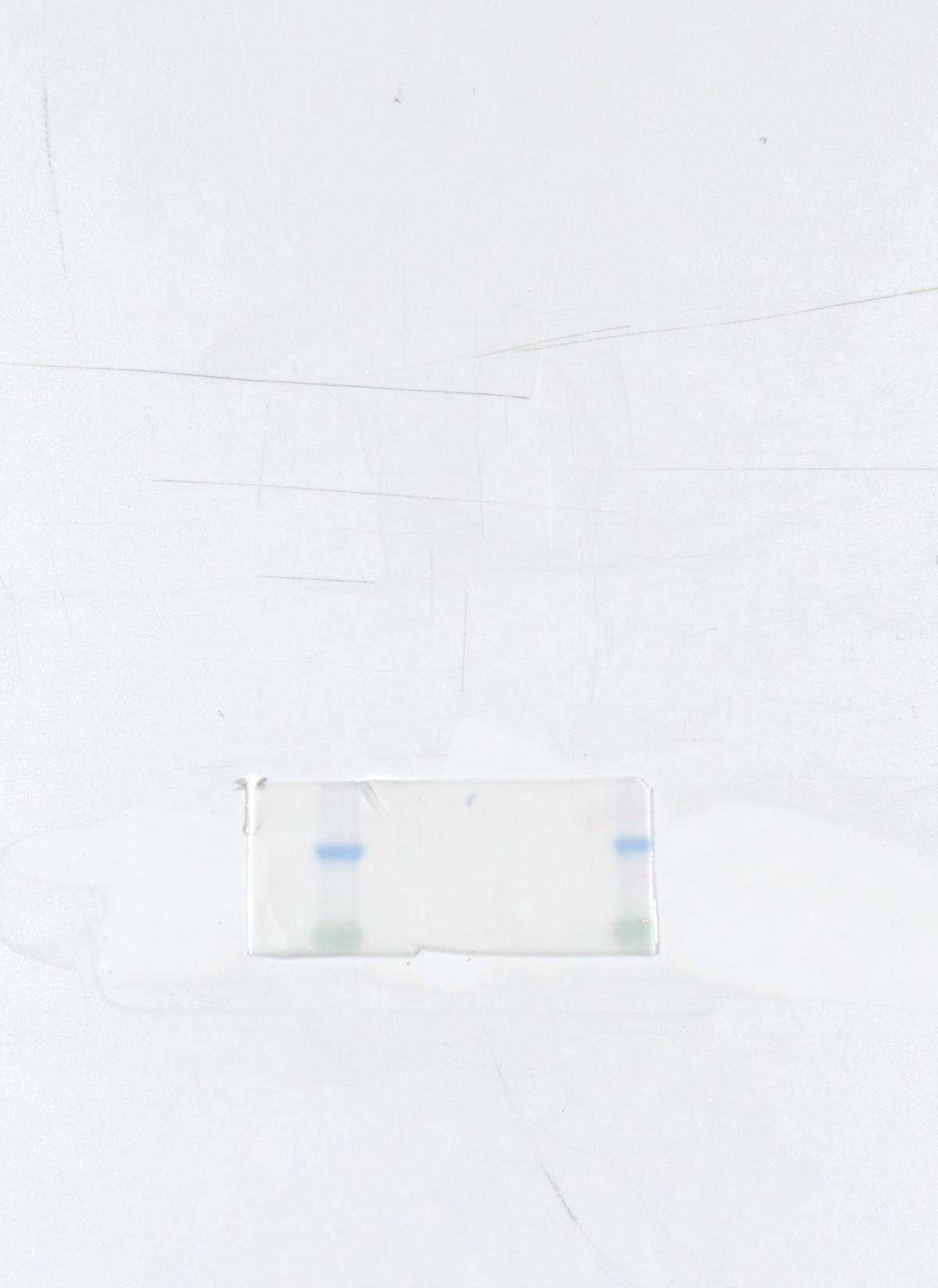

Supplement: Supplementary file 1 [file vetsci-12-01186-s001.zip › Supplementary Files/WB uncropped figure/Figure S3/GAPDH 11 20250426_124325_Ch/GAPDH 11 20250426_124325_Ch-Marker.jpg]

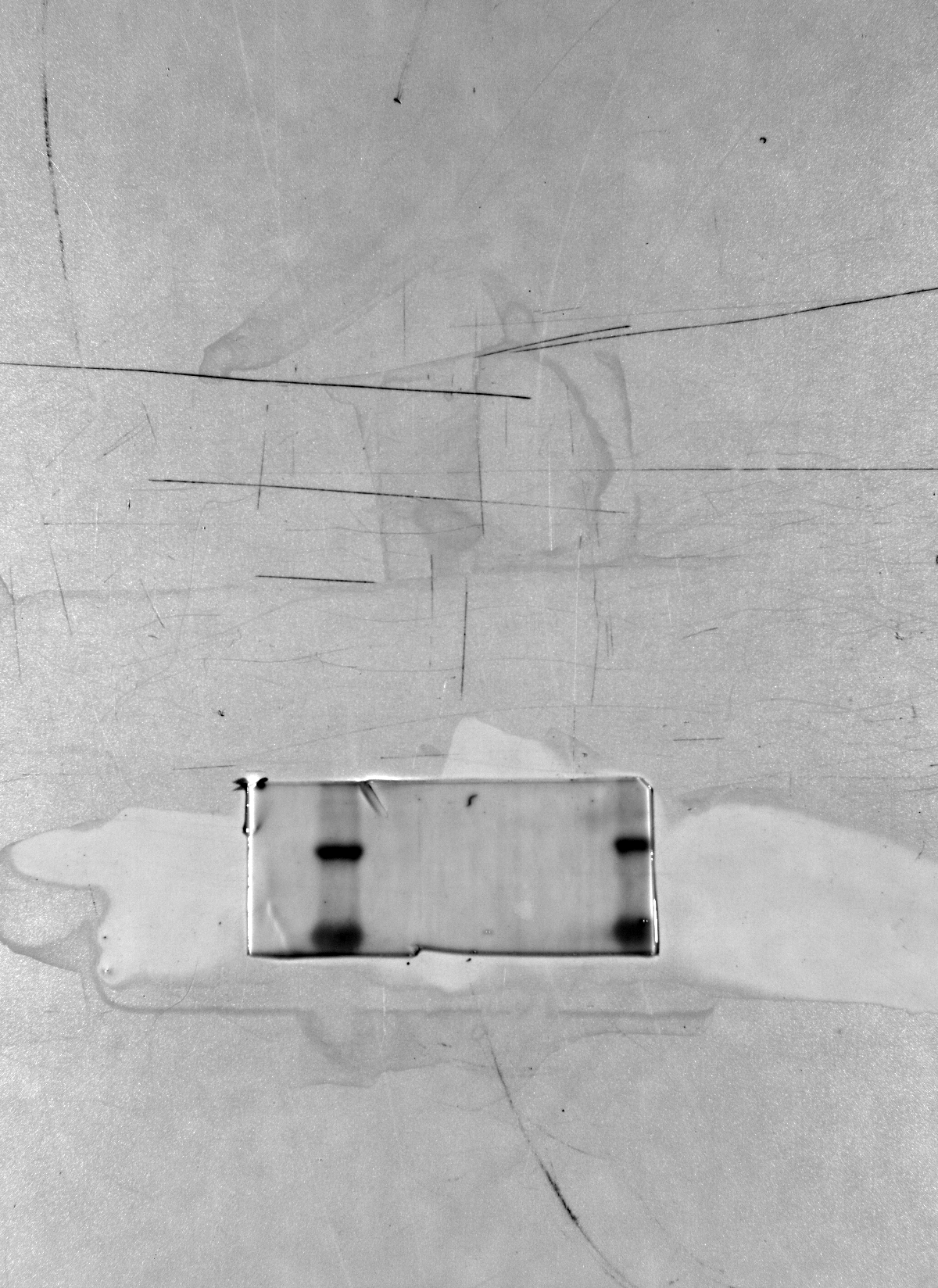

Supplement: Supplementary file 1 [file vetsci-12-01186-s001.zip › Supplementary Files/WB uncropped figure/Figure S3/GAPDH 11 20250426_124325_Ch/GAPDH 11 20250426_124325_Ch-Marker.tif]

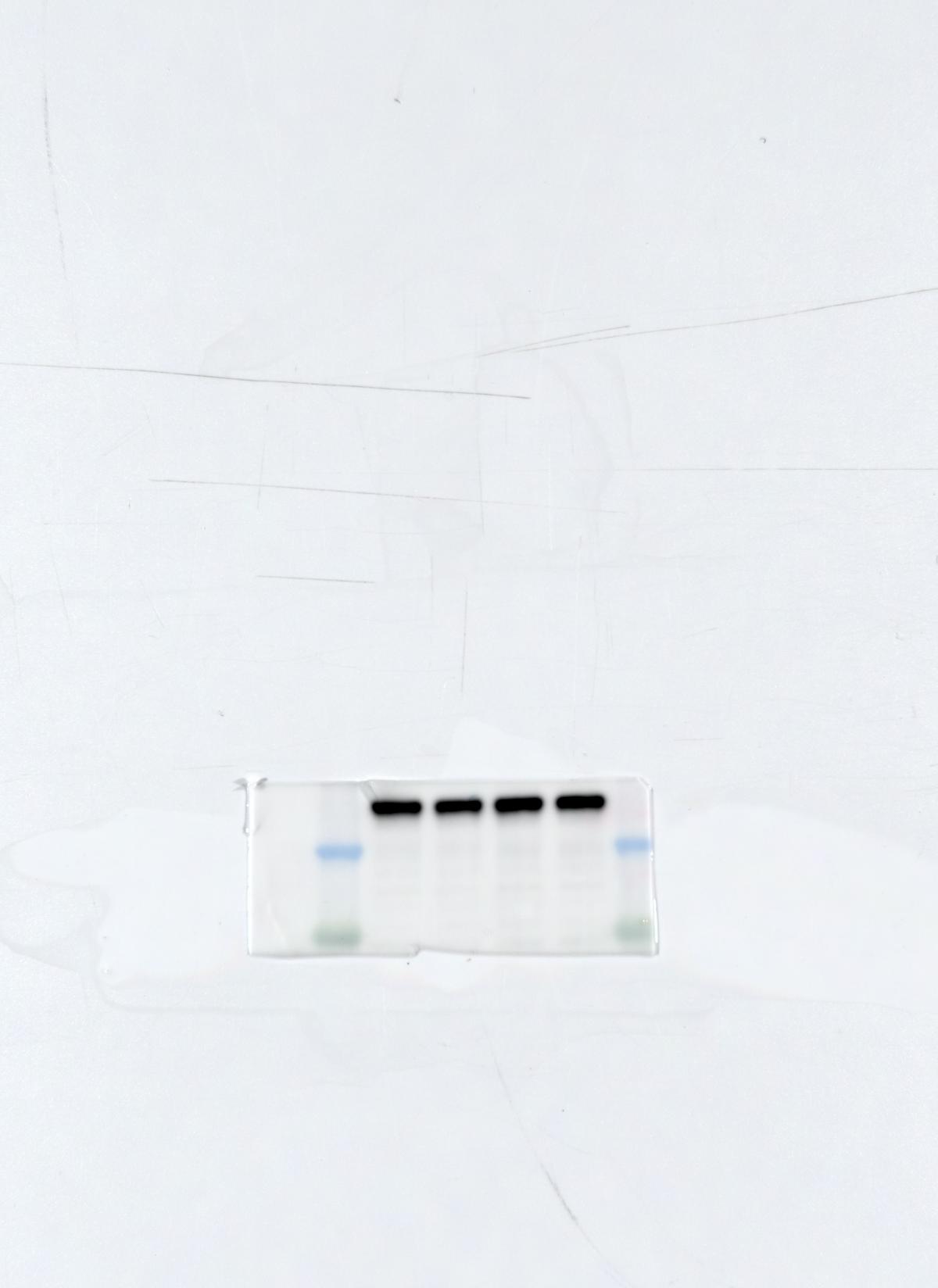

Supplement: Supplementary file 1 [file vetsci-12-01186-s001.zip › Supplementary Files/WB uncropped figure/Figure S3/GAPDH 11 20250426_124325_Ch/GAPDH 11 20250426_124325_Ch_Chemi+Marker.jpg]

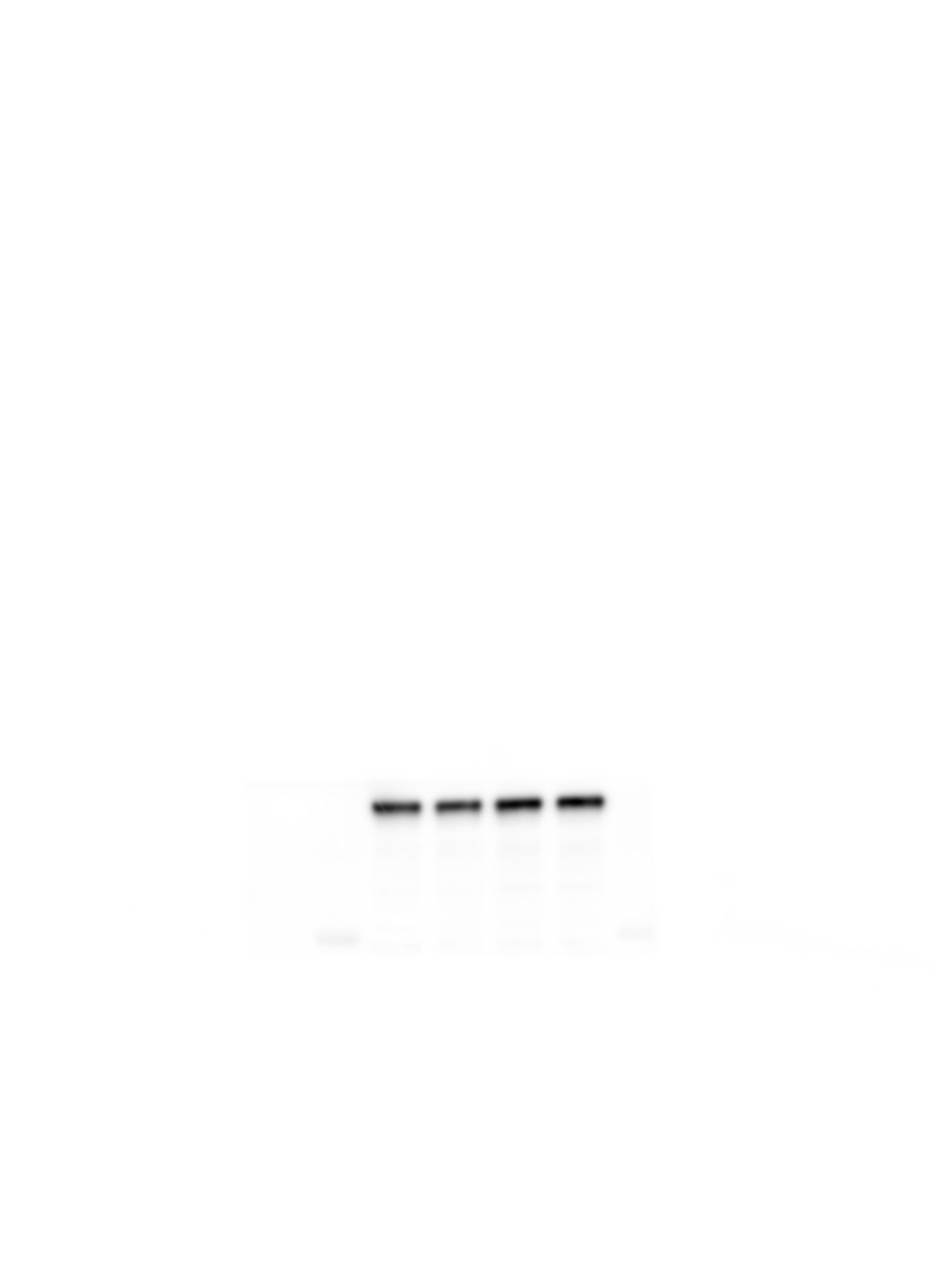

Supplement: Supplementary file 1 [file vetsci-12-01186-s001.zip › Supplementary Files/WB uncropped figure/Figure S3/GAPDH 11 20250426_124325_Ch/GAPDH 11 20250426_124325_Ch_Chemi.tif]

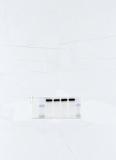

Supplement: Supplementary file 1 [file vetsci-12-01186-s001.zip › Supplementary Files/WB uncropped figure/Figure S3/GAPDH 11 20250426_124325_Ch/GAPDH 11 20250426_124325_Ch_Thumb.jpg]
